# Synthesis and Antiviral Evaluation of Nucleoside Analogues Bearing One Pyrimidine Moiety and Two D-Ribofuranosyl Residues

**DOI:** 10.3390/molecules26123678

**Published:** 2021-06-16

**Authors:** Olga V. Andreeva, Bulat F. Garifullin, Vladimir V. Zarubaev, Alexander V. Slita, Iana L. Yesaulkova, Alexandrina S. Volobueva, Mayya G. Belenok, Maria A. Man’kova, Liliya F. Saifina, Marina M. Shulaeva, Alexandra D. Voloshina, Anna P. Lyubina, Vyacheslav E. Semenov, Vladimir E. Kataev

**Affiliations:** 1 Arbuzov Institute of Organic and Physical Chemistry, Federal Research Center “Kazan Scientific Center of Russian Academy of Sciences”, Arbuzov 8, 420088 Kazan, Russia; andreeva@iopc.ru (O.V.A.); garifullin.bulat@iopc.ru (B.F.G.); maya@iopc.ru (M.G.B.); mankovamaria98@gmail.com (M.A.M.); lsayfina@iopc.ru (L.F.S.); mshulaeva@iopc.ru (M.M.S.); microbi@iopc.ru (A.D.V.); aplyubina@gmail.com (A.P.L.); 2 Pasteur Institute of Epidemiology and Microbiology, Mira 14, 197101 Saint Petersburg, Russia; zarubaev@gmail.com (V.V.Z.); a_slita@yahoo.com (A.V.S.); Yesaulkova@gmail.com (I.L.Y.); sasha-khrupina@mail.ru (A.S.V.)

**Keywords:** nucleoside analogues, antivirals, 1,2,3-triazole, influenza virus, coxsackievirus, click chemistry

## Abstract

A series of 1,2,3-triazolyl nucleoside analogues in which 1,2,3-triazol-4-yl-β-d-ribofuranosyl fragments are attached via polymethylene linkers to both nitrogen atoms of the heterocycle moiety (uracil, 6-methyluracil, thymine, quinazoline-2,4-dione, alloxazine) or to the C-5 and *N*-3 atoms of the 6-methyluracil moiety was synthesized. All compounds synthesized were evaluated for antiviral activity against influenza virus A/PR/8/34/(H1N1) and coxsackievirus B3. Antiviral assays revealed three compounds, **2i**, **5i**, **11c**, which showed moderate activity against influenza virus A H1N1 with IC_50_ values of 57.5 µM, 24.3 µM, and 29.2 µM, respectively. In the first two nucleoside analogues, 1,2,3-triazol-4-yl-β-d-ribofuranosyl fragments are attached via butylene linkers to *N*-1 and *N*-3 atoms of the heterocycle moiety (6-methyluracil and alloxazine, respectively). In nucleoside analogue **11c**, two 1,2,3-triazol-4-yl-2′,3′,5′-tri-*O*-acetyl-β-d-ribofuranose fragments are attached via propylene linkers to the C-5 and *N*-3 atoms of the 6-methyluracil moiety. Almost all synthesized 1,2,3-triazolyl nucleoside analogues showed no antiviral activity against the coxsackie B3 virus. Two exceptions are 1,2,3-triazolyl nucleoside analogs **2f** and **5f**, in which 1,2,3-triazol-4-yl-2′,3′,5′-tri-*O*-acetyl-β-d-ribofuranose fragments are attached to the C-5 and *N*-3 atoms of the heterocycle moiety (6-methyluracil and alloxazine respectively). These compounds exhibited high antiviral potency against the coxsackie B3 virus with IC_50_ values of 12.4 and 11.3 µM, respectively, although both were inactive against influenza virus A H1N1. According to theoretical calculations, the antiviral activity of the 1,2,3-triazolyl nucleoside analogues **2i**, **5i**, and **11c** against the H1N1 (A/PR/8/34) influenza virus can be explained by their influence on the functioning of the polymerase acidic protein (PA) of RNA-dependent RNA polymerase (RdRp). As to the antiviral activity of nucleoside analogs **2f** and **5f** against coxsackievirus B3, it can be explained by their interaction with the coat proteins VP1 and VP2.

## 1. Introduction

**Figure 1:**
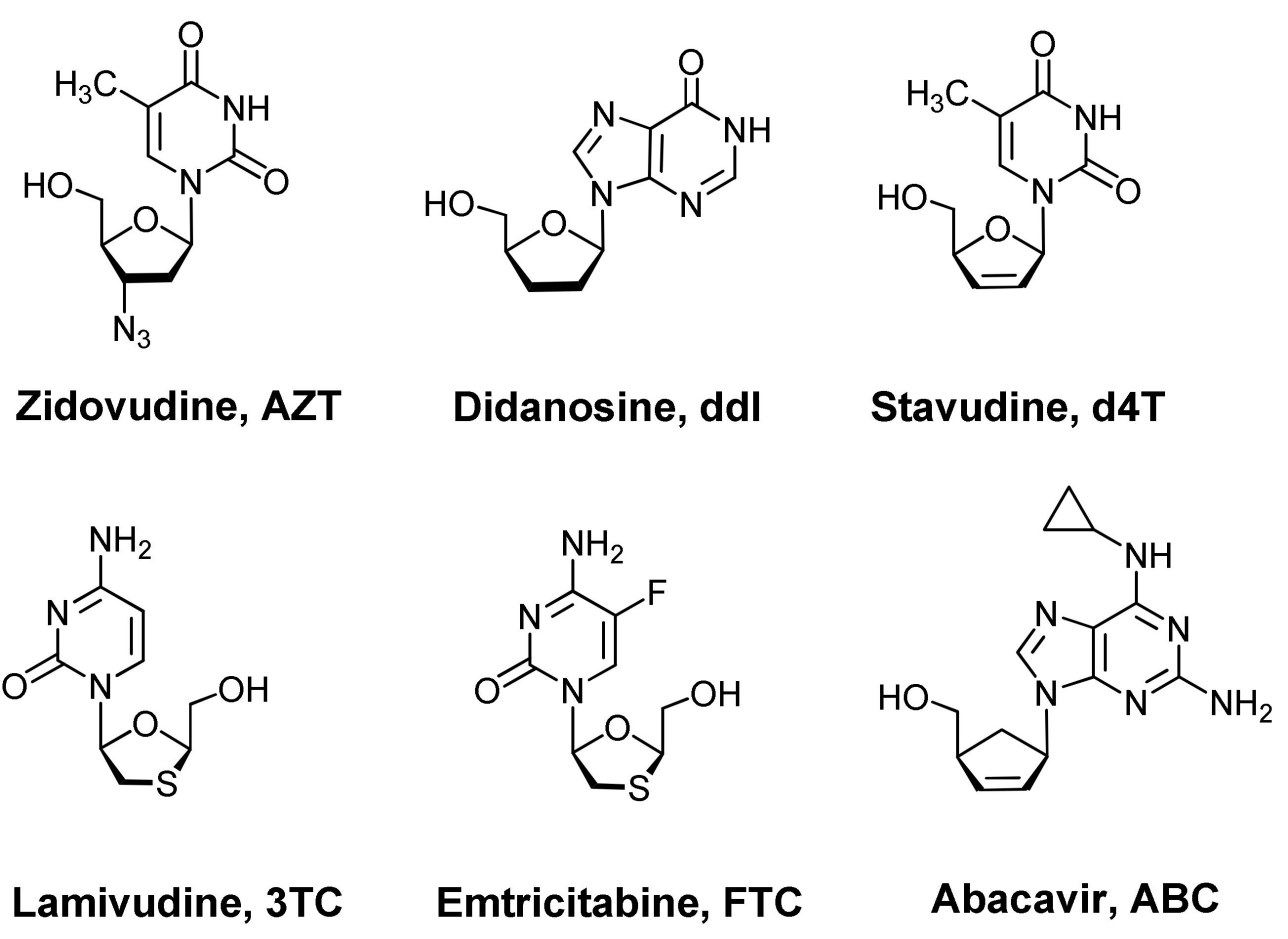
Example of nucleoside analogues—antiretroviral drugs.

Nucleoside analogues represent an efficient scaffold for the development of antiviral drugs. For example, the first six antivirals approved by the FDA (Silver Spring, MD, USA) for the treatment of HIV infection, namely zidovudine, didanosine, stavudine, lamivudine, emtricitabine, abacavir, are synthetic analogues of thymidine, cytidine, adenosine, and guanosine [1]. In general, the following methods are known for obtaining analogues of natural nucleosides: (a) functionalizing a nucleic base with various substituents [2]; (b) the introduction or removal of nitrogen atoms in the nucleic base [3]; (c) annulation to the nucleic base with another heterocycle [2]; (d) replacing the nucleic base with another nitrogen-containing heterocycle [4]; (e) functionalization of the d-ribofuranosyl residue with various substituents [5,6]; (f) removing one or both hydroxyl groups from the C-2′ and C-3′ atoms of the d-ribofuranosyl residue [1,2,6]; (g) removing both hydroxyl groups and hydrogen atoms from the C-2′ and C-3′ atoms of the d-ribofuranosyl residue [1,2]; (h) introducing additional sulfur or oxygen atoms into a d-ribofuranosyl residue [7,8]; (i) replacement of the d-ribofuranosyl residue with a cyclopentane or cyclopentene ring [1,2]; (j) phosphorylation of the hydroxyl group at the C-5′ atom of the d-ribofuranose residue, that is, the transition to nucleotides [9]; (k) replacement of a C-N glycosidic bond with a C-C bond [1,2]; synthesis of l-nucleoside analogues [1,2,7]. However, as a rule, in the synthesis of new nucleoside analogues several of the above methods are used simultaneously. So, for example, didanosine (Figure 1), unlike guanosine, has neither an amino group at the C-2 atom nor hydroxyl groups at the C-2′ and C-3′ atoms [1,2]. Abacavir (Figure 1), in comparison with adenosine, underwent both a modification of the adenine moiety (an amino group appeared at the C-2 position and a cyclopropane ring was attached to the amino group at the C-6 atom) and the sugar residue (the d-ribofuranosyl moiety was replaced by a cyclopentene ring) [1,2].

The emergence of click-chemistry reactions attracted the attention of medical chemists to 1,2,3-triazole as a bridge group, which easily could connect two pharmacophores to form a new biologically active molecule [10]. In addition, 1,2,3-triazole itself is a pharmacophore and is present in many drugs [11]. In recent years, nucleoside analogues with substituted 1,2,3-triazole rings in the nucleic base [3,12,13,14] in the d-ribofuranosyl residue [5,15,16] as well as nucleoside analogues in which the nucleic base was coupled to the d-ribofuranosyl residue through a 1,2,3-triazole bridge have been synthesized [17,18,19].

**Figure 2:**
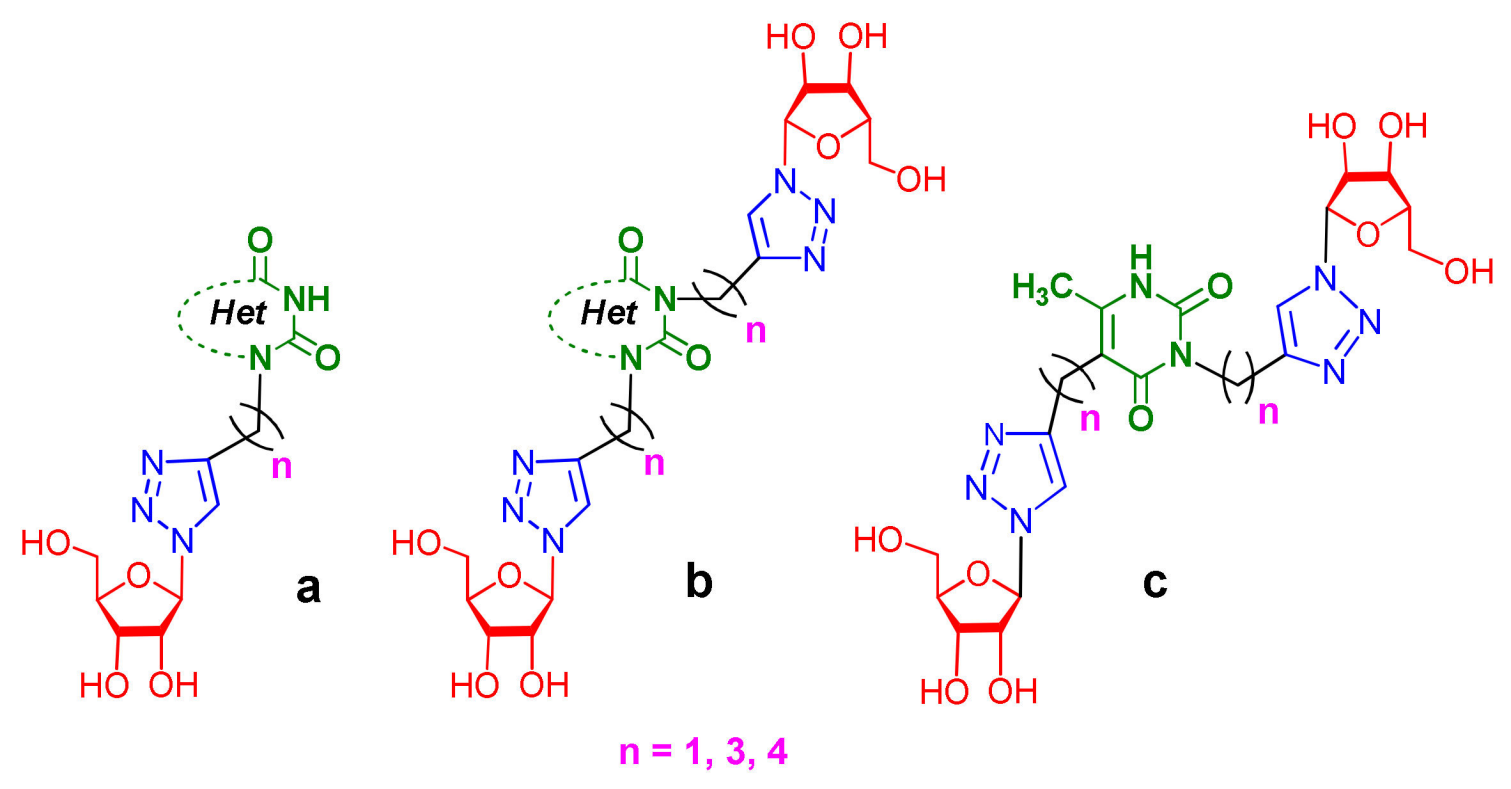
Nucleoside analogues being investigated by our group: (**a**) already obtained and evaluated compounds [20,21]; (**b,c**) the target compounds of this study. Het = uracil, 6-methyluracil, thymine, quinazoline-2,4-dione, alloxazine.

Recently, we synthesized nucleoside analogues in which a 1,2,3-triazol-4-yl-β-d-ribofuranosyl fragment was attached to the pyrimidine moiety (uracil, 6-methyluracil, thymine, quinazoline-2,4-dione) through a polymethylene linker of variable length [20,21] (Figure 2). Biological testing has revealed several compounds showing moderate (IC_50_ = 15, 30, 42–48 µM) antiviral activity against the influenza A (H1N1) virus. Herein, we report on the synthesis and antiviral evaluation of novel nucleoside analogues in which a 1,2,3-triazol-4-yl-β-d-ribofuranosyl fragment is attached via a polymethylene linker of variable length to both nitrogen atoms of the pyrimidine moiety (uracil, 6-methyluracil, thymine, quinazoline-2,4-dione, alloxazine) or to the C-5 and *N*-3 atoms of the 6-methyluracil moiety (Figure 2).

## 2. Results and Discussion

### 2.1. Chemistry

The strategy for the synthesis of the target nucleoside analogues (Figure 2) was based upon the copper-catalyzed alkyne–azide cycloaddition (CuAAC) reaction and was carried out according to a convergent scheme consisting of pyrimidine and carbohydrate routes completed with the formation of alkyne precursors related to alkynyl substituted pyrimidines and an azide precursor related to β-d-ribofuranose, respectively. At the final stage, the precursors were coupled by a CuAAC reaction.

**Scheme 1:**
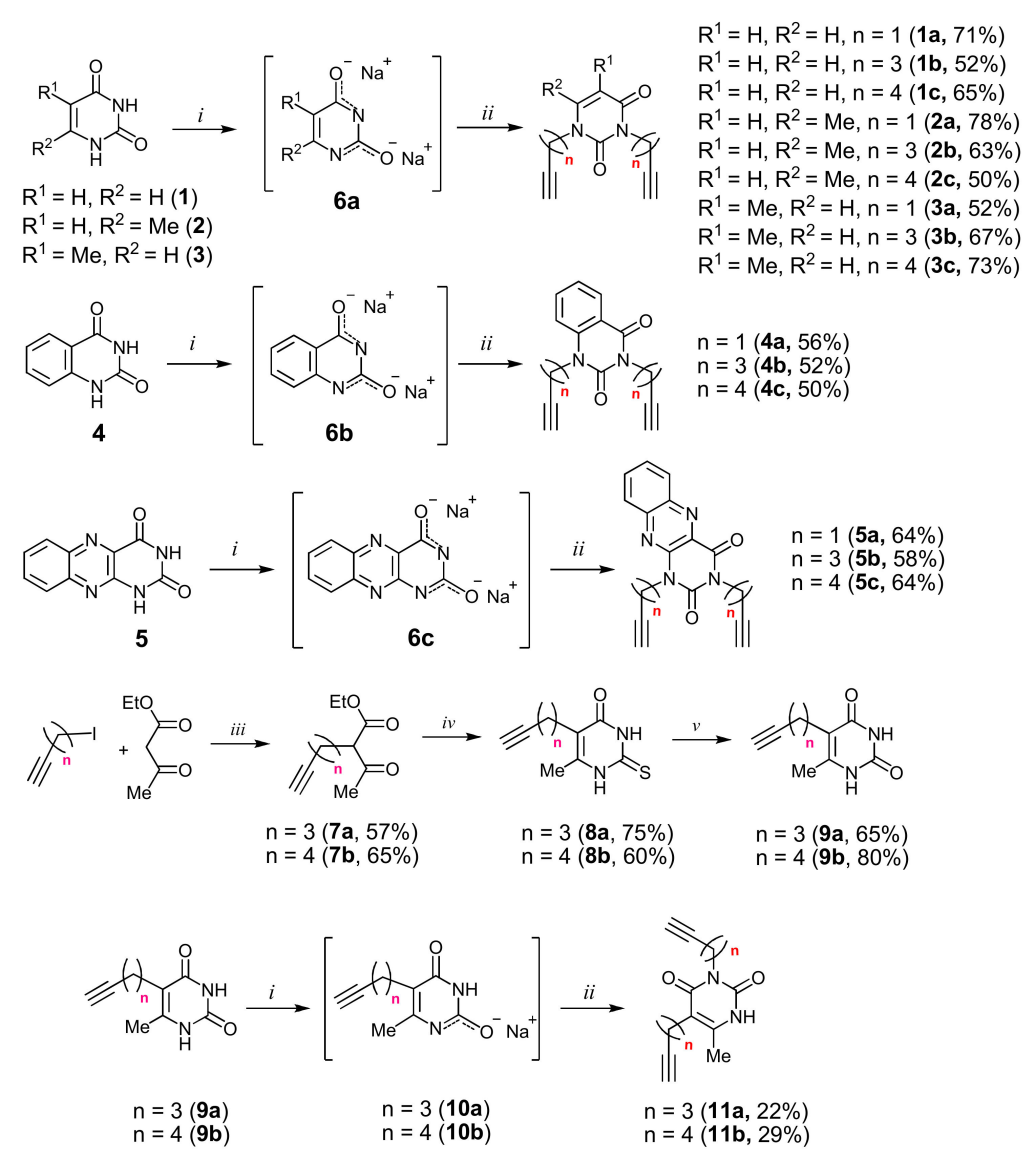
Synthesis of 1,3- and 3,5-bis(α,ω-alkinyl)pyrimidines. Reagents and conditions: (*i*) NaH, DMF, 67–70 °C, 8 h; (*ii*) HC≡C-(CH_2_)_n_-Hlg, DMF, 115–120 °C, 16 h; (*iii*) NaOEt, EtOH, 0–78 °C, 10 h; (*iv*) thiourea, K_2_CO_3_, MeOH, reflux, 15 h; (*v*) ClCH_2_COOH, H_2_O, reflux, 20 h.

Alkyne precursors related to alkynyl-substituted pyrimidines were prepared according to the synthetic routes summarized in Scheme 1. Uracil **1**, 6-methyluracil **2**, thymine **3**, quinazoline-2,4-dione **4**, and alloxazine **5** were converted with the excess of sodium hydride into corresponding disodium salts **6**, which were alkylated with propargyl bromide, 5-iodo-pent-1-yne, and 6-iodo-hex-1-yne without any purification to afford 1,3-bis(α,ω-alkynyl)derivatives of uracil **1a**–**c**, 6-methyluracil **2a,b,c**, thymine **3a,b,c**, quinazoline-2,4-dione **4a,b,c**, and alloxazine **5a,b,c**. Subsequently, 3,5-bis(α,ω-alkynyl)-6-methyluracils **11a,b** were prepared in two steps. At the first stage, acetoacetic ester was reacted with 5-iodo-1-pentyne or 6-iodo-1-hexyne to afford 3-(α,ω-alkyne)acetoacetic esters **7**, condensation of which with thiourea provided 2-thio-5-(α,ω-alkynyl)-6-methyluracils **8**. The hydrolysis of thiopyrimidines **8** with an aqueous solution of chloroacetic acid led to 5-alkynyl-6-methyluracils **9a,b** in 65–80% yields. At the second stage, pyrimidines **9** were converted to monosodium salts **10**, which were alkylated in situ with 5-iodo-1-pentyne and 6-iodo-1-hexyne to give 3,5-bis(α,ω-alkynyl)-6-methyluracils **11a,b** with 22–29% yields (Scheme 1).

**Scheme 2:**
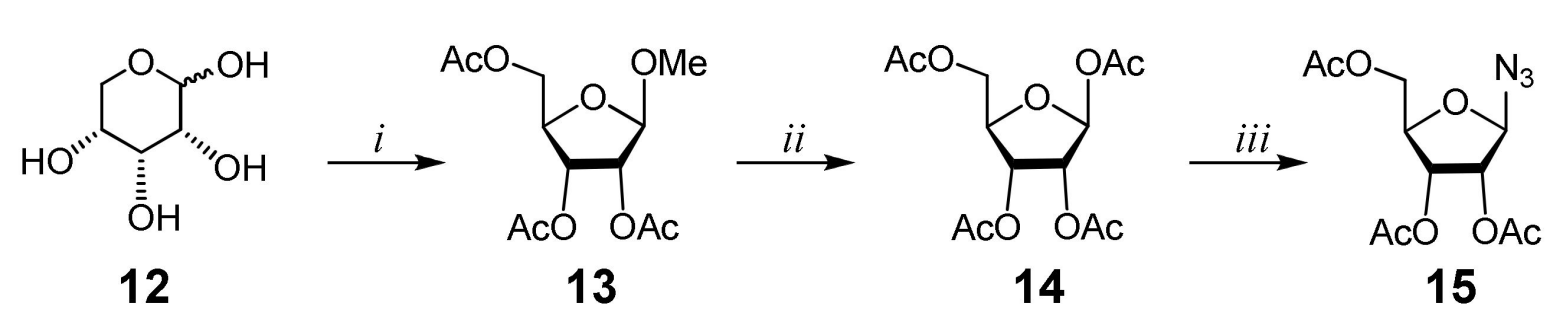
Synthesis of azido 2,3,5-tri-*O*-acetyl-β-d-ribofuranoside. Reagents and conditions: (*i*) a. MeOH, H_2_SO_4_, r.t., 6 h, Na_2_CO_3_; b. Py, Ac_2_O, r.t., 24 h (42%); (*ii*) Ac_2_O, AcOH, 5% H_2_SO_4_, 0 °C, 3 h, NaHCO_3_ (43%); (*iii*) TMSN_3_, SnCl_4_, r.t., 24 h (95%).

Afterward, 2,3,5-tri-*O*-acetyl-β-d-ribofuranosylazide **15** (Scheme 2), whose preparation has recently been reported by our group [20,21], was used as the azide precursor of a CuAAC reaction. Commercially available d-ribose **12** was reacted with MeOH to afford methyl d-α/β-ribofuranoside, which was immediately acetylated to obtain a mixture of α- and β-anomers of methyl-2,3,5-tri-*O*-acetyl-d-ribofuranoside **13**, which were separated through silica gel flash column chromatography. The methoxy group of the β-anomer of monosaccharide **13** was then replaced by an acetoxy group by the reaction with a mixture of glacial acetic acid and Ac_2_O in the presence of H_2_SO_4_. The obtained 1,2,3,5-tetra-*O*-acetyl-β-d-ribofuranoside **14** was treated with trimethylsilyl azide (TMSN_3_) in the presence of tin tetrachloride to provide the azide precursor **15** in 95% yield (Scheme 2).

**Scheme 3:**
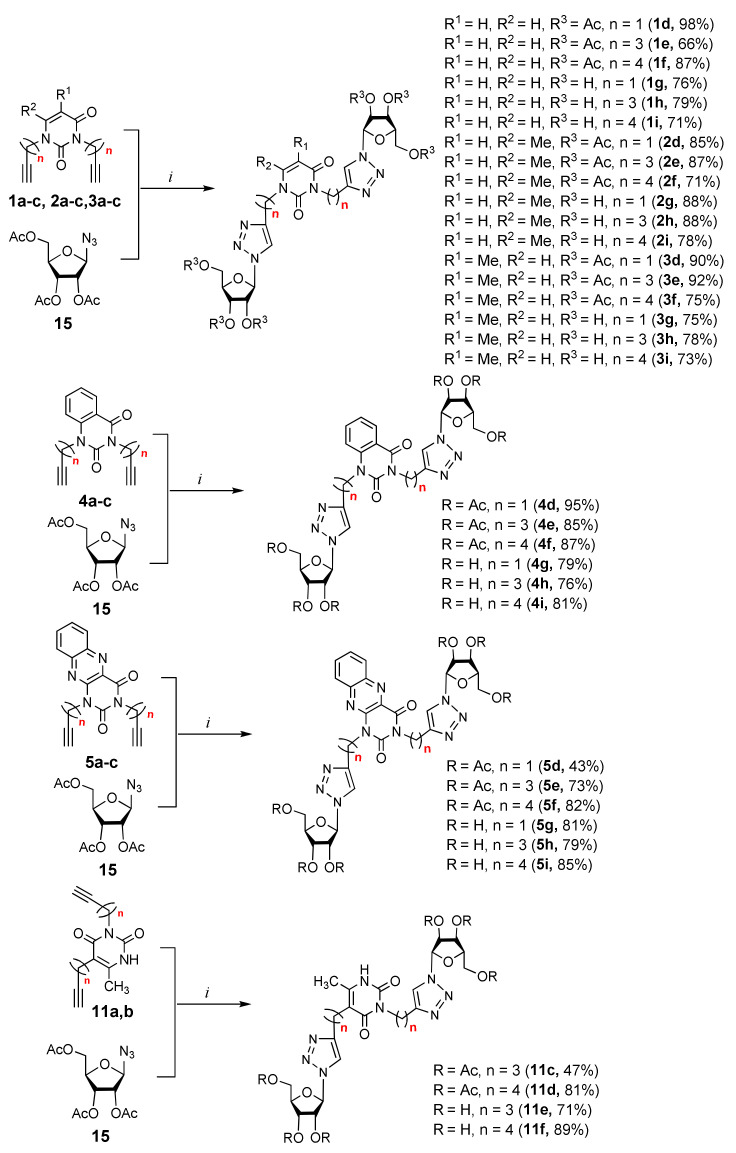
Synthesis of the target nucleoside analogues. Reagents and conditions: (*i*) a. *tert*-BuOH/H_2_O, CuSO_4_·5H_2_O, Na l-ascorbate, 40 °C, 48 h; b. MeOH, MeONa, Amberlist^®^15H+, r.t.

Finally, a CuAAC reaction was utilized to complete the convergent scheme and couple alkyne precursors **1a**–**c**, **2a**–**c**, **3a**–**c**, **4a**–**c**, **5a**–**c**, **11a,b** with azide precursor **15** to afford nucleoside analogues **1d**–**f**, **2d**–**f**, **3d**–**f**, **4d**–**f**, **5d**–**f**, **11c,d** bearing one pyrimidine moiety and two 1,2,3-triazol-4-yl-β-d-ribofuranosyl fragments with protected hydroxyl groups (Scheme 3). Removal of the acetyl protecting groups in a methanolic sodium methoxide solution provided the target nucleoside analogues **1g**–**i**, **2g**–**i**, **3g**–**i**, **4g**–**i**, **5g**–**i**, **11e,f** having sugar free hydroxyls (Scheme 3). 

Procedures for the synthesis of the compounds, characterization of the compounds synthesized are detalized in Supplementary Materials. NMR spectra of the compounds are also shown there, Figures S1–S96.

### 2.2. Antiviral Evaluation

Several synthesized 1,2,3-triazolyl nucleoside analogues were evaluated for their in vitro antiviral activity against the A/Puerto Rico/8/34 (H1N1) influenza virus (for MDCK cells) and coxsackievirus B3 (for Vero cells). The resulting data expressed as virus-inhibiting activity (IC_50_), cytotoxicity (CC_50_), as well as selectivity index (SI), which is the ratio CC_50_/IC_50_, are presented in Table 1 and Table 2. The data of Table 1 demonstrate that the antiviral activity against influenza virus A H1N1 depends more on the length of the linker connecting the heterocyclic moiety to the 1,2,3-triazol-4-yl-β-d-ribofuranosyl fragments than on the nature of the heterocycle. Both compounds **2i** and **5i**, in which the heterocyclic moiety is coupled to the 1,2,3-triazol-4-yl-β-d-ribofuranosyl fragments through butylene linkers, exhibited moderate activity (IC_50_ values 57.5 µM and 24.3 µM, respectively) in contrast to compounds **2g**, **4g**, **5g** and **2h**, **4h**, **5h** possessing methylene or propylene linkers, respectively, which were found to be inactive (IC_50_ > 100 µM). Thus, both in the series of compounds **2g**, **2h**, **2f**, **2i** possessing 6-methyluracil as the heterocyclic moiety and in the series of compounds **5g**, **5h**, **5f**, **5i** possessing alloxazine as the heterocyclic moiety with an increase in the length of polymethylene linkers, the antiviral activity increased by 9 and 7 times, respectively. The dependence of the antiviral activity of compounds **2g**, **2h**, **2f**, **2i** and **5g**, **5h**, **5f**, **5i** on the nature of the heterocyclic moiety was less pronounced, but present. The antiviral activity of the lead compound **5i** possessing the alloxazine moiety coupled to the 1,2,3-triazol-4-yl-β-d-ribofuranosyl fragments via butylene linkers (IC_50_ = 24.3 μM, SI = 17) exceeded the antiviral activity of compound **2i**, which possessed the 6-methyluracil moiety coupled to the 1,2,3-triazol-4-yl-β-d-ribofuranosyl fragments also via butylene linkers (IC_50_ = 57.5 µM, SI = 8) twice. That is, the increase in the size and the lipophilicity of the heterocyclic moiety of 1,2,3-triazole nucleoside analogues upon passing from compound **2i** to compound **5i** was accompanied by an increase in antiviral activity. However, this pattern was violated by compound **4i**, which has the quinazoline-2,4-dione moiety coupled to the 1,2,3-triazol-4-yl-β-d-ribofuranosyl fragments also via butylene linkers.

Since the size and the lipophilicity of quinazoline-2,4-dione is average between the sizes and the lipophilicities of 6-methyluracil and alloxazin, it could be assumed that compound **4i** has to show the antiviral activity in the range between the activities of compound **2i** possessing the 6-methyluracil moiety and compound **5i** possessing the alloxazine moiety. However, compound **4i** was found to be inactive against influenza virus A H1N1 (IC_50_ = 297 µM). At the same time, it should be noted that the analogue of compound **4i** with only one 1,2,3-triazolylribofuranosyl fragment attached to the *N*-1 atom of the quinazoline-2,4-dione moiety, namely, compound **4m**, showed moderate antiviral activity against the H1N1 influenza virus (IC_50_ = 30 µM, Table 1, [22]). This fact indicates the significant dependence of the antiviral activity of 1,2,3-triazole nucleoside analogues against the influenza A H1N1 virus on the attachment of the second 1,2,3-triazol-4-yl-β-d-ribofuranosyl fragment to the heterocyclic moiety via a polymethylene linker. Indeed, the addition of the second 1,2,3-triazol-4-yl-β-d-ribofuranosyl fragment to the 6-methyluracil moiety of nucleoside analogue **2k** with one 1,2,3-triazol-4-yl-β-d-ribofuranosyl fragment, showed moderate antiviral activity (IC_50_ = 43 µM [22]), that is, the transition from compound **2k** to compound **2h** led to a complete loss of activity (IC_50_ > 492.9 µM, Table 1). A similar loss of activity occurred in the result of the addition of the second 1,2,3-triazol-4-yl-β-d-ribofuranosyl fragment to the nucleoside analogues **4j**, **4m**, **11h,** which showed moderate antiviral activity (the values of IC_50_ are 42, 30, and 15 μM, respectively, Table 1). The IC_50_ values of their derivatives **4g**, **4i**, **11e** possessing two 1,2,3-triazolylribofuranosyl fragments are 180.1, 297.3, and 164.3 μM, respectively (Table 1). However, the addition of the second 1,2,3-triazol-4-yl-β-d-ribofuranosyl fragment to the heterocyclic moiety of nucleoside analogues **2j**, **2m**, **4k**, **11i**, **11j**, that is, the transition to disubstituted nucleoside analogues **2g**, **2i**, **4h**, **11d**, **11f**, did not cause changes in the antiviral activity (Table 1). Disubstituted nucleoside analogues **2g**, **4h**, **11d**, **11f** did not demonstrate antiviral activity against the influenza A H1N1 virus just like monosubstituted nucleoside analogues **2j**, **4k**, **11i**, **11j**, and disubstituted nucleoside analogue **2i** showed approximately the same moderate activity (IC_50_ = 57.5 μM) as its monosubstituted precursor **2m** (IC_50_ = 48 μM, Table 1).

Unexpectedly, antiviral activity against the influenza A H1N1 virus (IC_50_ = 29.2 µM, CC_50_ > 349 µM, SI = 12) was demonstrated by nucleoside analogue **11c** with protected (acylated) hydroxyl groups of the sugar residue (Table 1). Moreover, its derivative **11e** possessing free hydroxyl groups turned out to be completely inactive (Table 1). It should be noted that other nucleoside analogues with protected hydroxyl groups **2f**, **4f**, **5f**, **11d** evaluated in this study were also inactive against the influenza A H1N1 virus. So, for reasons so far unclear, compound **11c** was the only one among the investigated nucleoside analogues with two 1,2,3-triazolyl-2′,3′,5′-tri-*O*-acetyl-ribofuranosyl fragments that showed the same antiviral activity against the influenza A H1N1virus as compound **5i** with two 1,2,3-triazol-4-yl-β-d-ribofuranosyl fragments with free hydroxyl groups. A similar case was described earlier [22] when we found that the analogue of compound **11c** with one 1,2,3-triazolyl-2′,3′,5′-tri-*O*-acetyl-β-d-ribofuranosyl fragment attached via a propylene linker to the C-5 atom of the 6-methyluracil moiety (compound **11g** in Table 1) was able to inhibit in vitro replication of the influenza A H1N1 virus and the coxsackie B3 virus with IC_50_ values of 67 and 9 µM, respectively.

The results of an in vitro study of cytotoxic and antiviral properties of several synthesized compounds against the coxsackie B3 virus (for Vero cells) are presented in Table 2. It can be seen that almost all the compounds studied were inactive against this virus. Two exceptions are 1,2,3-triazolyl nucleoside analogues **2f** and **5f**, in which the heterocyclic moiety (6-methyluracil in the case of **2f** and alloxazine in the case of **5f**) is coupled to 1,2,3-triazol-4-yl-2′,3′,5′-tri-*O*-acetyl-β-d-ribofuranosyl fragments via butylene linkers. These compounds exhibited high antiviral potency against the coxsackie B3 virus, with IC_50_ values of 12.4 and 11.3 µM, respectively (Table 2), although both were inactive against influenza virus A H1N1 (Table 1). Moreover, these 1,2,3-triazolyl nucleoside analogues showed very high cytotoxicity (CC_50_ = 18 µM) against Vero cells that were used as a cell host for the growth of the coxsackie B3 virus, that is, infected cells were dying almost immediately as soon as the inhibition of virus replication began (Table 2). Interestingly, these compounds possessing protected hydroxyl groups of β-d-ribofuranosyl residues as well as their derivatives **2i** and **5i** with free hydroxyl groups appeared to be not cytotoxic against human cancer cell lines M-HeLa, HuTu-80, PC-3, normal cell line WI-38, and the MDCK cell line, which was used as a cell host to grow the influenza A H1N1 virus (Table 1 and Table 3). By the way, analogues of nucleosides **2i** and **5i** with free hydroxyl groups of ribofuranosyl residues, in contrast to their precursors **2f** and **5f**, were not cytotoxic also against the Vero cell line (Table 2).

### 2.3. Molecular Docking

#### 2.3.1. Influenza Virus A H1N1

Of all drug targets of influenza virus A H1N1, like all other RNA viruses, including SARS-CoV-2, RNA-dependent RNA polymerase (RdRp) is the most promising drug target since it plays a critical role in RNA viruses replication [24,25]. The RdRp consists of three separate polypeptides called polymerase basic 2 (PB2), polymerase basic 1 (PB1), and polymerase acidic (PA) [26]. It has been shown that the antiviral activity of nucleoside analogues is due to interactions with the fragment of PA containing the N-terminal endonuclease domain (PA-Nter) of RdRp [24,27]. Therefore, we decided to test the ability of the lead compounds **2i**, **5i**, **11c** to bind to RdRp of influenza virus A H1N1 in molecular docking simulations described in Supplementary Materials [28,29,30,31,32,33]. As a potential target we chose the ligand-binding domain (LBD) of acidic polymerase (PA) of RdRp. The PA-Nter domain has a cation-dependent endonuclease active-site core, the catalytic residues His41, Glu80, Asp108, and Glu119 being conserved among all influenza A subtypes and strains [29,34]. The literature has revealed that cellular kinases and virally encoded kinases metabolize nucleoside analogues to their 5-phosphorylated derivatives [35,36]. At first, nucleoside kinases encoded by the host cell or the virus infecting the host cell catalyze phosphorylation of a nucleoside analogue to its 5-monophosphate form. Subsequently, the transformation of 5-monophosphates to the corresponding 5-diphosphates and triphosphates is carried out by nucleoside, nucleotidyl, and nucleoside diphosphate kinases, respectively [35,36,37]. Accordingly, 5-triposphate (TP) derivatives of the lead compounds **2i-TP** and **5i-TP** were also taken into consideration.

**Figure 3:**
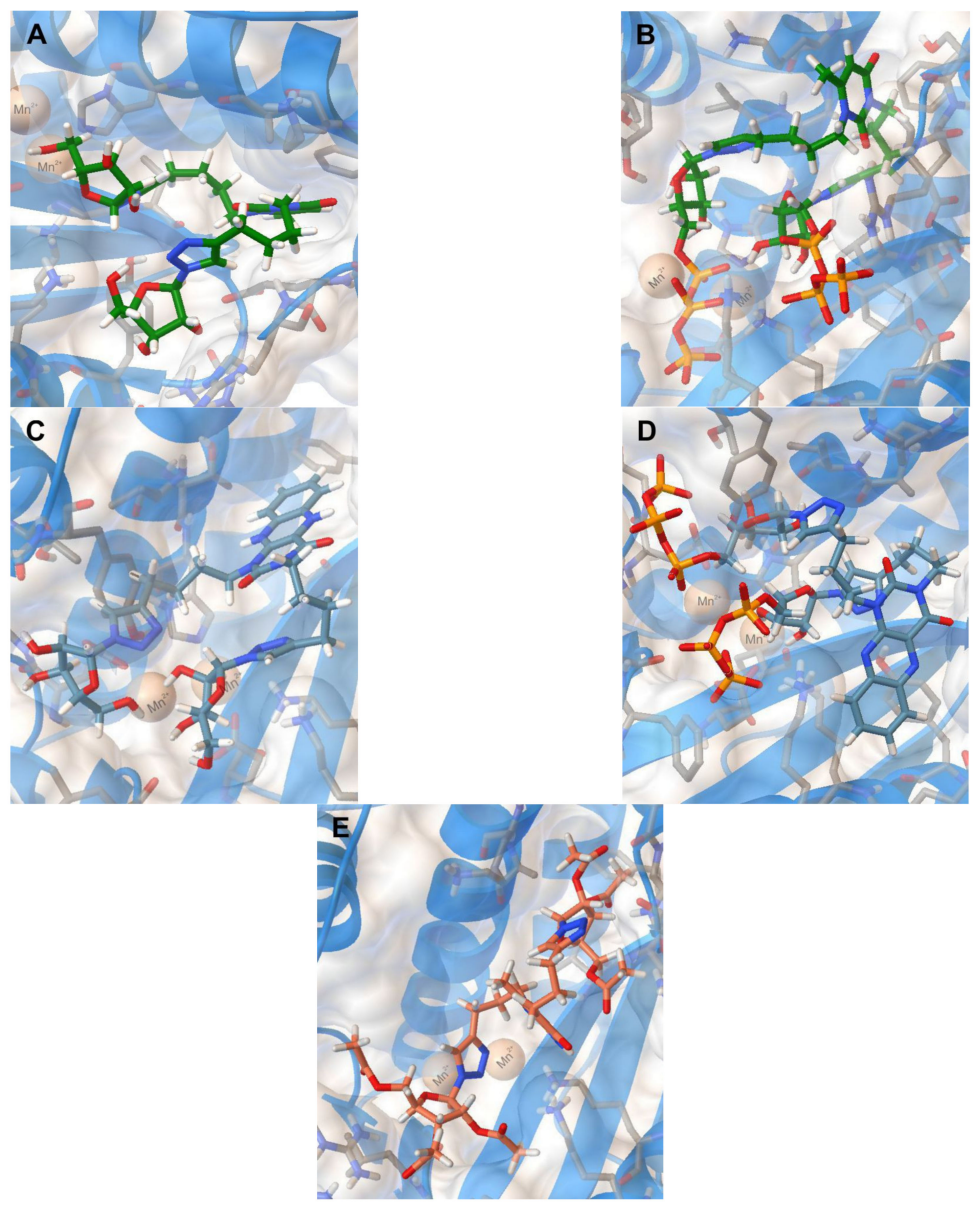
Molecular docking simulations of the optimized docking model of compounds **2i** (**A**), **2i**-**TP** (**B**), **5i** (**C**), **5i-TP** (**D**), **11c** (**E**) in the PA-Nter (PDB code 5I13) active site obtained in the lowest-energy conformations.

Thus, we have evaluated the binding of **2i**, **2i-TP**, **5i**, **5i-TP**, and **11c** to the N-terminal domain of the RdRp acidic subunit of influenza virus A/Puerto Rico/8/1934/H1N1 [29] (PDB code 5I13). The positions of the optimized docking models of compounds **2i**, **2i-TP**, **5i**, **5i-TP**, and **11c** exhibiting the best binding energies are shown in Figure 3. According to the docking simulations, all studied molecules are located deep in the cavity of the active site of PA N-ter, being approximately in one area of space, but the conformations of compounds **2i**, **2i-TP**, **5i**, **5i-TP** differ significantly from the conformation of compound **11c**. The conformations of the first four compounds are folded with the 1,2,3-triazol-4-yl-β-d-ribofuranosyl fragments oriented in one direction while the conformation of **11c** is unfolded with the 1,2,3-triazol-4-yl-β-d-ribofuranosyl fragments oriented orthogonally to the plane of the 6-methyluracil ring in different directions (Figure 3). Nevertheless, **11c** has interactions with the same protein residues Lys134, Lys137, Arg196, Leu106 as compounds **2i**, **2i-TP**, **5i**, **5i-TP** (Table 4).

The main structural fragments of compounds **2i**, **5i**, and **11c** that ensure their interaction with the PA-N-ter active center are ribofuranosyl residues and 1,2,3-triazole rings. The ribofuranosyl residues of **2i** are linked to the amino groups of the side chains of Lys134 and Lys137 protein residues via hydrogen bonds and the 1,2,3-triazole ring of this compound has H-bonding with the Val122 residue. In the case of compound **5i**, one of the ribofuranosyl moieties has interaction with the Leu106 residue, the other one with the Gly81 residue. The 1,2,3-triazole ring of **5i** has H-bonding with the Tyr24 residue and, in addition, is engaged in the π-cationic interaction with one of the two Mn^2+^ cations located in the active site of the PA protein. The other 1,2,3-triazole ring of **5i** and the protonated amino group of the side chain of Lys134 residue interact via π-cationic interaction. Compound **11c** is retained in the PA-Nter cavity via the π-cationic interaction of the 1,2,3-triazole ring with Mn^2+^ cation and H-bonding of another 1,2,3-triazole ring with the Arg196 protein residue. The H-bonding of the amino acid residues Arg84, Leu106, Lys137 with the carbonyl oxygen atoms of the acetate protecting groups of the ribofuranosyl moiety and H-bonding of the terminal amino group of the Lys134 protein residue with the carbonyl group of the uracil moiety is a feature of the binding of compound **11c** in the active site of PA-Nter. The binding of **2i-TP** and **5i-TP** in the PA-Nter active site is due to the interaction of their phosphate groups with the Lys134, Lys137, Gly81, Arg84 protein residues and the H-bonding of the ribofuranosyl moiety with the Val122 and Tyr23 protein residues. Moreover, one 1,2,3-triazole ring of **2i-TP** is engaged in the π-cationic interaction with the protonated terminal amino group of the Lys34 residue and the 1,2,3-triazole ring of **5i-TP** has the π-π stacking with the Tyr24 protein residue.

According to the molecular docking calculation, **5i** and **5i-TP** have the highest binding energies to the PA-Nter active site among the compounds studied (−9.3 and −9.1 kkal/mol, respectively). The binding energies of **2i** and **11c** are worse (−8.7 and −8.1 kkal/mol, respectively). Thus, the results of the molecular docking calculations approximately correspond to some decrease in antiviral activity against influenza virus A H1N1 in the series of compounds studied: IC_50_ (**5i**) < IC_50_ (**11c**) < IC_50_ (**2i**). Therefore, one can conclude that antiviral activity of the 1,2,3-triazolyl nucleoside analogues **2i**, **5i**, **11c** against the H1N1 (A/PR/8/34) influenza virus can be explained by their influence on the functioning of the polymerase acidic protein of RNA-dependent RNA polymerase.

#### 2.3.2. Coxsackievirus B3

Coxsackievirus B3 belongs to the *Enteroviruses* genus of the *Picornaviridae* family [38]. All picornaviruses have a similar structure and are small icosahedral viruses with a capsid size of about 27–30 nm. These viral particles consist of 60 copies of a complex of four different viral proteins VP1, VP2, VP3, and VP4 called coxsackievirus B3 coat proteins [39]. The surface of the capsid has a pronounced “canyon” that plays a key role in the binding of the virus to cell receptors and recognition by neutralizing antibodies [40]. Inside this “canyon” is a small endogenous lipid called the “pocket factor” [41]. Inhibitors of enterovirus infection (e.g., pleconaril) displace the “pocket factor” from the “canyon” and stabilize the viral capsid, thereby inhibiting uncoating of the virus and the release of the genome into the infected cell [42].

**Figure 4:**
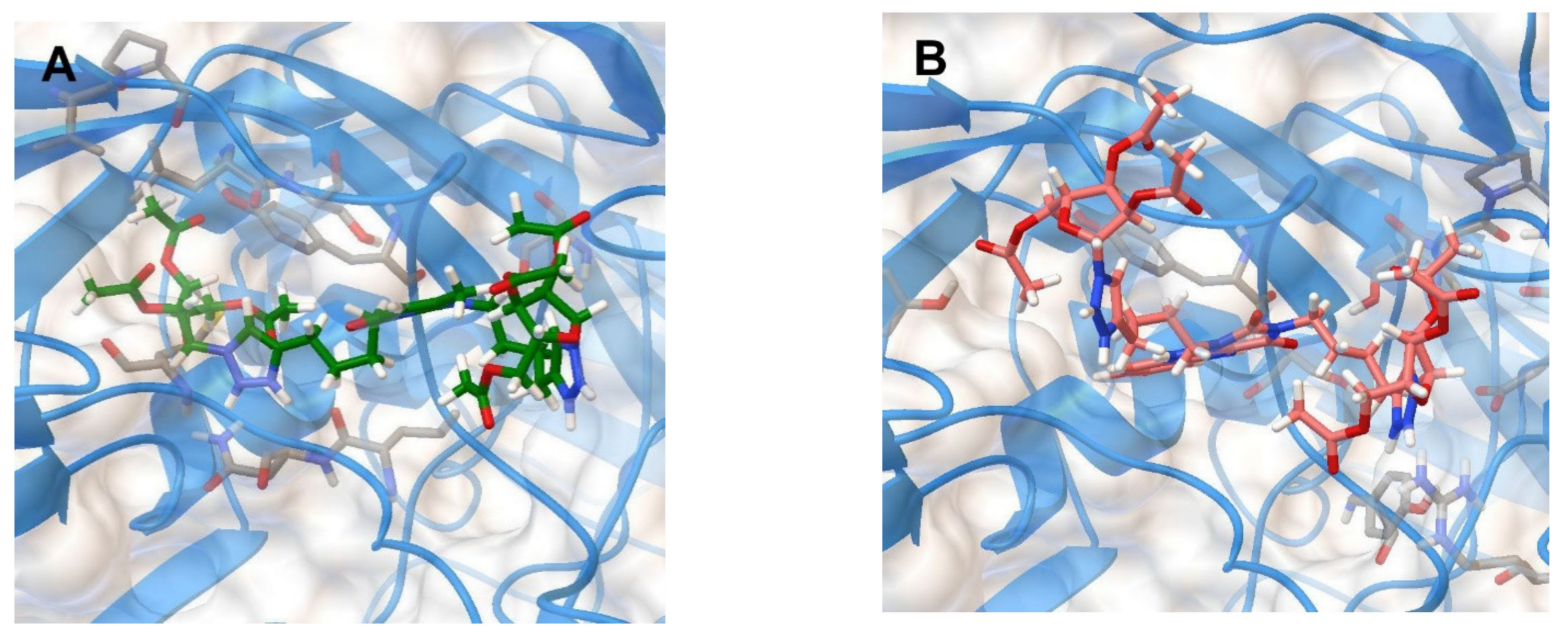
Molecular docking simulations of the optimized docking model of compounds **2f** (**A**) and **5f** (**B**) in the “canyon” of the coxsackievirus B3 coat protein (PDB code 1COV) obtained in the lowest-energy conformations.

Therefore, we decided to test the ability of the lead compounds **2f** and **5f** to bind to the “canyon” of the coxsackievirus B3 capsid in molecular docking simulations. The binding energies of **2f** and **5f** with the coxsackievirus B3 coat protein (PDB code 1COV [30]) have been evaluated (Table 5). The positions of the optimized docking models of compounds **2f** and **5f** exhibiting the best binding energies (−8.3 and −9.2 kkal/mol, respectively) are shown in Figure 4.

According to the docking simulations, compound **2f** is located deep in the “canyon” due to H-bonding with the Ser209 residue of VP2, π-π stacking of 1,2,3-triazole ring with aromatic ring of the Tyr189 residue of VP1, and several hydrophobic interactions. Unlike **2f**, compound **5f** forms more hydrogen bonds: with the amino acid residues Tyr143 and Ser190 of VP1 and with the amino acid residue Arg196 of VP2. Perhaps because of this, **5f** has the better binding energy (−9.2 kkal/mol) than **2f** (−8.3 kkal/mol) that corresponds to the IC_50_ values shown by these nucleoside analogs (11.4 and 12.4 µM, respectively) (Table 5). The results obtained suggest that the antiviral activity of nucleoside analogs **2f** and **5f** against Coxsackievirus B3 is caused by their interaction with the coat proteins VP1 and VP2.

## 3. Conclusions

In summary, a series of novel nucleoside analogues in which two 1,2,3-triazol-4-yl-β-D-ribofuranosyl fragments are attached via polymethylene linkers to the *N*-1 and *N*-3 atoms of the pyrimidine moiety (uracil, 6-methyluracil, thymine, quinazoline-2,4-dione, alloxazine) or to the C-5 and *N*-3 atoms of the 6-methyluracil moiety was synthesized for the first time using the click chemistry methodology. Antiviral assays revealed three compounds, **2i**, **5i**, **11c**, which showed moderate activity against influenza virus A/PR/8/34/(H1N1) with IC_50_ values of 57.5 µM, 24.3 µM, and 29.2 µM, respectively. Two nucleoside analogues **2f** and **5f** in which 1,2,3-triazol-4-yl-2′,3′,5′-tri-*O*-acetyl-β-d-ribofuranosyl fragments are attached to the C-5 and *N*-1 atoms of the heterocycle moiety (6-methyluracil and alloxazine, respectively) exhibited high antiviral potency against the coxsackie B3 virus with IC_50_ values of 12.4 and 11.3 µM, respectively, although both were inactive against the influenza virus A H1N1. It should be noted that this is the second case we have observed when nucleoside analogues with protected hydroxyl groups of ribofuranosyl residue exhibited antiviral activity. The first one was mentioned earlier [22]. According to theoretical calculations, the antiviral activity of the 1,2,3-triazolyl nucleoside analogues **2i**, **5i**, **11c** against the H1N1 (A/PR/8/34) influenza virus can be explained by their influence on the functioning of the polymerase acidic protein of RNA-dependent RNA polymerase. As to the antiviral activity of nucleoside analogs **2f** and **5f** against coxsackievirus B3, it can be explained by their interaction with the coat proteins VP1 and VP2.

## Figures and Tables

**Table 1:** Antiviral activity against A/Puerto Rico/8/34 (H1N1) influenza virus and cytotoxicity of 1,2,3-triazolyl nucleoside analogues synthesized in this study and earlier ones published.

Compound	Structure	CC_50_ ^1^ (μM)	IC_50_ ^2^ (μM)	SI ^3^
**2g**	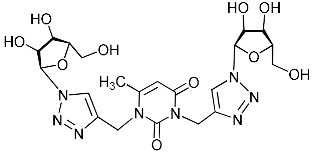	>543.0	>543.0	1
**2j**	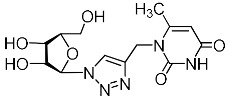	>885	>885	1
**2h**	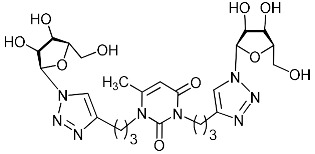	>492.9	>492.9	1
**2k**	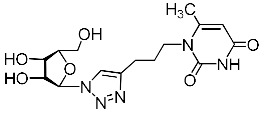	254 ± 19 ^4^	43 ± 5 ^4^	6 ^4^
**2f**	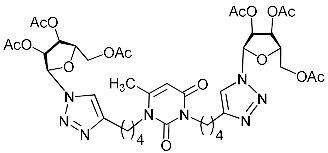	>337.5	96.3 ± 12.4	4
**2i**	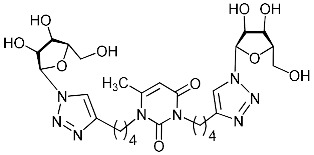	>471.2	57.5 ± 7.1	8
**2m**	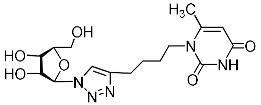	311 ± 27 ^4^	48 ± 6 ^4^	6 ^4^
**4g**	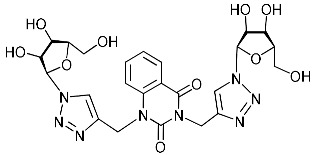	>509.8	180.1 ± 19.9	3
**4j**	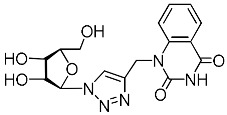	132 ± 9	42 ± 5	3
**4h**	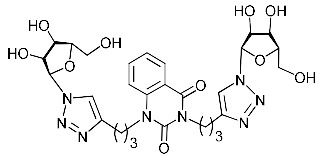	>465.4	186.2 ± 21.6	3
**4k**	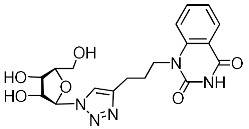	>744 ^4^	212 ± 25 ^4^	4 ^4^
**4f**	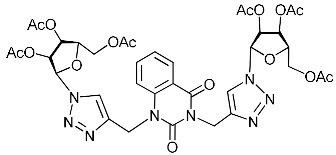	>324.0	103.9 ± 15.1	3
**4i**	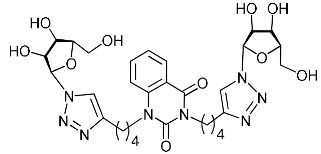	>446.0	297.3 ± 32.4	2
**4m**	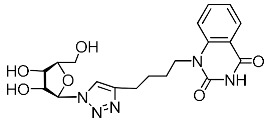	>719	30 ± 4	24
**5g**	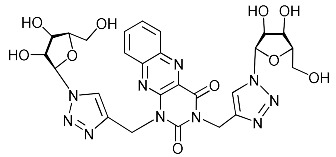	>468.3	187.3 ± 20.8	3
**5h**	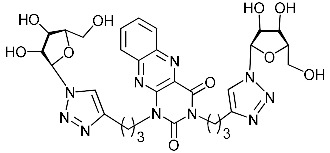	>430.6	114.8 ± 15.2	4
**5f**	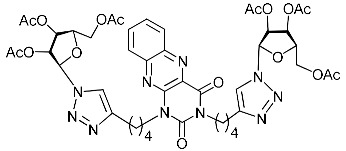	>307.7	91.6 ± 10.8	3
**5i**	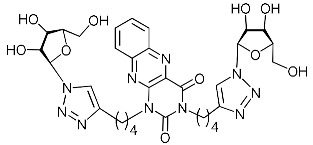	>414.0	24.3 ± 3.2	17
**11c**	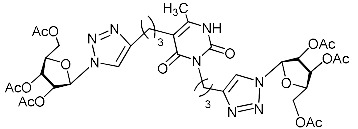	>349.0	29.2 ± 4.3	12
**11g**	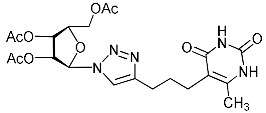	104 ± 8 ^4^	67 ± 8 ^4^	2 ^4^
**11e**	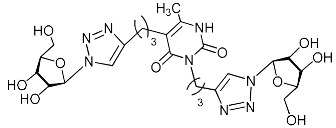	>492.9	164.3 ± 19.2	3
**11h**	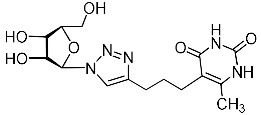	79 ± 6	15 ± 3	5
**11d**	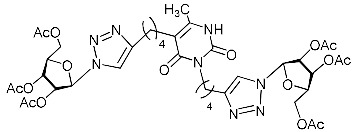	>338.0	119.3 ± 8.7	3
**11i**	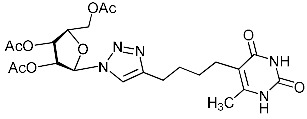	>592 ^4^	>592 ^4^	1 ^4^
**11f**	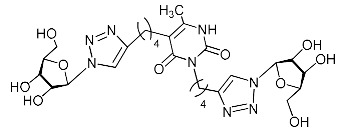	>471.2	>471.2	1
**11j**	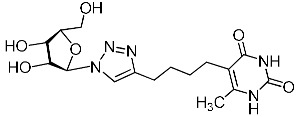	>787	>787	1
	Rimantadine	340 ± 16	77 ± 8	4
	Oseltamivir carboxylate	>200	0.3 ± 0.06	>667

^1^ CC_50_ is the median cytotoxic concentration, i.e., the concentration causing 50% cell death. ^2^ IC50 is the concentration causing 50% inhibition of virus replication. ^3^ SI is the selectivity index, which is the CC_50_/IC_50_ ratio. ^4^ Data from [22].

**Table 2:** Antiviral activity against the Coxsackie B3 virus and cytotoxicity of synthesized 1,2,3-triazolyl nucleoside analogues.

Compound	Structure	CC_50_ ^1^ (μM)	IC_50_ ^2^ (μM)	SI ^3^
**2g**	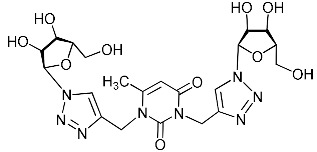	>543.0	>543.0	1
**2j**	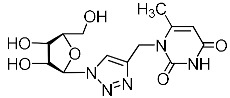	>1180 ^4^	>1180 ^4^	1 ^4^
**2h**	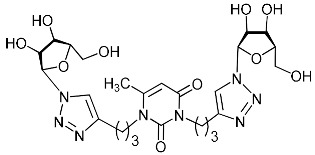	>492.9	>492.9	1
**2k**	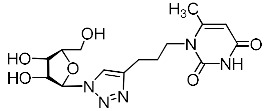	>1090 ^4,3^	>1090 ^4,3^	1 ^4,3^
**2f**	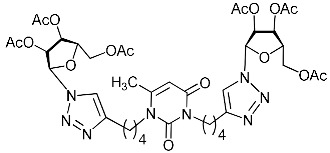	17.7 ± 0.6	>12.4	1
**2i**	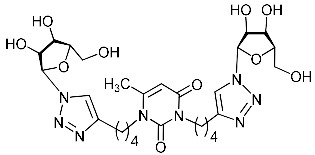	>471.2	>471.2	1
**2m**	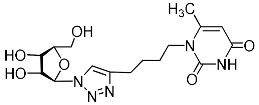	656 ± 33 ^4,3^	106 ± 11 ^4,3^	6 ^4,3^
**4g**	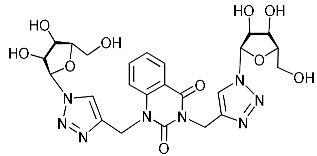	380.6 ± 26.1	>169.9	2
**4j**	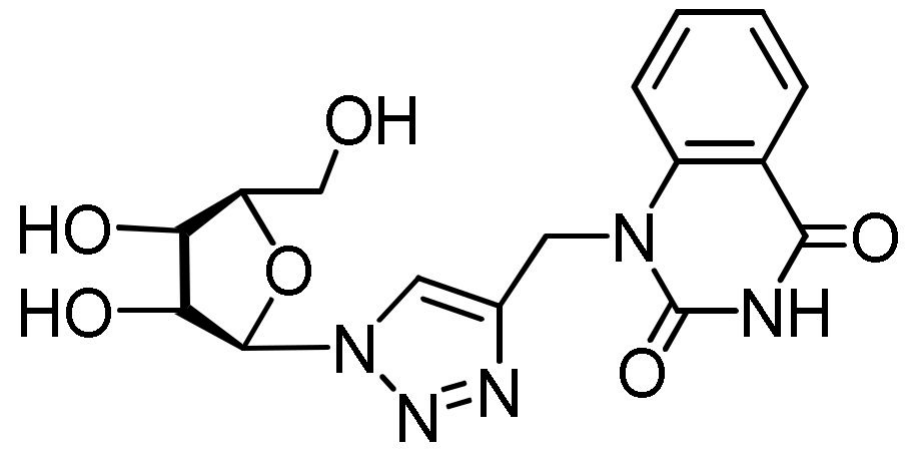	166 ± 12 ^4^	133 ± 15^4^	1 ^4^
**4h**	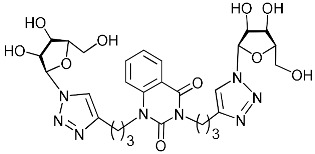	>465.4	>465.4	1
**4k**	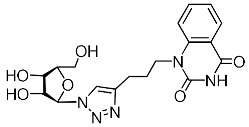	101 ± 8 ^4,3^	>82 ^4,3^	1 ^4,3^
**4i**	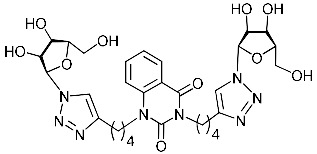	>446.0	>446.0	1
**4m**	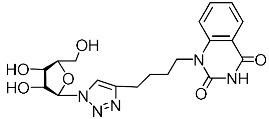	101 ± 9 ^4,3^	>79 ^4,3^	1 ^4,3^
**5g**	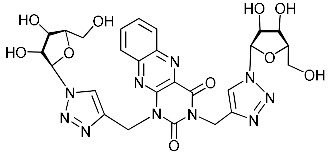	>468.3	>468.3	1
**5h**	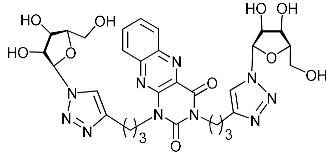	>430.6	>430.6	1
**5f**	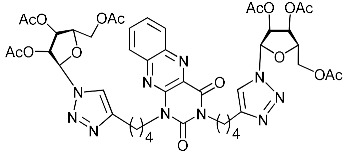	17.8 ± 1.1	>11.3	2
**5i**	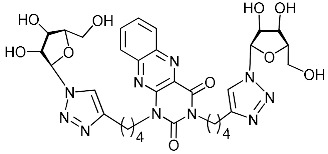	>414.0	>414.0	1
**11e**	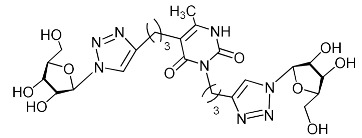	394.3 ± 21.1	>164.3	2
**11g**	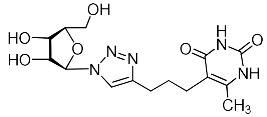	140 ± 10 ^4^	>34 ^4^	4 ^4^
**11f**	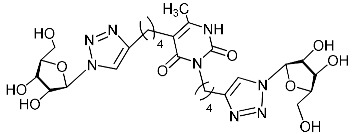	>471.2	>471.2	1
**11h**	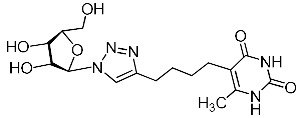	525 ± 31 ^4^	>263 ^4^	2 ^4^
Pleconaril		>1000	21.6	46

^1^ CC_50_ is the median cytotoxic concentration, i.e., the concentration causing 50% cell death. ^2^ IC_50_ is the concentration causing 50% inhibition of virus replication. ^3^ SI is the selectivity index, which is the CC_50_/IC_50_ ratio. ^4^ Data from [22].

**Table 3:** Cytotoxic activity of several 1,2,3-triazolyl nucleoside analogues against human cancer and normal human cell lines (IC_50_ values in µM with standard errors) ^1^.

Compound	Structure	IC_50_ (µM)			
		Cancer Cell Lines			Normal Cell Line
		M-HeLa ^2^	HuTu-80 ^3^	PC-3 ^4^	WI-38 ^5^
**2f**	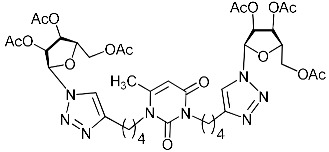	88.1 ± 7.4	>100	>100	>100
**2i**	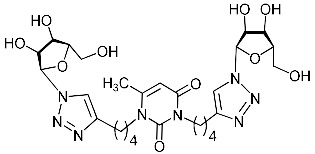	100 ± 8.5	>100	>100	>100
**5f**	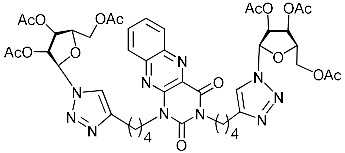	71.4 ± 6.3	>100	66.8 ± 5.3	>100
**5i**	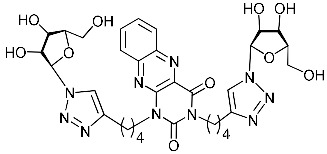	71 ± 6.1	>100	>100	>100
	5-Fluorouracil	62.0 ± 4.9	65.2 ± 5.5	10.3 ± 0.8	82.5 ± 6.6

**^1^** Viability of the cells studied was evaluated from fluorescence intensity data [23]. ^2^ M-HeLa is a human cervix epitheloid carcinoma. ^3^ HuTu-80 is a duodenal adenocarcinoma. ^4^ PC-3 is a human Caucasian prostate adenocarcinoma. ^5^ WI-38 is a diploid human cell strain composed of fibroblasts derived from lung tissue of a 3-month gestation aborted female fetus.

**Table 4:** Inhibitory activity against influenza virus A H1N1 of the lead compounds as well as their binding energies and ligand–protein interactions obtained by molecular docking simulations.

Compound	Structure	IC_50_ (µM)	−E_bind_ (kkal/mol)	Ligand–Protein Interactions
**2i**	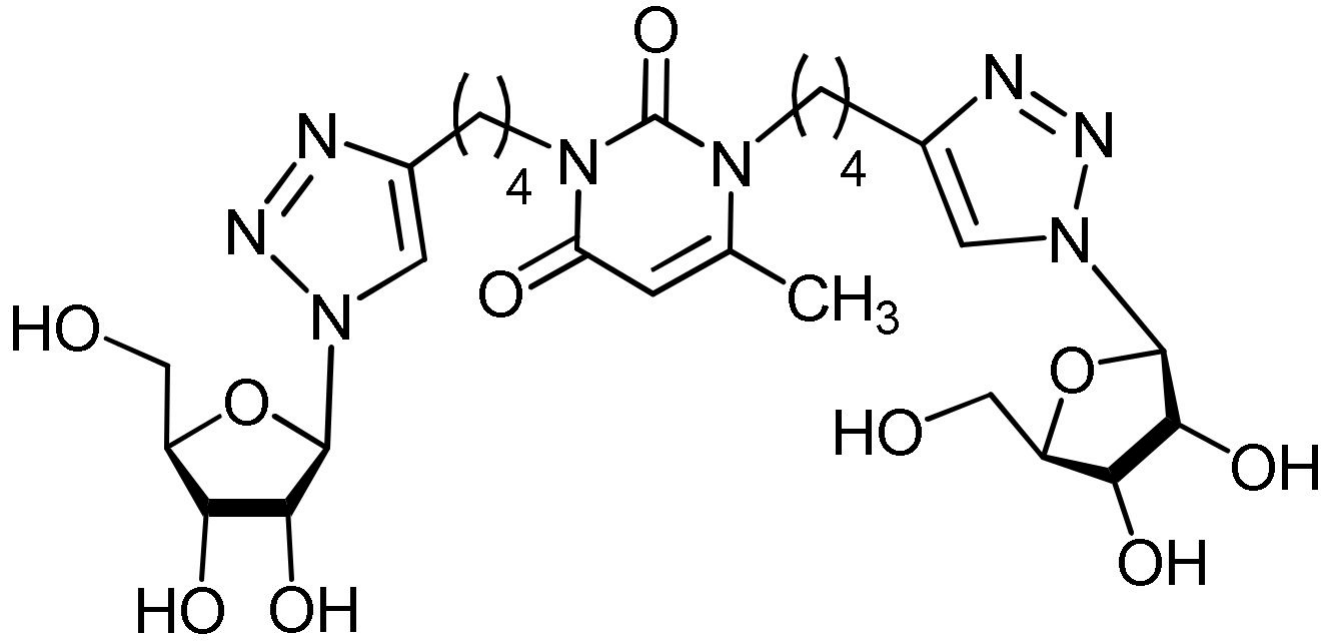	57.5	8.7	H-bonding: Val122, Lys134, Lys137
**2i-TP**	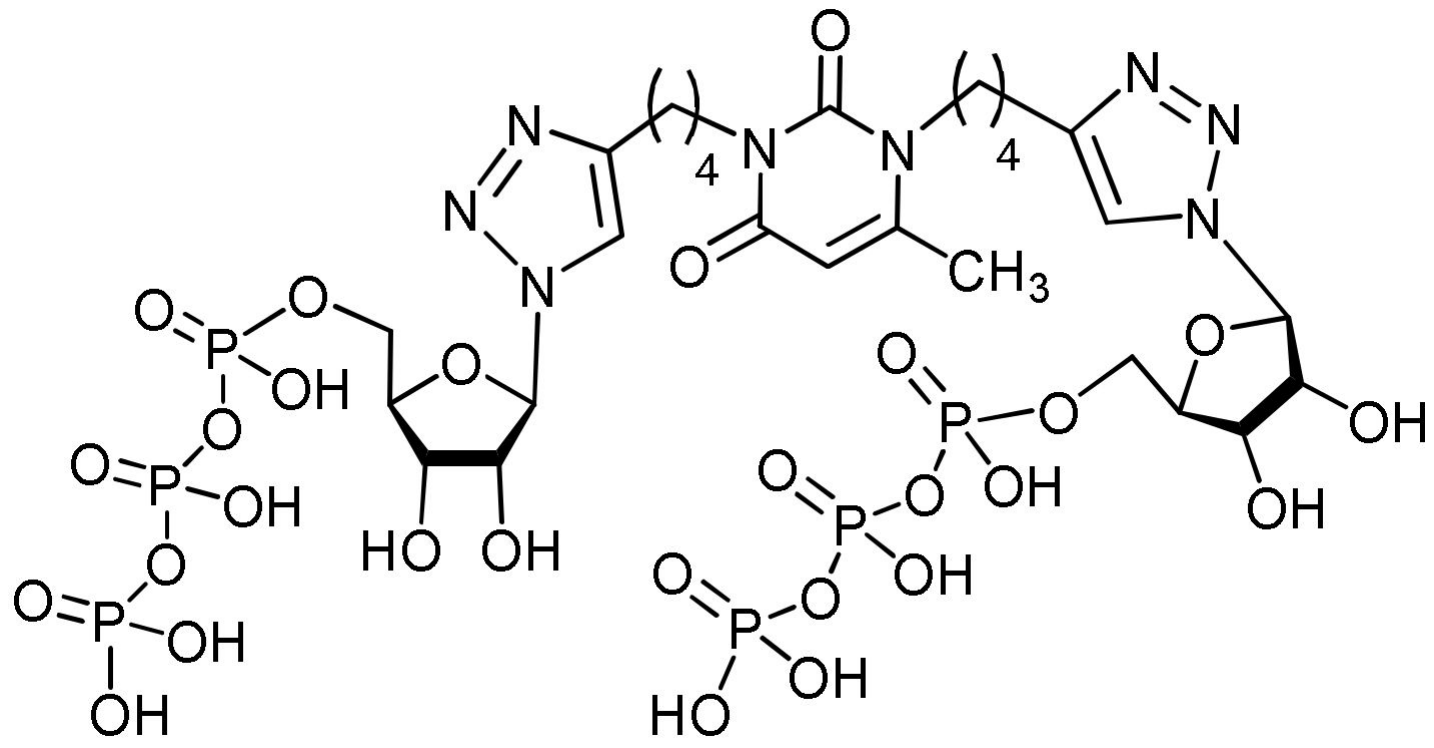	−	8.8	H-bonding: Tyr24, Val122, Lys134, Lys137, Arg196 π-cation: Lys34
**5i**	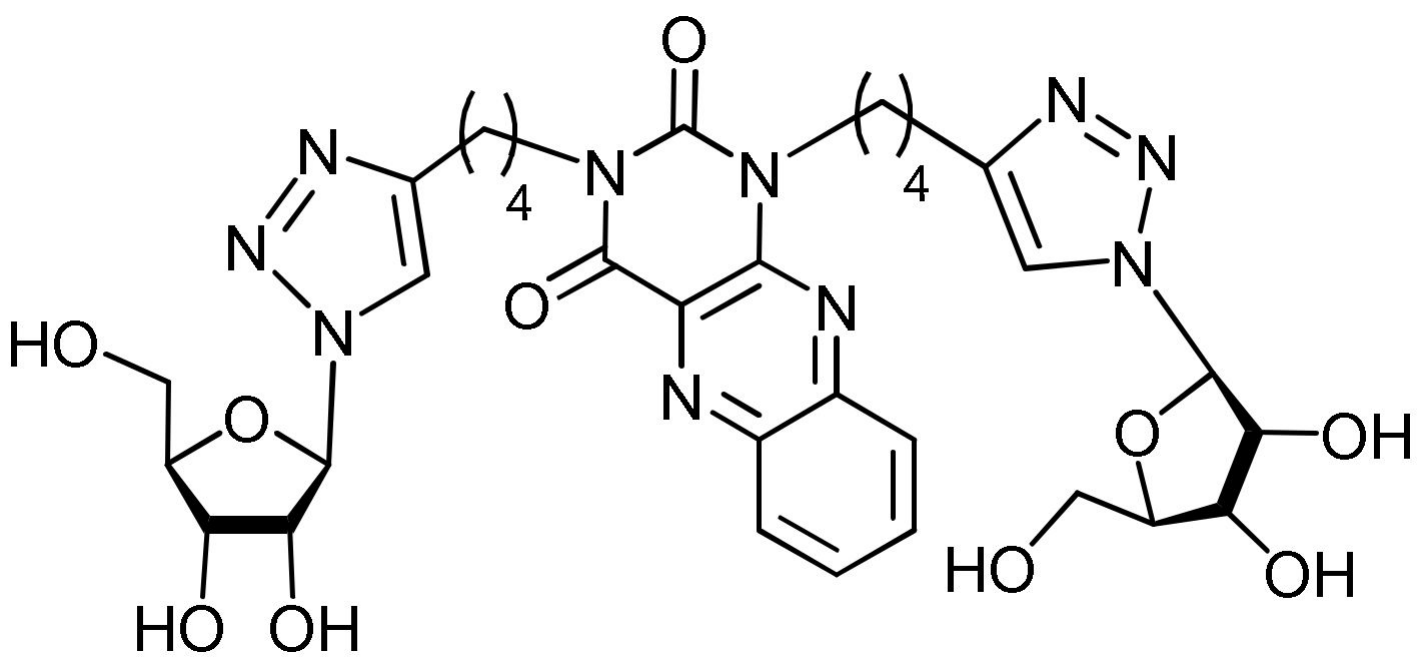	24.3	9.3	H-bonding: Tyr24, Gly81, Leu106 π-cation: Mn^2+^, Lys134
**5i-TP**	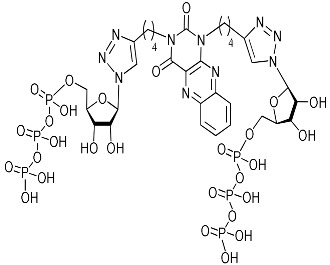	−	9.1	H-bonding: Tyr24, Gly81, Arg84, Lys137 π-π: Tyr24
**11c**	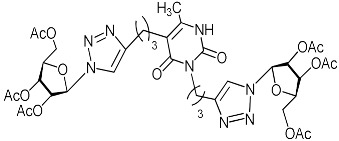	29.2	8.1	H-bonding: Arg84, Leu106, Lys134, Lys137, Arg196 π-cation: Mn^2+^.

**Table 5:** Inhibitory activity against Coxsackievirus B3 of the lead compounds as well as their binding energies and ligand-protein interactions obtained via molecular docking simulations.

Compound	Structure	IC_50_ (µM)	−E_bind_ (kkal/mol)	Ligand–Protein Interactions
**2f**	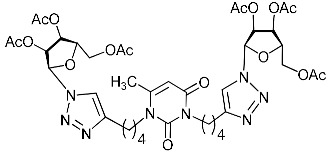	>12.4	8.3	H-bonding: Ser209 (VP2) π-π: Tyr189 (VP1)
**5f**	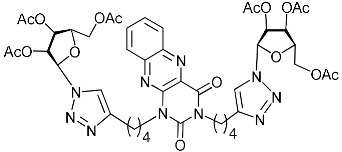	>11.3	9.2	H-bonding: Tyr143 (VP1), Ser190 (VP1), Arg196 (VP2)

## Data Availability

Raw data are stored at the authors.

## Supplementary Materials

The following are available online: general information (S2), procedures for the synthesis of the compounds (S2–S3, S8, S9–S10), characterization of the compounds synthesized (S3–S28) including their NMR spectra (Figures S1–S96), the molecular docking study (S78), and description of the antiviral and cytotoxicity assays (S77). References [21,23,28,29,30,31,32,33] are cited in the supplementary.

## Sample Availability

Samples of the compounds are kept amongst the authors.
