# Supplementary material for: Synthesis and Antiviral Evaluation of Nucleoside Analogues Bearing One Pyrimidine Moiety and Two D-Ribofuranosyl Residues
Source: Molecules. 2021 Jun 16;26(12):3678. doi: 10.3390/molecules26123678 (PMC8234143; doi:10.3390/molecules26123678)
Supplement: Supplementary file 1 [file molecules-26-03678-s001.zip › molecules-1240741-supplementary.pdf]

molecules

MDPI

Supplementary Material

Synthesis and Antiviral Evaluation of Nucleoside Analogues Bearing One Pyrimidine Moiety and Two D-Ribofuranosyl Residues

Olga V. Andreeva [1], Bulat F. Garifullin [1], Vladimir V. Zarubaev [2], Alexander V. Slita [2], Iana L. Yesaulkova [2], Alexandrina S. Volobueva [2], Mayya G. Belenok [1], Maria A. Man'kova [1], Liliya F. Saifina [1], Marina M. Shulaeva [1], Alexandra D. Voloshina [1], Anna P. Lyubina [1], Vyacheslav E. Semenov [1,*] and Vladimir E. Kataev [1,*]

[1] Arbuzov Institute of Organic and Physical Chemistry, Federal Research Center "Kazan Scientific Center of Russian Academy of Sciences", Arbuzov 8, Kazan 420088, Russia; andreeva@iopc.ru (O.V.A.); garifullin.bulat@iopc.ru (B.F.G.); maya@iopc.ru (M.G.B.); mankovamaria98@gmail.com (M.A.M.); lsayfina@iopc.ru (L.F.S.); mshulaeva@iopc.ru (M.M.S.); microbi@iopc.ru (A.D.V.); aplyubina@gmail.com (A.P.L.)

[2] Pasteur Institute of Epidemiology and Microbiology, Mira 14, Saint Petersburg 197101, Russia; zarubaev@gmail.com (V.V.Z.), a_slita@yahoo.com (A.V.S.); Yesaulkova@gmail.com (I.L.Y.); sasha-khrupina@mail.ru (A.S.V.)

* Correspondence: sve@iopc.ru (V.E.S.); kataev@iopc.ru (V.E.K.); Tel.: +7-843-279-47-09 (V.E.S.)

Content:

1. General information …… S2
2. General procedure for the synthesis of 1,3-bis(alkynyl)-2,4(1*H*,3*H*)-pyrimidine-, quinazoline-, and benzo[g]pteridinediones …… S2
2.1 Characterization of compounds …… S3
3. Preparation of 6-methyl-3,5-bis(alkynyl)-2,4(1*H*,3*H*)-pyrimidinediones …… S8
3.1 Characterization of compounds …… S9
4. General procedure for the synthesis of the protected 1,2,3-triazolyl nucleoside analogues …. S9
5. General procedure for the synthesis of the 1,2,3-triazolyl nucleoside analogues with free hydroxyl groups …… S10
6. Characterization of the 1,2,3-triazolyl nucleoside analogues …… S10
7. NMR spectra Figures S1–S96…… S29
8. Antiviral assay …… S77
9. Cytotoxicity assay …… S77
10. Molecular docking study …… S78
11. References …… S78

1. General information

Melting points were obtained on ElectrothermalIA 9000 instrument (Electrothermal, Great Britain). ^{1}H NMR spectra were recorded on 400 MHz and 600 MHz Brucker Advance. ^{13}C NMR spectra were obtained in the above instrument operating at 100.6 MHz. Mass spectra (MALDI) were recorded in a positive ion mode on a Bruker Ultraflex III TOF/TOF mass spectrometer for 10^{-3} mg/mL solutions in MeOH.The ESI MS measurements were performed using an AmazonX ion trap mass spectrometer (Bruker Daltonic GmbH, Germany) in positive mode in the mass range of 70–3000. The capillary voltage was 3500 V, nitrogen drying gas 10 $L{\cdot}min^{-1}$, desolvation temperature 250 °C. A methanol/water solution (70:30) was used as a mobile phase at a flow rate of 0.2 mL/min by binary pump (Agilent 1260 chromatograph, USA). The sample was dissolved in methanol to a concentration of 10^{-6} $g{\cdot}L^{-1}$.Flash chromatography was performed on silica gel 60 (40-63 μm, Buchi, Sepacore). Thin-layer chromatography was carried out on plates with silica gel (Sorbfil, Russia). Spots of compounds were visualized by using ultraviolent fluorescence under a short wavelength (254 nm) followed by heating the plates (at ca. 150 °C) after immersion in a solution of 5% H_2SO_4 and 95% H_2O. All reactions sensitive to air and/or moisture were carried out under argon atmosphere with anhydrous solvents. Anhydrous solvents were purified and dried (where appropriate) according to standard procedures.

Uracil **1**, 6-methyluracil **2**, thymine **3**, quinazoline-2,4-dione **4**, alloxazine **5** and propargyl bromide were purchased from Sigma-Aldrich; D-ribose was purchased from Acros. 5-Iodo-pent-1-yn and 6-iodo-hex-1-yn were prepared from purchased 5-chloro-1-pentyne and 6-chloro-1-hexyne (Sigma-Aldrich) by their reaction with sodium iodide in acetone. 1,3-Bis(prop-2-yn-1-yl)-6-methyluracil **2b**, 2,3,5-tri-*O*-acetyl-β-D-ribofuranosyl azide **15**, nucleoside analogues **2e** and **2h** were prepared as described earlier [1,2]. Spectral data of **2b**, **15**, **2e**, **2h** were in keeping with published ones [1,2].

2. General procedure for the synthesis of 1,3-bis(alkynyl)-2,4(1*H*,3*H*)-pyrimidine-, quinazoline-, and benzo[g]pteridinediones

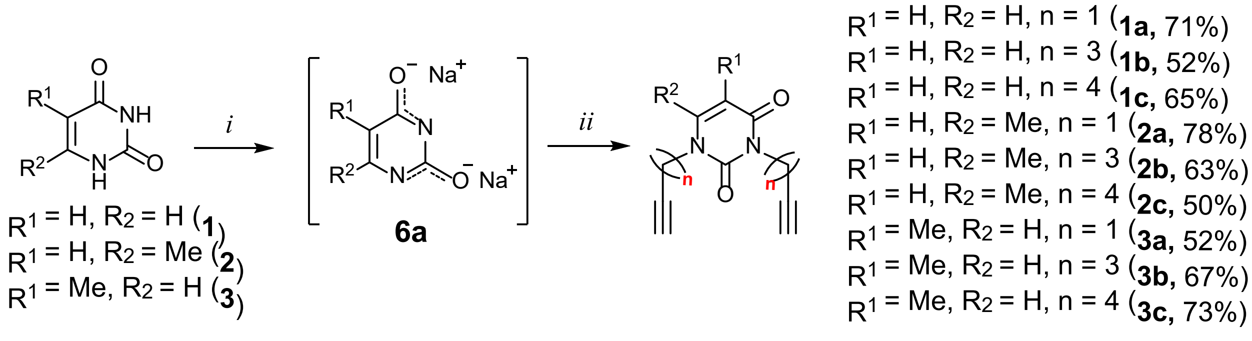

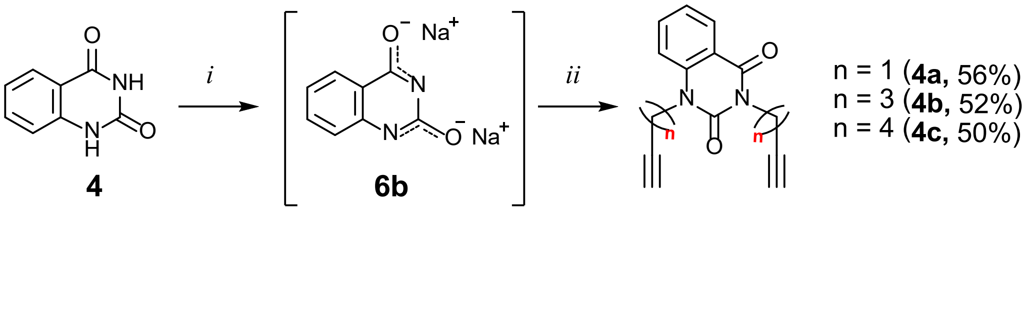

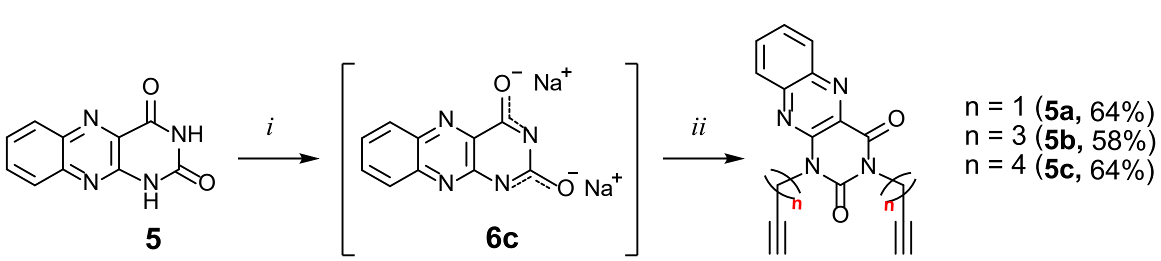

Synthesis of 1,3-bis(α,ω-alkinyl)-2,4(1*H*,3*H*)-pyrimidinediones. Reagents and conditions: (*i*) NaH, DMF, 65–70 °C, 8 h; (*ii, iii*) HC≡C-$(CH_2)_n$-Hlg, DMF, 115–120 °C, 16 h

Sodium hydride (30 mmol) preliminarily treated with hexane was added to a solution of 15 mmol of uracil 1, 6-methyluracil 2, thymine 3, quinazoline-2,4-dione 4, and alloxazine 5 in DMF (150 mL). The reaction mixture was stirred for 8 h at 65–70 °C, cooled to room temperature and 60 mmol of the corresponding alkyne (propargyl bromide, 5-iodo-1-pentyne or 6-iodo-1-hexyne) was added to the sodium salt thus obtained. The mixture was stirred at 115–120 °C until the pH achieved 7.0–7.3 (12–16 h). The solvent was distilled off, 100 mL of chloroform was added to the residue and filtered. The filtrate was concentrated to 10–15 mL and purified by column chromatography on silica gel. The column was eluted first with petroleum ether and then by the mixture of petroleum ether-ethyl acetate (1.5:1). The target compounds **1a–c**, **2a–c**, **3a–c, 4a–c**, **5a–c** were isolated from the second fraction.

2.1. Characterization of compounds

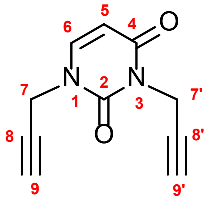

1,3-Bis(prop-2-yn-1-yl)-2,4(1H,3H)-pyrimidinedione (**1a**).

A white powder (2.00 g, 71%). M.p. 108 °C. ^{1}H-NMR (400 MHz, $CDCl_3$): δ7.46 (d, 1H, *J* = 8.0 Hz, H-6), 5.82 (d, 1H, *J* = 8.0 Hz, H-5), 4.68 (d, 2H, *J* = 2.4 Hz, 2H-7′), 4.58 (d, 2H, *J* = 2.8 Hz, 2H-7),2.50 (t, 1H, *J* = 2.8 Hz, H-9′), 2.16 (t, 1H, *J* = 2.4 Hz, H-9). ^{13}C-NMR (100 MHz, $CDCl_3$): δ 161.50 (C=O, C-4), 150.31 (C=O, C-2), 140.67 (C-6), 102.19 (C-5), 75.81 (C-8, C-8′), 70.74 (C-9,C-9″), 37.81 (C-7), 30.35 (C-7′).ESI-MS: calcd. for $C_{10}H_8N_2O_2$ [M + H]$^+$ 189.1; found [M + H]$^+$189.2. Anal.,%: C 63.78; H 4.55, N 14.92. $C_{10}H_8N_2O_2$. Calcd., %: C 63.83; H 4.29; N 14.89. *M* 188.06.

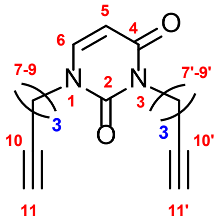

1,3-Bis(pent-4-yn-1-yl)-2,4(1H,3H)-pyrimidinedione (**1b**).

Oil (1.90 g, 52%). ^{1}H-NMR (400 MHz, $CDCl_3$): δ 7.15 (d, 1H, *J* = 8.0 Hz, H-6), 5.67 (d, 1H, *J* = 7.4 Hz, H-5), 3.99 (t, 2H, *J* = 7.2 Hz, 2H-7′), 3.84 (t, 2H, *J* = 6.8 Hz, 2H-7), 2.26-2.20 (m, 4H, 2H-9′, 2H-9), 2.01 (t, 1H, *J* = 2.4 Hz, H-11′), 1.92 (t, 1H, *J* = 2.8 Hz, H-11), 1.91-1.80 (m, 4H, 2H-8′, 2H-8). ^{13}C-NMR (100 MHz, $CDCl_3$): δ 162.95 (C=O, C-4), 151.38 (C=O, C-2), 142.46 (C-6), 101.50 (C-5), 83.32 (C-10), 82.30 (C-10′), 70.00 (C-11), 68.56 (C-11′), 48.67 (C-7), 40.39 (C-7′), 27.07 (C-9), 26.34 (C-9′), 16.29 (C-8), 15.47 (C-8′). ESI-MS: calcd. for

$C_{14}H_{16}N_2O_2$[M + H]+ 245.1; found [M + H]+ 245.2. Anal.,%: C 68.88; H 6.55, N 11.52. $C_{14}H_{16}N_2O_2$. Calcd., %: C 68.83; H 6.60; N 11.47. *M*244.12.

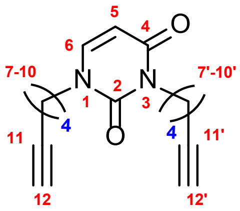

1,3-Bis(hex-5-yn-1-yl)-2,4(1H,3H)-pyrimidinedione (**1c**).

Oil (2.65 g, 65%). ^{1}H-NMR (600 MHz, $CDCl_3$): δ 7.08 (d, 1H, *J* = 7.8 Hz, H-6), 5.64 (d, 1H, *J* = 7.8 Hz, H-5), 3.88 (t, 2H, *J* = 7.2 Hz, 2H-7′), 3.70 (t, 2H, *J* = 7.2 Hz, 2H-7), 2.20-2.15 (m, 4H, 2H-10′, 2H-10), 1.92 (t, 1H, *J* = 2.4 Hz, H-12′), 1.88 (t, 1H, *J* = 2.4 Hz, H-12), 1.79-1.74 (m, 2H, 2H-8′), 1.70-1.65 (m, 2H, 2H-8), 1.52-1.48 (m, 4H, 2H-9′, 2H-9). ^{13}C-NMR (100 MHz, $CDCl_3$): δ162.79 (C=O, C-4), 151.23 (C=O, C-2), 141.91 (C-6), 101.67 (C-5), 83.88 (C-11), 83.36 (C-11′), 69.03 (C-12), 68.44 (C-12′), 48.45(C-7), 40.45(C-7′), 27.88 (C-10), 26.59 (C-10′), 25.67 (C-8), 24.99 (C-8′),17.83 (C-9, C-9′). ESI-MS: calcd. for $C_{16}H_{20}N_2O_2$[M + H]+ 273.2; found [M + H]+273.2. Anal.,%: C 70.58; H 7.45, N 10.22. $C_{16}H_{20}N_2O_2$. Calcd., %: C 70.56; H 7.40; N 10.29. *M*272.15.

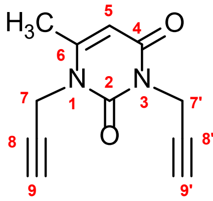

6-Methyl-1,3-bis(prop-2-yn-1-yl)-2,4(1H,3H)-pyrimidinedione (**2a**).

A white powder (3.13 g, 78%). M.p. 118 °C. ^{1}H-NMR (600 MHz, DMSO-d_6): δ 5.74 (s, 1H, H-5), 4.69 (d, 2H, *J* = 2.4 Hz, 2H-7′), 4.52 (d, 2H, *J* = 2.4 Hz, 2H-7), 3.36 (t, 1H, *J* = 2.4 Hz, H-9′), 3.05 (t, 1H, *J* = 2.4 Hz, H-9), 2.36 (s, 3H, CH_3-6).^{13}C-NMR (100 MHz, DMSO-d_6): δ 160.04 (C=O, C-4), 152.60 (C=O, C-2), 150.40 (C-6), 100.45 (C-5), 78.89 (C-8), 78.32 (C-8′), 75.11 (C-9), 72.74 (C-9′), 33.81 (C-8), 29.89 (C-8′), 18.61 (CH_3). ESI-MS: calcd. for $C_{11}H_{10}N_2O_2$[M + H]+ 203.1; found [M + H]+ 203.2. Anal.,%: C 65.38; H 5.03, N 13.81. $C_{11}H_{10}N_2O_2$. Calcd., %: C 65.34; H 4.98; N 13.85. *M* 202.07.

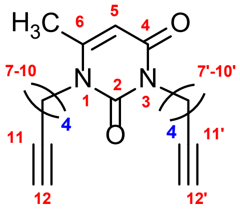

6-Methyl-1,3-bis(hex-5-yn-1-yl)-2,4(1H,3H)-pyrimidinedione (**2c**).

A white powder (2.15 g, 50%). M.p. 66 °C. ^{1}H-NMR (600 MHz, $CDCl_3$): δ 5.56 (s, 1H, H-5), 3.92 (t, 2H, *J* = 7.2 Hz, 2H-7′), 3.82 (t, 2H, *J* = 7.2 Hz, 2H-7),2.26-2.20 (m, 4H, 2H-10′, 2H-10), 2.23 (s, 3H, CH_3-6), 1.95 (t, 1H, *J* = 2.4 Hz, H-12′), 1.91 (t, 1H, *J* = 2.4 Hz, H-12),1.79-1.70(m, 4H, 2H-9′, 2H-9), 1.60-1.53 (m, 4H, 2H-8′, 2H-8). ^{13}C-NMR (100 MHz, $CDCl_3$): δ 162.11 (C=O, C-4), 151.99 (C=O, C-2), 150.91 (C-6), 101.74 (C-5), 84.07 (C-11), 83.43 (C-11′), 69.08 (C-12), 68.47 (C-12′), 44.55 (C-7), 40.66 (C-7′), 27.83 (C-10), 26.77(C-10′), 25.82 (C-8), 25.40 (C-8′), 19.63 (C-9), 18.13, (C-9′), 17.96 (CH_3). ESI-MS: calcd. for $C_{17}H_{22}N_2O_2$[M + H]+ 287.2; found [M + H]+ 287.1. Anal.,%: C 71.79; H 7.28, N 9.83. $C_{17}H_{22}N_2O_2$. Calcd., %: C 71.74; H 7.34; N 9.78. *M*286.17.

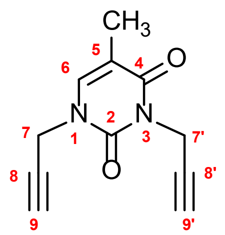

5-Methyl-1,3-bis(prop-2-yn-1-yl)-2,4(1H,3H)-pyrimidinedione (**3a**).

Oil (1.58 g, 52%). ^{1}H-NMR (600 MHz, $CDCl_3$): δ 7.29 (s, 1H, H-6), 4.75 (d, 2H, *J* = 1.8 Hz, 2H-7′), 4.60 (d, 2H, *J* = 2.4 Hz, 2H-7),2.49 (t, 1H, *J* = 2.4 Hz, H-9′), 2.18 (t, 1H, *J* = 2.4 Hz, H-9), 2.02 (s, 3H, CH_3-5). ^{13}C-NMR (100 MHz, $CDCl_3$): δ 162.46 (C=O, C-4), 150.35 (C=O, C-2), 136.66 (C-6), 110.79 (C-5), 78.07 (C-8), 76.33 (C-8′), 75.27 (C-9), 70.63 (C-9′), 37.50 (C-7), 30.63 (C-7′), 13.00 (CH_3). ESI-MS: calcd. for $C_{11}H_{10}N_2O_2$[M + H]$^+$ 203.1; found [*M*+H]$^+$ 203.2. Anal.,%: C 65.40; H 4.92, N 13.91. $C_{11}H_{10}N_2O_2$. Calcd., %: C 65.34; H 4.98; N 13.85. *M*202.07.

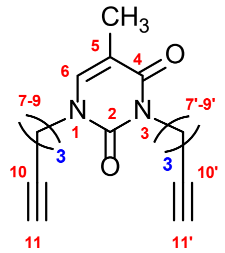

5-Methyl-1,3-bis(pent-4-yn-1-yl)-2,4(1H,3H)-pyrimidinedione (**3b**).

Oil (2.60 g, 67%). ^{1}H-NMR (400 MHz, $CDCl_3$): δ 7.00 (s, 1H, H-6),4.03 (t, 2H, *J* = 7.4 Hz, 2H-7′), 3.83 (t, 2H, *J* = 6.8 Hz, 2H-7), 2.29-2.21 (m, 4H, 2H-9′, 2H-9), 2.02 (t, 1H, *J* = 2.4 Hz, H-11′), 1.93 (t, 1H, *J* = 2.8 Hz, H-11), 1.91 (s, 3H, CH_3-5), 1.90-1.81 (m, 4H, 2H-8′, 2H-8). ^{13}C-NMR (100 MHz, $CDCl_3$): δ 163.61 (C=O, C-4), 151.26 (C=O, C-2), 138.53 (C-6), 109.59 (C-5), 83.29 (C-10), 82.39 (C-10′), 69.76 (C-11), 68.47 (C-11′), 48.31(C-7), 40.50(C-7′), 27.14 (C-9), 26.34 (C-9′), 16.25 (C-8), 15.47 (C-8′), 12.86 (CH_3). ESI-MS: calcd. for $C_{15}H_{18}N_2O_2$[M + H]$^+$ 245.1; found [M + H]$^+$ 259.1. Anal.,%: C 69.78; H 7.05, N 10.82. $C_{15}H_{18}N_2O_2$. Calcd., %: C 69.74; H 7.02; N 10.84. *M*258.14.

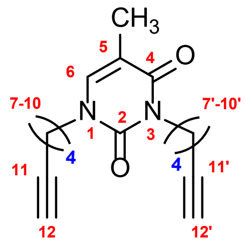

5-Methyl-1,3-bis(hex-5-yn-1-yl)-2,4(1H,3H)-pyrimidinedione (**3c**).

A white powder (3.13 g, 73%). M.p. 67 °C. ^{1}H-NMR (600 MHz, $CDCl_3$): δ6.94 (s, 1H, H-6), 3.92 (t, 2H, *J* = 7.2 Hz, 2H-7′), 3.69 (t, 2H, *J* = 7.2 Hz, 2H-7), 2.22-2.17 (m, 4H, 2H-10′, 2H-10), 1.94 (t, 1H, *J* = 2.4 Hz, H-12′), 1.89 (t, 1H, *J* = 2.4 Hz, H-12), 1.87 (s, 3H, CH_3-5), 1.79-1.74 (m, 2H, 2H-8′), 1.72-1.67(m, 2H, 2H-8), 1.55-1.49 (m, 4H, 2H-9′, 2H-9). ^{13}C-NMR (100 MHz, $CDCl_3$): δ 163.56 (C=O, C-4), 151.26 (C=O, C-2), 138.08 (C-6), 109.69 (C-5), 83.99 (C-11), 83.36 (C-11′), 68.98 (C-12), 68.41 (C-12′), 48.71 (C-7), 40.72 (C-7′), 27.99 (C-10), 26.72 (C-10′), 25.78 (C-8), 25.09 (C-8′), 18.05 (C-9), 17.91, (C-9′), 12.90 (CH_3). ESI-MS: calcd. for $C_{17}H_{22}N_2O_2$[M + H]$^+$ 287.2; found [M + H]$^+$ 287.1. Anal.,%: C 71.68; H 7.35, N 9.72. $C_{17}H_{22}N_2O_2$. Calcd., %: C 71.74; H 7.34; N 9.78. *M* 286.17.

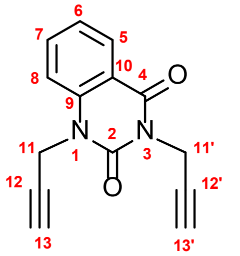

1,3-Bis(prop-2-yn-1-yl)-2,4(1H,3H)-quinazolinedione (**4a**).

A white powder (2.00 g, 56%). M.p. 168 °C. ^{1}H-NMR (400 MHz, $CDCl_3$): δ 8.26 (d, 1H, *J* = 8.0 Hz, H-5), 7.73 (t, 1H, *J* = 8.0 Hz, H-6), 7.39 (d, 1H, *J* = 8.4 Hz, H-8), 7.31 (t, 1H, *J* = 7.6 Hz, H-7), 4.96 (d, 2H, *J* = 2.4 Hz, 2H-11′), 4.86 (d, 2H, *J* = 2.4 Hz, 2H-11), 2.32 (t, 1H, *J* = 2.0 Hz, H-13′), 2.20 (t, 1H, *J* = 2.4 Hz, H-13). ^{13}C-NMR (100 MHz, $CDCl_3$): δ 160.68 (C=O, C-4), 149.70 (C=O, C-2), 138.93 (C-9), 135.39 (C-10), 129.26 (C-8), 123.59 (C-5), 115.63 (C-7), 114.16 (C-6), 78.09 (C-12, C-12′), 73.47 (C-13), 70.88 (C-13′), 33.39 (C-11), 31.12 (C-11′). ESI-MS: calcd. for $C_{14}H_{10}N_2O_2$[*M*+H]$^+$ 239.1; found [M + H]$^+$ 239.0. Anal.,%: C 70.55; H 4.18, N 11.82. $C_{14}H_{10}N_2O_2$. Calcd., %: C 70.58; H 4.23; N 11.76. *M*238.07.

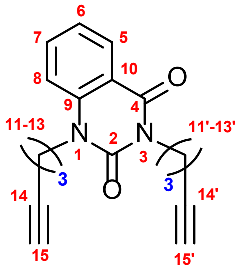

1,3-Bis(pent-2-yn-1-yl)-2,4(1H,3H)-quinazolinedione (**4b**).

A white powder, 52%. M.p. 85 °C. ^{1}H-NMR ($CDCl_3$, 400 MHz): δ 8.19 (d, 1H, *J* = 8.0 Hz, H-5), 7.64 (t, 1H, *J* = 8.0 Hz, H-6), 7.28-7.20 (m, 2H, H-7, H-8), 4.25-4.21 (m, 2H, H-11′), 4.17-4.14 (m, 2H, H-11), 2.35-2.33 (m, 2H, H-13′), 2.29-2.26 (m, 2H, H-13), 2.04 (t, 1H, *J* = 2.0 Hz, H-15′), 1.96-1.90 (m, 5H, H-15, H-12′, H-12). ^{13}C-NMR ($CDCl_3$, 100 MHz): δ 161.62 (C=O, C-4), 150.73 (C=O, C-2), 139.66 (C-9), 135.01 (C-10), 129.11 (C-8), 122.78 (C-5), 115.66 (C-7), 113.30 (C-6), 83.32 (C-14), 82.84 (C-14′), 69.40 (C-15), 68.60 (C-15′), 42.68 (C-11), 41.01 (C-11′), 26.56 (C-13), 25.69 (C-13′), 16.33 (C-12), 16.01 (C-12′). ESI-MS *m/z*: calcd for $C_{18}H_{18}N_2O_2$ 294.1; found 295.1 [M + H]$^+$. Elemental Analysis calcd for $C_{18}H_{18}N_2O_2$, C 73.45, H 6.16, N 9.52%; found C 73.40, H 6.18, N 9.57%.

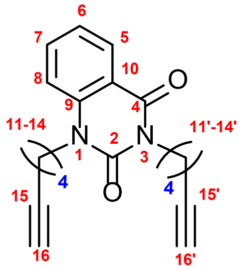

1,3-Bis(hex-2-yn-1-yl)-2,4(1H,3H)-quinazolinedione (**4c**).

A white powder, 50%. M.p. 73 °C. ^{1}H-NMR ($CDCl_3$, 600 MHz): δ 8.17 (d, 1H, *J* = 7.8 Hz, H-5), 7.62 (t, 1H, *J* = 7.8 Hz, H-6), 7.20-7.17 (m, 2H, H-7, H-8), 4.11 (t, 2H, *J* = 7.8 Hz, H-11′), 4.06 (t, 2H, 1H, *J* = 7.8 Hz, H-11), 2.27-2.24 (m, 2H, H-14′), 2.22-2.19 (m, 2H, H-14), 1.95 (t, 1H, *J* = 2.4 Hz, H-16′), 1.90 (t, 1H, *J* = 2.4 Hz, H-16), 1.85-1.81 (m, 2H, H-12′), 1.80-1.75 (m, 2H, H-12), 1.65-1.60 (m, 2H, H-13′), 1.59-1.54 (m, 2H, H-13). ^{13}C-NMR ($CDCl_3$, 100 MHz): δ 161.48 (C=O, C-4), 150.61 (C=O, C-2), 139.58 (C-9), 134.82 (C-10), 129.02 (C-8), 122.61 (C-5), 115.62 (C-7), 113.37 (C-6), 83.93 (C-15), 83.50 (C-15′), 68.96 (C-16), 68.47 (C-16′), 42.99 (C-11), 41.15 (C-11′), 26.93 (C-14), 26.18 (C-14′), 25.78 (C-12), 25.33 (C-12′), 18.07

(C-13), 17.94 (C-13′). ESI-MS *m/z*: calcd for $C_{20}H_{22}N_2O_2$ 322.2; found 323.1 $[M + H]^+$. Elemental Analysis calcd for $C_{20}H_{22}N_2O_2$, C 74.51, H 6.88, N 8.69%; found C 74.47, H 6.93, N 8.63%.

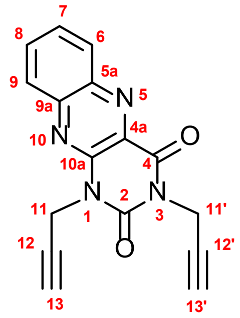

1,3-Bis(prop-2-yn-1-yl)-2,4(1H,3H)-benzo[g]pteridinedione (**5a**).

An yellow powder, 64%. M.p. 163 °C. ^{1}H-NMR (DMSO-*d*$_6$, 600 MHz): δ 8.25 (d, 1H, *J* = 7.8 Hz, H-5), 8.07 (d, 1H, *J* = 8.4 Hz, H-9), 8.01 (t, 1H, *J* = 7.8 Hz, H-7), 7.86 (t, 1H, *J* = 7.8 Hz, H-8), 5.07 (d, 2H, *J* = 1.8 Hz, H-11′), 4.76 (d, 2H, *J* = 2.4 Hz, H-11), 3.21-3.19 (m, 2H, H-13′, H-13). ^{13}C-NMR (100 MHz, DMSO-*d*$_6$): δ 158.15 (C=O, C-4), 148.98 (C=O, C-2), 144.27, 141.84 (C-4a, C-10a), 139.04, 133.92 (C-5a, C-9a), 130.89, 129.99, 129.28, 127.46 (C-6, C-7, C-8, C-9), 78.71 (C-12), 78.49 (C-12′), 73.90 (C-13), 73.70 (C-13′), 31.82 (C-11), 31.25 (C-11′). ESI-MS: calcd. for $C_{16}H_{10}N_4O_2$ $[M + H]^+$ 290.1; found $[M + H]^+$ 291.1. Anal.,%: C 66.25; H 3.53, N 19.22. $C_{16}H_{10}N_4O_2$. Calcd., %: C 66.20; H 3.47; N 19.30.

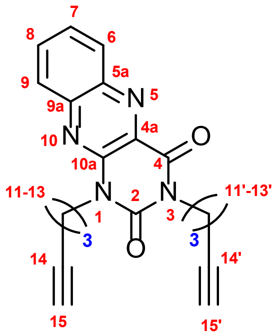

1,3-Bis(pent-2-yn-1-yl)-2,4(1H,3H)-benzo[g]pteridinedione (**5b**).

An yellow powder, 58%. M.p. 148 °C. ^{1}H-NMR (DMSO-*d*$_6$, 400 MHz): δ 8.22 (d, 1H, *J* = 7.8 Hz, H-5), 8.03-7.95 (m, 2H, H-9, H-7), 7.84-7.80 (m, 1H, H-8), 4.40 (t, 2H, *J* = 6.8 Hz, H-11′), 4.08 (t, 2H, *J* = 7.2 Hz, H-11), 2.77-2.74 (m, 2H, H-15′, H-15), 2.33-2.26 (m, 4H, H-13′, H-13), 1.98-1.91 (m, 2H, H-12′), 1.88-1.81 (m, 2H, H-12). ^{13}C-NMR (100 MHz, DMSO-*d*$_6$): δ 159.10 (C=O, C-4), 150.13 (C=O, C-2), 145.32, 141.92 (C-4a, C-10a), 138.67, 133.32 (C-5a, C-9a), 131.26, 129.79, 128.67, 127.32 (C-6, C-7, C-8, C-9), 83.86 (C-14), 83.76 (C-14′), 71.12 (C-15), 71.03 (C-15′), 41.20 (C-11), 40.90 (C-11′), 26.02 (C-13), 25.93 (C-13′), 15.50 (C-12), 15.43 (C-12′). ESI-MS: calcd. for $C_{20}H_{18}N_4O_2$ $[M + H]^+$ 346.1; found $[M + H]^+$ 347.1. Anal.,%: C 69.40; H 5.29, N 16.22. $C_{20}H_{18}N_4O_2$. Calcd., %: C 69.35; H 5.24; N 16.17.

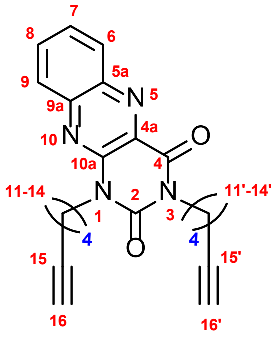

1,3-Bis(hex-2-yn-1-yl)-2,4(1H,3H)-benzo[g]pteridinedione (**5c**).

An yellow powder, 64%. M.p. 114–115 °C. ^{1}H-NMR (DMSO-d_6, 400 MHz): δ 8.21 (d, 1H, *J* = 8.4 Hz, H-5), 8.02-7.94 (m, 2H, H-9, H-7), 7.83-7.80 (m, 1H, H-8), 4.31 (t, 2H, *J* = 6.8 Hz, H-11′), 4.01 (t, 2H, *J* = 6.8 Hz, H-11), 2.74-2.71 (m, 2H, H-16′, H-16), 2.27-2.20 (m, 4H, H-14′, H-14), 1.87-1.80 (m, 2H, H-12′), 1.79-1.71 (m, 2H, H-12), 1.61-1.50 (m, 4H, H-13′, H-13). ^{13}C-NMR (100 MHz, DMSO-d_6): δ 159.02 (C=O, C-4), 150.04 (C=O, C-2), 145.24, 141.95 (C-4a, C-10a), 138.67, 133.29 (C-5a, C-9a), 131.19, 129.78, 128.63, 127.33 (C-6, C-7, C-8, C-9), 84.21 (C-15), 84.16 (C-15′), 71.15 (C-16), 71.10 (C-16′), 41.37 (C-11), 41.07 (C-11′), 26.38 (C-14), 26.10 (C-14′), 25.29 (C-12), 25.18 (C-12′), 17.39 (C-13), 17.38 (C-13′). ESI-MS: calcd. for $C_{22}H_{22}N_4O_2$ [*M*+H]$^+$ 374.2; found [M + H]$^+$ 375.2. Anal.,%: C 70.51; H 5.89, N 15.00. $C_{22}H_{22}N_4O_2$. Calcd., %: C 70.57; H 5.92; N 14.96.

3. Preparation of 6-methyl-3,5-bis(alkynyl)-2,4(1*H*,3*H*)-pyrimidinediones

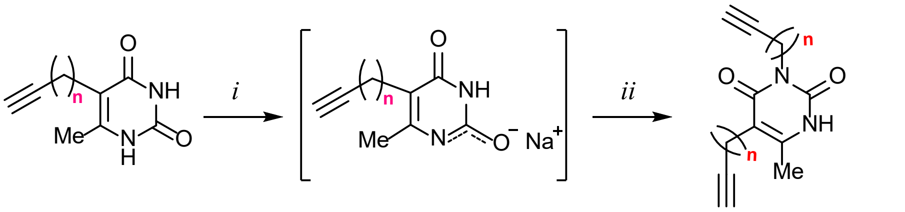

n = 3 (**9a**)
n = 4 (**9b**)

n = 3 (**10a**)
n = 4 (**10b**)

n = 3 (**11a,** 22%)
n = 4 (**11b,** 29%)

Synthesis of 6-methyl-3,5-bis(α,ω-alkynyl)-2,4(1*H*,3*H*)-pyrimidinediones. Reagents and conditions: (i) NaH, DMF, 65–70 °C, 8 h; (*ii*, *iii*) HC≡C-$(CH_2)_n$-I, DMF, 115–120 °C, 16 h

6-Methyl-5-(pent-4-yn-1-yl)-2,4(1*H*,3*H*)-pyrimidinediones **9a** and **9b** as well as their monosodium salts **10a** and **10b** were prepared as described earlier [3]. Spectral data of **9a**, **9b** were in keeping with published ones [3].

Sodium hydride (10mmol) preliminarily treated with hexane was added to a solution of 10 mmol of 6-methyl-5-(pent-4-yn-1-yl)-2,4(1*H*,3*H*)-pyrimidinedione **9a** or 6-methyl-6-(hex-5-yn-1-yl)- 2,4(1*H*,3*H*)-pyrimidinedione **9b** in DMF (100 mL). The reaction mixture was stirred for 8 h at 65–70 °C, cooled to room temperature and 20 mmol of the 5-iodo-1-pentyne or 6-iodo-1-hexyne was added to the sodium salt **10** thus obtained. The mixture was stirred at 115–120 °C until the pH achieved 7.0–7.3 (12–16 h). The solvent was distilled off, 100 mL of chloroform was added to the residue and filtered. The filtrate was concentrated to 10–15 mL and purified by column chromatography on silica gel. The column was eluted first with petroleum ether, mixture of petroleum ether-ethyl acetate 3:1 and then by the mixture of petroleum ether-ethyl acetate 1.5:1. The target 6-methyl-1,5-bis(alkynyl)-2,4(1*H*,3*H*)-pyrimidinediones **11a**, **11b** were isolated from the third fractions.

3.1. Characterization of compounds

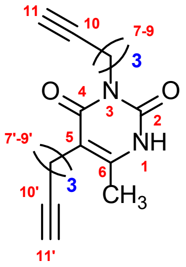

6-Methyl-1,5-bis(pent-4-yn-1-yl)-2,4(1H,3H)-pyrimidinedione (**11a**).

A white powder, 22%. M.p. 118 °C. ^{1}H-NMR ($CDCl_3$, 600 MHz): δ10.54 (s, 1H, H-1), 4.02 (t, 2H, *J* = 7.2 Hz, H-7), 2.48 (t, 2H, *J* = 7.2 Hz, H-7′),2.26-2.18 (m, 7H, H-9′, H-9, CH_3), 1.96 (t, 1H, *J* = 2.4 Hz,H-11′), 1.93 (t, 1H, *J* = 2.4 Hz,H-11), 1.90-1.85 (m, 2H, H-8′), 1.71-1.68 (m, 2H, H-8).^{13}C-NMR ($CDCl_3$,100 MHz): δ163.48 (C=O, C-4), 152.90 (C=O, C-2), 145.46 (C-6), 110.21 (C-5), 84.17 (C-10′), 83.41 (C-10), 68.81 (C-11′), 68.42 (C-11),39.89(C-7), 29.65(C-7′), 29.31 (C-9′), 27.41(C-9), 26.63 (C-8′), 24.35 (C-8), 18.09 (C-9′, C-9), 16.37 (CH_3).).ESI-MS *m*/*z*: calcd for $C_{15}H_{18}N_2O_2$258.1; found 259.0 [M + H]$^+$. Elemental Analysis calcd for $C_{15}H_{18}N_2O_2$, C 69.74, H 7.02, N 10.84%; found C 69.76, H 7.07, N 10.77%.

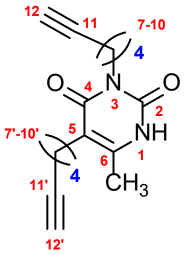

6-Methyl-1,5-bis(hex-5-yn-1-yl)-2,4(1H,3H)-pyrimidinedione (**11b**).

A white powder, 29%. M.p. 91 °C. ^{1}H-NMR ($CDCl_3$, 400 MHz): δ10.42 (s, 1H, H-1), 3.94 (t, 2H, *J* = 7.8 Hz, H-7), 2.37 (t, 2H, *J* = 7.2 Hz, H-7′),2.25-2.20 (m, 4H, H-10′, H-10), 2.16 (s, 3H, CH_3), 1.93-1.91 (m, 2H, H-12′, H-12), 1.79-1.71 (m, 2H, H-8′), 1.60-1.55 (m, 6H, H-9′, H-8, H-9).^{13}C-NMR ($CDCl_3$,100 MHz): δ163.51 (C=O, C-4), 152.82 (C=O, C-2), 144.93 (C-6), 110.86 (C-5), 84.27 (C-11′), 84.10 (C-11), 68.46(C-12′), 68.37 (C-12), 40.07(C-7), 28.12(C-7′), 27.89 (C-10′), 26.88(C-10), 25.84 (C-8′), 24.75(C-8), 18.1718.12(C-9′, C-9), 16.42 (CH_3).ESI-MS*m*/*z*: calcd for $C_{17}H_{22}N_2O_2$286.2; found 287.1 [M + H]$^+$. Elemental Analysis calcd for $C_{17}H_{22}N_2O_2$,C 71.30, H 7.34, N 9.78%; found C 71.26, H 7.37, N 9.73%.

4. General procedure for the synthesis of the protected 1,2,3-triazolyl nucleoside analogues

To a solution of *N*-1,*N*-3-bisalkynylated pyrimidines **1a–c**, **2a–c**, **3a–c**, **4a–c**, **5a–c**, **11a, 11b** (1 mmol) and the protected azido β-D-ribofuranose (2 mmol) **15** in a mixture of 1:1 tert-butanol/water was added freshly prepared solution of sodium ascorbate (2 mmol) in 5 mL water and $CuSO_4 \times 5H_2O$ (2 mmol) in 5 mL water. The reaction mixture was stirred at 40 °C for 48 h, then was concentrated under reduced pressure. The residue was taken up in methylene chloride, washed successively with water, dried over anhydrous sodium sulphate and concentrated under vacuum to provide desired compounds **1d–f**, **2d–f**, **3d–f**, **4d–f**, **5d–f, 11c, 11d**.

5. General procedure for the synthesis of the 1,2,3-triazolyl nucleoside analogues with free hydroxyl groups

The protected 1,2,3-triazolyl nucleoside analogues **1d–f; 2d–f; 3d–f; 4d–f; 5d–f; 11c,11d** were dissolved in anhydrous MeOH at room temperature and the pH was adjusted to 9.0 using a solution of 0.1 N MeONa/MeOH. The deacetylation procedure was monitored by TLC and upon its completion the pH adjusted to 7.0 with acidic ion-exchange resin Amberlyst 15. After filtration, the filtrate was concentrated under reduced pressure to afford the corresponding target compounds **1g–i; 2g–i; 3g–i; 4g–i; 5g–i;, 11e, 11f**.

6. Characterization of the 1,2,3-triazolyl nucleoside analogues

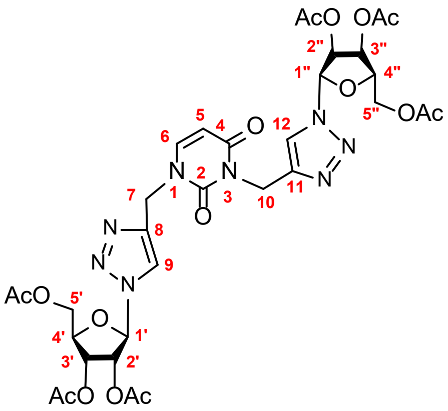

1,3-Bis{[1-(2,3,5-tri-O-acetyl-β-D-ribofuranosyl)-1H-1,2,3-triazol-4-yl]methyl}pyrimidine-2,4(1H,3H)-dione (**1d**)

A white foam, 98% yield. ^{1}H-NMR ($CDCl_3$, 400 MHz) δ 7.91, 7.78 (2s, 2H, H-9, H-12), 7.45 (d, 1H, J = 7.7 Hz, H-6), 6.11, 6.08 (2d, 2H, J = 3.6 Hz, H-1′, H-1″), 5.80-5.74 (m, 2H, H-3′, H-3″), 5.73 (d, 1H, J = 8.1 Hz, H-5), 5.57 (t, 2H, J = 5.3 Hz, H-2′, H-2″), 5.26-5.14 (m, 2H, H-10), 4.98 (q, 2H, J = 14.9 Hz, H-7), 4.48-4.40 (m, 2H, H-4′, H-4″), 4.40-4.33 (m, 2H, H-5a′, H-5a″), 4.23-4.14 (m, 2H, H-5b′, H-5b″), 2.09, 2.08, 2.08, 2.07, 2.06, 2.05 (6s, 18H, 6OAc). ^{13}C-NMR ($CDCl_3$, 100 MHz) δ 170.38, 169.33, 169.12 (6C=O, $OCOCH_3$), 162.34 (C=O, C-4), 151.07 (C=O, C-2), 143.29, 142.22 (2C, C-8, C-11), 142.42 (CH, C-6), 123.28, 122.83 (2CH, C-9, C-12), 101.98 (CH, C-5), 90.11, 89.93 (2CH, C-1′, C-1″), 80.95, 80.80 (2CH, C-4′, C-4″), 74.28, 74.24 (2CH, C-3′, C-3″), 70.70, 70.67 (2CH, C-2′, C-2″), 62.79 ($2CH_2$, C-5′, C-5″), 43.97, 35.91 ($2CH_2$, C-7, C-10), 20.57, 20.37, 20.29 ($6CH_3$, $OCOCH_3$). MALDI MS *m/z*: calcd for $C_{32}H_{39}N_8O_{16}$ 791.2, $C_{32}H_{38}N_8NaO_{16}$ 813.2; found 791.3 [M + H]$^+$, 813.3 [M + Na]$^+$. Elemental Analysis calcd for $C_{32}H_{38}N_8O_{16}$, C 48.61, H 4.84, N 14.17%; found C 48.58, H 4.89, N 14.19%.

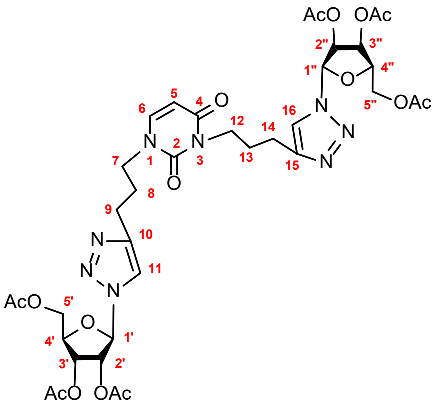

1,3-Bis{3-[1-(2,3,5-tri-O-acetyl-β-D-ribofuranosyl)-1H-1,2,3-triazol-4-yl]propyl}pyrimidine-2,4(1H,3H)-dione (**1e**)

A white foam, 66% yield. ^{1}H-NMR ($CDCl_3$, 400 MHz) δ 7.67, 7.59 (2s, 2H, H-11, H-16), 7.26 (d, 1H, *J* = 7.7 Hz, H-6), 6.11 (d, 2H, *J* = 4.0 Hz, H-1′, H-1″), 5.8 (t, 2H, *J* = 4.4 Hz, H-3′, H-3″), 5.67 (d, 1H, *J* = 8.0 Hz, H-5), 5.63-5.57 (m, 2H, H-2′, H-2″), 4.48-4.42 (m, 2H, H-4′, H-4″), 4.38 (dd, 2H, *J* = 12.3, 3.1 Hz, H-5a′, H-5a″), 4.26-4.18 (m, 2H, H-5b′, H-5b″), 4.01 (t, 2H, *J* = 7.0 Hz, H-12), 3.82 (t, 2H, *J* = 7.0 Hz, H-7), 2.83-2.74 (m, 4H, H-9, H-14), 2.16-2.02 (m, 22 H, 6OAc, H-8, H-13). ^{13}C-NMR ($CDCl_3$, 100 MHz) δ 170.40, 170.29, 169.40, 169.26, 169.21 (6C=O, $OCOCH_3$), 163.07 (C=O, C-4), 151.49 (C=O, C-2), 147.83, 146.85 (2C, C-10, C-15), 142.72 (CH, C-6), 120.49, 120.28 (2CH, C-11, C-16), 101.44 (CH, C-5), 89.90, 89.80 (2CH, C-1′, C-1″), 80.82, 80.71 (2CH, C-4′, C-4″), 74.26, 74.24 (2CH, C-3′, C-3″), 70.86, 70.81 (2CH, C-2′, C-2″), 63.04, 63.01 (2CH_2, C-5′, C-5″), 48.81, 40.48 (2CH_2, C-7, C-12), 28.12, 26.57 (2CH_2, C-9, C-14), 23.05, 22.06 (2CH_2, C-8, C-13), 20.66, 20.43, 20.36 (6CH_3, $OCOCH_3$). MALDI MS *m*/*z*: calcd for $C_{36}H_{47}N_8O_{16}$ 847.3; found 847.3 $[M + H]^+$. Elemental Analysis calcd for $C_{36}H_{46}N_8O_{16}$, C 51.06, H 5.48, N 13.23%; found C 51.11, H 5.54, N 13.18%.

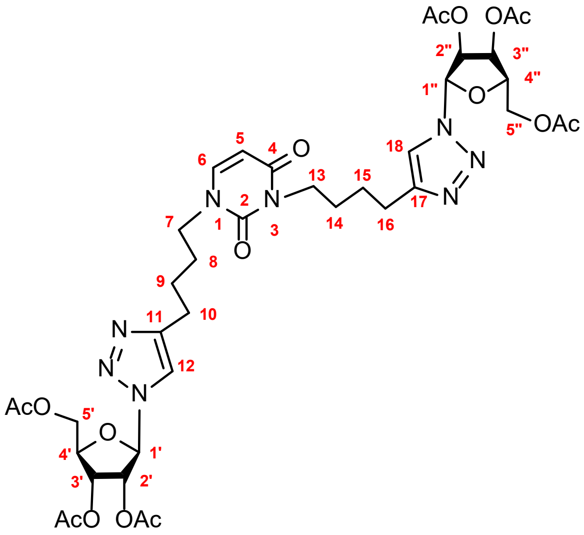

1,3-Bis{4-[1-(2,3,5-tri-O-acetyl-β-D-ribofuranosyl)-1H-1,2,3-triazol-4-yl]butyl}pyrimidine-2,4(1H,3H)-dione (**1f**)

A white foam, 87% yield. ^{1}H-NMR ($CDCl_3$, 400 MHz) δ 7.52, 7.48 (2s, 2H, H-12, H-18), 7.10 (d, 1H, *J* = 8.1 Hz, H-6), 6.10 (t, 2H, *J* = 3.7 Hz, H-1′, H-1″), 5.82-5.77 (m, 2H, H-3′, H-3″), 5.68 (d, 1H, *J* = 8.1 Hz, H-5), 5.60 (q, 2H, *J* = 5.4 Hz, H-2′, H-2″), 4.47-4.41 (m, 2H, H-4′, H-4″), 4.41-4.35 (m, 2H, H-5a′, H-5a″), 4.21 (dd, 2H, *J* = 12.3, 4.6 Hz, H-5b′, H-5b″), 3.95 (t, 2H, *J* = 6.6 Hz, H-13), 3.74 (t, 2H, *J* = 3.7 Hz, H-7), 2.76 (t, 4H, *J* = 6.4 Hz, H-10, H-16), 2.13-2.03 (m, 18H, 6OAc), 1.80-1.65 (m, 8H, H-8, H-9, H-14, H-15). ^{13}C-NMR ($CDCl_3$, 100 MHz) δ 170.35, 170.27, 169.38, 169.24, 169.19 (6C=O, $OCOCH_3$), 162.98 (C=O, C-4), 151.40 (C=O, C-2), 148.20, 147.59 (2C, C-11, C-17), 142.12 (CH, C-6), 120.16, 120.10 (2CH, C-12, C-18), 101.64 (CH, C-5), 89.88, 89.78 (2CH, C-1′, C-1″), 80.76 (2CH, C-4′, C-4″), 74.25 (2CH, C-3′, C-3″), 70.83, 70.78 (2CH, C-2′, C-2″), 62.95 (2CH_2, C-5′, C-5″), 49.36, 40.67 (2CH_2, C-7, C-13), 28.35, 26.89, 26.47, 26.05, 25.05, 24.85 (6CH_2, C-8, C-9, C-10, C-14, C-15, C-16), 20.64, 20.41, 20.35 (6CH_3, $OCOCH_3$). MALDI MS *m*/*z*: calcd for $C_{38}H_{51}N_8O_{16}$ 875.8; found 875.4 $[M + H]^+$. Elemental Analysis calcd for $C_{38}H_{50}N_8O_{16}$, C 52.17, H 5.76, N 12.81%; found C 52.13, H 5.74, N 12.88%.

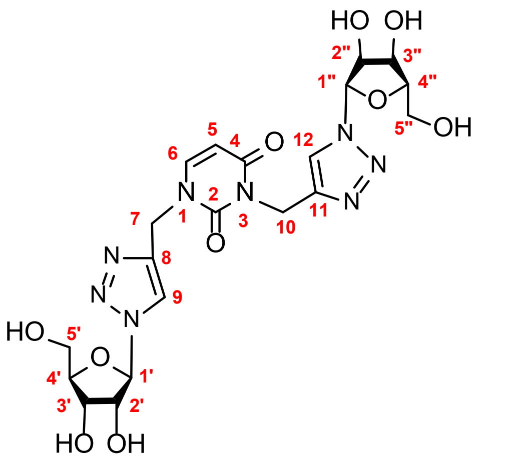

1,3-Bis{[1-(β-D-ribofuranosyl)-1H-1,2,3-triazol-4-yl]methyl}pyrimidine-2,4(1H,3H)-dione (**1g**)

A white foam, 76% yield. ^{1}H-NMR (CD_3OD, 400 MHz) δ 8.27, 8.14 (2s, 2H, H-9, H-12), 7.70 (d, 1H, *J* = 7.7 Hz, H-6), 6.02, 5.99 (2d, 2H, *J* = 3.7 Hz, H-1′, H-1″), 5.76 (d, 1H, *J* = 8.1 Hz, H-5), 5.18 (s, 2H, H-10), 5.05 (s, 2H, H-7), 4.50-4.44 (m, 2H, H-3′, H-3″), 4.37-4.32 (m, 2H, H-2′, H-2″), 4.13-4.08 (m, 2H, H-4′, H-4″), 3.83-3.76 (m, 2H, H-5a′, H-5a″), 3.70-3.64 (m, 2H, H-5b′, H-5b″). ^{13}C-NMR (CD_3OD, 100 MHz) δ 163.34 (C=O, C-4), 151.27 (C=O, C-2), 143.86 (CH, C-6), 143.20, 142.44 (2C, C-8, C-11), 122.78, 122.54 (2CH, C-9, C-12), 100.77 (CH, C-5), 93.05, 92.96 (2CH, C-1′, C-1″), 85.85, 85.78 (2CH, C-4′, C-4″), 75.64, 75.58 (2CH, C-3′, C-3″), 70.50, 70.48 (2CH, C-2′, C-2″), 61.49, 61.40 ($2CH_2$, C-5′, C-5″), 43.73, 35.62 ($2CH_2$, C-7, C-10). MALDI MS *m*/*z*: calcd for $C_{20}H_{27}N_8O_{10}$ 539.4; found 539.2 $[M + H]^+$. Elemental Analysis calcd for $C_{20}H_{26}N_8O_{10}$, C 44.61, H 4.87, N 20.81%; found C 44.69, H 4.91, N 20.85%.

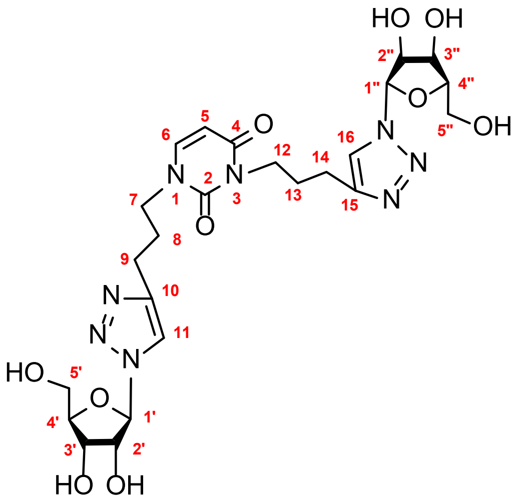

1,3-Bis{3-[1-(β-D-ribofuranosyl)-1H-1,2,3-triazol-4-yl]propyl}pyrimidine-2,4(1H,3H)-dione (**1h**)

A white foam, 79% yield. ^{1}H-NMR (CD_3OD, 600 MHz) δ 8.03 (2s, 2H, H-11, H-16), 7.52 (d, 1H, *J* = 8.0 Hz, H-6), 6.01-5.96 (m, 2H, H-1′, H-1″), 5.67 (d, 1H, *J* = 8.0 Hz, H-5), 4.48 (t, 2H, *J* = 4.4 Hz, H-3′, H-3″), 4.30 (t, 2H, *J* = 4.9 Hz, H-2′, H-2″), 4.13-4.09 (m, 2H, H-4′, H-4″), 3.97 (t, 2H, *J* = 7.1 Hz, H-12), 3.85-3.77 (m, 4H, H-7, H-5a′, H-5a″), 3.68 (dd, 2H, *J* = 12.2, 4.3 Hz, H-5b′, H-5b″), 2.75 (q, 4H, *J* = 7.1 Hz, H-9, H-14), 2.10-2.04 (m, 2H, H-13), 2.02-1.96 (m, 2H, H-8). ^{13}C-NMR (CD_3OD, 100 MHz) δ 165.53 (C=O, C-4), 152.95 (C=O, C-2), 148.56, 148.07 (2C, C-10, C-15), 145.47 (CH, C-6), 122.20, 122.05 (2CH, C-11, C-16), 101.69 (CH, C-5), 94.33 (2CH, C-1′, C-1″), 87.12 (2CH, C-4′, C-4″), 77.01 (2CH, C-3′, C-3″), 71.98, 71.95 (2CH, C-2′, C-2″), 63.02, 62.95 ($2CH_2$, C-5′, C-5″), 50.25, 41.70 ($2CH_2$, C-7, C-12), 29.17, 27.96

(2CH$_2$, C-9, C-14), 23.91, 23.34 (2CH$_2$, C-8, C-13). MALDI MS *m*/*z*: calcd for $C_{24}H_{34}N_8NaO_{10}$ 617.5; found 617.0 [M + Na]$^+$. Elemental Analysis calcd for $C_{24}H_{34}N_8O_{10}$, C 48.48, H 5.76, N 18.85%; found C 48.67, H 5.83, N 18.93%.

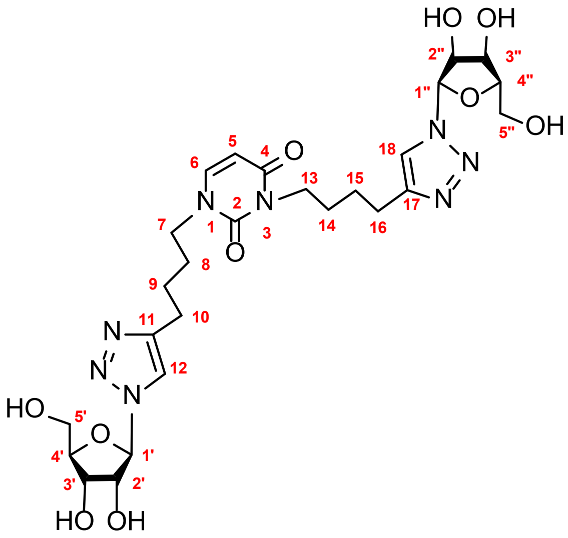

1,3-Bis{4-[1-(β-D-ribofuranosyl)-1H-1,2,3-triazol-4-yl]butyl}pyrimidine-2,4(1H,3H)-dione (**1i**)

A white foam, 71% yield. ^{1}H-NMR (CD$_3$OD, 400 MHz) *δ* 8.00 (s, 2H, H-12, H-18), 7.53 (d, 1H, *J* = 7.7 Hz, H-6), 5.99 (d, 2H, *J* = 4.0 Hz, H-1′, H-1″), 5.69 (d, 1H, *J* = 7.7 Hz, H-5), 4.48 (t, 2H, *J* = 4.6 Hz, H-3′, H-3″), 4.30 (t, 2H, *J* = 5.0 Hz, H-2′, H-2″), 4.11 (q, 2H, *J* = 4.4 Hz, H-4′, H-4″), 3.93 (t, 2H, *J* = 6.8 Hz, H-13), 3.83-3.76 (m, 4H, H-7, H-5a′, H-5a″), 3.68 (dd, 2H, *J* = 12.1, 4.1 Hz, H-5b′, H-5b″), 2.74 (t, 4H, *J* = 6.6 Hz, H-10, H-16), 1.76-1.61 (m, 8H, H-8, H-9, H-14, H-15). ^{13}C-NMR (CD$_3$OD, 100 MHz) *δ* 165.53 (C=O, C-4), 152.95 (C=O, C-2), 148.92, 148.73 (2C, C-11, C-17), 145.39 (CH, C-6), 122.11, 122.09 (2CH, C-12, C-18), 101.70 (CH, C-5), 94.32 (2CH, C-1′, C-1″), 87.14, 87.11 (2CH, C-4′, C-4″), 77.03 (2CH, C-3′, C-3″), 71.96 (2CH, C-2′, C-2″), 62.99, 62.95 (2CH$_2$, C-5′, C-5″), 50.40, 41.77 (2CH$_2$, C-7, C-13), 29.25, 27.87, 27.65, 27.22, 25.82, 25.74 (6CH$_2$, C-8, C-9, C-10, C-14, C-15, C-16). MALDI MS *m*/*z*: calcd for $C_{26}H_{39}N_8O_{10}$ 623.6; found 623.3 [M + H]$^+$. Elemental Analysis calcd for $C_{26}H_{38}N_8O_{10}$, C 50.16, H 6.15, N 18.00%; found C 50.23, H 6.54, N 18.05%.

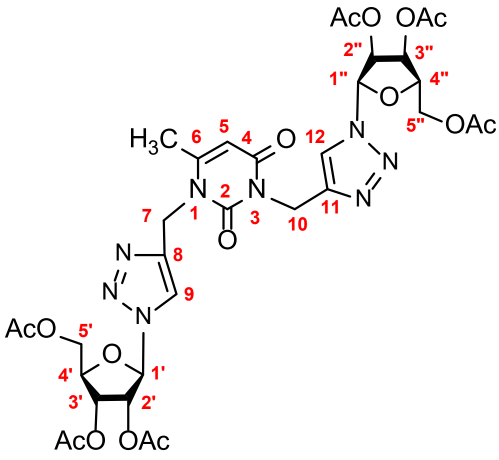

6-Methyl-1,3-bis{[1-(2,3,5-tri-O-acetyl-β-D-ribofuranosyl)-1H-1,2,3-triazol-4-yl]methyl}pyrimidine-2,4(1H,3H)-dione (**2d**)

A white foam, 85% yield. ^{1}H-NMR (CDCl$_3$, 400 MHz) *δ* 7.95, 7.77 (2s, 2H, H-9, H-12), 6.12, 6.09 (2d, 2H, *J* = 3.8 Hz, H-1′, H-1″), 5.82-5.76 (m, 2H, H-3′, H-3″), 5.63-5.57 (m, 3H, H-5, H-2′, H-2″), 5.27-5.16 (m, 2H, H-10), 5.15-5.03 (m, 2H, H-7), 4.50-4.43 (m, 2H, H-4′, H-

4''), 4.42-4.35 (m, 2H, H-5a', H-5a''), 4.26-4.16 (m, 2H, H-5b', H-5b''), 2.50 (s, 3H, CH_3-6), 2.11, 2.11, 2.10, 2.10, 2.09, 2.09 (6s, 18H, 6OAc). ^{13}C-NMR ($CDCl_3$, 100 MHz) δ 170.38, 170.30, 169.31, 169.11 (6C=O, $OCOCH_3$), 161.54 (C=O, C-4), 152.02 (C=O, C-2), 143.54, 143.45 (2C, C-8, C-11), 142.90 (C, C-6), 123.69, 122.74 (2CH, C-9, C-12), 101.73 (CH, C-5), 90.09, 89.95 (2CH, C-1', C-1''), 80.98, 80.80 (2CH, C-4', C-4''), 74.29 (2CH, C-3', C-3''), 70.79, 70.73 (2CH, C-2', C-2''), 62.87, 62.79 (2CH_2, C-5', C-5''), 39.93, 36.00 (2CH_2, C-7, C-10), 20.53 (CH_3, CH_3-6), 20.33, 20.26, 20.21 (6CH_3, $OCOCH_3$). MALDI MS *m/z*: calcd for $C_{33}H_{41}N_8O_{16}$ 805.7, $C_{33}H_{40}N_8NaO_{16}$ 827.7, $C_{33}H_{40}N_8KO_{16}$ 843.8; found 805.5 $[M + H]^+$, 827.5 $[M + Na]^+$, 843.5 $[M + K]^+$. Elemental Analysis calcd for $C_{33}H_{40}N_8O_{16}$, C 49.25, H 5.01, N 13.92%; found C 49.48, H 5.09, N 14.00%.

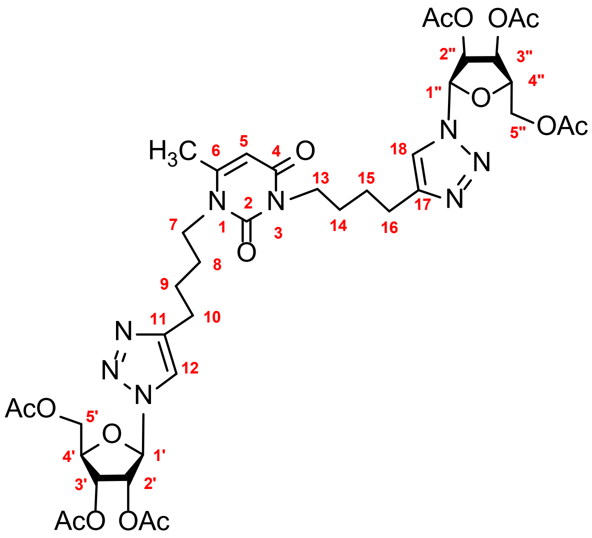

6-Methyl-1,3-bis{4-[1-(2,3,5-tri-O-acetyl-β-D-ribofuranosyl)-1H-1,2,3-triazol-4-yl]butyl}pyrimidine-2,4(1H,3H)-dione (**2f**)

A white foam, 71% yield. ^{1}H-NMR ($CDCl_3$, 400 MHz) δ 7.52, 7.50 (2s, 2H, H-12, H-18), 6.11, 6.10 (2d, 2H, *J* = 3.9 Hz, H-1', H-1''), 5.83-5.78 (m, 2H, H-3', H-3''), 5.60 (q, 2H, *J* = 5.1 Hz, H-2', H-2''), 5.4 (s, 1H, H-5), 4.47-4.41 (m, 2H, H-4', H-4''), 4.41-4.36 (m, 2H, H-5a', H-5a''), 4.21 (dd, 2H, *J* = 12.3, 4.6 Hz, H-5b', H-5b''), 3.94 (t, 2H, *J* = 6.6 Hz, H-13), 3.81 (t, 2H, *J* = 7.3 Hz, H-7), 2.80-2.73 (m, 4H, H-10, H-16), 2.21 (s, 3H, CH_3-6), 2.13-2.05 (m, 18 H, 6OAc), 1.80-1.66 (m, 8H, H-8, H-9, H-14, H-15). ^{13}C-NMR ($CDCl_3$, 100 MHz) δ 170.37, 170.29, 169.39, 169.37, 169.25,169.20 (6C=O, $OCOCH_3$), 162.14 (C=O, C-4), 152.02 (C=O, C-2), 151.05 (C, C-6), 148.26, 147.68 (2C, C-11, C-17), 120.16, 120.11 (2CH, C-12, C-18), 101.72 (CH, C-5), 89.89, 89.78 (2CH, C-1', C-1''), 80.76 (2CH, C-4', C-4''), 74.26 (2CH, C-3', C-3''), 70.85, 70.78 (2CH, C-2', C-2''), 62.96 (2CH_2, C-5', C-5''), 44.79, 40.73 (2CH_2, C-7, C-13), 28.25, 26.96, 26.49, 26.35, 25.09, 24.92 (6CH_2, C-8, C-9, C-10, C-14, C-15, C-16), 20.65, 20.43, 20.37 (6CH_3, $OCOCH_3$), 19.70 (CH_3, CH_3-6). MALDI MS *m/z*: calcd for $C_{39}H_{52}N_8NaO_{16}$ 911.8; found 911.7 $[M + Na]^+$. Elemental Analysis calcd for $C_{39}H_{52}N_8O_{16}$, C 52.70, H 5.90, N 12.61%; found C 52.81, H 5.95, N 12.67%.

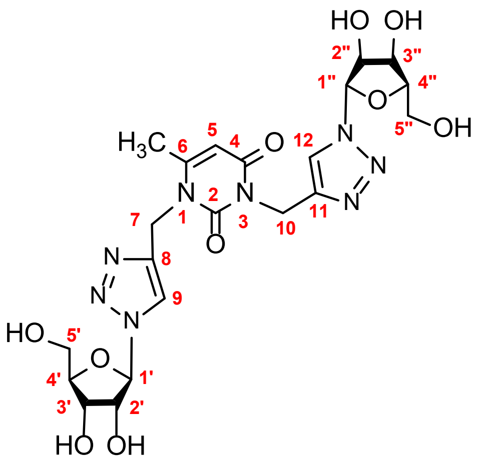

6-Methyl-1,3-bis{[1-(β-D-ribofuranosyl)-1H-1,2,3-triazol-4-yl]methyl}pyrimidine-2,4(1H,3H)-dione (**2g**)

A white foam, 88% yield. ^{1}H-NMR (CD_3OD, 600 MHz) δ 8.25, 8.13 (2s, 2H, H-9, H-12), 6.01, 5.98 (2d, 2H, J = 3.8 Hz, H-1′, H-1″), 5.67 (s, 1H, H-5), 5.20-5.15 (m, 4H, H-10, H-7), 4.48-4.44 (m, 2H, H-3′, H-3″), 4.32-4.27 (m, 2H, H-2′, H-2″), 4.13-4.08 (m, 2H, H-4′, H-4″), 3.82-3.76 (m, 2H, H-5a′, H-5a″), 3.70-3.64 (m, 2H, H-5b′, H-5b″), 2.44 (s, 3H, CH_3-6). ^{13}C-NMR (CD_3OD, 100 MHz) δ 163.96 (C=O, C-4), 155.20 (C=O, C-2), 153.37 (C, C-6), 144.70, 144.38 (2C, C-8, C-11), 124.09, 123.87 (2CH, C-9, C-12), 102.01 (CH, C-5), 94.47, 94.41 (2CH, C-1′, C-1″), 87.25, 87.21 (2CH, C-4′, C-4″), 77.08, 77.04 (2CH, C-3′, C-3″), 71.93, 71.87 (2CH, C-2′, C-2″), 62.90, 62.80 ($2CH_2$, C-5′, C-5″), 41.25, 37.19 ($2CH_2$, C-7, C-10), 20.17 (CH_3, CH_3-6). MALDI MS *m/z*: calcd for $C_{21}H_{29}N_8O_{10}$ 553.5; found 553.2 $[M + H]^+$. Elemental Analysis calcd for $C_{21}H_{28}N_8O_{10}$, C 45.65, H 5.11, N 20.28%; found C 45.76, H 5.18, N 20.29%.

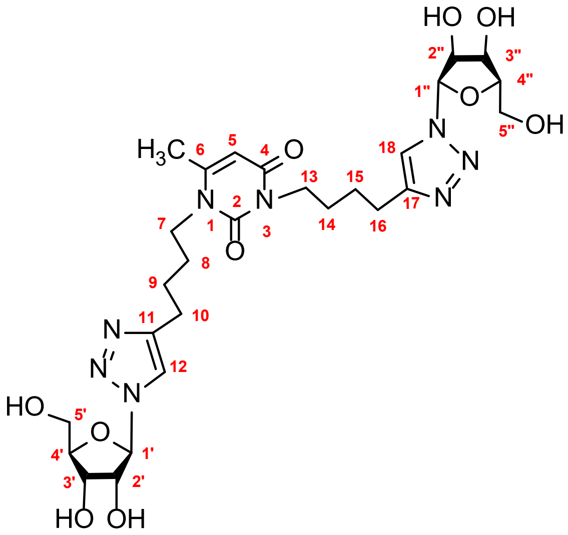

6-Methyl-1,3-Bis{4-[1-(2,3,5-tri-O-acetyl-β-D-ribofuranosyl)-1H-1,2,3-triazol-4-yl]butyl}pyrimidine-2,4(1H,3H)-dione (**2i**)

A white foam, 78% yield. ^{1}H-NMR (CD_3OD, 400 MHz) δ 8.02, 8.00 (2s, 2H, H-12, H-18), 5.99 (t, 2H, J = 3.6 Hz, H-1′, H-1″), 5.60 (s, 1H, H-5), 4.49-4.45 (m, 2H, H-3′, H-3″), 4.30 (t, 2H, J = 5.1 Hz, H-2′, H-2″), 4.13-4.09 (m, 2H, H-4′, H-4″), 3.92 (t, 2H, J = 6.8 Hz, H-13), 3.87 (t, 2H, J = 7.3 Hz, H-7), 3.80 (dd, 2H, J = 12.2, 3.2 Hz, H-5a′, H-5a″), 3.68 (dd, 2H, J = 12.3, 4.4 Hz, H-5b′, H-5b″), 2.80-2.72 (m, 4H, H-10, H-16), 2.28 (s, 3H, CH_3-6), 1.77-1.61 (m, 8H, H-8, H-9, H-14, H-15). ^{13}C-NMR (CD_3OD, 100 MHz) δ 164.69 (C=O, C-4), 154.92 (C=O, C-2), 153.46 (C, C-6), 148.96, 148.78 (2C, C-11, C-17), 122.14, 122.09 (2CH, C-12, C-18), 101.81 (CH, C-5), 94.96 (2CH, C-1′, C-1″), 87.17, 87.14 (2CH, C-4′, C-4″), 77.07, 77.05 (2CH, C-3′, C-3″), 71.99 (2CH, C-2′, C-2″), 62.99, 62.96 ($2CH_2$, C-5′, C-5″), 45.99, 41.90 ($2CH_2$, C-7,

C-13), 28.94, 27.90, 27.66, 27.43, 25.84, 25.76 (6CH$_2$, C-8, C-9, C-10, C-14, C-15, C-16), 19.78 (CH$_3$, CH$_3$-6). MALDI MS *m/z*: calcd for $C_{27}H_{40}N_8NaO_{10}$ 659.7; found 660.2 [M + Na]$^+$. Elemental Analysis calcd for $C_{27}H_{40}N_8O_{10}$, C 50.94, H 6.33, N 17.60%; found C 51.01, H 6.85, N 17.68%.

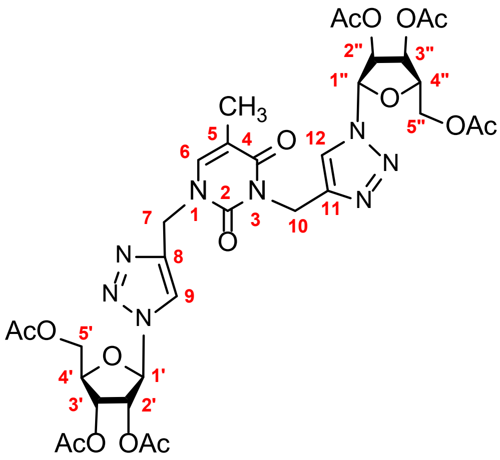

5-Methyl-1,3-bis{[1-(2,3,5-tri-O-acetyl-β-D-ribofuranosyl)-1H-1,2,3-triazol-4-yl]methyl}pyrimidine-2,4(1H,3H)-dione (**3d**)

A white foam, 90% yield. ^{1}H-NMR (CDCl$_3$, 400 MHz) δ 7.91, 7.78 (2s, 2H, H-9, H-12), 7.30 (s, 1H, H-6), 6.12, 6.10 (2d, 2H, *J* = 3.8 Hz, H-1′, H-1′′), 5.83-5.76 (m, 2H, H-3′, H-3′′), 5.62-5.66 (m, 2H, H-2′, H-2′′), 5.30-5.18 (m, 2H, H-10), 5.06-4.91 (m, 2H, H-7), 4.50-4.42 (m, 2H, H-4′, H-4′′), 4.42-4.36 (m, 2H, H-5a′, H-5a′′), 4.26-4.16 (m, 2H, H-5b′, H-5b′′), 2.12, 2.11, 2.10, 2.10, 2.09, 2.09 (6s, 18H, 6OAc), 1.91 (s, 3H, CH$_3$-5). ^{13}C-NMR (CDCl$_3$, 100 MHz) δ 170.46, 170.35, 169.38, 169.36, 169.17 (6C=O, OCOCH$_3$), 163.24 (C=O, C-4), 151.11 (C=O, C-2), 143.57, 142.57 (2C, C-8, C-11), 138.47 (CH, C-6), 123.25, 122.87 (2CH, C-9, C-12), 110.45 (C, C-5), 90.19, 89.97 (2CH, C-1′, C-1′′), 81.06, 80.90 (2CH, C-4′, C-4′′), 74.37, 74.34 (2CH, C-3′, C-3′′), 70.82, 70.75 (2CH, C-2′, C-2′′), 62.89, 62.85 (2CH$_2$, C-5′, C-5′′), 43.79, 36.18 (2CH$_2$, C-7, C-10), 20.65, 20.44, 20.37 (6CH$_3$, OCOCH$_3$), 12.91 (CH$_3$, CH$_3$-5). MALDI MS *m/z*: calcd for $C_{33}H_{41}N_8O_{16}$ 805.7, $C_{33}H_{40}N_8NaO_{16}$ 827.7; found 805.3 [M + H]$^+$, 827.3 [M + Na]$^+$. Elemental Analysis calcd for $C_{33}H_{40}N_8O_{16}$, C 49.25, H 5.01, N 13.92%; found C 49.39, H 5.11, N 13.98%.

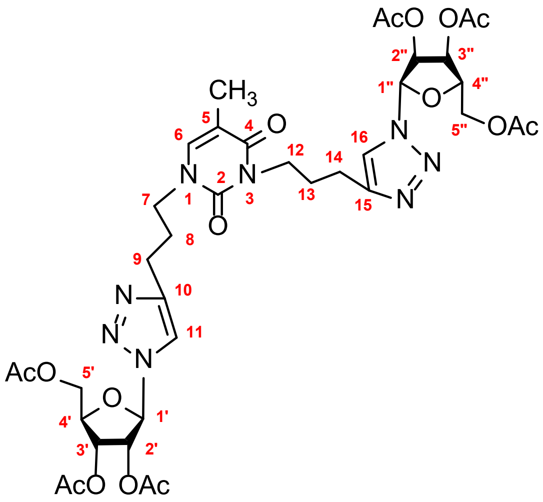

5-Methyl-1,3-bis{3-[1-(2,3,5-tri-O-acetyl-β-D-ribofuranosyl)-1H-1,2,3-triazol-4-yl]propyl}pyrimidine-2,4(1H,3H)-dione (**3e**)

A white foam, 92% yield. ^{1}H-NMR (CDCl$_3$, 400 MHz) δ 7.69, 7.57 (2s, 2H, H-11, H-16), 7.08 (d, 1H, *J* = 1.1 Hz, H-6), 6.11, 6.10 (2d, 2H, *J* = 3.5 Hz, H-1′, H-1′′), 5.82-5.78 (m, 2H, H-

3′, H-3″), 5.6 (q, 2H, *J* = 5.7 Hz, H-2′, H-2″), 4.47-4.41 (m, 2H, H-4′, H-4″), 4.38 (dd, 2H, *J* = 12.3, 2.7 Hz, H-5a′, H-5a″), 4.24-4.18 (m, 2H, H-5b′, H-5b″), 4.02 (t, 2H, *J* = 7.2 Hz, H-12), 3.79 (t, 2H, *J* = 7.2 Hz, H-7), 2.81-2.72 (m, 4H, H-9, H-14), 2.12-2.03 (m, 22 H, 6OAc, H-8, H-13), 1.89 (s, 3H, CH_3-5). ^{13}C-NMR ($CDCl_3$, 100 MHz) δ 170.28, 170.19, 169.32, 169.29, 169.16, 169.11 (6C=O, $OCOCH_3$), 163.67 (C=O, C-4), 151.36 (C=O, C-2), 147.77, 146.83 (2C, C-10, C-15), 138.69 (CH, C-6), 120.44, 120.23 (2CH, C-11, C-16), 109.56 (C, C-5), 89.78, 89.67 (2CH, C-1′, C-1″), 80.67, 80.61 (2CH, C-4′, C-4″), 74.14, 74.12 (2CH, C-3′, C-3″), 70.77, 70.70 (2CH, C-2′, C-2″), 62.94, 62.90 ($2CH_2$, C-5′, C-5″), 48.42, 40.55 ($2CH_2$, C-7, C-12), 28.11, 26.58 ($2CH_2$, C-9, C-14), 22.96, 22.10 ($2CH_2$, C-8, C-13), 20.55, 20.33, 20.25 ($6CH_3$, $OCOCH_3$), 12.81 (CH_3, CH_3-5). MALDI MS *m*/*z*: calcd for $C_{37}H_{49}N_8O_{16}$ 861.8; found 861.7 $[M + H]^+$. Elemental Analysis calcd for $C_{37}H_{48}N_8O_{16}$, C 51.63, H 5.62, N 13.02%; found C 51.78, H 5.74, N 13.08%.

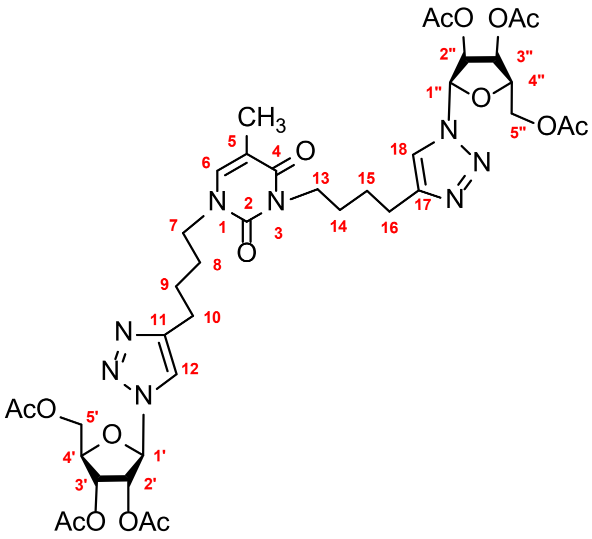

5-Methyl-1,3-bis{4-[1-(2,3,5-tri-O-acetyl-β-D-ribofuranosyl)-1H-1,2,3-triazol-4-yl]butyl}pyrimidine-2,4(1H,3H)-dione (**3f**)

A white foam, 75% yield. ^{1}H-NMR ($CDCl_3$, 400 MHz) δ 7.51, 7.48 (2s, 2H, H-12, H-18), 6.94 (d, 1H, *J* = 1.1 Hz, H-6), 6.10-6.07 (m, 2H, H-1′, H-1″), 5.81-5.76 (m, 2H, H-3′, H-3″), 5.61-5.56 (m, 2H, H-2′, H-2″), 4.46-4.40 (m, 2H, H-4′, H-4″), 4.40-4.34 (m, 2H, H-5a′, H-5a″), 4.22-4.16 (m, 2H, H-5b′, H-5b″), 3.95 (t, 2H, *J* = 6.4 Hz, H-13), 3.70 (t, 2H, *J* = 6.2 Hz, H-7), 2.80-2.70 (m, 4H, H-10, H-16), 2.10-2.02 (m, 18H, 6OAc), 1.88 (s, 3H, CH_3-6), 1.77-1.63 (m, 8H, H-8, H-9, H-14, H-15). ^{13}C-NMR ($CDCl_3$, 100 MHz) δ 170.32, 170.26, 169.37, 169.35, 169.23, 169.18 (6C=O, $OCOCH_3$), 163.67 (C=O, C-4), 151.32 (C=O, C-2), 148.19, 147.60 (2C, C-11, C-17), 138.32 (CH, C-6), 120.17, 120.08 (2CH, C-12, C-18), 109.72 (C, C-5), 89.81, 89.72 (2CH, C-1′, C-1″), 80.68 (2CH, C-4′, C-4″), 74.19 (2CH, C-3′, C-3″), 70.77, 70.71 (2CH, C-2′, C-2″), 62.89 ($2CH_2$, C-5′, C-5″), 49.01, 40.86 ($2CH_2$, C-7, C-13), 28.38, 26.94, 26.49, 26.05, 25.04, 24.85 ($6CH_2$, C-8, C-9, C-10, C-14, C-15, C-16), 20.60, 20.38, 20.32 ($6CH_3$, $OCOCH_3$), 12.92 (CH_3, CH_3-5). MALDI MS *m*/*z*: calcd for $C_{39}H_{53}N_8O_{16}$ 889.9, $C_{39}H_{52}N_8NaO_{16}$ 911.8; found 889.3 $[M + H]^+$, 911.3 $[M + Na]^+$. Elemental Analysis calcd for $C_{39}H_{52}N_8O_{16}$, C 52.70, H 5.90, N 12.61%; found C 52.79, H 5.98, N 12.69%.

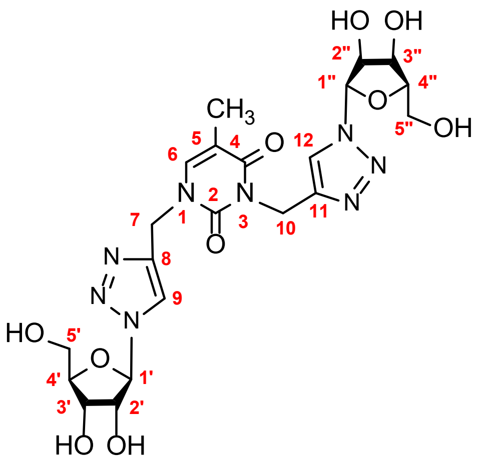

5-Methyl-1,3-bis{[1-(β-D-ribofuranosyl)-1H-1,2,3-triazol-4-yl]methyl}pyrimidine-2,4(1H,3H)-dione (**3g**)

A white foam, 75% yield. ^{1}H-NMR (CD_3OD, 400 MHz) δ 8.26, 8.13 (2s, 2H, H-9, H-12), 7.56 (s, 1H, H-5), 6.01, 5.98 (2d, 2H, *J* = 3.8 Hz, H-1′, H-1″), 5.20 (s, 2H, H-10), 5.03 (s, 2H, H-7), 4.50-4.43 (m, 2H, H-3′, H-3″), 4.32-4.26 (m, 2H, H-2′, H-2″), 4.13-4.07 (m, 2H, H-4′, H-4″), 3.83-3.75 (m, 2H, H-5a′, H-5a″), 3.72-3.62 (m, 2H, H-5b′, H-5b″), 1.88 (s, 3H, CH_3-5). ^{13}C-NMR (CD_3OD, 100 MHz) δ 165.21 (C=O, C-4), 152.65 (C=O, C-2), 144.10 (2C, C-8, C-11), 141.21 (CH, C-6), 124.07, 123.90 (2CH, C-9, C-12), 110.97 (C, C-5), 94.47, 94.37 (2CH, C-1′, C-1″), 87.25, 87.18 (2CH, C-4′, C-4″), 77.07, 77.01 (2CH, C-3′, C-3″), 71.91, 71.89 (2CH, C-2′, C-2″), 62.89, 62.81 (2CH_2, C-5′, C-5″), 44.90, 37.26 (2CH_2, C-7, C-10), 12.94 (CH_3, CH_3-5). MALDI MS *m*/*z*: calcd for $C_{21}H_{29}N_8O_{10}$ 553.5; found 553.2 $[M + H]^+$. Elemental Analysis calcd for $C_{21}H_{28}N_8O_{10}$, C 45.65, H 5.11, N 20.28%; found C 45.70, H 5.19, N 20.31%.

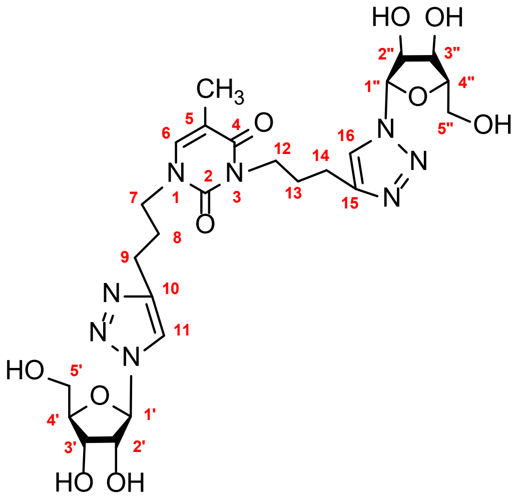

5-Methyl-1,3-bis{3-[1-(β-D-ribofuranosyl)-1H-1,2,3-triazol-4-yl]propyl}pyrimidine-2,4(1H,3H)-dione (**3h**)

A white foam, 78% yield. ^{1}H-NMR (CD_3OD, 400 MHz) δ 8.03 (2s, 2H, H-11, H-16), 7.39 (d, 1H, *J* = 1.1 Hz, H-6), 6.00-5.96 (m, 2H, H-1′, H-1″), 4.50-4.44 (m, 2H, H-3′, H-3″), 4.34-4.27 (m, 2H, H-2′, H-2″), 4.11 (q, 2H, *J* = 4.4 Hz, H-4′, H-4″), 3.99 (t, 2H, *J* = 7.2 Hz, H-12), 3.83-3.76 (m, 4H, H-7, H-5a′, H-5a″), 3.71-3.65 (m, 2H, H-5b′, H-5b″), 2.79-2.71 (m, 4H, H-9, H-14), 2.10-1.95 (m, 4H, H-8, H-13), 1.87 (s, 3H, CH_3-5). ^{13}C-NMR (Pyridine-d_5, 100 MHz) δ 163.78 (C=O, C-4), 151.57 (C=O, C-2), 147.69, 147.07 (2C, C-10, C-15), 139.54 (CH, C-6), 120.66, 120.44 (2CH, C-11, C-16), 108.94 (C, C-5), 94.05, 93.97 (2CH, C-1′, C-1″), 87.18 (2CH, C-4′, C-4″), 77.14, 77.04 (2CH, C-3′, C-3″), 71.87, 71.80 (2CH, C-2′, C-2″), 62.42, 62.26 (2CH_2, C-5′, C-5″), 48.72, 40.97 (2CH_2, C-7, C-12), 28.73, 27.56 (2CH_2, C-9, C-14), 23.69, 22.80 (2CH_2, C-8, C-13), 12.97 (CH_3, CH_3-5). MALDI MS *m*/*z*: calcd for $C_{25}H_{36}N_8NaO_{10}$ 631.6;

found 631.4 [M + Na]+. Elemental Analysis calcd for $C_{25}H_{36}N_8O_{10}$, C 49.34, H 5.96, N 18.41%; found C 49.57, H 6.00, N 18.45%.

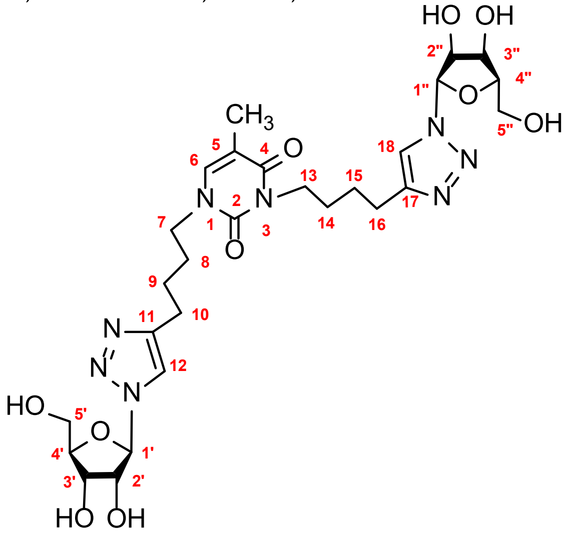

5-Methyl-1,3-bis{4-[1-(2,3,5-tri-O-acetyl-β-D-ribofuranosyl)-1H-1,2,3-triazol-4-yl]butyl}pyrimidine-2,4(1H,3H)-dione (**3i**)

A white foam, 73% yield. ^{1}H-NMR (CD_3OD, 400 MHz) δ 8.00 (s, 2H, H-12, H-18), 7.41 (s, 1H, H-6), 5.99 (d, 2H, *J* = 3.9 Hz, H-1′, H-1″), 4.48 (t, 2H, *J* = 4.5 Hz, H-3′, H-3″), 4.34-4.26 (m, 2H, H-2′, H-2″), 4.14-4.07 (m, 2H, H-4′, H-4″), 3.94 (t, 2H, *J* = 6.6 Hz, H-13), 3.84-3.73 (m, 4H, H-7, H-5a′, H-5a″), 3.68 (dd, 2H, *J* = 12.1, 5.5 Hz, H-5b′, H-5b″), 2.74 (t, 4H, *J* = 5.9 Hz, H-10, H-16), 1.87 (s, 3H, CH_3-5), 1.76-1.58 (m, 8H, H-8, H-9, H-14, H-15). ^{13}C-NMR (CD_3OD, 100 MHz) δ 165.82 (C=O, C-4), 152.95 (C=O, C-2), 149.00, 148.87 (2C, C-11, C-17), 141.43 (CH, C-6), 122.15 (2CH, C-12, C-18), 110.48 (C, C-5), 94.30 (2CH, C-1′, C-1″), 87.14, 87.11 (2CH, C-4′, C-4″), 77.00 (2CH, C-3′, C-3″), 71.97 (2CH, C-2′, C-2″), 62.99, 62.95 (2CH_2, C-5′, C-5″), 50.11, 42.01 (2CH_2, C-7, C-13), 29.29, 27.92, 27.65, 27.2, 25.83, 25.75 (6CH_2, C-8, C-9, C-10, C-14, C-15, C-16), 12.94 (CH_3, CH_3-5). MALDI MS *m*/*z*: calcd for $C_{27}H_{40}N_8NaO_{10}$ 636.6; found 637.5 [M + Na]+. Elemental Analysis calcd for $C_{27}H_{40}N_8O_{10}$, C 50.94, H 6.33, N 17.60%; found C 51.98, H 6.47, N 17.69%.

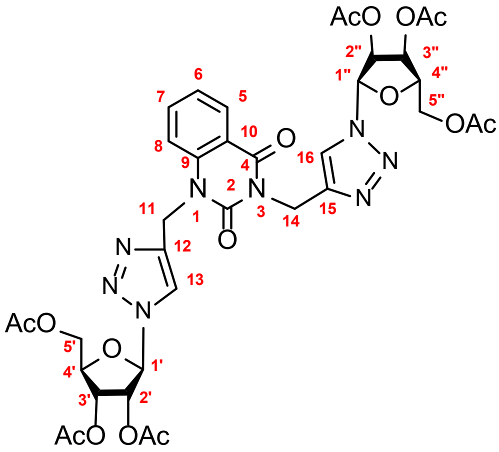

1,3-Bis{[1-(2,3,5-tri-O-acetyl-β-D-ribofuranosyl)-1H-1,2,3-triazol-4-yl]methyl}quinazoline-2,4(1H,3H)-dione (**4d**)

A white foam, 95% yield. ^{1}H-NMR ($CDCl_3$, 400 MHz) δ 8.18 (dd, 1H, *J* = 8.1, 1.1 Hz, H-Ar), 7.90, 7.85 (2s, 2H, H-13, H-16), 7.79-7.75 (m, 1H, H-Ar), 7.72-7.65 (m, 1H, H-Ar), 7.25-7.21 (m, 1H, H-Ar), 6.12, 6.11 (2d, 2H, *J* = 3.7 Hz, H-1′, H-1″), 5.81-5.75 (m, 2H, H-3′, H-3″), 5.63-5.57 (m, 2H, H-2′, H-2″), 5.46-5.36 (m, 4H, H-11, H-14), 4.48-4.42 (m, 2H, H-4′, H-4″), 4.41-4.33 (m, 2H, H-5a′, H-5a″), 4.20 (dd, 2H, *J* = 12.1, 4.0 Hz, H-5b′, H-5b″), 2.12-2.06 (m, 18H, 6OAc). ^{13}C-NMR ($CDCl_3$, 100 MHz) δ 170.37, 170.28, 169.30, 169.12, 169.09

(6C=O, $OCOCH_3$), 161.35 (C=O, C-4), 150.73 (C=O, C-2), 143.17 (2C, C-12, C-15), 139.62, 135.50, 128.89 (3C, C-8, C-9, C-10), 123.33, 123.24 (2CH, C-13, C-16), 122.70, 115.45, 114.61 (3C, C-5, C-6, C-7), 90.09, 89.99 (2CH, C-1′, C-1″), 80.96, 80.85 (2CH, C-4′, C-4″), 74.32 (2CH, C-3′, C-3″), 70.76 (2CH, C-2′, C-2″), 62.80 (2CH_2, C-5′, C-5″), 39.32, 36.56 (2CH_2, C-11, C-14), 20.50, 20.34, 20.27 (6CH_3, $OCOCH_3$). MALDI MS *m*/*z*: calcd for $C_{36}H_{41}N_8O_{16}$ 841.7, $C_{36}H_{40}N_8NaO_{16}$ 863.7, $C_{36}H_{40}N_8KO_{16}$ 879.8; found 841.1 $[M + H]^+$, 863.1 $[M + Na]^+$, 879.1 $[M + K]^+$. Elemental Analysis calcd for $C_{36}H_{40}N_8O_{16}$, C 51.43, H 4.80, N 13.33%; found C 51.48, H 4.91, N 13.37%.

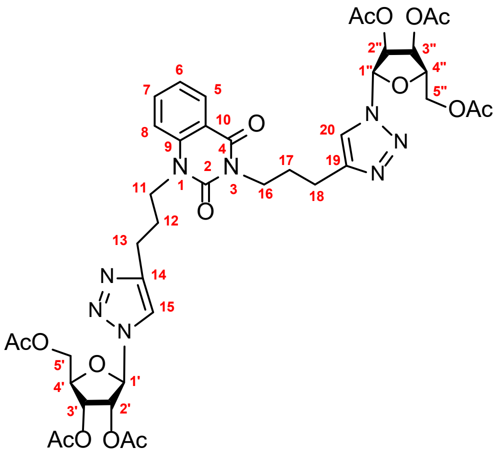

1,3-Bis{3-[1-(2,3,5-tri-O-acetyl-β-D-ribofuranosyl)-1H-1,2,3-triazol-4-yl]propyl}quinazoline-2,4(1H,3H)-dione (**4e**)

A white foam, 85% yield. ^{1}H-NMR ($CDCl_3$, 400 MHz) δ 8.20 (dd, 1H, *J* = 7.9, 1.3 Hz, H-Ar), 7.71, 7.62 (2s, 2H, H-15, H-20), 7.69-7.64 (m, 1H, H-Ar), 7.27-7.20 (m, 2H, H-Ar), 6.12 (t, 2H, *J* = 4.0 Hz, H-1′, H-1″), 5.85-5.78 (m, 2H, H-3′, H-3″), 5.65-5.59 (m, 2H, H-2′, H-2″), 4.50-4.43 (m, 2H, H-4′, H-4″), 4.42-4.35 (m, 2H, H-5a′, H-5a″), 4.27-4.20 (m, 4H, H-16, H-5b′, H-5b″), 4.17 (t, 2H, *J* = 7.2 Hz, H-11), 2.92-2.80 (m, 4H, H-13, H-18), 2.20-2.03 (m, 22H, H-12, H-17, 6OAc). ^{13}C-NMR ($CDCl_3$, 100 MHz) δ 170.33, 170.24, 169.35, 169.33, 169.19, 169.14 (6C=O, $OCOCH_3$), 161.69 (C=O, C-4), 150.77 (C=O, C-2), 147.79, 147.06 (2C, C-14, C-19), 139.61, 135.18, 128.98, 122.82 (4C, C-Ar), 120.44, 120.19 (2CH, C-15, C-20), 115.54, 113.67 (2C, C-Ar), 89.84, 89.75 (2CH, C-1′, C-1″), 80.73, 80.69 (2CH, C-4′, C-4″), 74.22, 74.19 (2CH, C-3′, C-3″), 70.83, 70.76 (2CH, C-2′, C-2″), 62.98, 62.94 (2CH_2, C-5′, C-5″), 43.05, 41.06 (2CH_2, C-11, C-16), 26.90, 26.36 (2CH_2, C-13, C-18), 23.03, 22.69 (2CH_2, C-12, C-17), 20.59, 20.37, 20.30 (6CH_3, $OCOCH_3$). MALDI MS *m*/*z*: calcd for $C_{40}H_{49}N_8O_{16}$ 897.8, $C_{40}H_{48}N_8NaO_{16}$ 919.8; found 897.4 $[M + H]^+$, 919.1 $[M + Na]^+$. Elemental Analysis calcd for $C_{40}H_{48}N_8O_{16}$, C 53.57, H 5.39, N 12.49%; found C 53.68, H 5.43, N 12.51%.

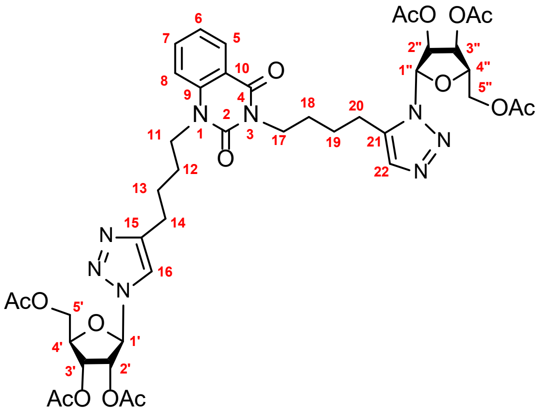

1,3-Bis{4-[1-(2,3,5-tri-O-acetyl-β-D-ribofuranosyl)-1H-1,2,3-triazol-4-yl]butyl}quinazoline-2,4(1H,3H)-dione (**4f**)

A white foam, 87% yield. ^{1}H-NMR ($CDCl_3$, 400 MHz) δ 8.17 (d, 1H, *J* = 6.6 Hz, H-Ar), 7.67-7.59 (m, 1H, H-Ar), 7.52, 7.49 (2s, 2H, H-16, H-22), 7.24-7.12 (m, 2H, H-Ar), 6.12-6.04 (m, 2H, H-1′, H-1″), 5.83-5.75 (m, 2H, H-3′, H-3″), 5.62-5.54 (m, 2H, H-2′, H-2″), 4.47-4.39 (m, 2H, H-4′, H-4″), 4.38-4.30 (m, 2H, H-5a′, H-5a″), 4.24-4.16 (m, 2H, H-5b′, H-5b″), 4.15-4.02 (m, 4H, H-11, H-17), 2.84-2.70 (m, 4H, H-14, H-20), 2.12-1.98 (m, 18H, 6OAc), 1.85-1.68 (m, 8H, H-12, H-13, H-18, H-19). ^{13}C-NMR ($CDCl_3$, 100 MHz) δ 170.28, 170.23, 169.33, 169.31, 169.17, 169.14 (6C=O, $OCOCH_3$), 161.63 (C=O, C-4), 150.70 (C=O, C-2), 148.18, 147.75 (2C, C-15, C-21), 139.65, 135.01, 129.03, 122.70 (4C, C-Ar), 120.12, 120.04 (2CH, C-16, C-22), 115.64, 113.50 (2C, C-Ar), 89.82, 89.76 (2CH, C-1′, C-1″), 80.71 (2CH, C-4′, C-4″), 74.21 (2CH, C-3′, C-3″), 70.79, 70.76 (2CH, C-2′, C-2″), 62.90 ($2CH_2$, C-5′, C-5″), 43.26, 41.30 ($2CH_2$, C-11, C-17), 27.20, 26.65, 26.51, 26.34, 25.08, 24.96 ($6CH_2$, C-12, C-13, C-14, C-18, C-19, C-20), 20.56, 20.35, 20.28 ($6CH_3$, $OCOCH_3$). MALDI MS *m*/*z*: calcd for $C_{42}H_{53}N_8O_{16}$ 925.9, $C_{42}H_{52}N_8NaO_{16}$ 947.9; found 925.3 $[M + H]^+$, 947.4 $[M + Na]^+$. Elemental Analysis calcd for $C_{42}H_{52}N_8O_{16}$, C 54.54, H 5.67, N 12.12%; found C 54.65, H 5.73, N 12.14%.

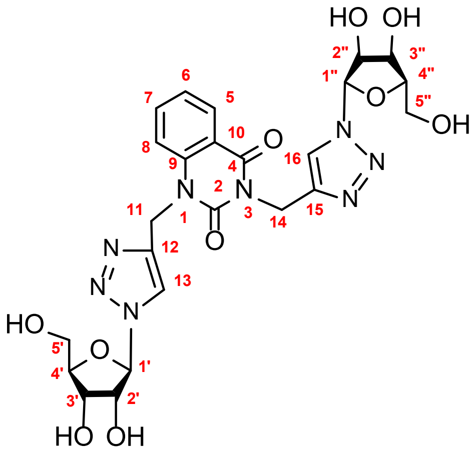

1,3-Bis{[1-(β-D-ribofuranosyl)-1H-1,2,3-triazol-4-yl]methyl}quinazoline-2,4(1H,3H)-dione (**4g**)

A white foam, 79% yield. ^{1}H-NMR (CD_3OD, 400 MHz) δ 8.25, 8.22 (2s, 2H, H-13, H-16), 8.14 (dd, 1H, *J* = 7.7, 1.5 Hz, H-Ar), 7.73-7.67 (m, 1H, H-Ar), 7.60-7.56 (m, 1H, H-Ar), 7.30-7.24 (m, 1H, H-Ar), 6.01-5.96 (m, 2H, H-1′, H-1″), 5.46 (s, 2H, H-14), 5.37 (s, 2H, H-11), 4.48-4.42 (m, 2H, H-3′, H-3″), 4.31-4.24 (m, 2H, H-2′, H-2″), 4.11-4.06 (m, 2H, H-4′, H-4″), 3.81-3.74 (m, 2H, H-5a′, H-5a″), 3.69-3.62 (m, 2H, H-5b′, H-5b″). ^{13}C-NMR (Pyridine-d_5, 100 MHz) δ 161.29 (C=O, C-4), 150.91 (C=O, C-2), 144.06, 143.41 (2C, C-12, C-15), 140.11, 135.22, 128.74 (3C, C-Ar), 120.01 (2CH, C-13, C-16), 115.94, 115.07 (3C, C-Ar), 94.25, 94.13 (2CH, C-1′, C-1″), 87.22, 87.16 (2CH, C-4′, C-4″), 77.08, 76.98 (2CH, C-3′, C-3″), 71.70, 71.63 (2CH, C-2′, C-2″), 62.12, 61.99 ($2CH_2$, C-5′, C-5″), 39.69, 37.20 ($2CH_2$, C-11, C-14). MALDI MS *m*/*z*: calcd for $C_{24}H_{28}N_8NaO_{10}$ 611.5; found 611.4 $[M + Na]^+$. Elemental Analysis calcd for $C_{24}H_{28}N_8O_{10}$, C 48.98, H 4.80, N 19.04%; found C 49.01, H 4.89, N 19.07%.

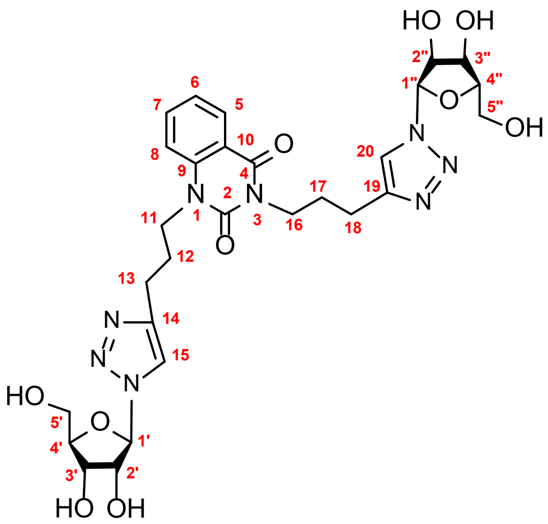

1,3-Bis{3-[1-(β-D-ribofuranosyl)-1H-1,2,3-triazol-4-yl]propyl}quinazoline-2,4(1H,3H)-dione (**4h**)

A white foam, 76% yield. ^{1}H-NMR (CD_3OD, 400 MHz) *δ* 8.13 (dd, 1H, *J* = 8.1, 1.5 Hz, H-Ar), 8.05, 8.04 (2s, 2H, H-15, H-20), 7.76-7.70 (m, 1H, H-Ar), 7.40-7.36 (m, 1H, H-Ar), 7.30-7.24 (m, 1H, H-Ar), 5.98, 5.95 (2d, 2H, *J* = 4.0 Hz, H-1′, H-1′′), 4.48-4.40 (m, 2H, H-3′, H-3′′), 4.32-4.27 (m, 2H, H-2′, H-2′′), 4.26-4.20 (m, 2H, H-4′, H-4′′), 4.15-4.07 (m, 4H, H-11, H-16), 3.79 (dd, 2H, *J* = 12.1, 3.3 Hz, H-5a′, H-5a′′), 3.68 (dd, 2H, *J* = 12.4, 4.4 Hz, H-5b′, H-5b′′), 2.89-2.75 (m, 4H, H-13, H-18), 2.17-2.01 (m, 4H, H-12, H-17). ^{13}C-NMR (CD_3OD, 100 MHz) *δ* 163.53 (C=O, C-4), 152.30 (C=O, C-2), 148.60, 148.31 (2C, C-14, C-19), 141.16, 136.72, 129.64, 124.15 (4C, C-Ar), 122.19, 122.05 (2CH, C-15, C-20), 116.64, 115.44 (2C, C-Ar), 94.36 (2CH, C-1′, C-1′′), 87.13, 87.05 (2CH, C-4′, C-4′′), 77.05, 77.04 (2CH, C-3′, C-3′′), 71.96, 71.95 (2CH, C-2′, C-2′′), 62.98 ($2CH_2$, C-5′, C-5′′), 44.16, 42.32 ($2CH_2$, C-11, C-16), 28.27, 27.75 ($2CH_2$, C-13, C-18), 23.96, 23.57 ($2CH_2$, C-12, C-17). MALDI MS *m*/*z*: calcd for $C_{28}H_{36}N_8NaO_{10}$ 667.6; found 667.2 $[M + Na]^+$. Elemental Analysis calcd for $C_{28}H_{36}N_8O_{10}$, C 52.17, H 5.63, N 17.38%; found C 52.47, H 5.71, N 17.40%.

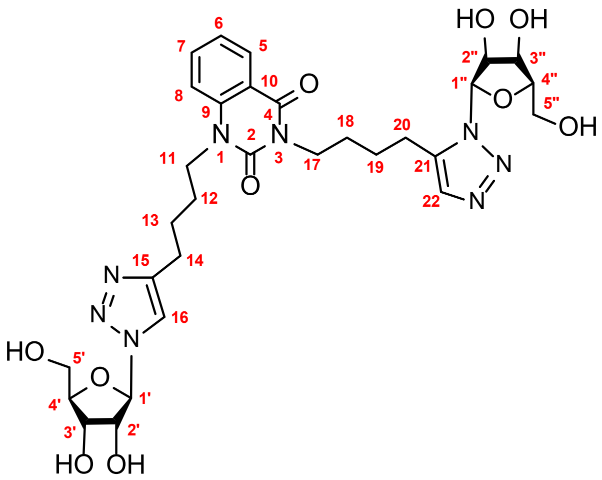

1,3-Bis{4-[1-(β-D-ribofuranosyl)-1H-1,2,3-triazol-4-yl]butyl}quinazoline-2,4(1H,3H)-dione (**4i**)

A white foam, 81% yield. ^{1}H-NMR (CD_3OD, 400 MHz) *δ* 8.13 (dd, 1H, *J* = 7.9, 1.3 Hz, H-Ar), 8.04 (s, 2H, H-16, H-22), 7.77-7.71 (m, 1H, H-Ar), 7.41-7.36 (m, 1H, H-Ar), 7.31-7.25 (m, 1H, H-Ar), 6.02 (d, 2H, *J* = 4.0 Hz, H-1′, H-1′′), 4.54-4.48 (m, 2H, H-3′, H-3′′), 4.36-4.31 (m, 2H, H-2′, H-2′′), 4.19 (t, 2H, *J* = 6.8 Hz, H-17), 4.16-4.12 (m, 2H, H-4′, H-4′′), 4.11-4.05 (m, 2H, H-11), 3.86-3.78 (m, 2H, H-5a′, H-5a′′), 3.74-3.68 (m, 2H, H-5b′, H-5b′′), 2.84-2.72 (m, 4H, H-14, H-20), 1.88-1.68 (m, 8H, H-12, H-13, H-18, H-19). ^{13}C-NMR (CD_3OD, 100

MHz) δ 163.46 (C=O, C-4), 152.22 (C=O, C-2), 148.95, 148.82 (2C, C-15, C-21), 141.14, 136.65, 129.60, 124.07 (4C, C-Ar), 122.11 (2CH, C-16, C-22), 116.66, 115.44 (2C, C-Ar), 94.31 (2CH, C-1′, C-1″), 87.13, 87.10 (2CH, C-4′, C-4″), 77.02, 77.00 (2CH, C-3′, C-3″), 71.97 (2CH, C-2′, C-2″), 62.99, 62.97 ($2CH_2$, C-5′, C-5″), 44.33, 42.41 ($2CH_2$, C-11, C-17), 28.14, 27.70, 27.65, 27.39, 25.85, 25.81 ($6CH_2$, C-12, C-13, C-14, C-18, C-19, C-20). MALDI MS *m/z*: calcd for $C_{30}H_{41}N_8O_{10}$ 673.7, $C_{30}H_{40}N_8NaO_{10}$ 695.6, $C_{30}H_{40}N_8KO_{10}$ 711.7; found 673.4 $[M + H]^+$, 695.5 $[M + Na]^+$, 711.5 $[M + K]^+$. Elemental Analysis calcd for $C_{30}H_{40}N_8O_{10}$, C 53.57, H 5.99, N 16.66%; found C 53.59, H 6.03, N 16.69%.

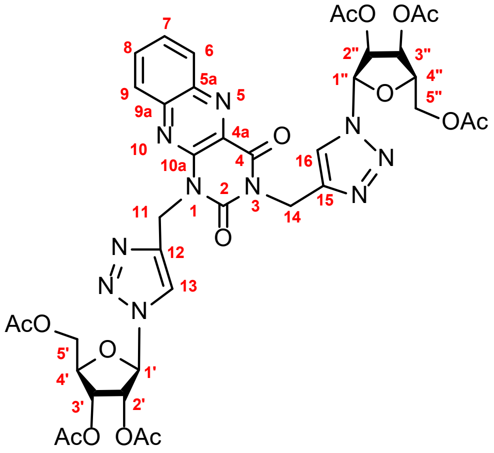

1,3-Bis{[1-(2,3,5-tri-O-acetyl-β-D-ribofuranosyl)-1H-1,2,3-triazol-4-yl]methyl}benzo[g]pteridine-2,4(1H,3H)-dione (**5d**)

A yellow foam, 43% yield. ^{1}H-NMR ($CDCl_3$, 600 MHz) δ 8.26 (d, 1H, *J* = 8.2 Hz, H-Ar), 8.07 (d, 1H, *J* = 8.8 Hz, H-Ar), 7.93 (s, 2H, H-13, H-16), 7.91-7.85 (m, 1H, H-Ar), 7.77-7.71 (m, 1H, H-Ar), 7.25-7.21 (m, 1H, H-Ar), 6.09 (t, 2H, *J* = 3.4 Hz, H-1′, H-1″), 5.87-5.74 (m, 4H, H-14, H-3′, H-3″), 5.61-5.55 (m, 2H, H-2′, H-2″), 5.54-5.42 (m, 2H, H-11), 4.46-4.40 (m, 2H, H-4′, H-4″), 4.38-4.30 (m, 2H, H-5a′, H-5a″), 4.22-4.14 (m, 2H, H-5b′, H-5b″), 2.15-2.03 (m, 18H, 6OAc). ^{13}C-NMR ($CDCl_3$, 100 MHz) δ 170.40, 170.31, 169.31, 169.14, 169.11 (6C=O, $OCOCH_3$), 159.23 (C=O, C-4), 149.90 (C=O, C-2), 144.56, 143.17 (2C, C-10a, C-4a), 142.80 (2C, C-12, C-15), 140.24, 134.06, 130.64, 129.58, 129.42, 128.09 (6C, C-Ar), 123.33, 123.19 (2CH, C-13, C-16), 90.00, 89.98 (2CH, C-1′, C-1″), 80.97, 80.96 (2CH, C-4′, C-4″), 74.28 (2CH, C-3′, C-3″), 70.84, 70.78 (2CH, C-2′, C-2″), 62.93, 62.95 ($2CH_2$, C-5′, C-5″), 37.42, 37.19 ($2CH_2$, C-11, C-14), 20.62, 20.56, 20.38, 20.31 ($6CH_3$, $OCOCH_3$). MALDI MS *m/z*: calcd for $C_{38}H_{41}N_{10}O_{16}$ 893.7, $C_{38}H_{40}N_{10}NaO_{16}$ 915.7; found 893.3 $[M + H]^+$, 915.3 $[M + Na]^+$. Elemental Analysis calcd for $C_{38}H_{40}N_{10}O_{16}$, C 51.12, H 4.52, N 15.69%; found C 51.21, H 4.55, N 15.71%.

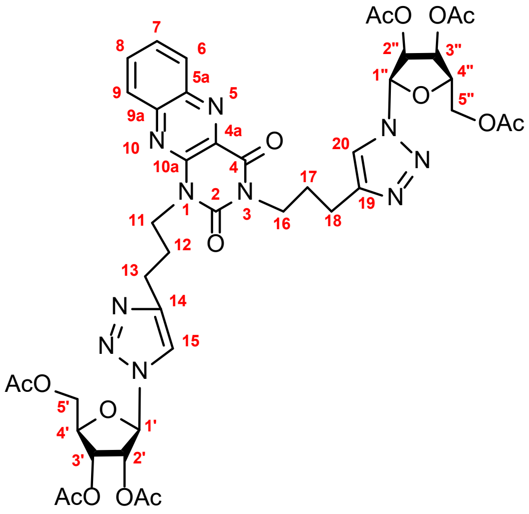

1,3-Bis{3-[1-(2,3,5-tri-O-acetyl-β-D-ribofuranosyl)-1H-1,2,3-triazol-4-yl]propyl}benzo[g]pteridine-2,4(1H,3H)-dione (**5e**)

A yellow foam, 73% yield. ^{1}H-NMR ($CDCl_3$, 400 MHz) δ 8.31 (dd, 1H, J = 8.4, 0.7 Hz, H-Ar), 8.01-7.97 (m, 1H, H-Ar), 7.91-7.86 (m, 1H, H-Ar), 7.77-7.71 (m, 2H, H-15, H-Ar), 7.61 (s, 1H, H-20), 6.12, 6.10 (2d, 2H, J = 3.8 Hz, H-1′, H-1″), 5.85-5.79 (m, 2H, H-3′, H-3″), 5.63-5.57 (m, 2H, H-2′, H-2″), 4.54 (t, 2H, J = 7.3 Hz, H-16), 4.47-4.39 (m, 2H, H-4′, H-4″), 4.39-4.33 (m, 2H, H-5a′, H-5a″), 4.28-4.17 (m, 4H, H-11, H-5b′, H-5b″), 2.94-2.84 (m, 4H, H-13, H-18), 2.28-2.12 (m, 4H, H-12, H-17), 2.11-2.01 (m, 18H, 6OAc). ^{13}C-NMR ($CDCl_3$, 100 MHz) δ 170.39, 170.28, 169.37, 169.35, 169.22, 169.16 (6C=O, $OCOCH_3$), 159.68 (C=O, C-4), 150.21 (C=O, C-2), 147.55, 147.48 (2C, C-14, C-19), 144.93, 143.33, 140.06, 133.87, 130.70, 129.60, 129.17, 127.94 (8C, C-Ar), 120.59, 120.18 (2CH, C-15, C-20), 89.84, 89.73 (2CH, C-1′, C-1″), 80.78, 80.73 (2CH, C-4′, C-4″), 74.24, 74.19 (2CH, C-3′, C-3″), 70.89, 70.80 (2CH, C-2′, C-2″), 62.98 ($2CH_2$, C-5′, C-5″), 42.18, 41.84 ($2CH_2$, C-11, C-16), 26.89, 26.85 ($2CH_2$, C-13, C-18), 23.07, 23.00 ($2CH_2$, C-12, C-17), 20.66, 20.62, 20.42, 20.34 ($6CH_3$, $OCOCH_3$). MALDI MS *m/z*: calcd for $C_{42}H_{49}N_{10}O_{16}$ 949.8, $C_{42}H_{48}N_{10}NaO_{16}$ 971.8, $C_{42}H_{48}N_{10}KO_{16}$ 987.9; found 949.6 $[M + H]^+$, 971.6 $[M + Na]^+$, 987.6 $[M + K]^+$. Elemental Analysis calcd for $C_{42}H_{48}N_{10}O_{16}$, C 53.16, H 5.10, N 14.76%; found C 53.31, H 5.19, N 14.77%.

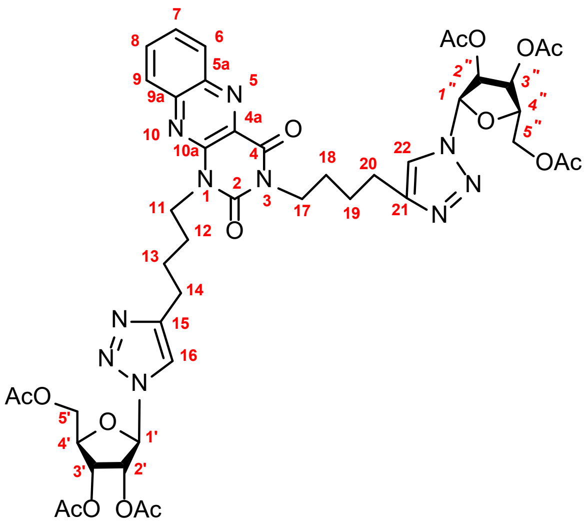

1,3-Bis{4-[1-(2,3,5-tri-O-acetyl-β-D-ribofuranosyl)-1H-1,2,3-triazol-4-yl]butyl}benzo[g]pteridine-2,4(1H,3H)-dione (**5f**)

A yellow foam, 82% yield. ^{1}H-NMR ($CDCl_3$, 400 MHz) δ 8.29 (d, 1H, J = 8.4 Hz, H-Ar), 8.02-7.98 (m, 1H, H-Ar), 7.90-7.84 (m, 1H, H-Ar), 7.75-7.69 (m, 1H, H-Ar), 7.54, 7.49 (2s, 2H, H-16, H-22), 6.11-6.06 (m, 2H, H-1′, H-1″), 5.82-5.76 (m, 2H, H-3′, H-3″), 5.61-5.55 (m,

2H, H-2′, H-2″), 4.50-4.40 (m, 4H, H-17, H-4′, H-4″), 4.40-4.32 (m, 2H, H-5a′, H-5a″), 4.24-4.14 (m, 4H, H-11, H-5b′, H-5b″), 2.88-2.74 (m, 4H, H-14, H-20), 2.12-2.00 (m, 18 H, 6OAc), 1.92-1.74 (m, 8H, H-12, H-13, H-18, H-19). ^{13}C-NMR ($CDCl_3$, 100 MHz) δ 170.30, 170.24, 169.32, 169.17, 169.15 (6C=O, $OCOCH_3$), 159.56 (C=O, C-4), 150.15 (C=O, C-2), 148.09, 148.02 (2C, C-15, C-21), 145.02, 143.37, 140.03, 133.73, 130.70, 129.76, 129.02, 127.93 (8C, C-Ar), 120.18, 120.00 (2CH, C-16, C-22), 89.86, 89.79 (2CH, C-1′, C-1″), 80.79, 80.73 (2CH, C-4′, C-4″), 74.26, 74.24 (2CH, C-3′, C-3″), 70.86, 70.78 (2CH, C-2′, C-2″), 62.95, 62.91 ($2CH_2$, C-5′, C-5″), 42.31, 42.06 ($2CH_2$, C-11, C-17), 27.15, 27.10, 26.45, 25.15, 25.03 ($6CH_2$, C-12, C-13, C-14, C-18, C-19, C-20), 20.61, 20.59, 20.38, 20.32 ($6CH_3$, $OCOCH_3$). MALDI MS *m*/*z*: calcd for $C_{44}H_{53}N_{10}O_{16}$ 977.9, $C_{44}H_{52}N_{10}NaO_{16}$ 999.9, $C_{44}H_{52}N_{10}KO_{16}$ 1016.0; found 977.8 $[M + H]^+$, 999.8 $[M + Na]^+$, 1016.8 $[M + K]^+$. Elemental Analysis calcd for $C_{44}H_{52}N_{10}O_{16}$, C 54.09, H 5.36, N 14.34%; found C 54.15, H 5.38, N 14.34%.

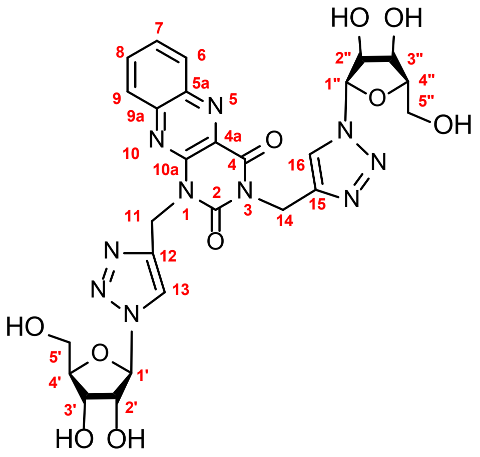

1,3-Bis{[1-(β-D-ribofuranosyl)-1H-1,2,3-triazol-4-yl]methyl}benzo[g]pteridine-2,4(1H,3H)-dione (**5g**)

A yellow foam, 81% yield. ^{1}H-NMR (CD_3OD, 400 MHz) δ 8.32, 8.29 (2s, 2H, H-13, H-16), 8.19-8.15 (m, 1H, H-Ar), 8.08 (dd, 1H, *J* = 8.4, 0.7 Hz, H-Ar), 7.95-7.90 (m, 1H, H-Ar), 7.81-7.76 (m, 1H, H-Ar), 6.00, 5.97 (2d, 2H, *J* = 3.8 Hz, H-1′, H-1″), 5.73 (s, 2H, H-14), 5.43 (s, 2H, H-11), 4.49-4.40 (m, 2H, H-3′, H-3″), 4.31-4.24 (m, 2H, H-2′, H-2″), 4.12-4.04 (m, 2H, H-4′, H-4″), 3.80-3.72 (m, 2H, H-5a′, H-5a″), 3.69-3.60 (m, 2H, H-5b′, H-5b″). ^{13}C-NMR (DMSO-*d*$_6$, 100 MHz) δ 158.76 (C=O, C-4), 149.75 (C=O, C-2), 142.55, 142.50 (2C, C-12, C-15), 144.91, 141.92, 138.99, 133.74, 131.02, 129.87, 129.12, 127.52 (8C, C-Ar), 122.75, 122.37 (2CH, C-13, C-16), 91.90 (2CH, C-1′, C-1″), 85.71, 85.67 (2CH, C-4′, C-4″), 74.84, 74.81 (2CH, C-3′, C-3″), 70.20, 70.18 (2CH, C-2′, C-2″), 61.26, 61.23 ($2CH_2$, C-5′, C-5″), 37.66, 37.23 ($2CH_2$, C-11, C-14). MALDI MS *m*/*z*: calcd for $C_{26}H_{28}N_{10}NaO_{10}$ 663.5; found 663.4 $[M + Na]^+$. Elemental Analysis calcd for $C_{26}H_{28}N_{10}O_{10}$, C 48.75, H 4.41, N 21.87%; found C 48.85, H 4.51, N 21.89%.

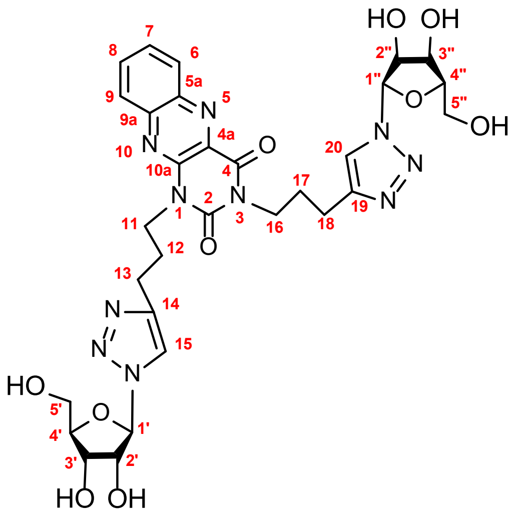

1,3-Bis{3-[1-(β-D-ribofuranosyl)-1H-1,2,3-triazol-4-yl]propyl}benzo[g]pteridine-2,4(1H,3H)-dione (**5h**)

A yellow foam, 79% yield. ^{1}H-NMR (CD_3OD, 400 MHz) δ 8.21 (d, 1H, *J* = 8.5 Hz, H-Ar), 8.06 (s, 1H, H-20), 8.04-8.00 (m, 2H, H-15, H-Ar), 7.97-7.92 (m, 1H, H-Ar), 7.82-7.78 (m, 1H, H-Ar), 5.96, 5.93 (2d, 2H, *J* = 4.0 Hz, H-1′, H-1″), 4.54-4.49 (m, 2H, H-3′, H-3″), 4.47-4.42 (m, 2H, H-2′, H-2″), 4.30-4.26 (m, 2H, H-16), 4.21 (t, 2H, *J* = 6.7 Hz, H-11), 4.12-4.03 (m, 2H, H-4′, H-4″), 3.77 (dd, 2H, *J* = 12.1, 3.0 Hz, H-5a′, H-5a″), 3.66 (dd, 2H, *J* = 12.8, 4.0 Hz, H-5b′, H-5b″), 2.90-2.83 (m, 4H, H-13, H-18), 2.25-2.12 (m, 4H, H-12, H-17). ^{13}C-NMR (DMSO-d_6, 100 MHz) δ 159.72 (C=O, C-4), 150.72 (C=O, C-2), 147.13 (2C, C-14, C-19), 145.88, 142.57, 139.30, 133.90, 131.83 , 130.37, 129.25, 128.01 (8C, C-Ar), 120.77 (2CH, C-15, C-20), 92.43 (2CH, C-1′, C-1″), 86.17 (2CH, C-4′, C-4″), 75.51 (2CH, C-3′, C-3″), 70.86 (2CH, C-2′, C-2″), 61.89 ($2CH_2$, C-5′, C-5″), 42.22, 41.97 ($2CH_2$, C-11, C-16), 27.49, 27.21 ($2CH_2$, C-13, C-18), 23.26, 23.14 ($2CH_2$, C-12, C-17). MALDI MS *m*/*z*: calcd for $C_{30}H_{37}N_{10}O_{10}$ 697.6; found 697.5 $[M + H]^+$. Elemental Analysis calcd for $C_{30}H_{36}N_{10}O_{10}$, C 51.72, H 5.21, N 20.11%; found C 51.81, H 5.28, N 20.15%.

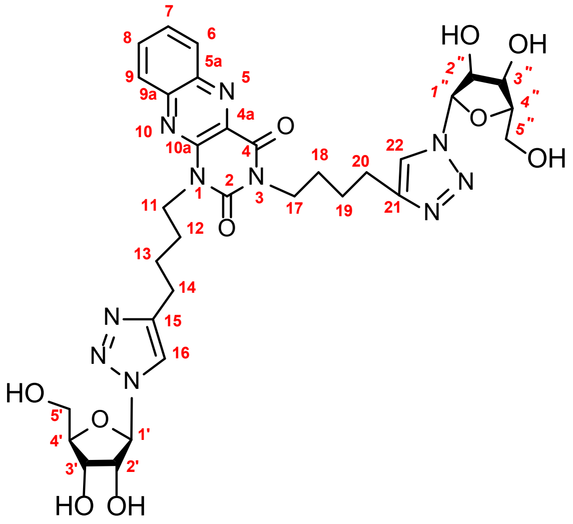

1,3-Bis{4-[1-(β-D-ribofuranosyl)-1H-1,2,3-triazol-4-yl]butyl}benzo[g]pteridine-2,4(1H,3H)-dione (**5i**)

A yellow foam, 85% yield. ^{1}H-NMR (DMSO-d_6, 400 MHz) δ 8.18 (d, 1H, *J* = 8.4 Hz, H-Ar), 8.01, 7.99 (2s, 2H, H-16, H-22), 7.98-7.92 (m, 2H, H-Ar), 7.82-7.77 (m, 1H, H-Ar), 5.87-5.82 (m, 2H, H-1′, H-1″), 4.35-4.27 (m, 2H, H-3′, H-3″), 4.12-4.06 (m, 2H, H-2′, H-2″), 4.02-3.92 (m, 4H, H-17, H-4′, H-4″), 3.61-3.54 (m, 4H, H-11, H-5a′, H-5a″), 3.51-3.43 (m, 2H, H-5b′, H-5b″), 2.72-2.63 (m, 4H, H-14, H-20), 1.80-1.60 (m, 8H, H-12, H-13, H-18, H-19). ^{13}C-

NMR (DMSO-d_6, 100 MHz) δ 159.75 (C=O, C-4), 150.56 (C=O, C-2), 147.58, 147.52 (2C, C-15, C-21), 145.64, 142.65, 139.19, 134.08, 131.27, 130.22, 129.38, 127.89 (8C, C-Ar), 120.97, 120.91 (2CH, C-16, C-22), 92.25 (2CH, C-1′, C-1′′), 85.97 (2CH, C-4′, C-4′′), 75.34 (2CH, C-3′, C-3′′), 70.73 (2CH, C-2′, C-2′′), 61.77 (2CH$_2$, C-5′, C-5′′), 42.17, 41.90 (2CH$_2$, C-11, C-17), 27.14, 26.85, 26.62, 26.41, 24.95 (6CH$_2$, C-12, C-13, C-14, C-18, C-19, C-20). MALDI MS *m/z*: calcd for $C_{32}H_{41}N_{10}O_{10}$ 725.7; found 725.5 [M + H]$^+$. Elemental Analysis calcd for $C_{32}H_{40}N_{10}O_{10}$, C 53.03, H 5.56, N 19.33%; found C 53.14, H 5.61, N 19.34%.

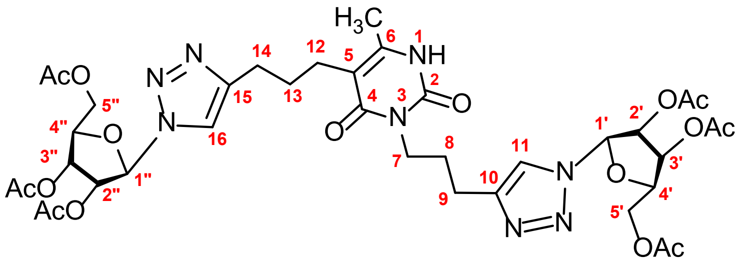

3,5-Bis{3-[1-(2,3,5-tri-O-acetyl-β-D-ribofuranosyl)-1H-1,2,3-triazol-4-yl]propyl}pyrimidine-2,4(1H,3H)-dione (**11c**)

A white foam, 47% yield. ^{1}H-NMR (CDCl$_3$, 400 MHz) δ 10.05 (s, 1H, NH), 7.63, 7.59 (2s, 2H, H-11, H-16), 6.08 (d, 2H, *J* = 3.8 Hz, H-1′, H-1′′), 5.80-5.74 (m, 2H, H-3′, H-3′′), 5.60-5.54 (m, 2H, H-2′, H-2′′), 4.44-4.38 (m, 2H, H-4′, H-4′′), 4.35 (dd, 2H, *J* = 12.4, 4.0 Hz, H-5a′, H-5a′′), 4.17 (dd, 2H, *J* = 12.4, 4.0 Hz, H-5b′, H-5b′′), 3.95 (t, 2H, *J* = 6.8 Hz, H-7), 2.78-2.68 (m, 4H, H-9, H-14), 2.39 (t, 2H, *J* = 7.3 Hz, H-12), 2.29-2.19 (m, 2H, H-8), 2.10 (s, 3H, CH$_3$-6), 2.08-1.97 (m, 18H, 6OAc), 1.84-1.74 (m, 2H, H-13). ^{13}C-NMR (CDCl$_3$, 100 MHz) δ 170.38, 170.33, 169.40, 169.37, 169.19 (6C=O, OCOCH$_3$), 163.67 (C=O, C-4), 152.16 (C=O, C-2), 148.25, 147.78 (2C, C-10, C-15), 145.35 (C, C-6), 120.21, 120.19 (2CH, C-11, C-16), 110.16 (C, C-5), 89.78 (2CH, C-1′, C-1′′), 80.67, 80.60 (2CH, C-4′, C-4′′), 74.23, 74.18 (2CH, C-3′, C-3′′), 70.80, 70.75 (2CH, C-2′, C-2′′), 62.98, 62.91 (2CH$_2$, C-5′, C-5′′), 39.99 (CH$_2$, C-7), 28.00, 26.86, 25.16, 24.80, 23.07 (5CH$_2$, C-8, C-9, C-12, C-13, C-14), 20.62, 20.39, 20.32 (6CH$_3$, OCOCH$_3$), 16.45 (CH$_3$, CH$_3$-6). MALDI MS *m/z*: calcd for $C_{37}H_{49}N_8O_{16}$ 861.8, $C_{37}H_{48}N_8NaO_{16}$ 883.8, $C_{37}H_{48}N_8KO_{16}$ 899.9; found 861.6 [M + H]$^+$, 883.6 [M + Na]$^+$, 899.6 [M + K]$^+$. Elemental Analysis calcd for $C_{37}H_{48}N_8O_{16}$, C 51.63, H 5.62, N 13.02%; found C 51.76, H 5.71, N 13.05%.

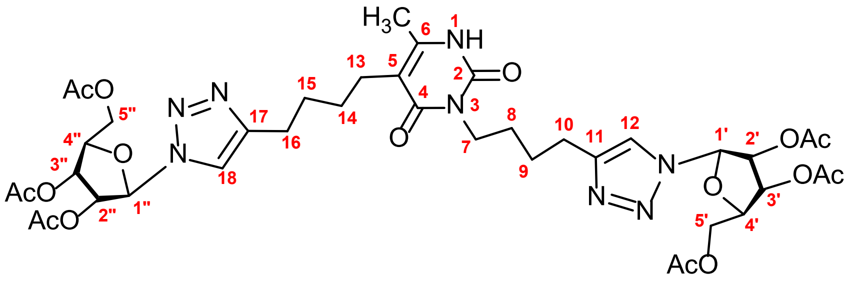

3,5-Bis{4-[1-(2,3,5-tri-O-acetyl-β-D-ribofuranosyl)-1H-1,2,3-triazol-4-yl]butyl}pyrimidine-2,4(1H,3H)-dione (**11d**)

A white foam, 81% yield. ^{1}H-NMR (CDCl$_3$, 400 MHz) δ 9.94 (s, 1H, NH), 7.55, 7.48 (2s, 2H, H-12, H-18), 6.10, 6.08 (2d, 2H, *J* = 3.6 Hz, H-1′, H-1′′), 5.80-5.74 (m, 2H, H-3′, H-3′′), 5.60-5.56 (m, 2H, H-2′, H-2′′), 4.44-4.40 (m, 2H, H-4′, H-4′′), 4.39-4.34 (m, 2H, H-5a′, H-5a′′), 4.22-4.16 (m, 2H, H-5b′, H-5b′′), 3.94-3.88 (m, 2H, H-7), 2.77-2.69 (m, 4H, H-10, H-16), 2.38-2.33 (m, 2H, H-13), 2.12-2.01 (m, 21H, 6OAc, CH$_3$-6), 1.73-1.63 (m, 6H, H-8, H-9, H-15), 1.51-1.44 (m, 2H, H-14). ^{13}C-NMR (CDCl$_3$, 100 MHz) δ 170.40, 170.26, 169.38, 169.34, 169.18 (6C=O, OCOCH$_3$), 163.58 (C=O, C-4), 152.16 (C=O, C-2), 148.34, 148.12 (2C, C-10, C-15), 144.91 (C, C-6), 120.10, 120.00 (2CH, C-12, C-18), 110.54 (C, C-5), 89.79, 89.75 (2CH, C-1′, C-1′′), 80.61, 80.52 (2CH, C-4′, C-4′′), 74.21, 74.17 (2CH, C-3′, C-3′′), 70.70 (2CH, C-2′, C-2′′), 62.92, 62.87 (2CH$_2$, C-5′, C-5′′), 39.99 (CH$_2$, C-7), 28.95, 28.24, 26.87, 26.34, 25.17, 24.88

(7CH$_2$, C-8, C-9, C-10, C-13, C-14, C-15, C-16), 20.58, 20.55, 20.35, 20.28 (6CH$_3$, OCOCH$_3$), 16.39 (CH$_3$, CH$_3$-6). MALDI MS *m/z*: calcd for $C_{39}H_{53}N_8O_{16}$ 889.9, $C_{39}H_{52}N_8NaO_{16}$ 911.8, $C_{39}H_{52}N_8KO_{16}$ 927.9; found 889.9 [M + H]$^+$, 911.9 [M + Na]$^+$, 927.8 [M+K]$^+$. Elemental Analysis calcd for $C_{39}H_{52}N_8O_{16}$, C 52.70, H 5.90, N 12.61%; found C 52.79, H 5.95, N 12.64%.

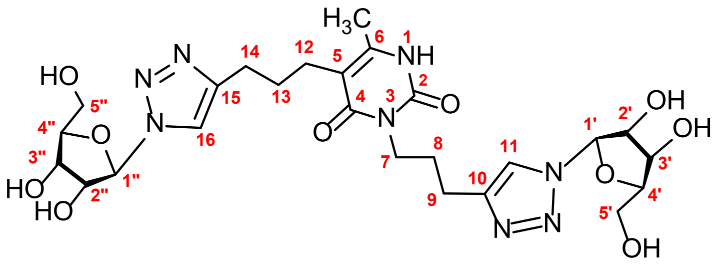

3,5-Bis{3-[1-(β-D-ribofuranosyl)-1H-1,2,3-triazol-4-yl]propyl}pyrimidine-2,4(1H,3H)-dione (**11e**)

A white foam, 71% yield. ^{1}H-NMR (CD$_3$OD, 400 MHz) δ 8.08, 8.07 (2s, 2H, H-11, H-16), 6.00, 5.99 (2d, 2H, J = 3.7 Hz, H-1′, H-1″), 4.50-4.46 (m, 2H, H-3′, H-3″), 4.34-4.27 (m, 2H, H-2′, H-2″), 4.15-4.09 (m, 2H, H-4′, H-4″), 3.94 (t, 2H, J = 7.2 Hz, H-7), 3.81 (dd, 2H, J = 12.3, 3.1 Hz, H-5a′, H-5a″), 3.69 (dd, 2H, J = 12.1, 4.4 Hz, H-5b′, H-5b″), 2.79-2.69 (m, 4H, H-9, H-14), 2.41 (t, 2H, J = 7.7 Hz, H-12), 2.10 (s, 3H, CH$_3$-6), 2.03-1.94 (m, 2H, H-8), 1.85-1.76 (m, 2H, H-13). ^{13}C-NMR (CD$_3$OD, 100 MHz) δ 165.82 (C=O, C-4), 152.89 (C=O, C-2), 149.00 (C, C-6), 148.50 (2C, C-10, C-15), 122.17 (2CH, C-11, C-16), 110.62 (C, C-5), 94.49 (2CH, C-1′, C-1″), 87.21 (2CH, C-4′, C-4″), 77.08 (2CH, C-3′, C-3″), 71.98 (2CH, C-2′, C-2″), 62.97 (2CH$_2$, C-5′, C-5″), 41.11 (CH$_2$, C-7), 29.29, 28.13, 25.91, 25.59, 23.87 (5CH$_2$, C-8, C-9, C-12, C-13, C-14), 16.29 (CH$_3$, CH$_3$-6). MALDI MS *m/z*: calcd for $C_{25}H_{37}N_8O_{10}$ 609.6, $C_{25}H_{36}N_8NaO_{10}$ 631.6; found 609.4 [M + H]$^+$, 631.4 [M + Na]$^+$. Elemental Analysis calcd for $C_{25}H_{36}N_8O_{10}$, C 49.34, H 5.96, N 18.41%; found C 49.41, H 6.01, N 18.44%.

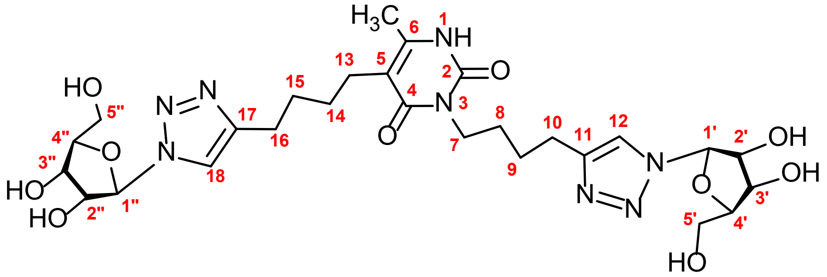

3,5-Bis{4-[1-(β-D-ribofuranosyl)-1H-1,2,3-triazol-4-yl]butyl}pyrimidine-2,4(1H,3H)-dione (**11f**)

A white foam, 89% yield. ^{1}H-NMR (DMSO-d_6, 400 MHz) δ 8.04 (s, 2H, H-12, H-18), 5.92-5.86 (m, 2H, H-1′, H-1″), 4.38-4.31 (m, 2H, H-3′, H-3″), 4.15-4.09 (m, 2H, H-2′, H-2″), 3.99-3.92 (m, 2H, H-4′, H-4″), 3.82-3.74 (m, 2H, H-7), 3.64-3.57 (m, 2H, H-5a′, H-5a″), 3.54-3.47 (m, 2H, H-5b′, H-5b″), 2.69-2.59 (m, 4H, H-10, H-16), 2.33-2.24 (m, 2H, H-13), 2.05 (s, 3H, CH$_3$-6), 1.65-1.51 (m, 6H, H-8, H-9, H-15), 1.43-1.35 (m, 2H, H-14). ^{13}C-NMR (DMSO-d_6, 100 MHz) δ 163.05 (C=O, C-4), 150.67 (C=O, C-2), 147.04, 146.85 (2C, C-10, C-15), 146.24 (C, C-6), 120.26, 120.17 (2CH, C-12, C-18), 108.30 (C, C-5), 91.80 (2CH, C-1′, C-1″), 85.58, 85.57 (2CH, C-4′, C-4″), 74.89, 74.87 (2CH, C-3′, C-3″), 70.29 (2CH, C-2′, C-2″), 61.33, 61.32 (2CH$_2$, C-5′, C-5″), 39.21 (CH$_2$, C-7), 28.64, 27.96, 26.85, 26.33, 24.72, 24.61, 24.25 (7CH$_2$, C-8, C-9, C-10, C-13, C-14, C-15, C-16), 15.72 (CH$_3$, CH$_3$-6). MALDI MS *m/z*: calcd for $C_{27}H_{41}N_8O_{10}$ 637.6, $C_{27}H_{40}N_8NaO_{10}$ 659.6; found 637.5 [M + H]$^+$, 659.5 [M + Na]$^+$. Elemental Analysis calcd for $C_{27}H_{40}N_8O_{10}$, C 50.94, H 6.33, N 17.60%; found C 51.01, H 6.38, N 17.63%.

7. NMR spectra

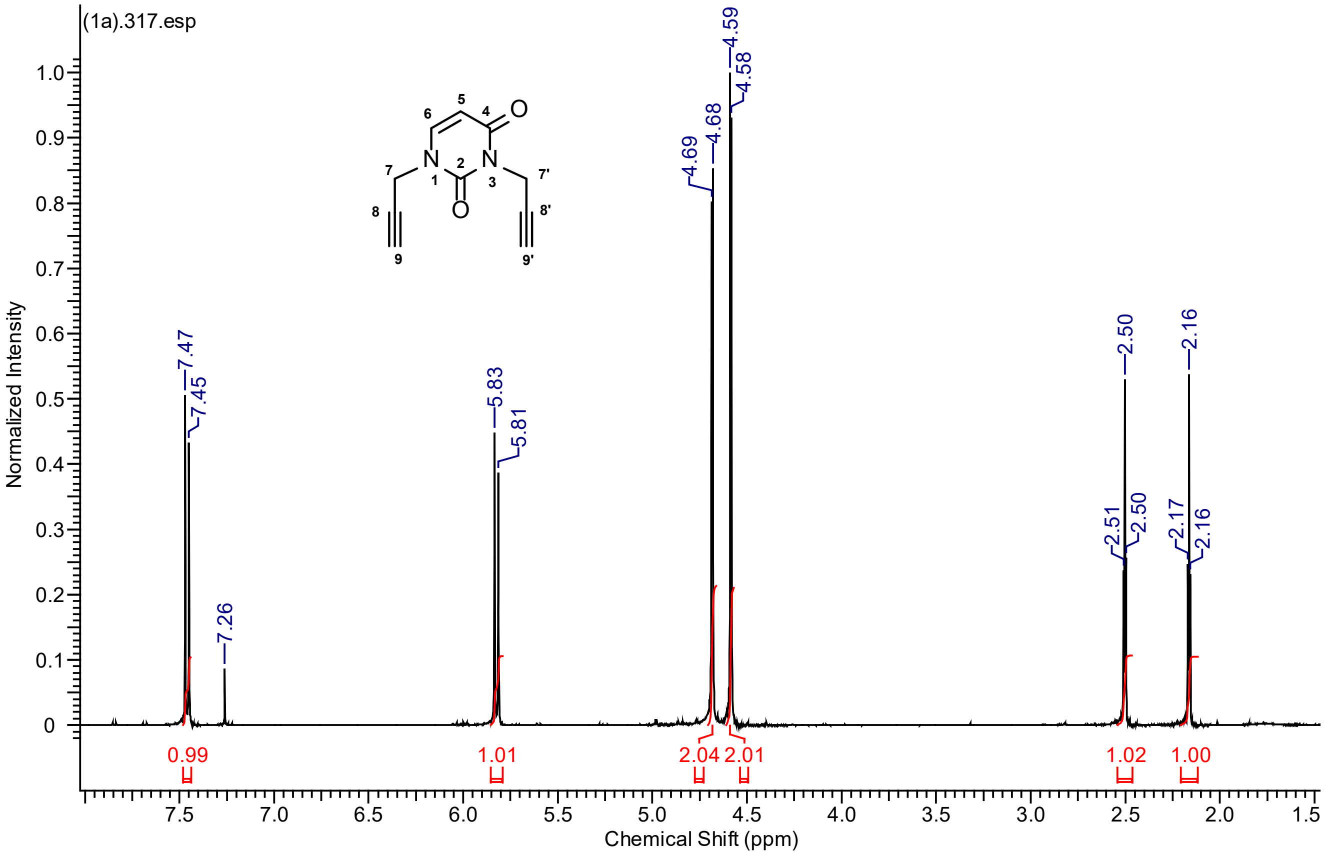

Figure S1. 1D ^{1}H-NMR spectrum of **1a** in $CDCl_3$ at T = 303K.

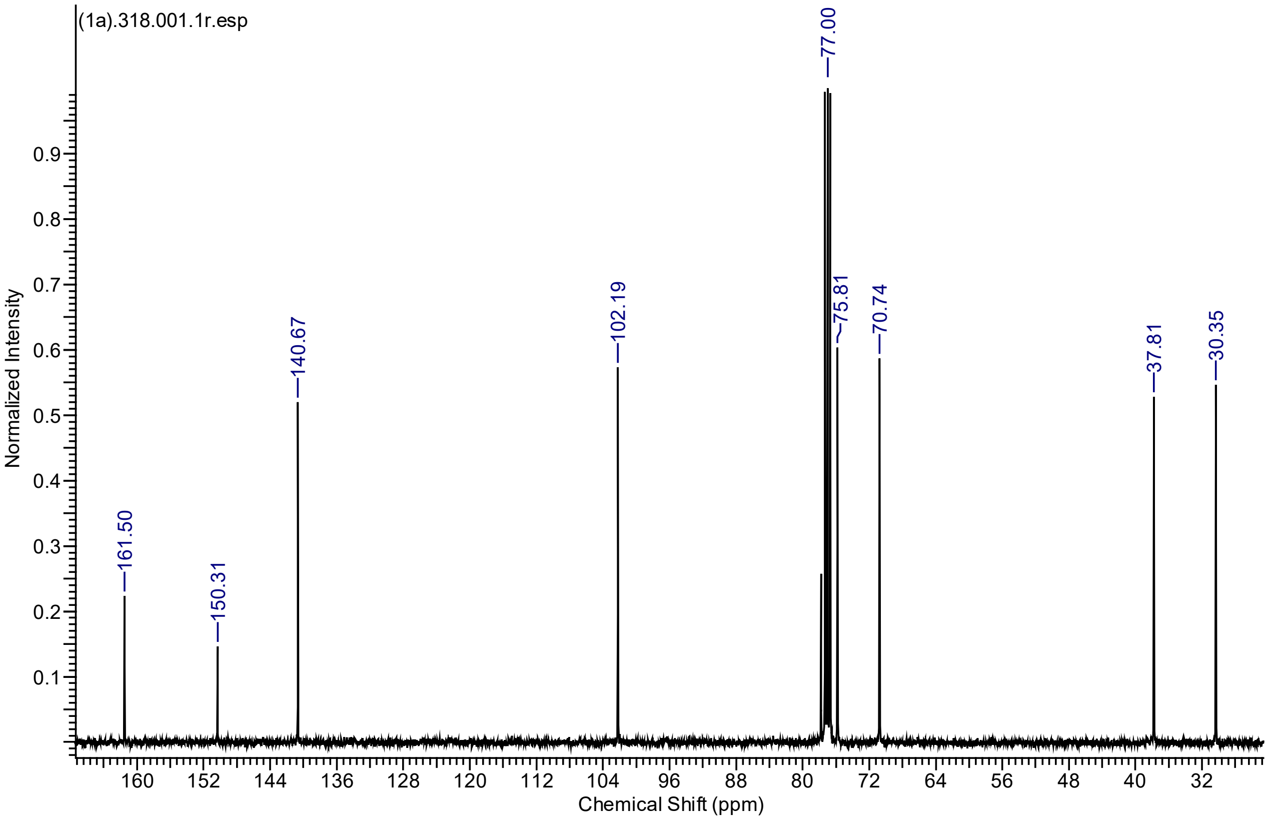

Figure S2. 1D ^{13}C-NMR spectrum of **1a** in $CDCl_3$ at T = 303K.

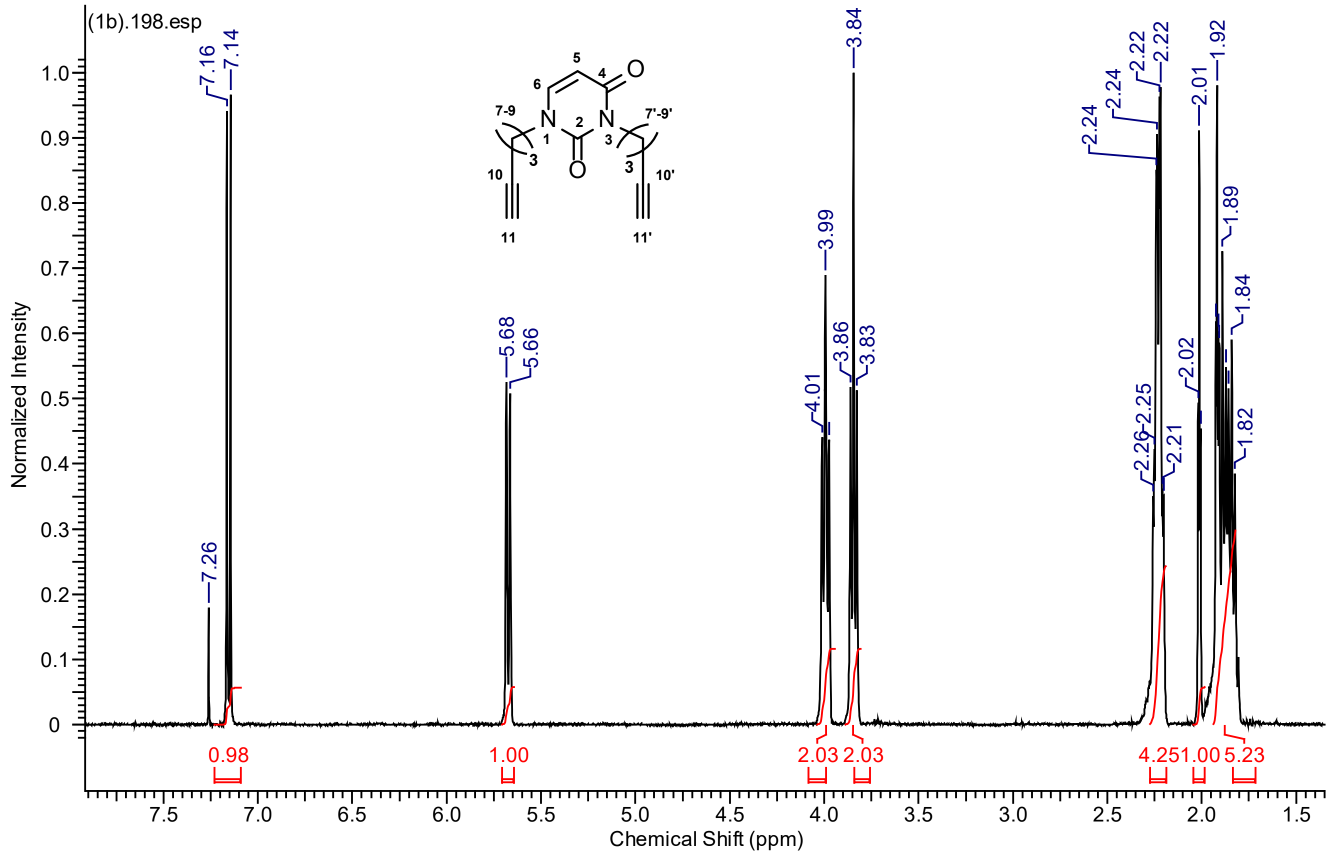

Figure S3. 1D ^{1}H-NMR spectrum of **1b** in $CDCl_3$ at T = 303K.

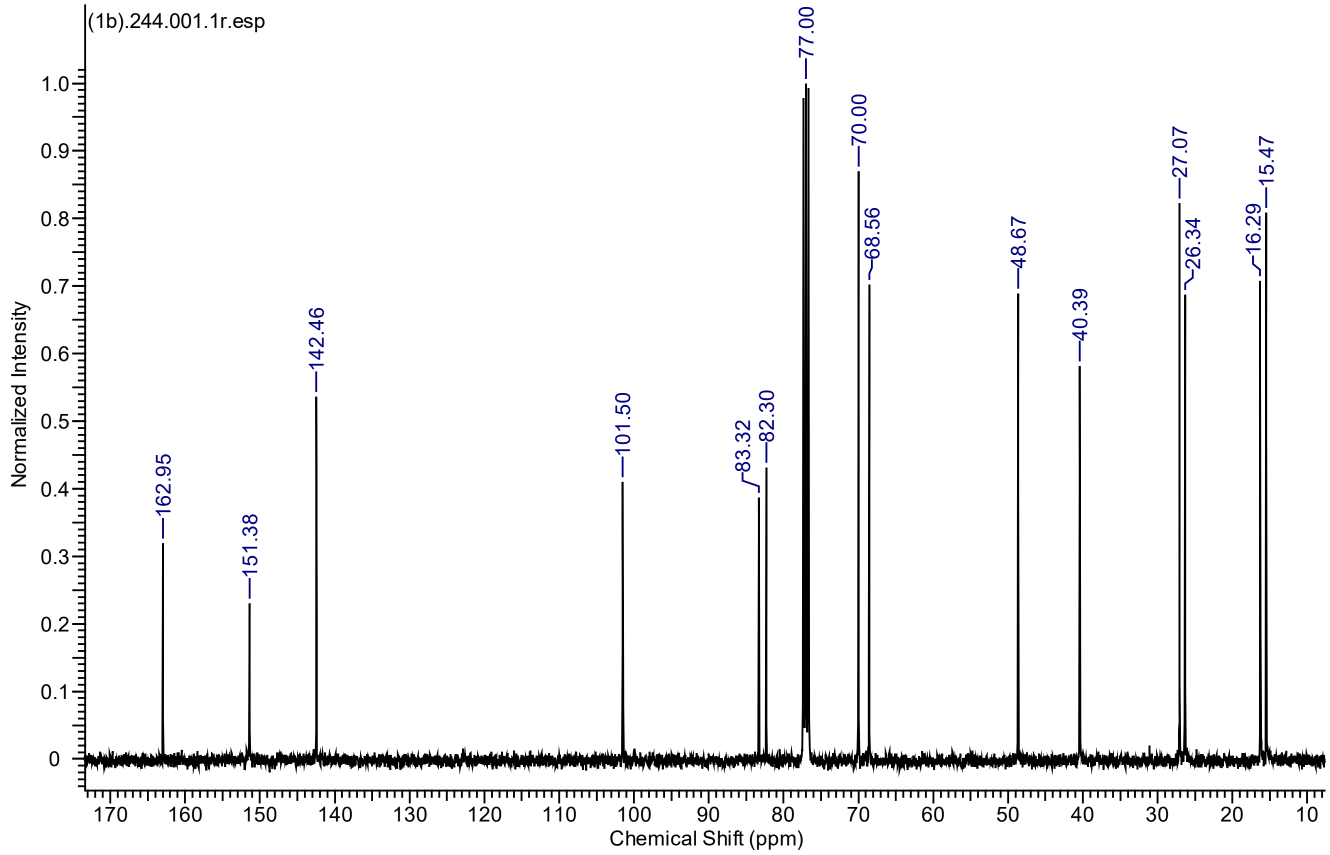

Figure S4. 1D ^{13}C-NMR spectrum of **1b** in $CDCl_3$ at T = 303K.

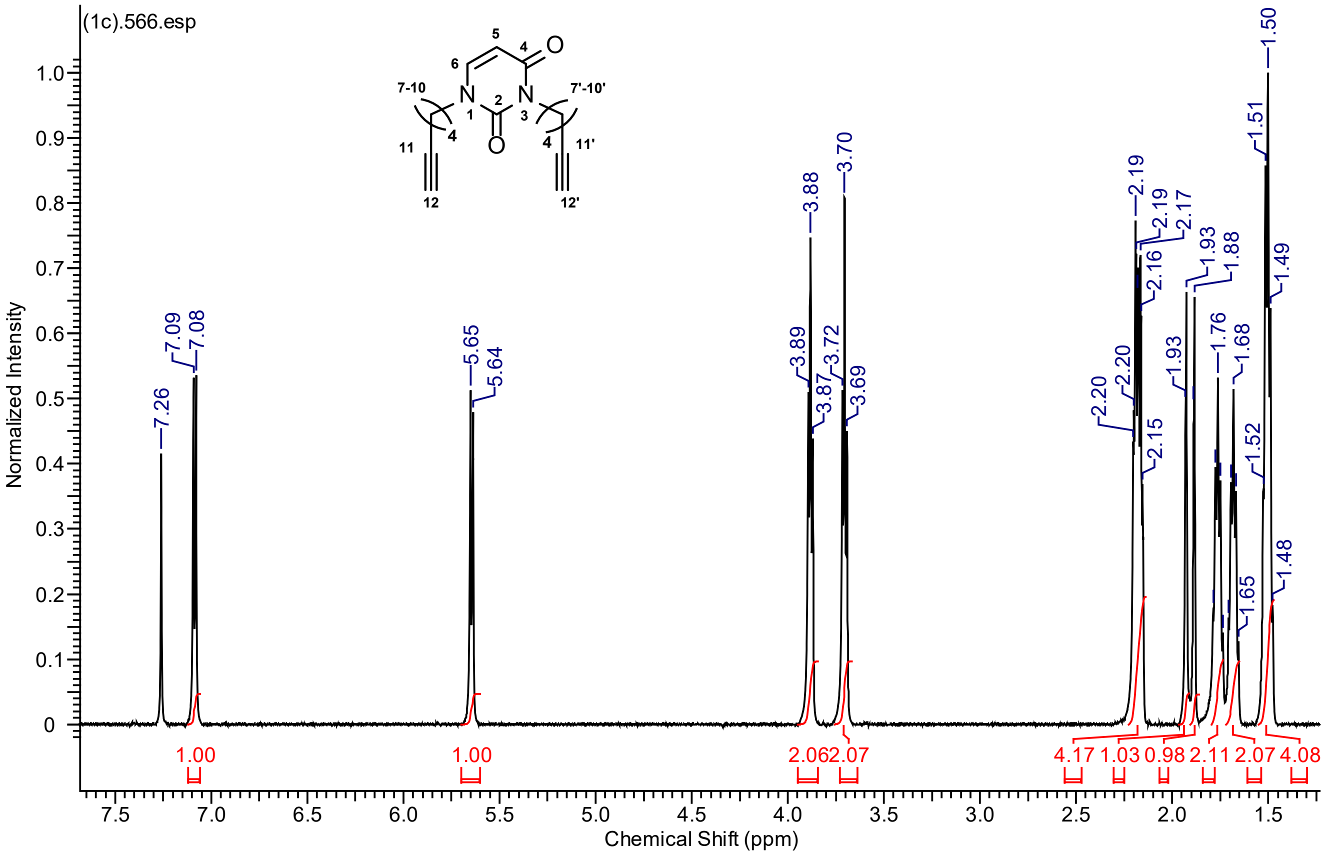

Figure S5. 1D ¹H-NMR spectrum of **1c** in CDCl3 at T = 303K.

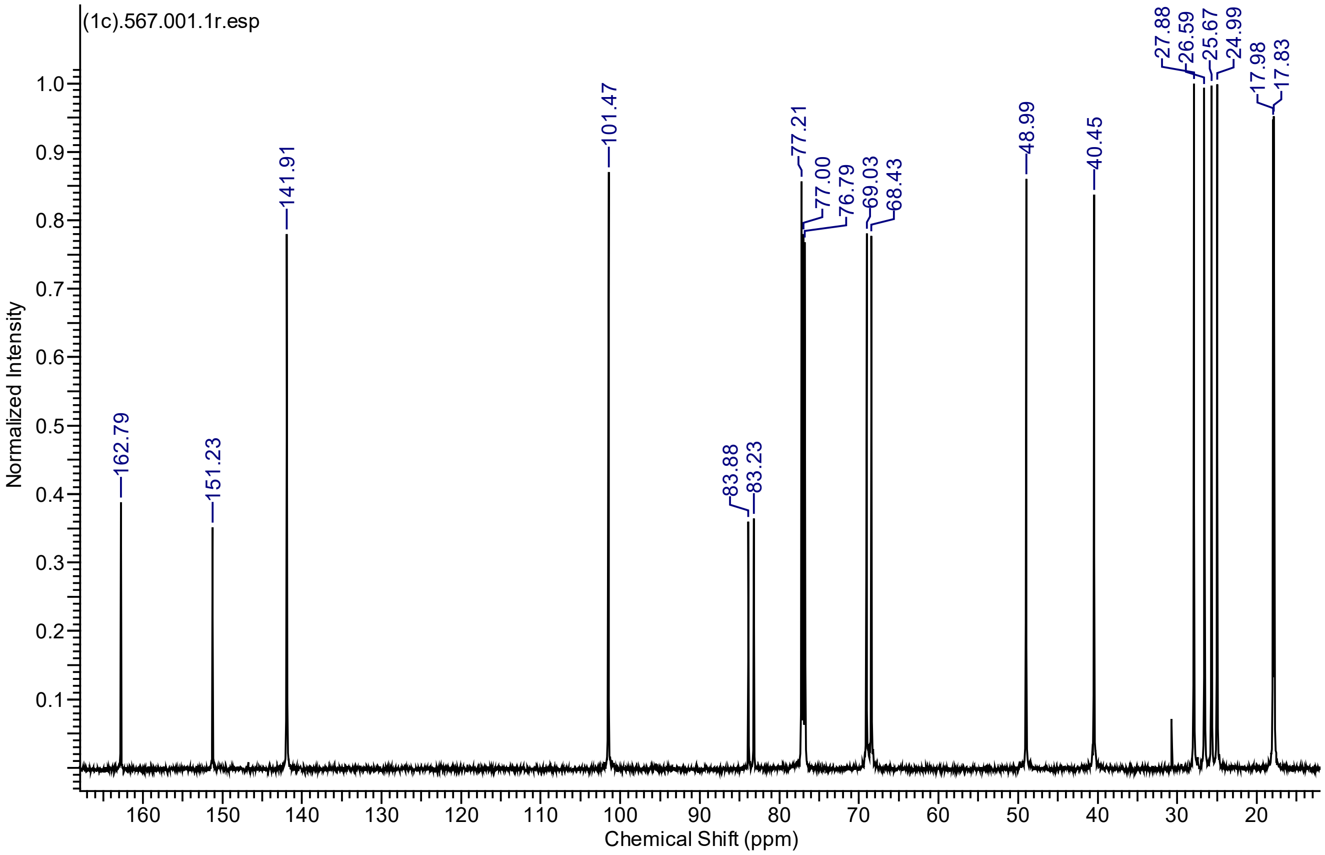

Figure S6. 1D ¹³C-NMR spectrum of **1c** in CDCl3 at T = 303 K.

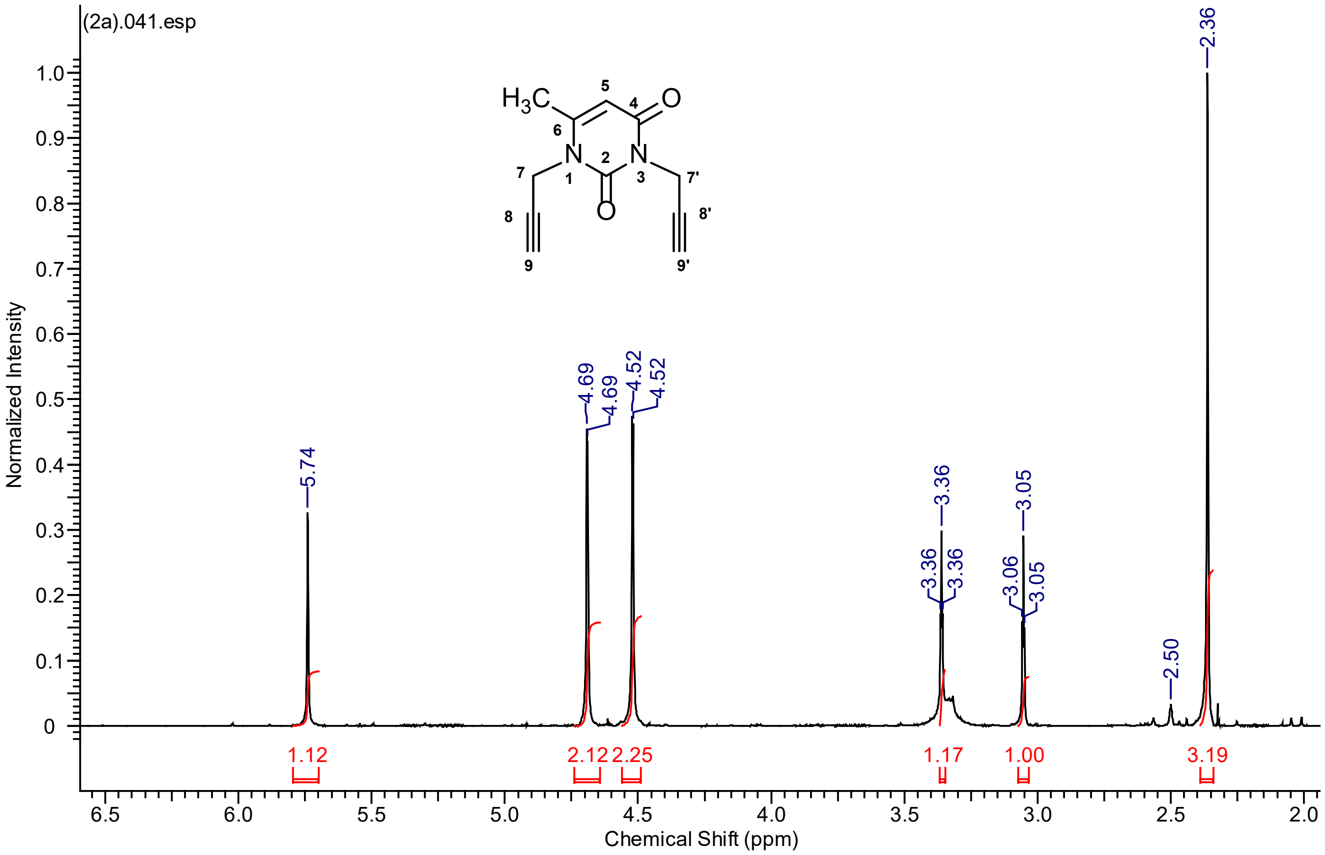

Figure S7. 1D ^{1}H-NMR spectrum of **2a** in $CDCl_3$ at T = 303K.

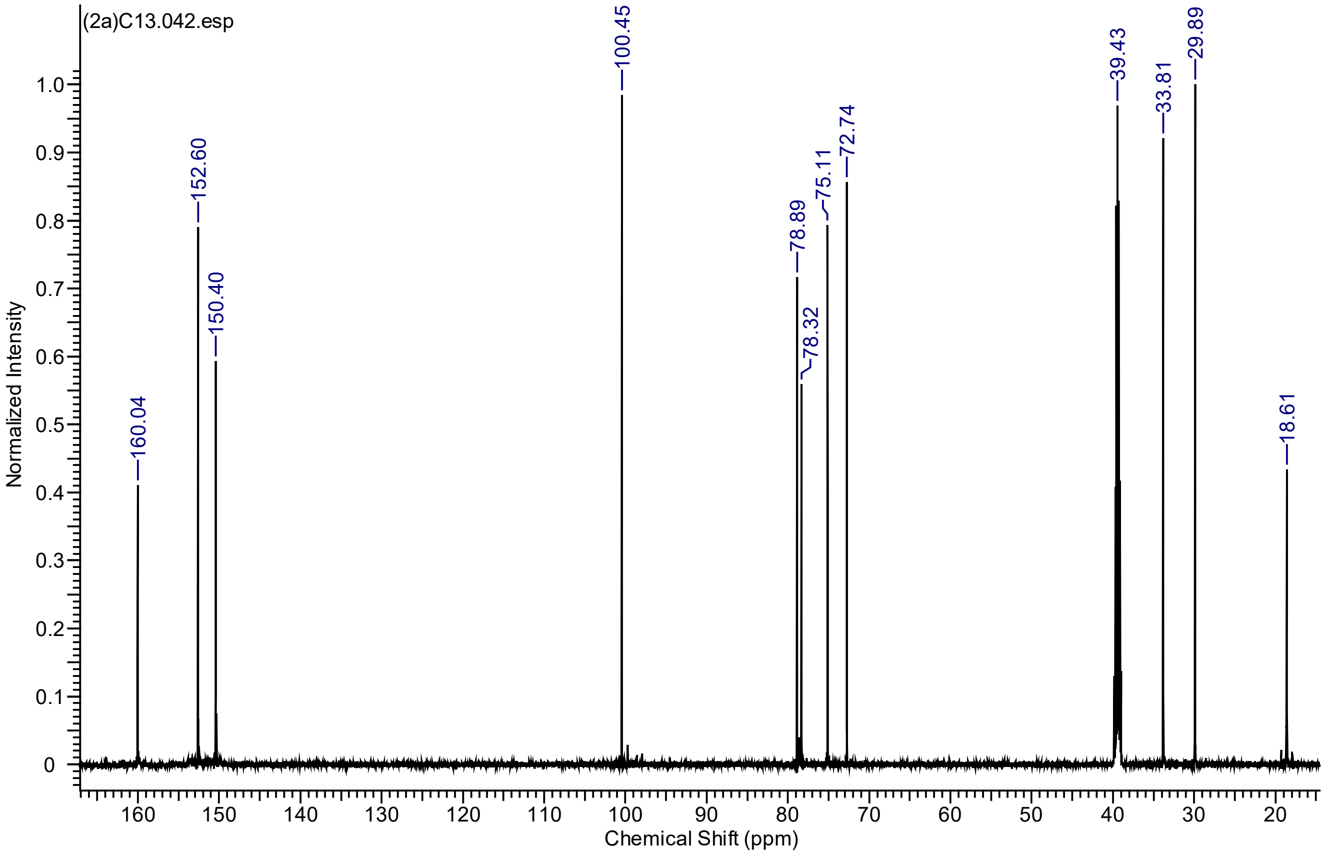

Figure S8. 1D ^{13}C-NMR spectrum of **2a** in DMSO-d_6 at T = 303K.

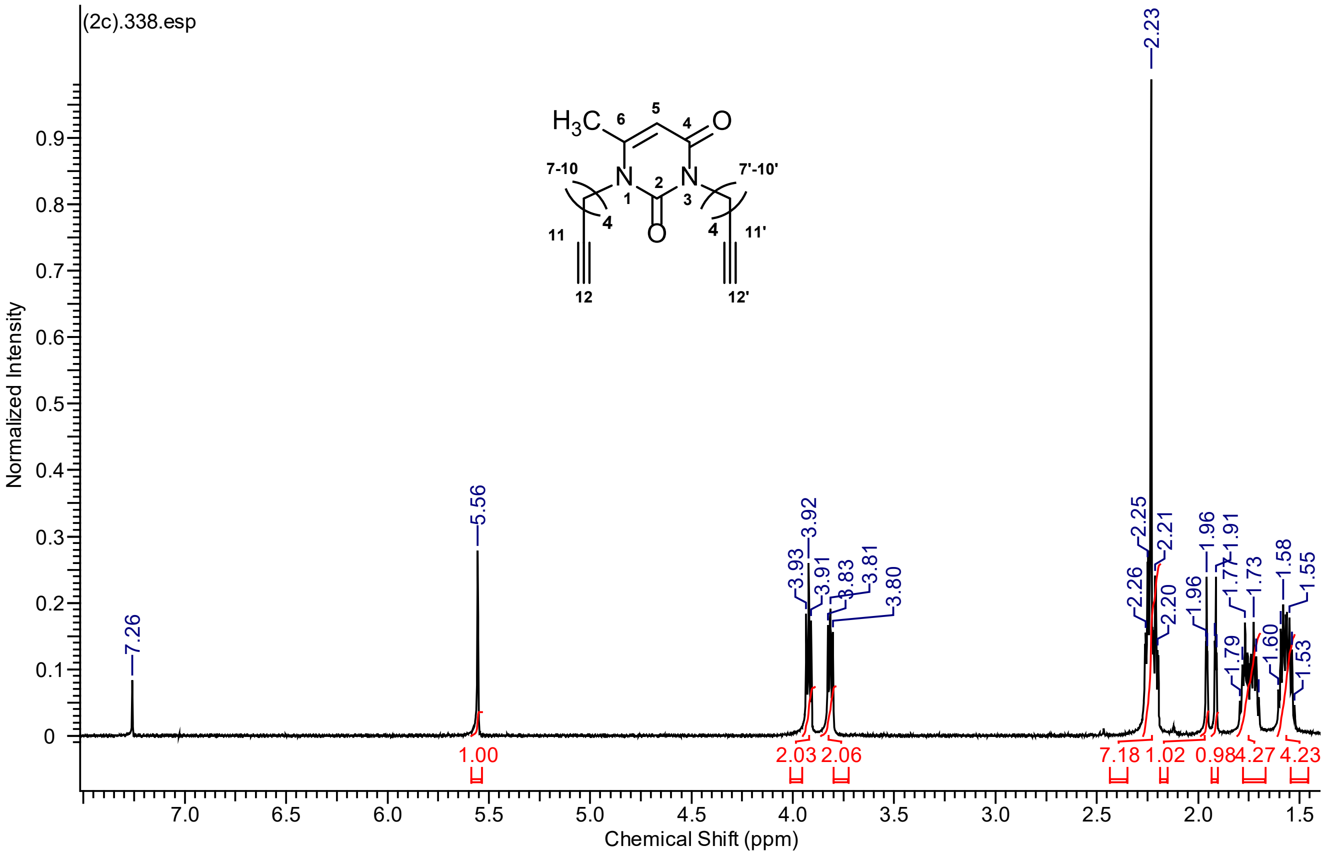

Figure S9. 1D ^{1}H-NMR spectrum of **2c** in $CDCl_3$ at T = 303K.

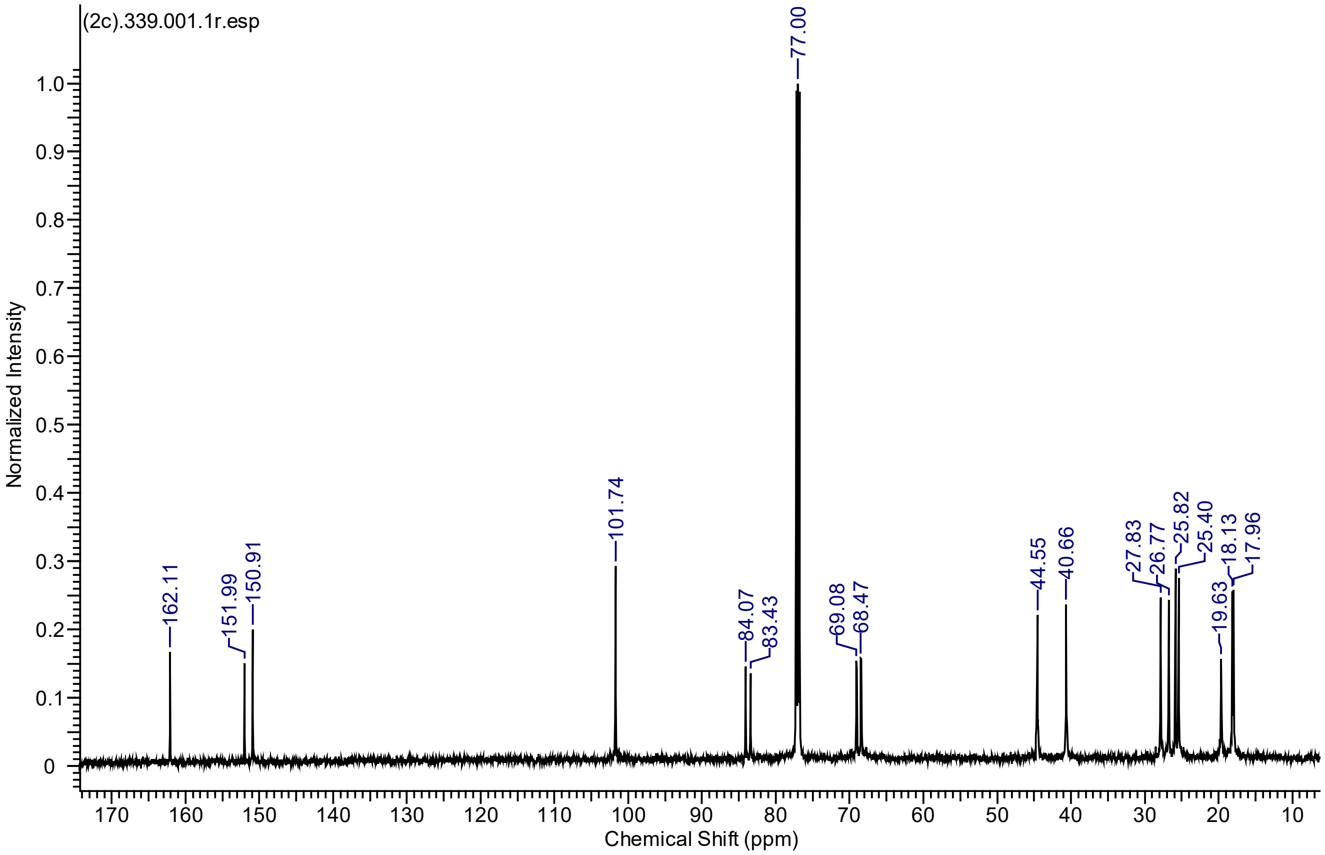

Figure S10. 1D ^{13}C-NMR spectrum of **2c** in $CDCl_3$ at T = 303K.

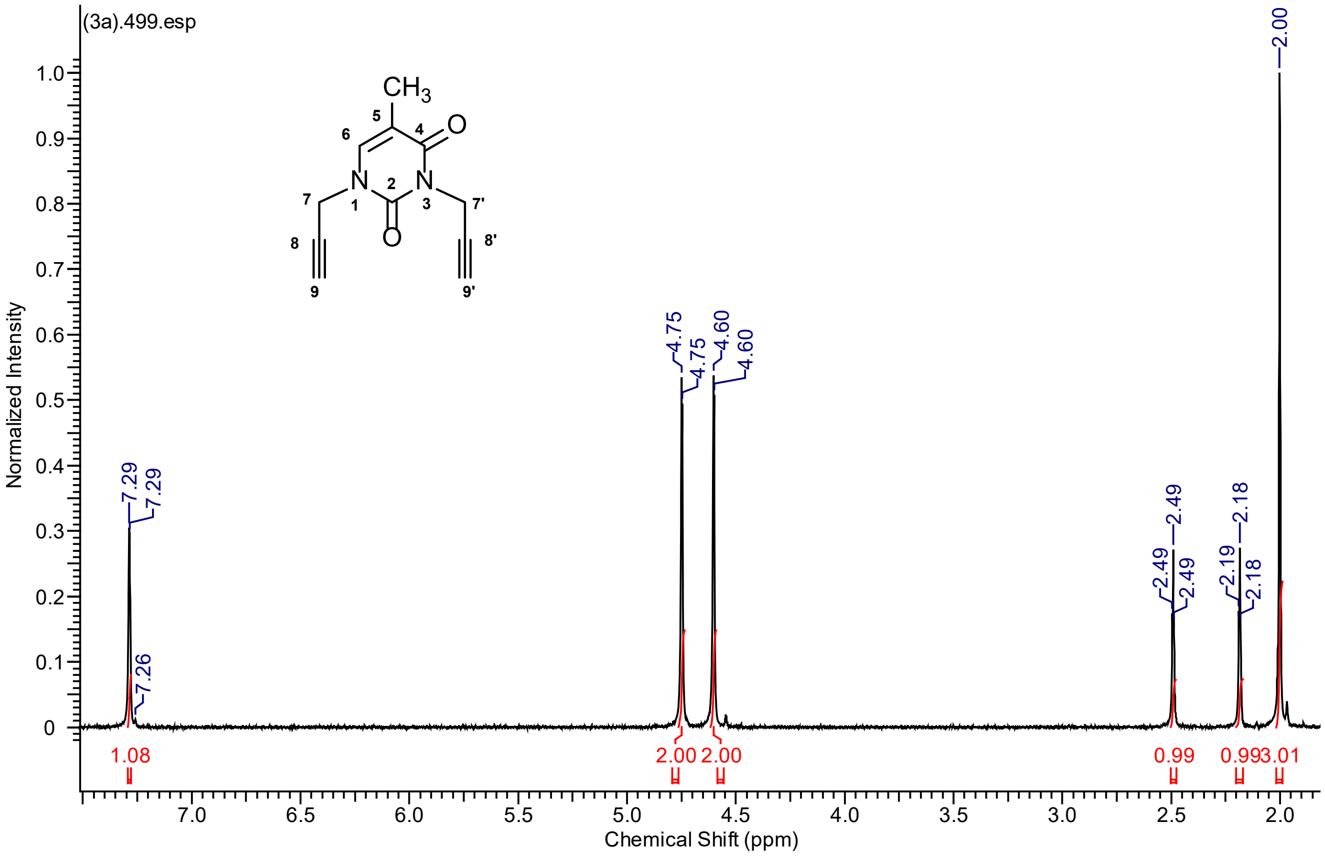

Figure S11. 1D ^{1}H-NMR spectrum of **3a** in $CDCl_3$ at T = 303K.

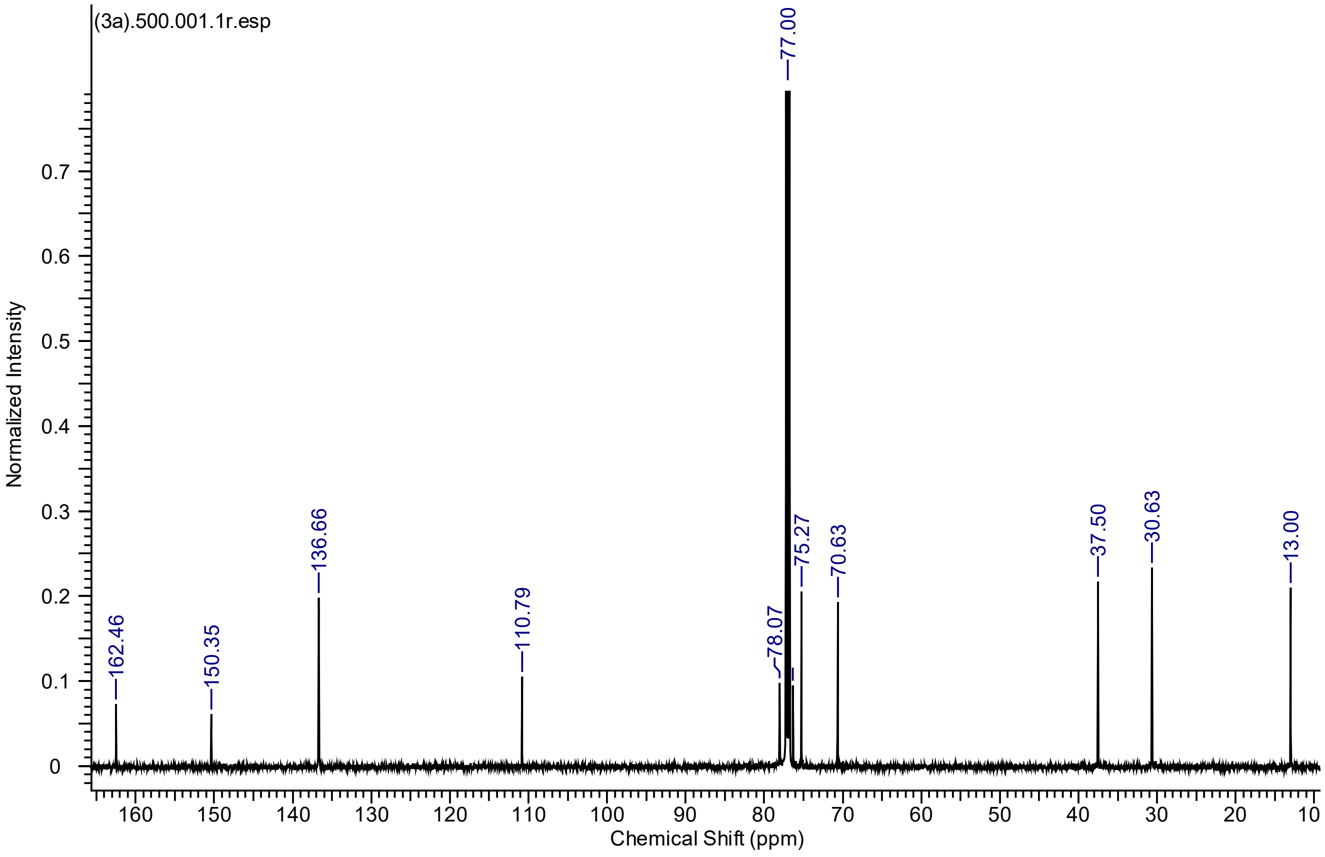

Figure S12. 1D ^{13}C-NMR spectrum of **3a** in $CDCl_3$ at T = 303K.

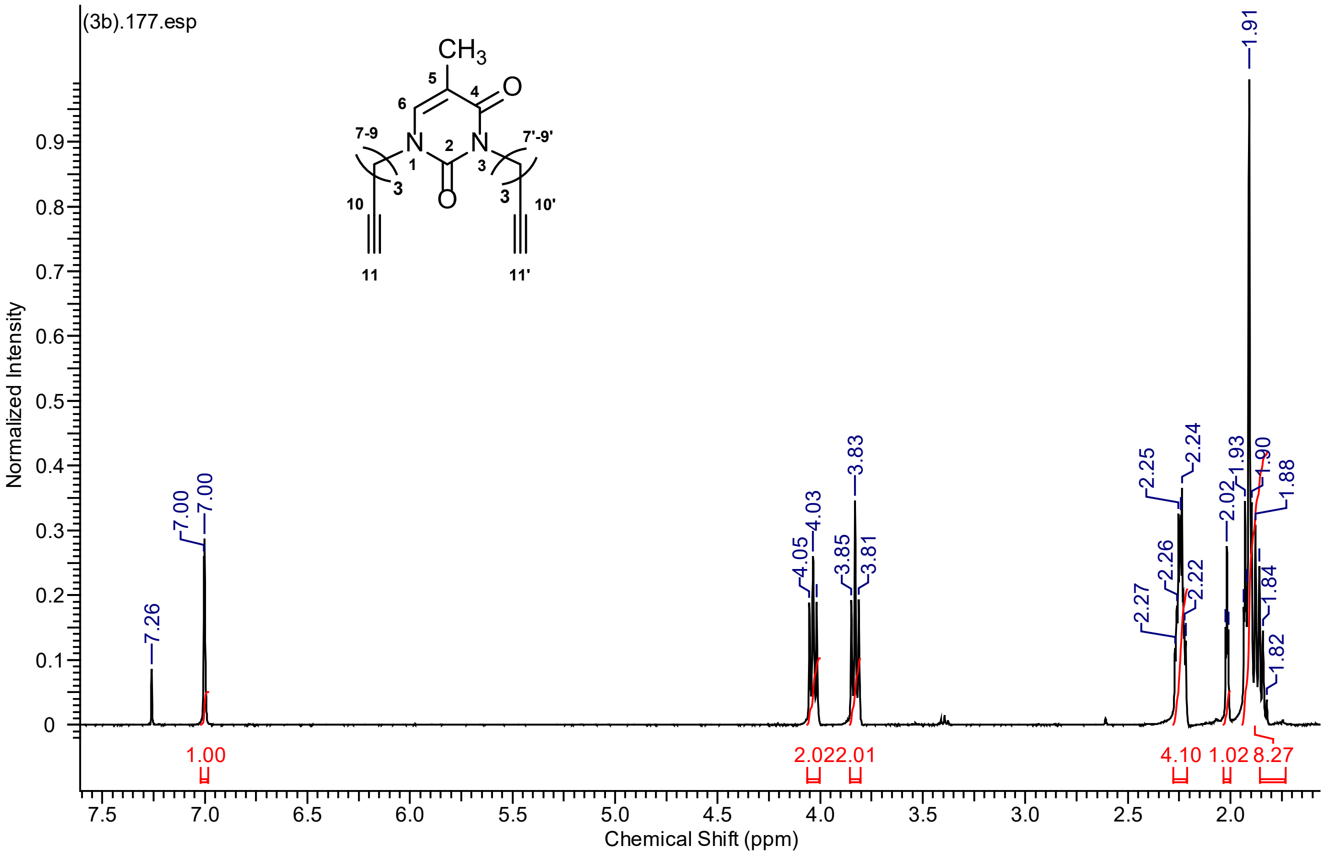

Figure S13. 1D ^{1}H-NMR spectrum of **3b** in $CDCl_3$ at T = 303.

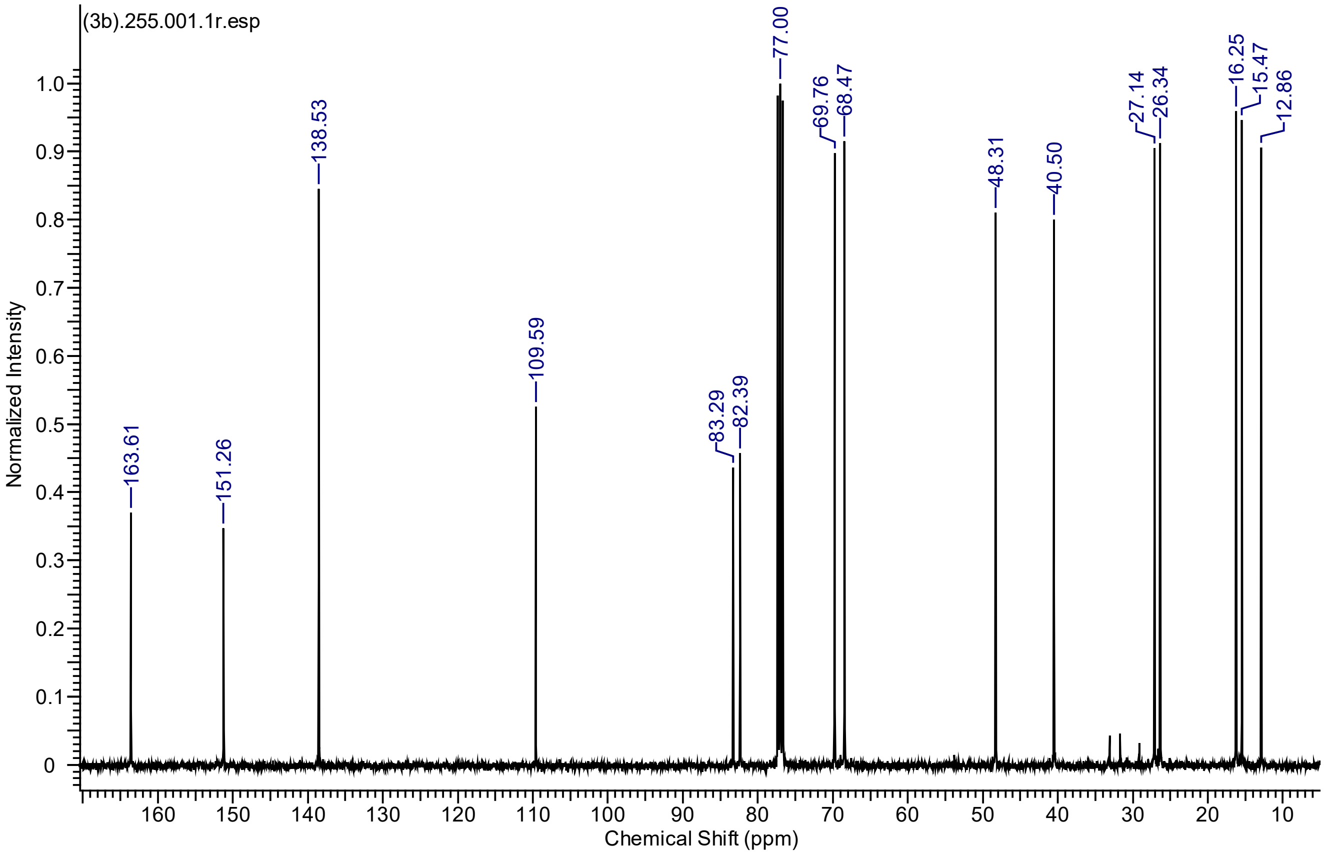

Figure S14. 1D ^{13}C-NMR spectrum of **3b** in $CDCl_3$ at T = 303K.

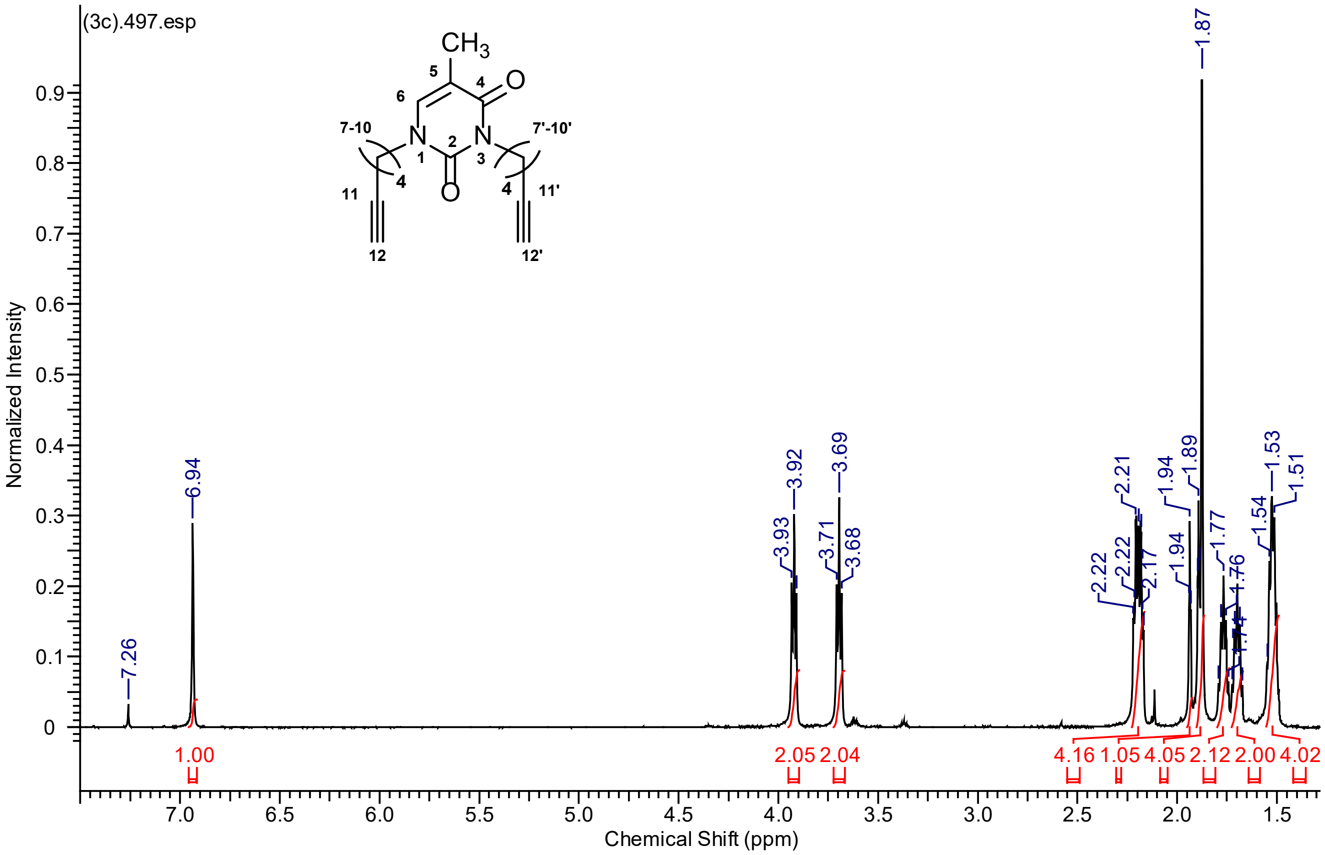

Figure S15. 1D ^{1}H-NMR spectrum of **3c** in $CDCl_3$ at T = 303K.

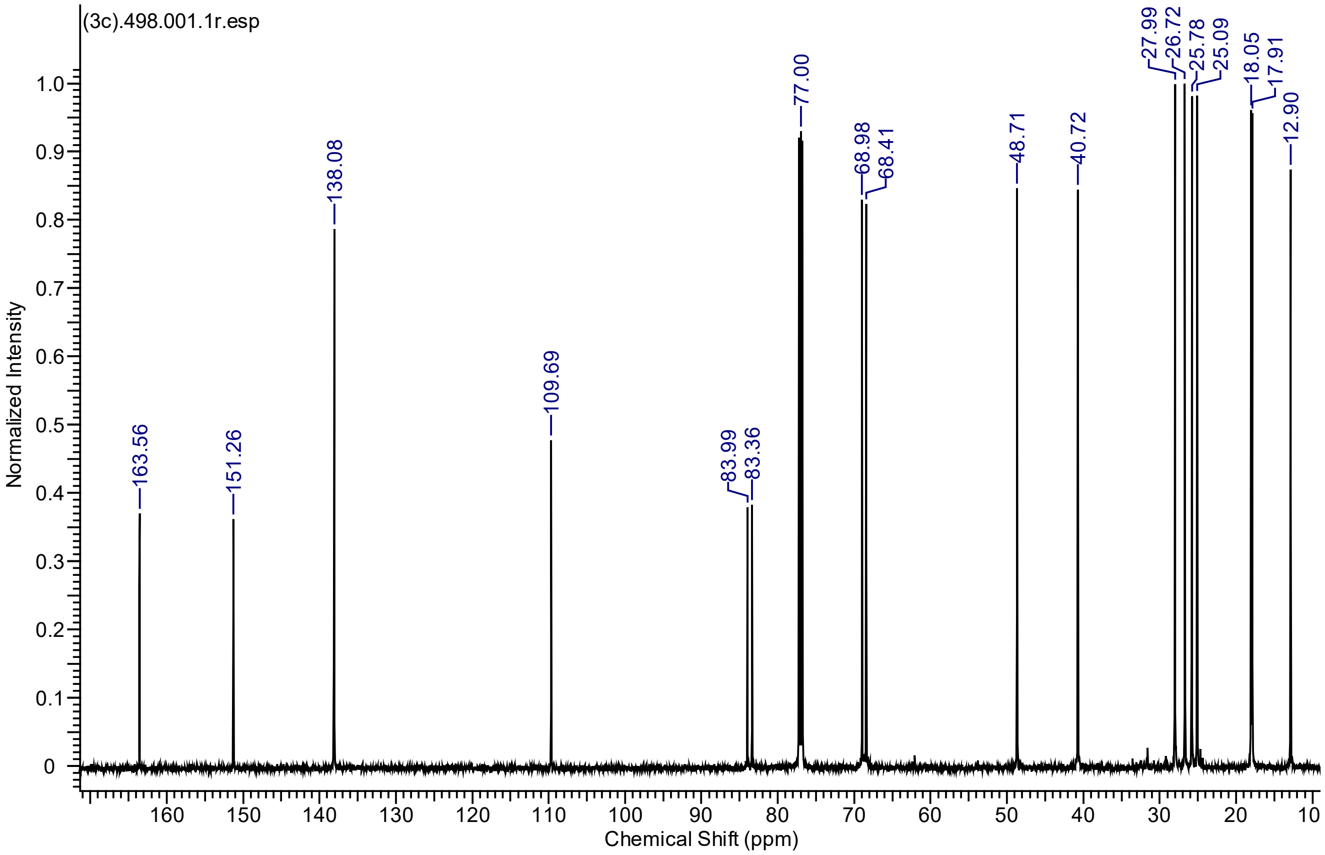

Figure S16. 1D ^{13}C-NMR spectrum of **3c** in $CDCl_3$ at T = 303K.

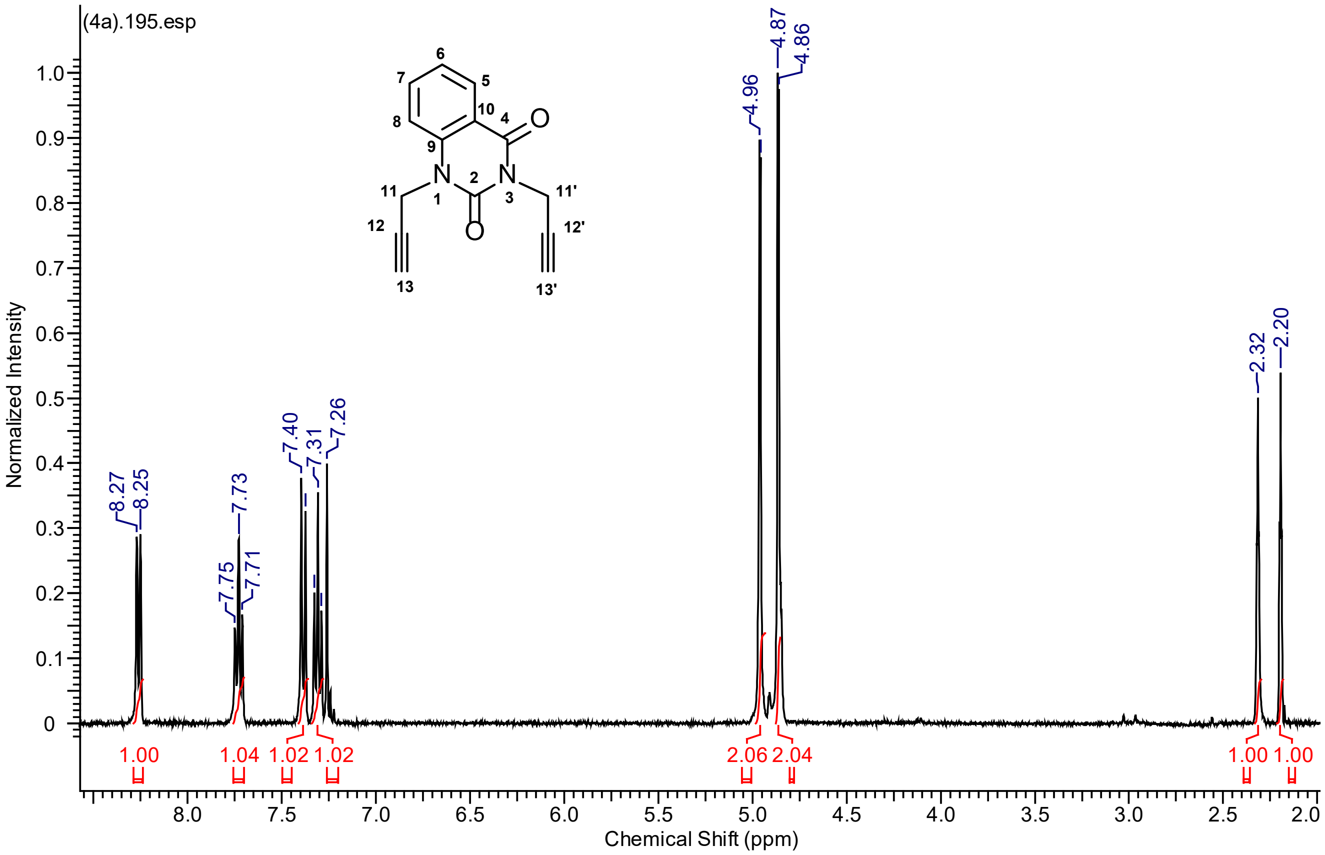

Figure S17. 1D ^{1}H-NMR spectrum of **4a** in $CDCl_3$ at T = 303K.

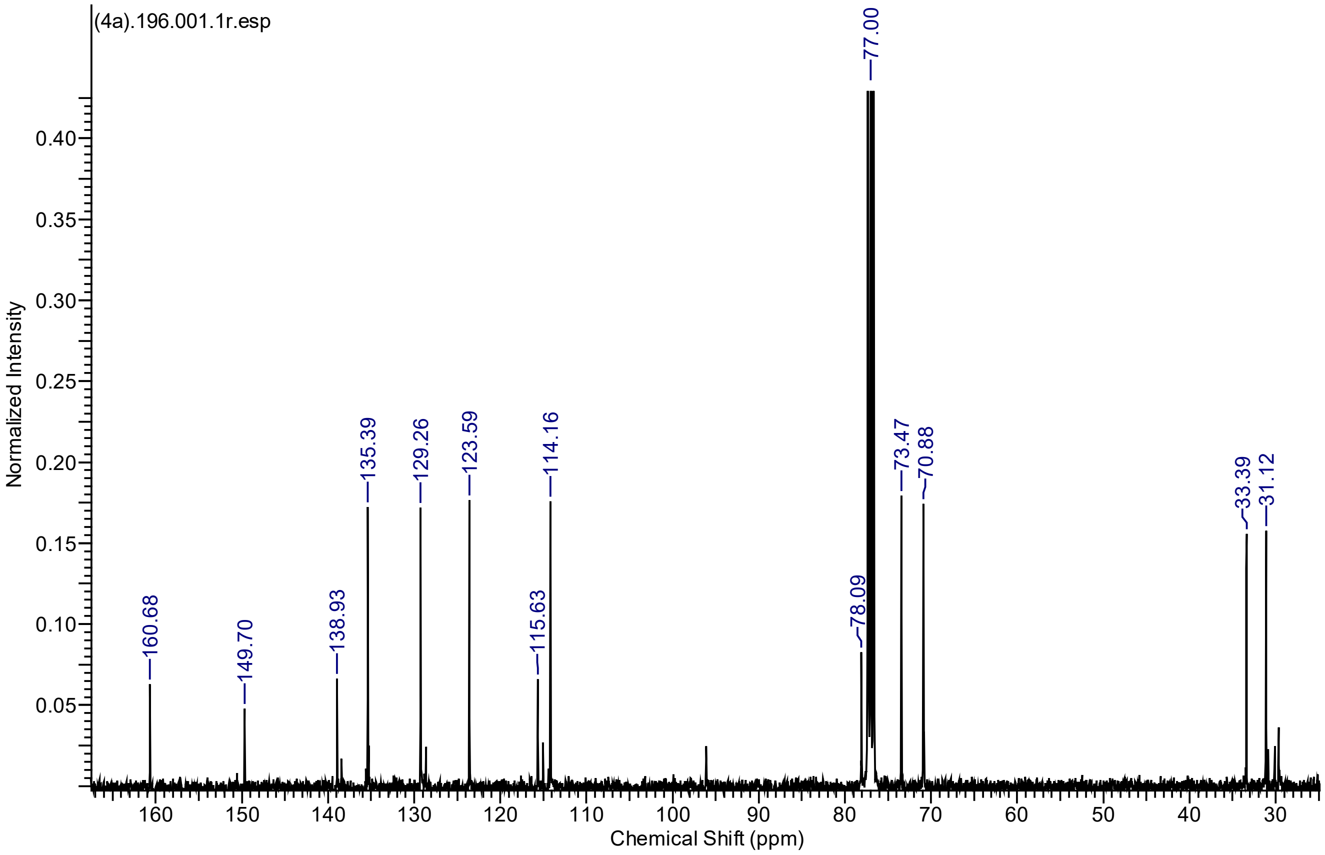

Figure S18. 1D ^{13}C-NMR spectrum of **4a** in $CDCl_3$ at T = 303K.

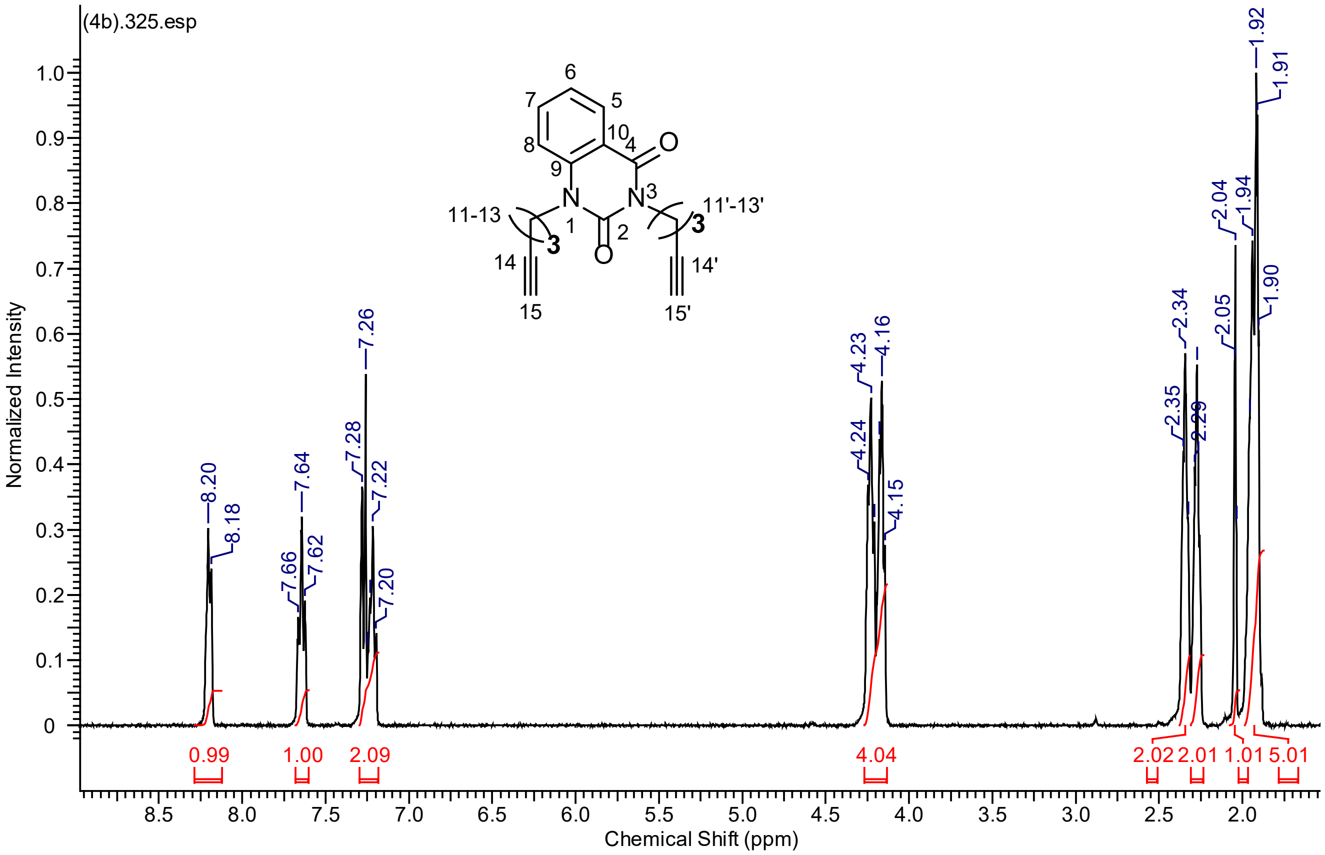

Figure S19. 1D ^{1}H NMR spectrum of **4b** in $CDCl_3$ at T = 303K.

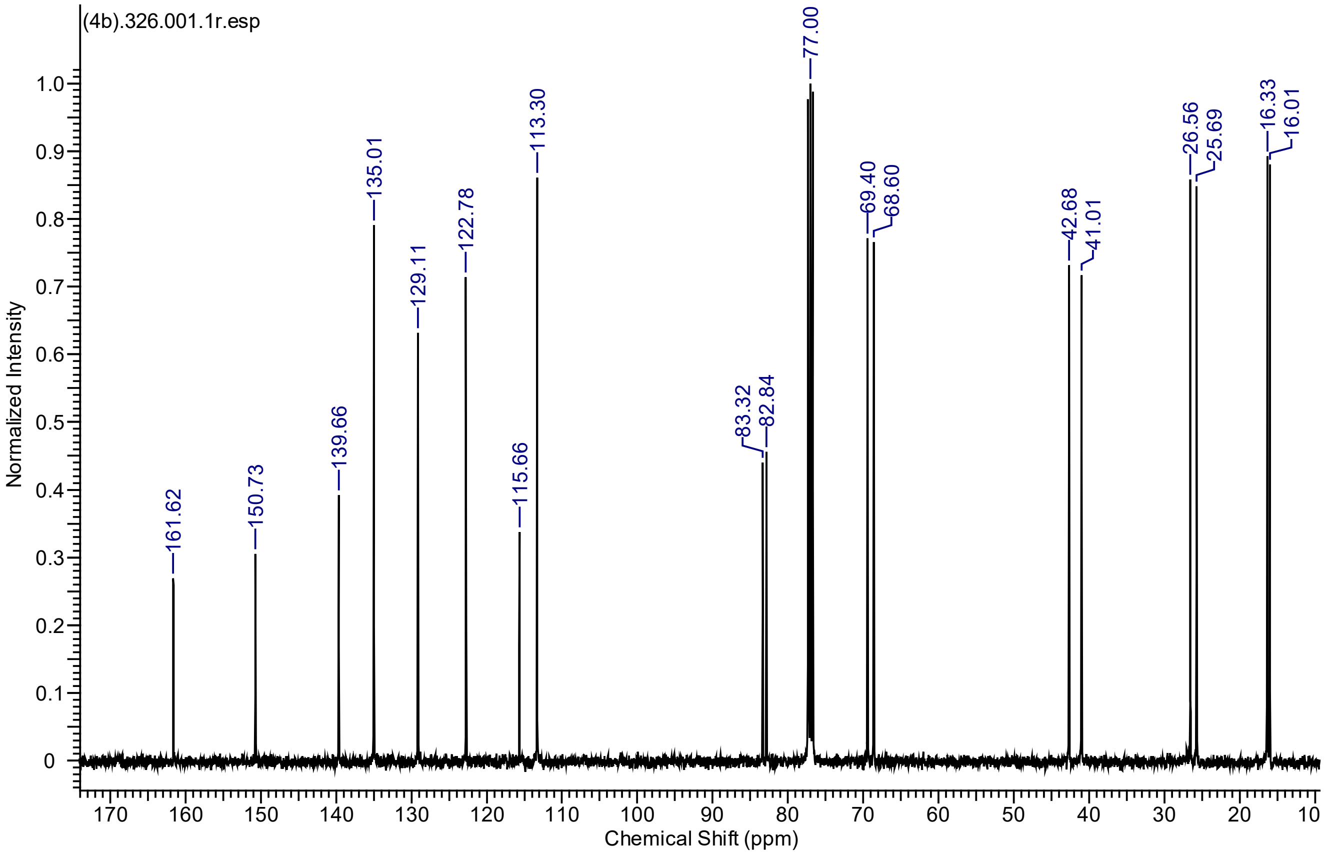

Figure S20. 1D ^{13}C NMR spectrum of **4b** in $CDCl_3$ at T = 303K.

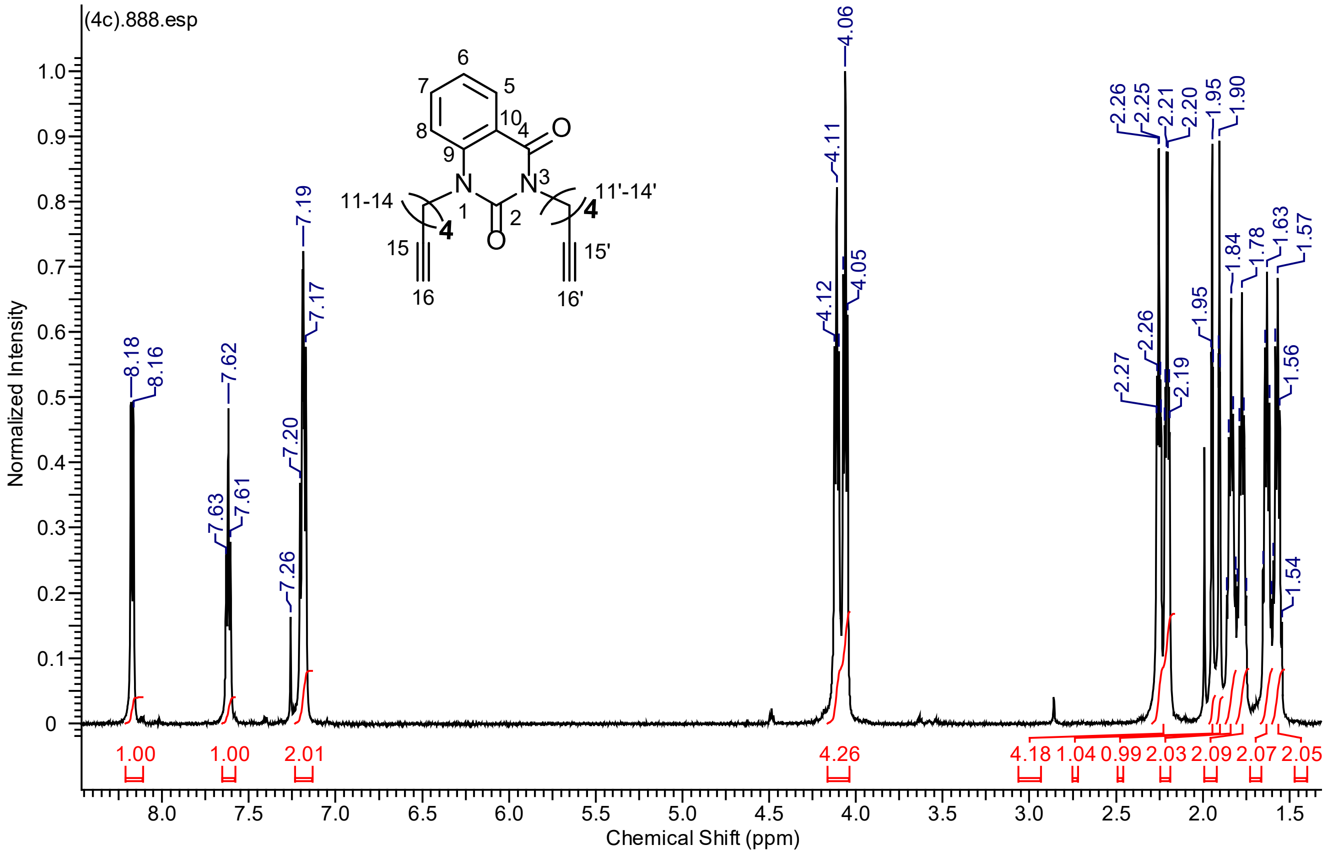

Figure S21. 1D ^{1}H-NMR spectrum of **4c** in $CDCl_3$ at T = 303K.

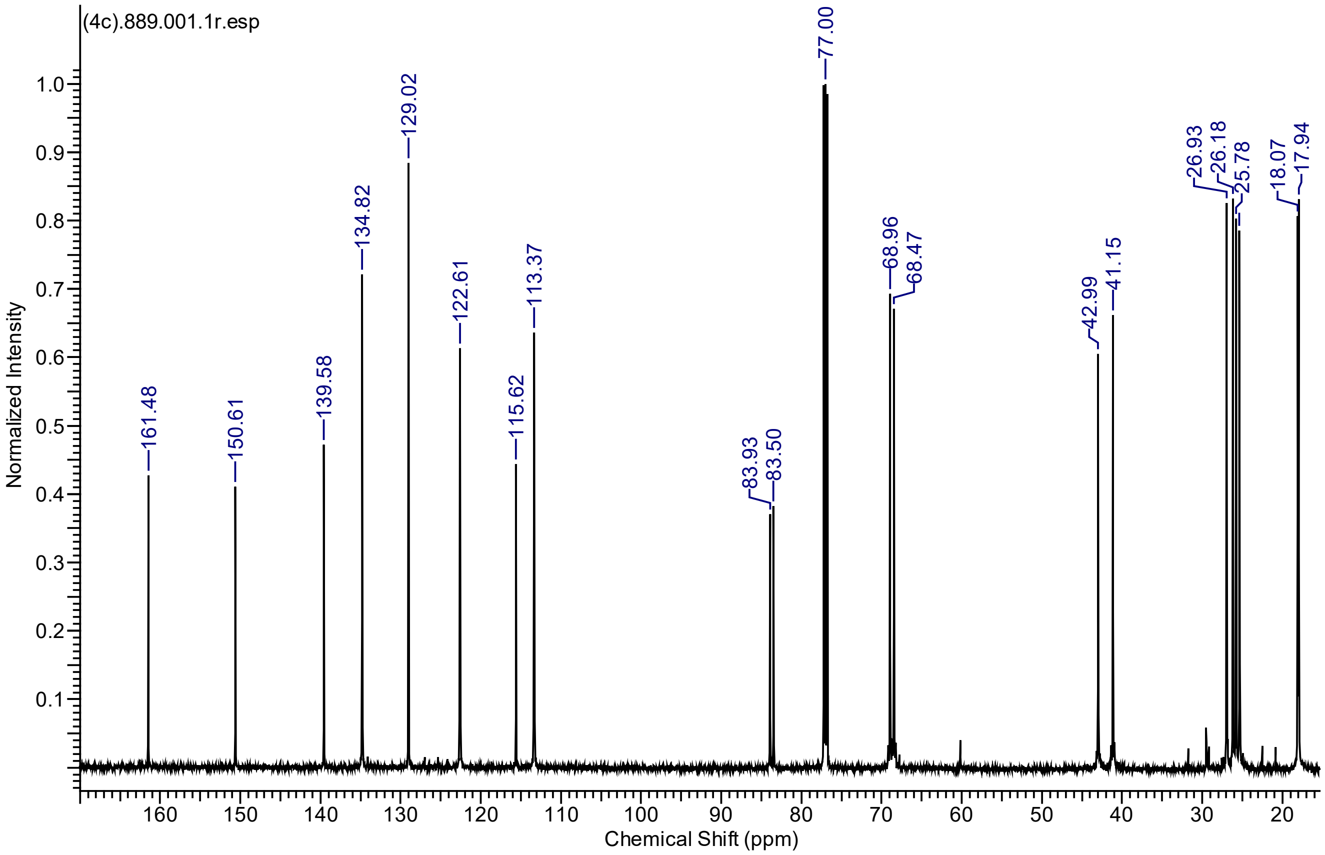

Figure S22. 1D ^{13}C-NMR spectrum of **4c** in $CDCl_3$ at T = 303K.

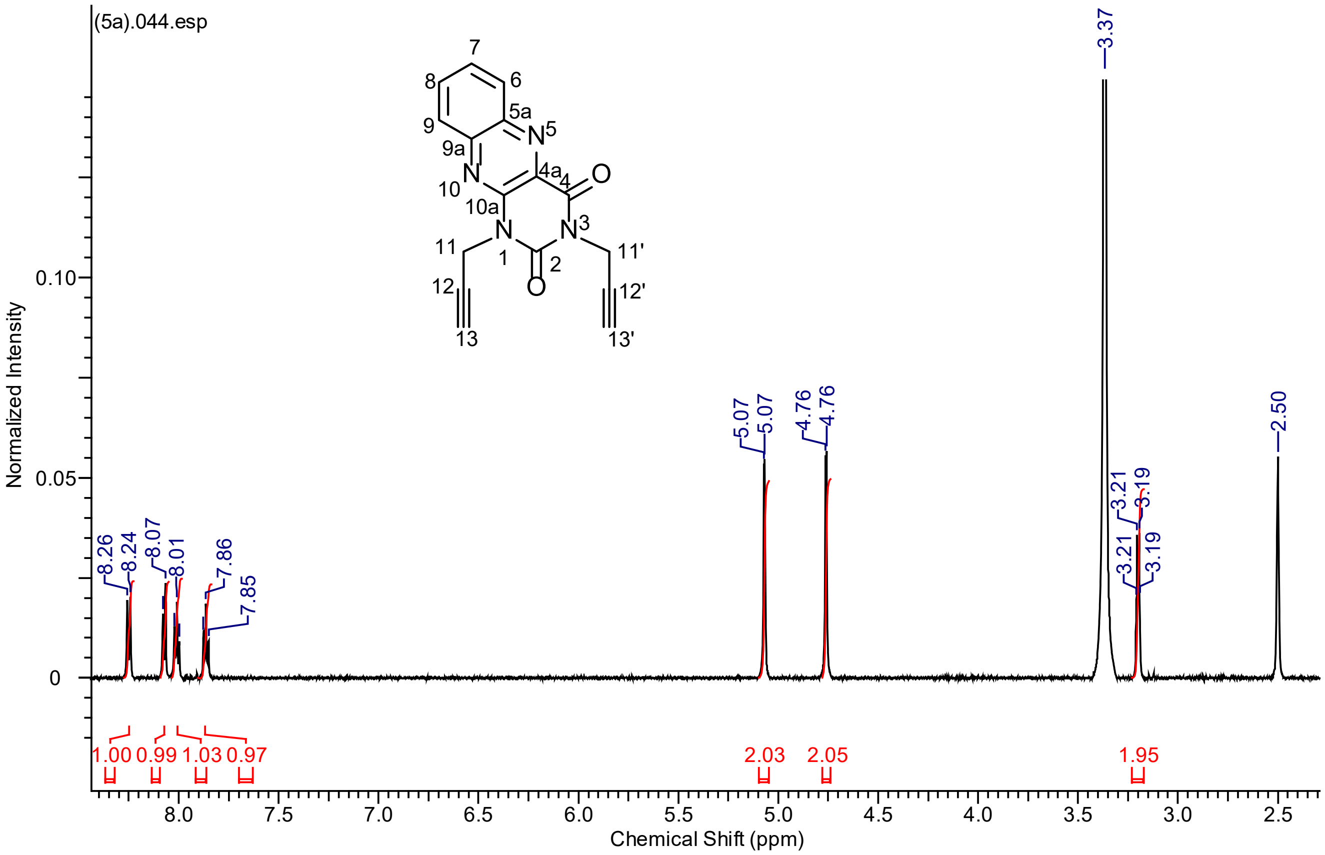

Figure S23. 1D ^{1}H-NMR spectrum of **5a** in DMSO-*d*$_6$ at T = 303K.

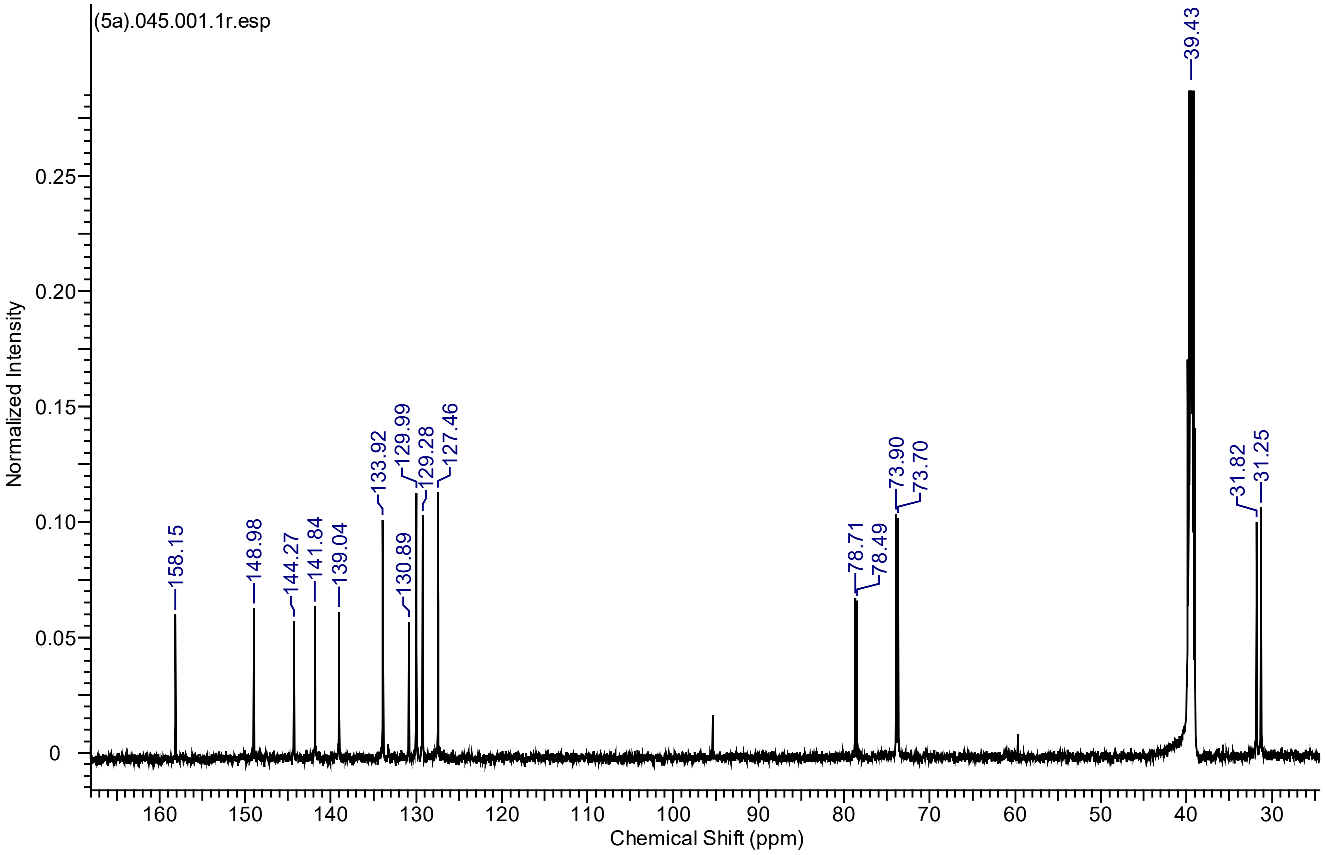

Figure S24. 1D ^{13}C-NMR spectrum of **5a** in DMSO-*d*$_6$ at T = 303K.

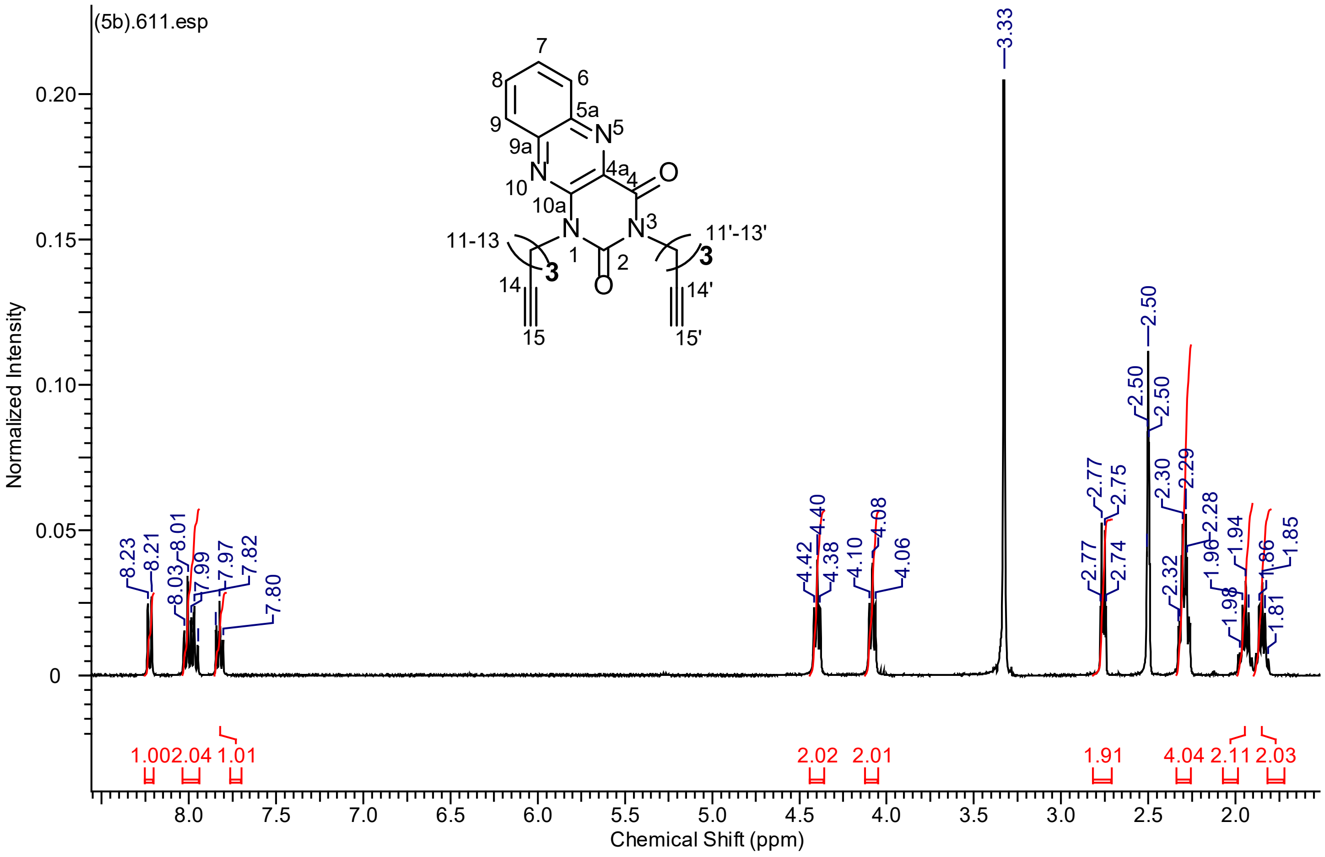

Figure S25. 1D ^{1}H-NMR spectrum of **5b** in DMSO-d_6 at T = 303K.

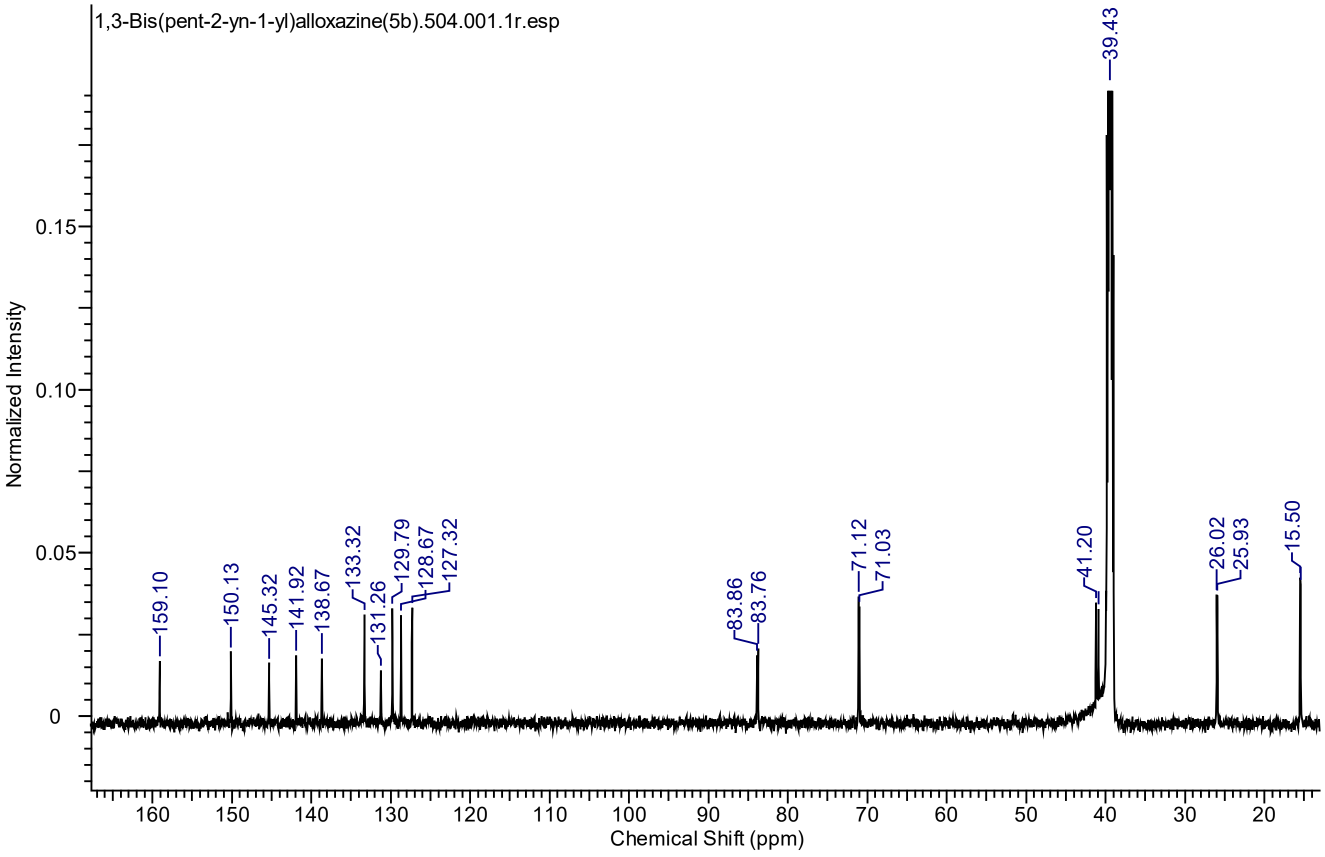

Figure S26. 1D ^{13}C-NMR spectrum of **5b** in DMSO-d_6 at T = 303K.

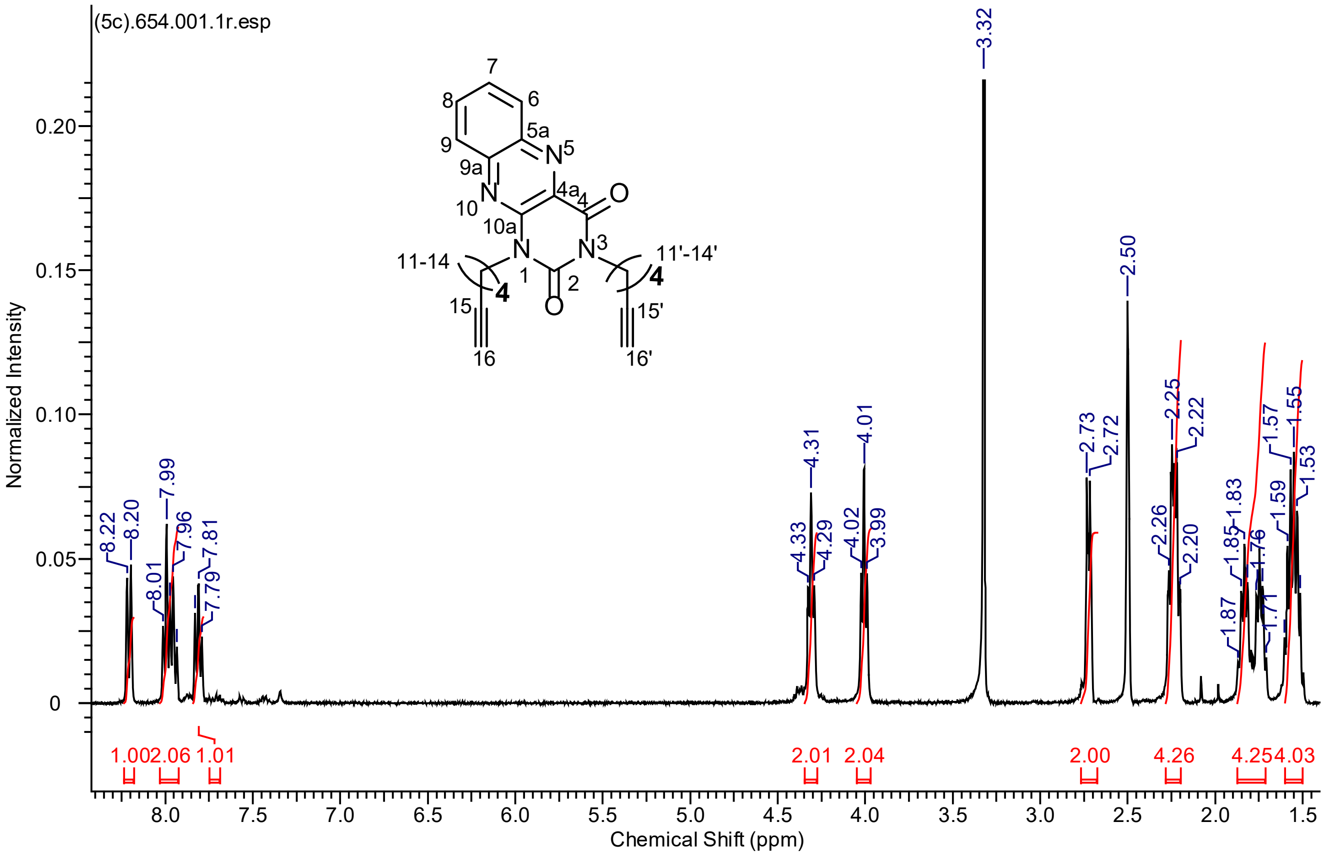

Figure S27. 1D ^{1}H-NMR spectrum of **5c** in DMSO-d_6 at T = 303K.

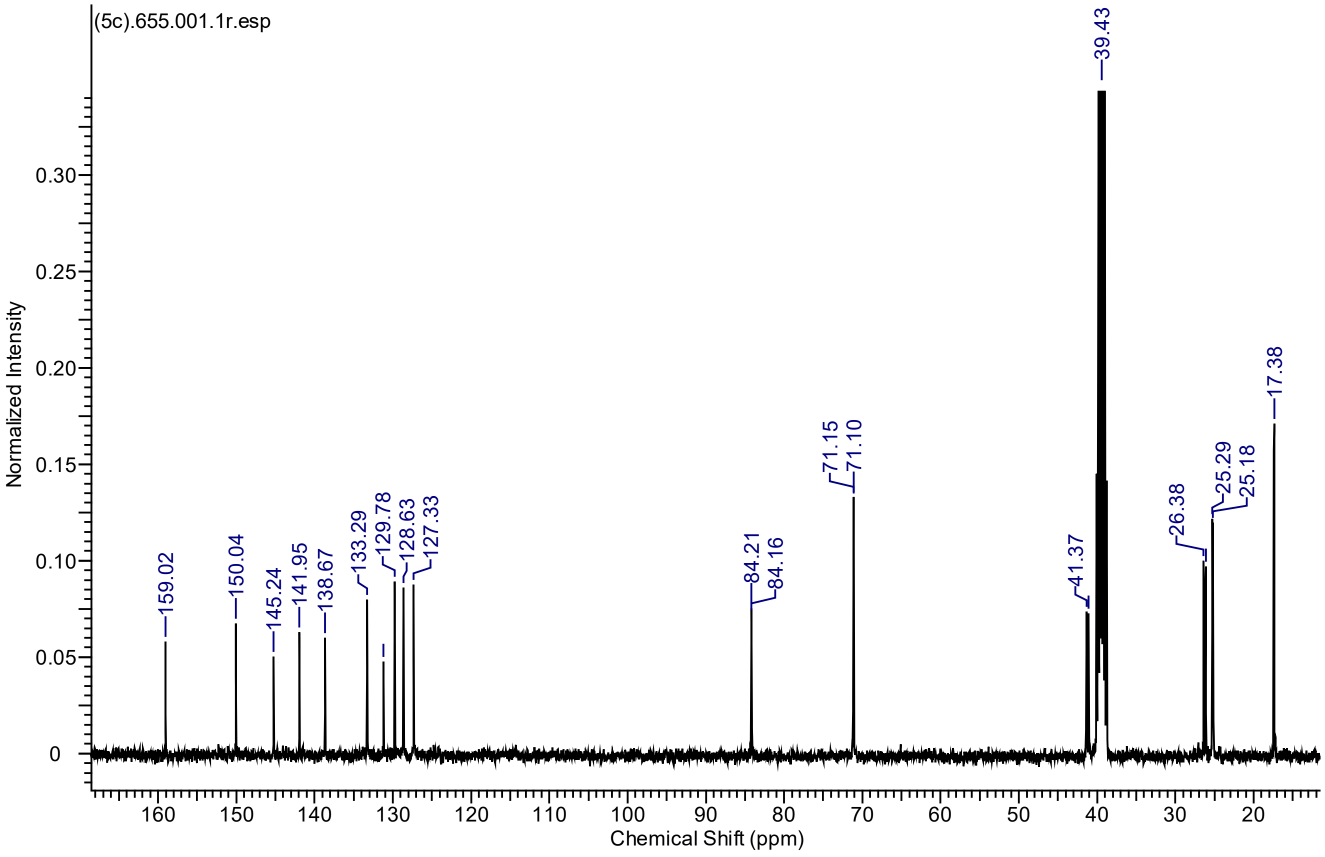

Figure S28. 1D ^{13}C-NMR spectrum of **5c** in DMSO-d_6 at T = 303K.

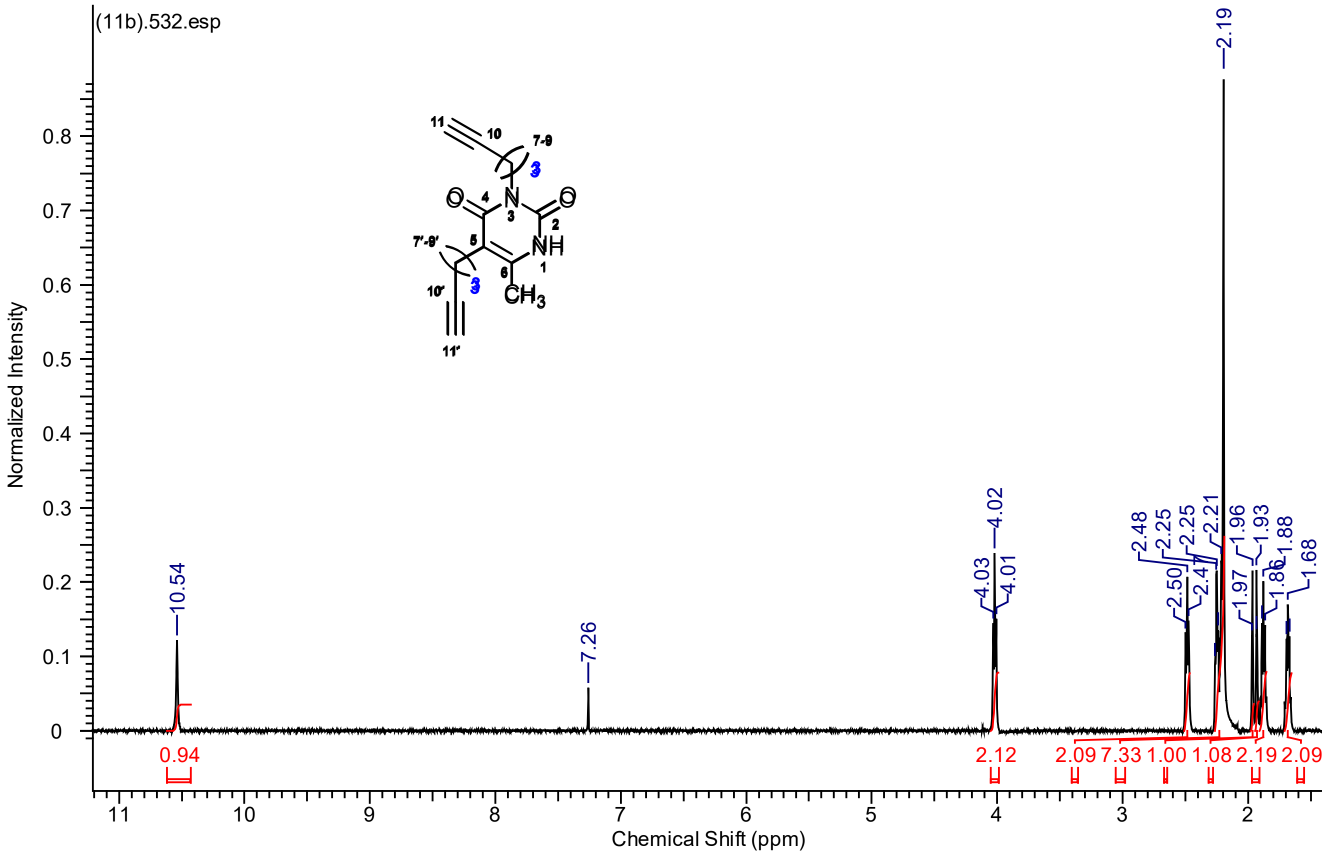

Figure S29. 1D ^{1}H-NMR spectrum of **11a** in DMSO-d_6 at T = 303K.

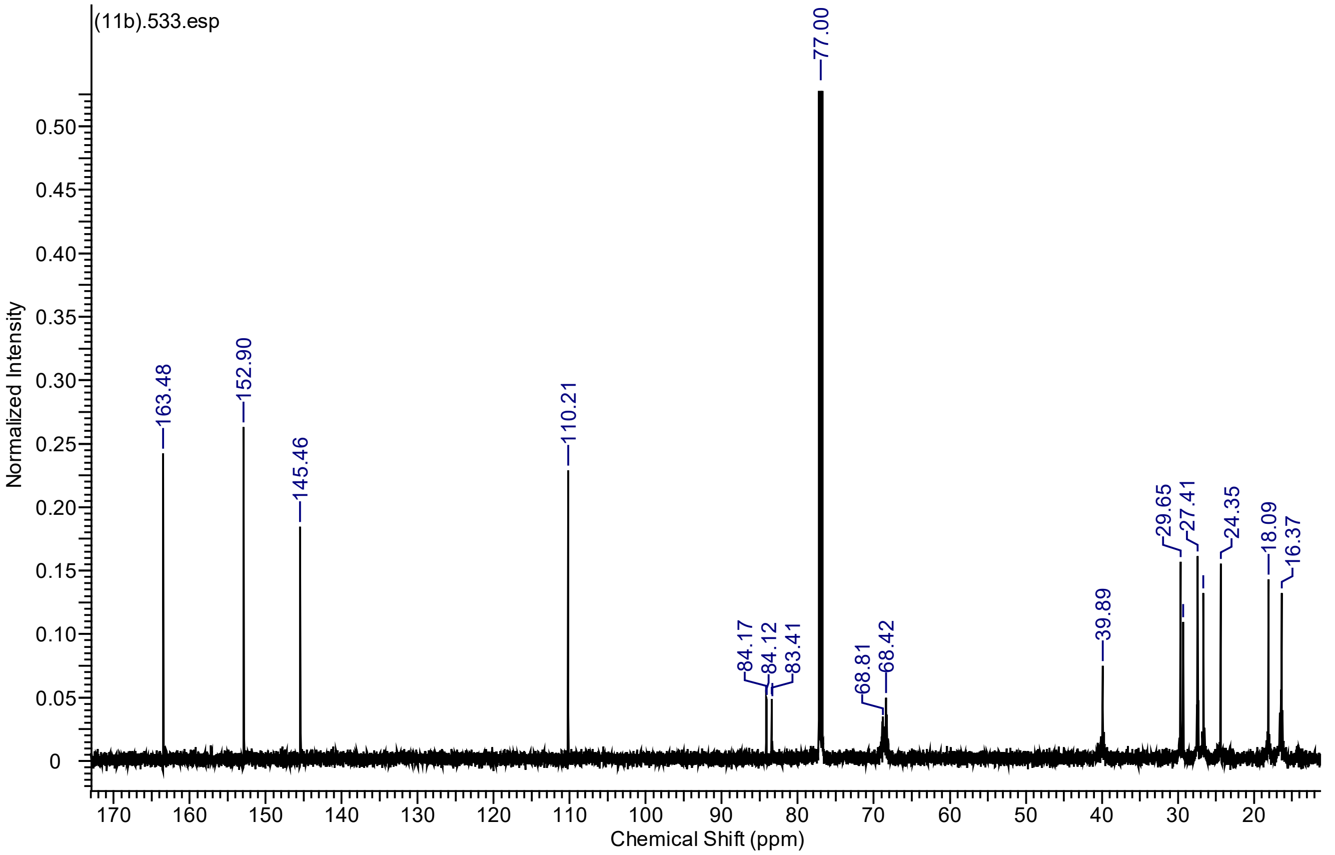

Figure S30. 1D ^{13}C-NMR spectrum of **11a** in DMSO-d_6 at T = 303K.

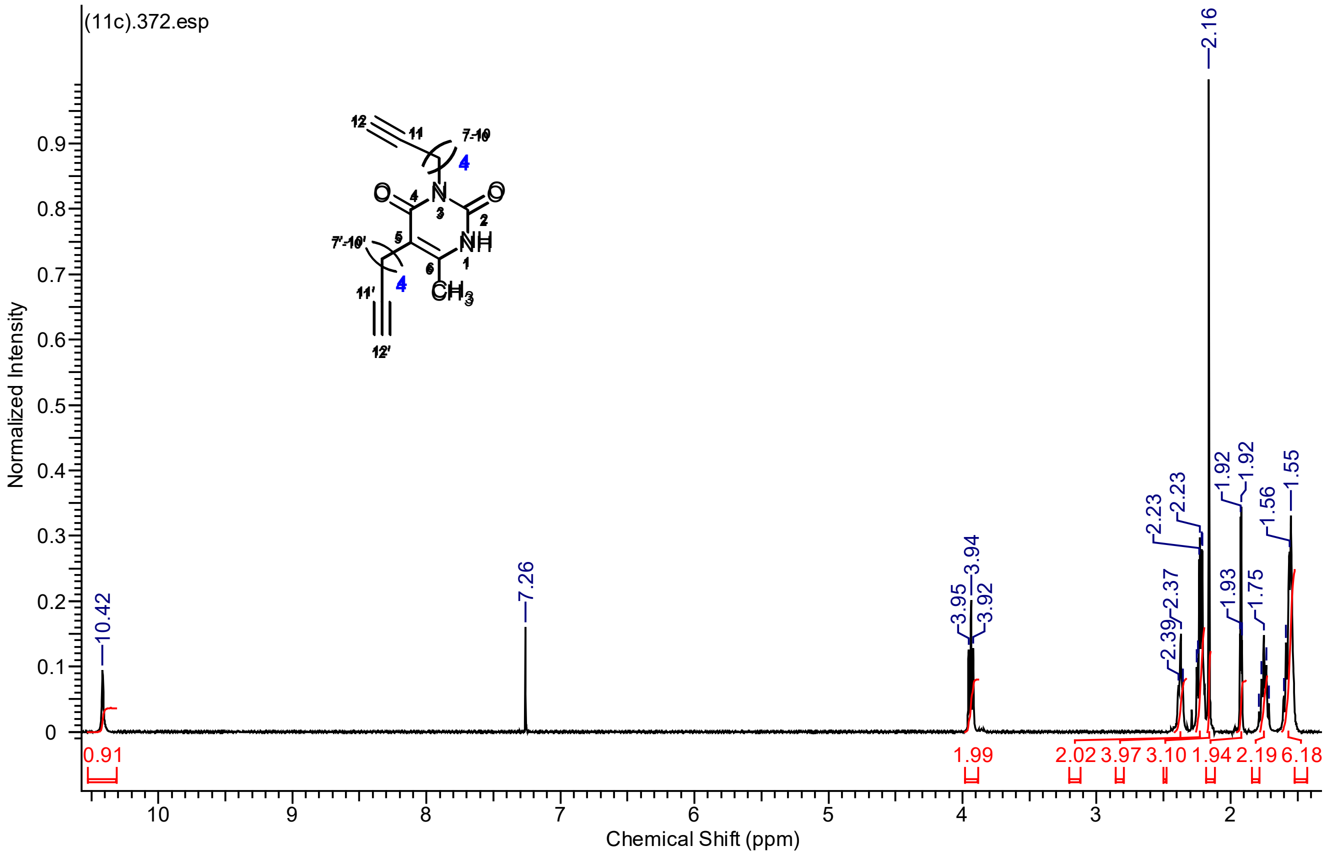

Figure S31. 1D ^{1}H-NMR spectrum of **11b** in DMSO-d_6 at T = 303K.

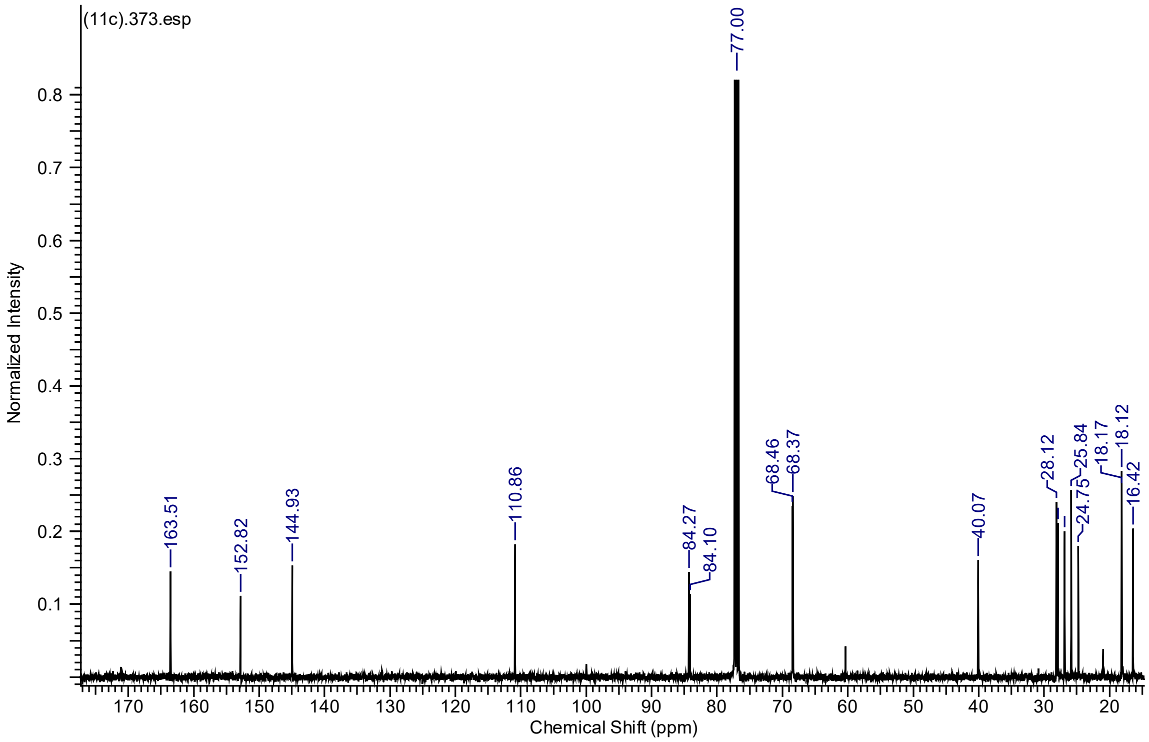

Figure S32. 1D ^{13}C-NMR spectrum of **11b** in DMSO-d_6 at T = 303K.

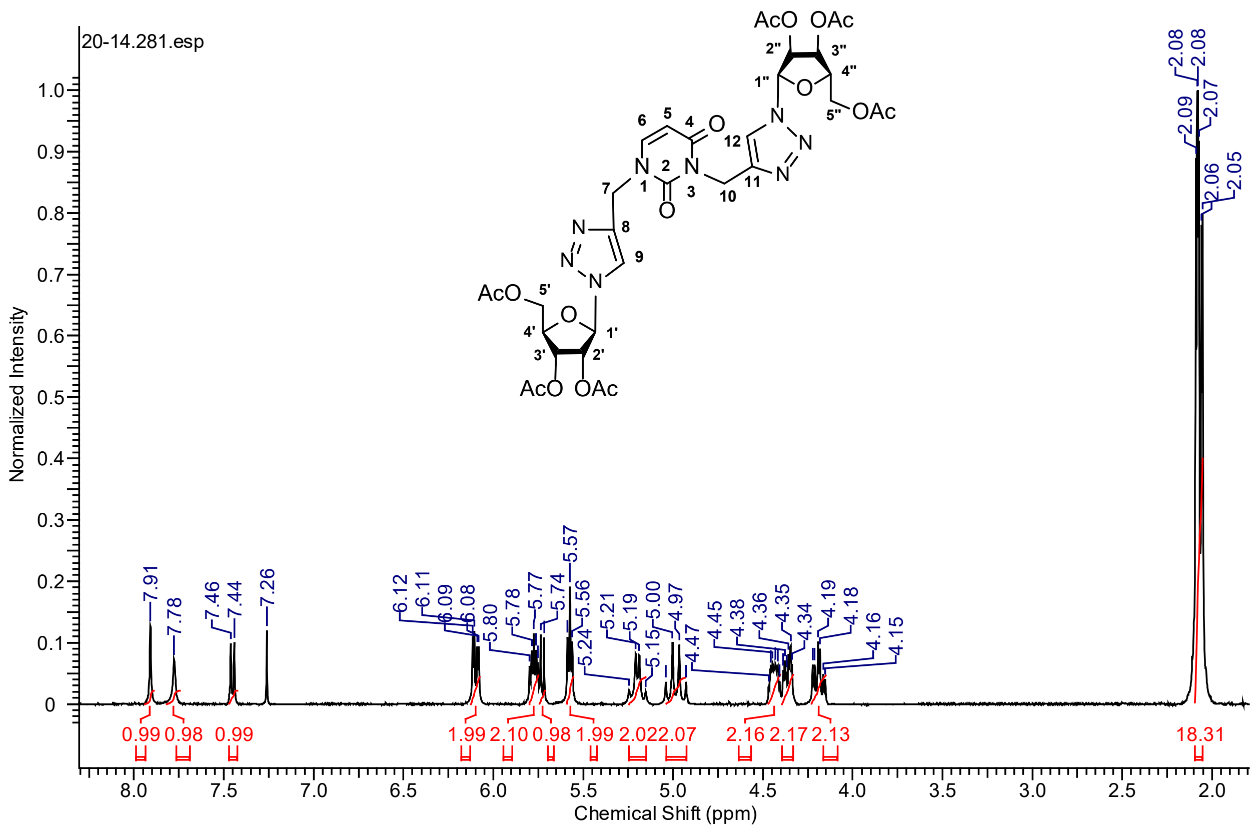

Figure S33. 1D ^{1}H-NMR spectrum of **1d** in $CDCl_3$ at T = 303K.

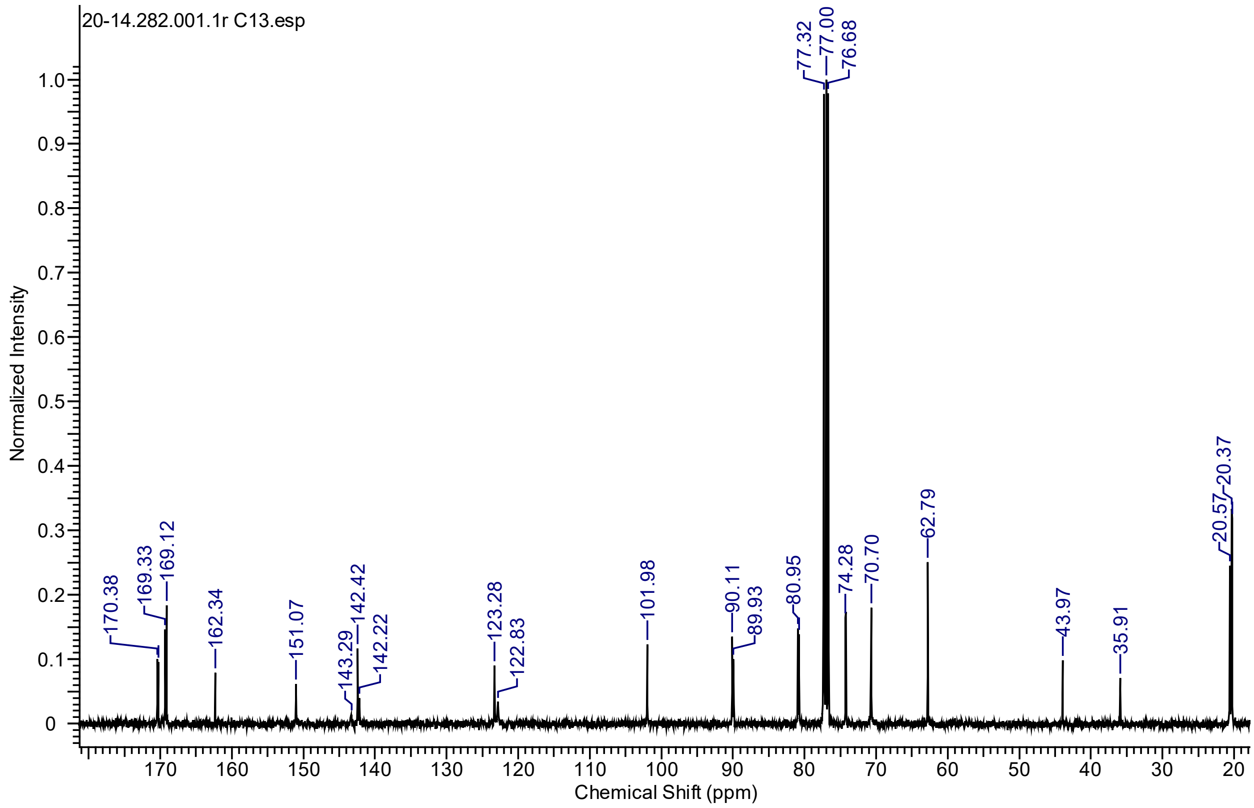

Figure S34. 1D ^{13}C-NMR spectrum of **1d** in $CDCl_3$ at T = 303K.

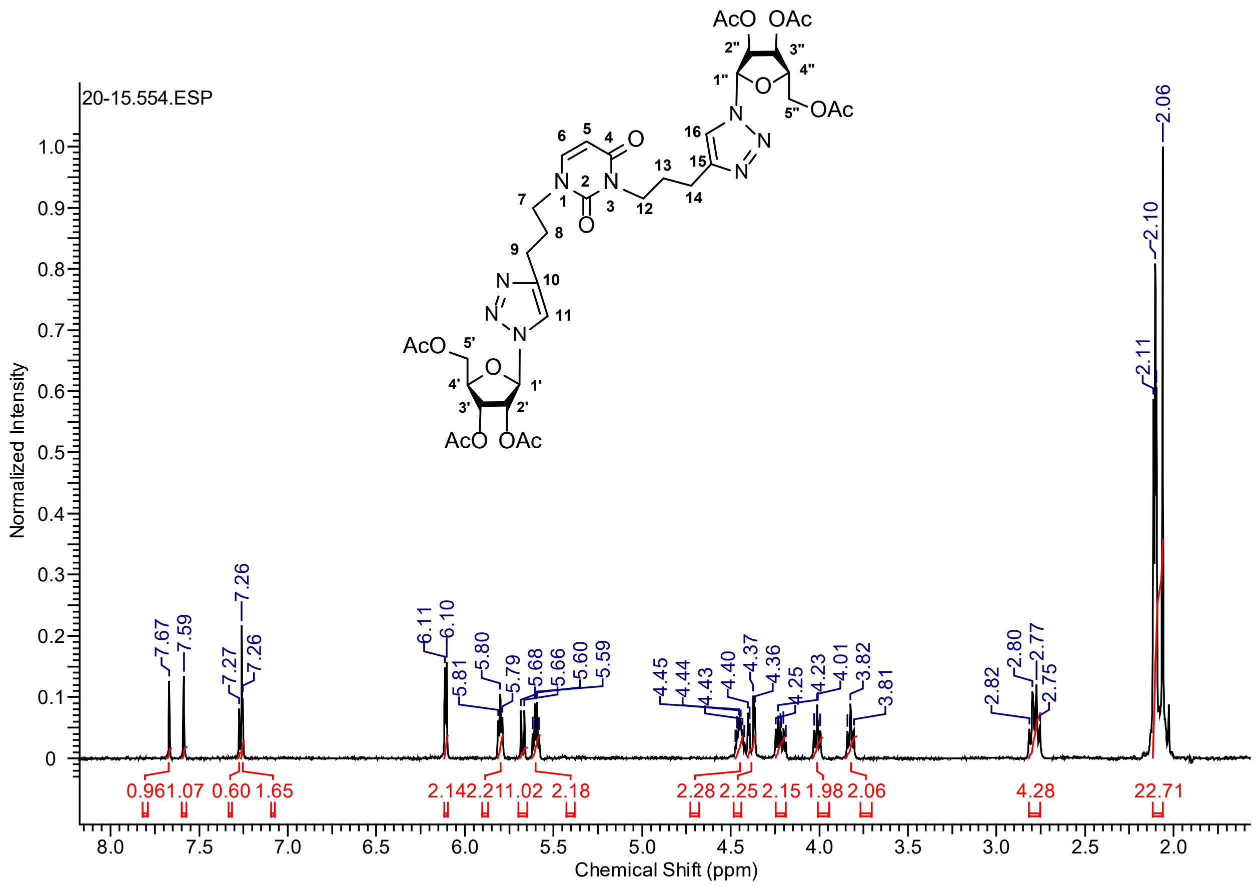

Figure S35. 1D ^{1}H-NMR spectrum of **1e** in $CDCl_3$ at T = 303K.

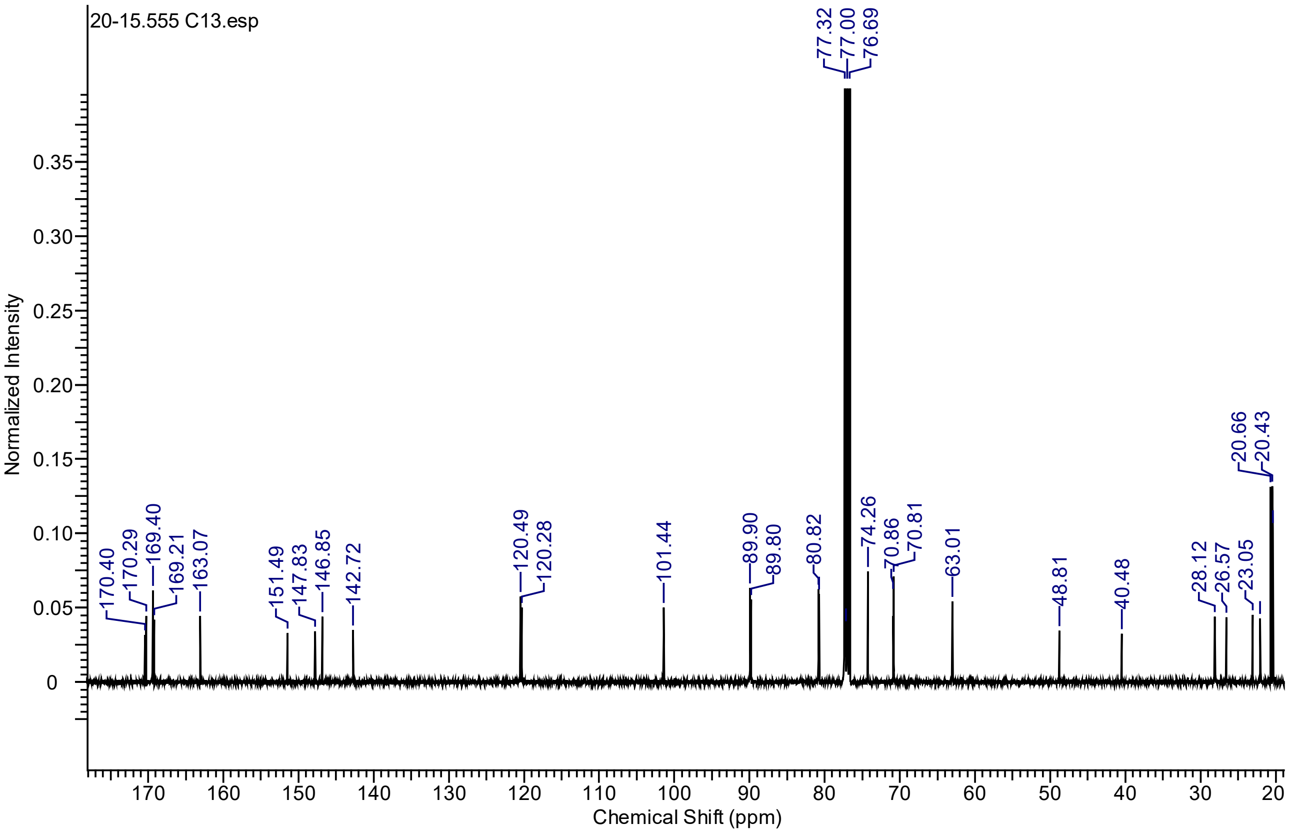

Figure S36. 1D ^{13}C-NMR spectrum of **1e** in $CDCl_3$ at T = 303K.

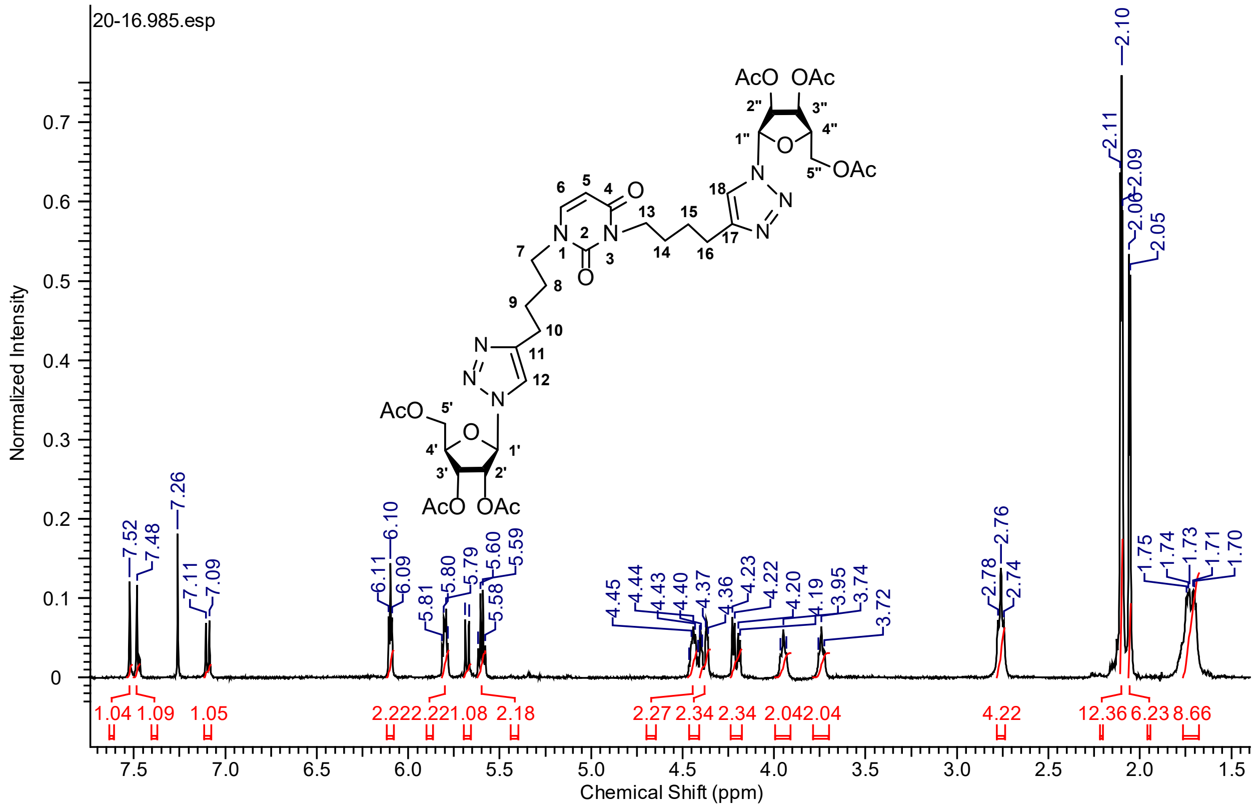

Figure S37. 1D ^{1}H-NMR spectrum of **1f** in $CDCl_3$ at T = 303K.

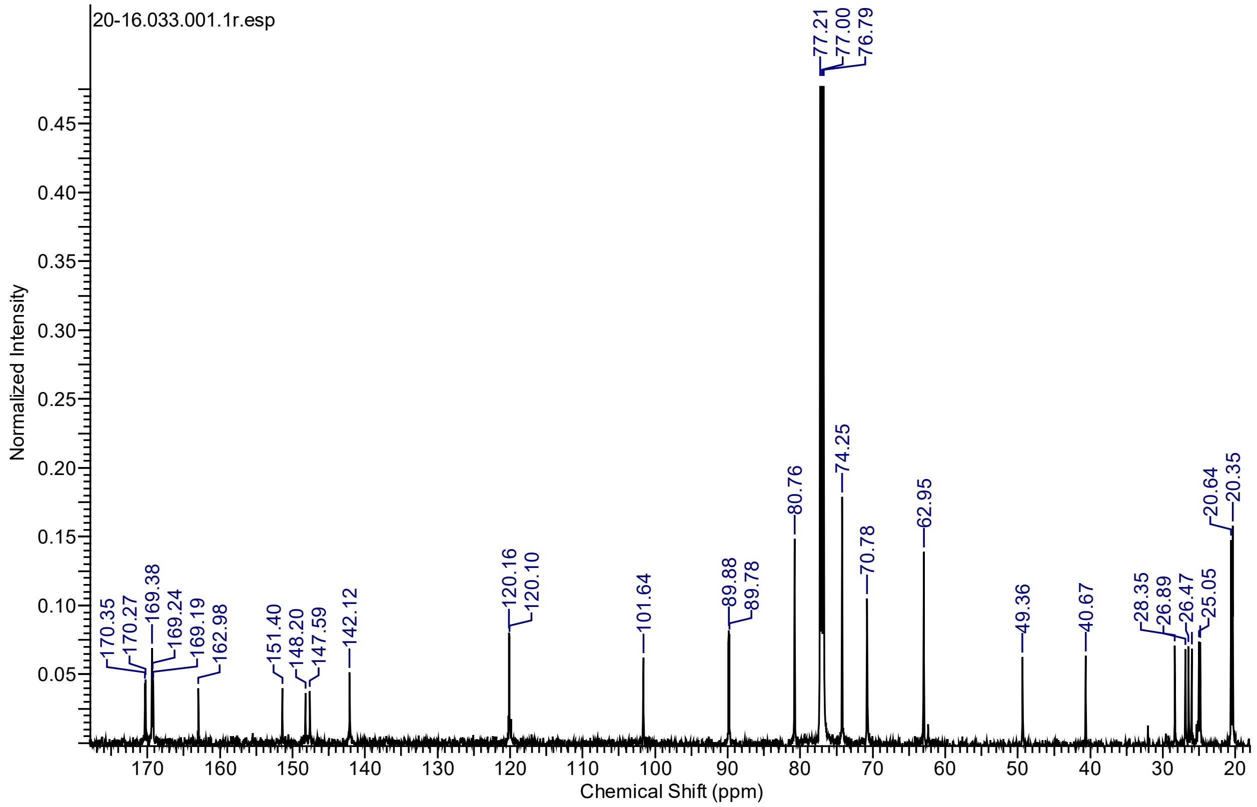

Figure S38. 1D ^{13}C-NMR spectrum of **1f** in $CDCl_3$ at T = 303K.

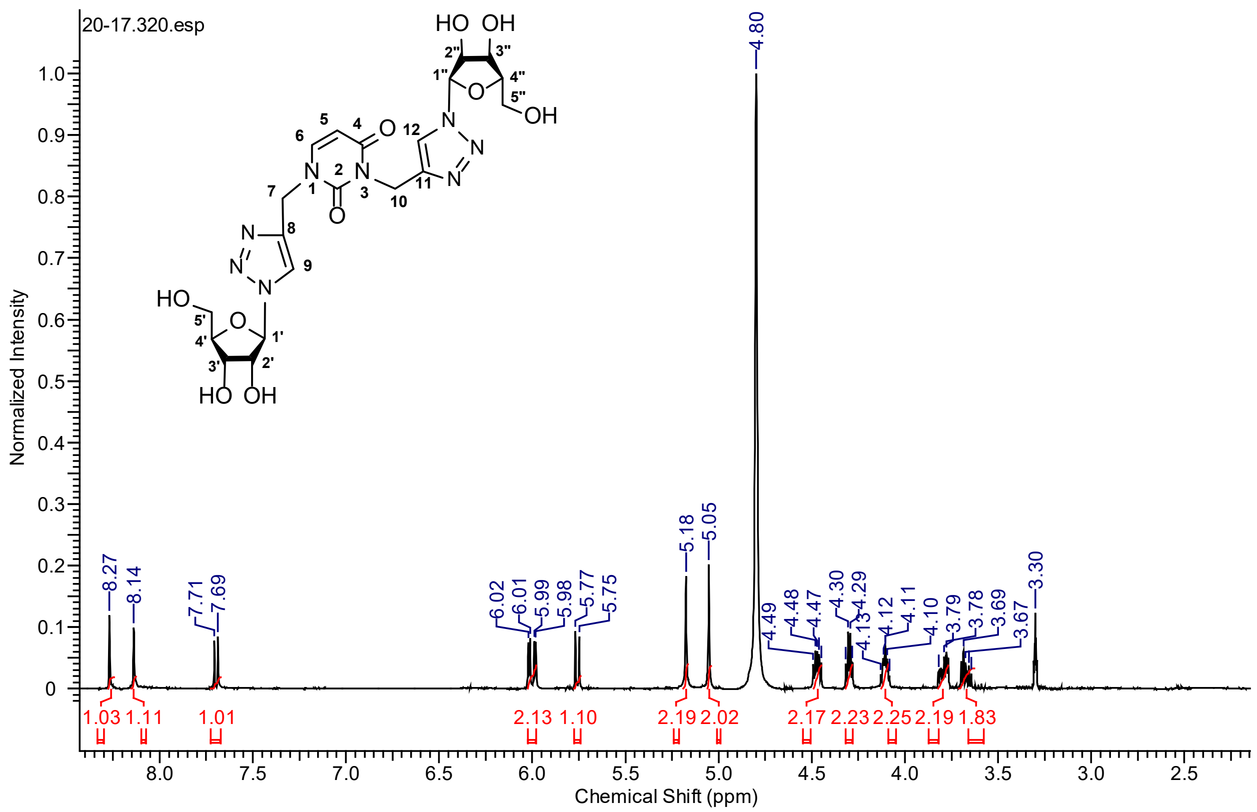

Figure S39. 1D ^{1}H-NMR spectrum of **1g** in CD_3OD at T = 303K.

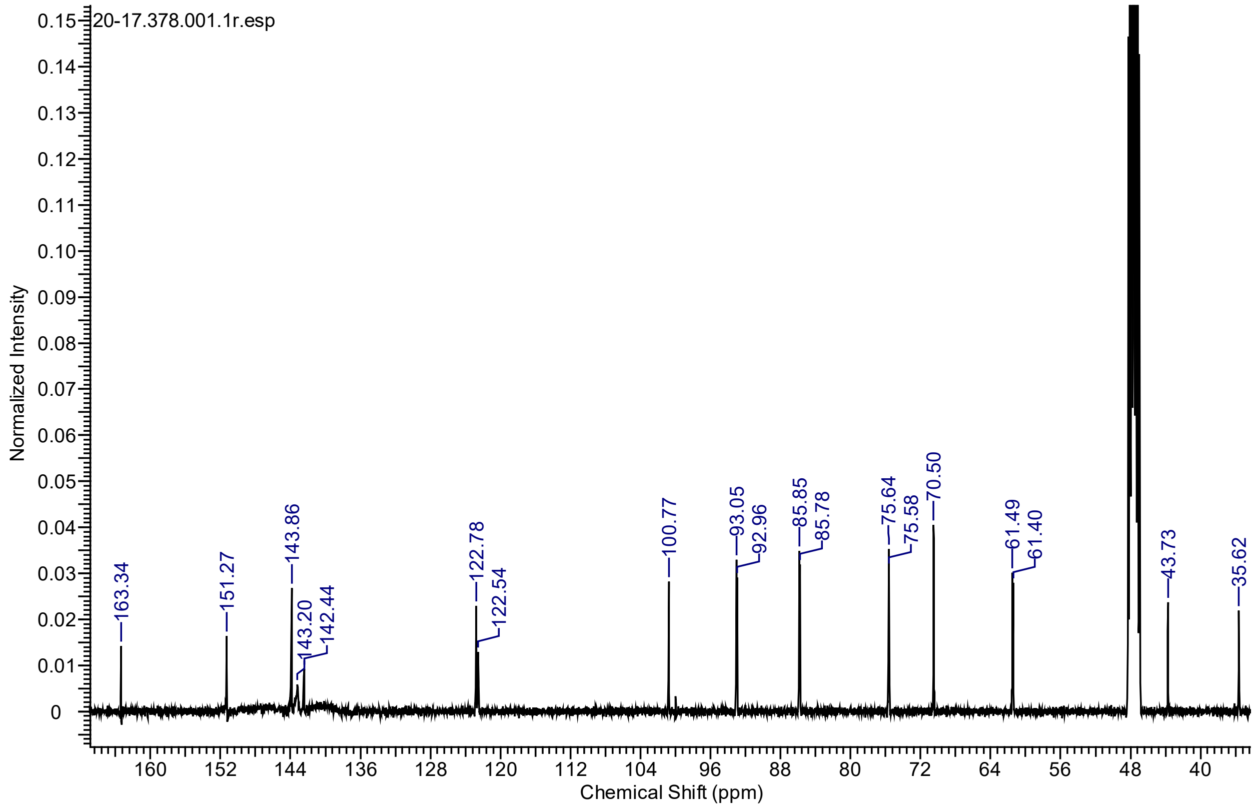

Figure S40. 1D ^{13}C-NMR spectrum of **1g** in CD_3OD at T = 303K.

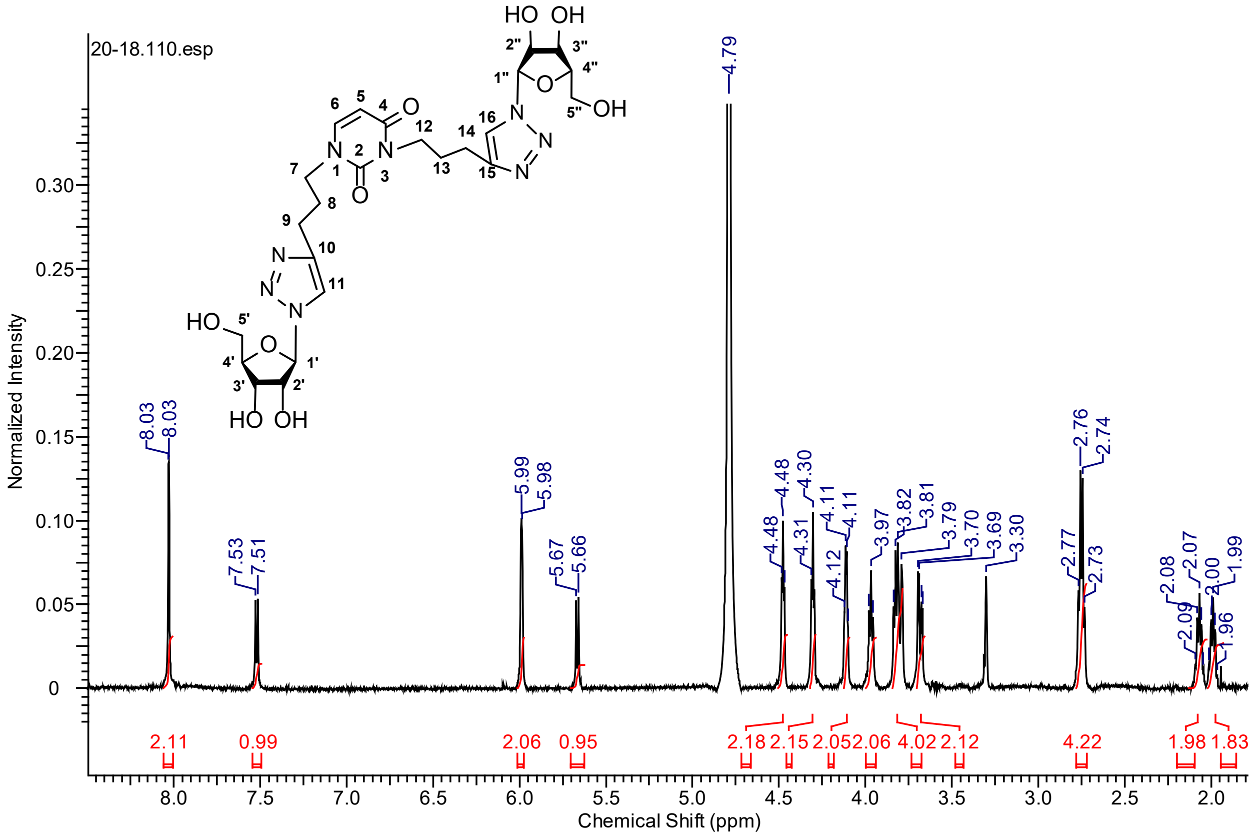

Figure S41. 1D ^{1}H-NMR spectrum of **1h** in CD_3OD at T = 303K.

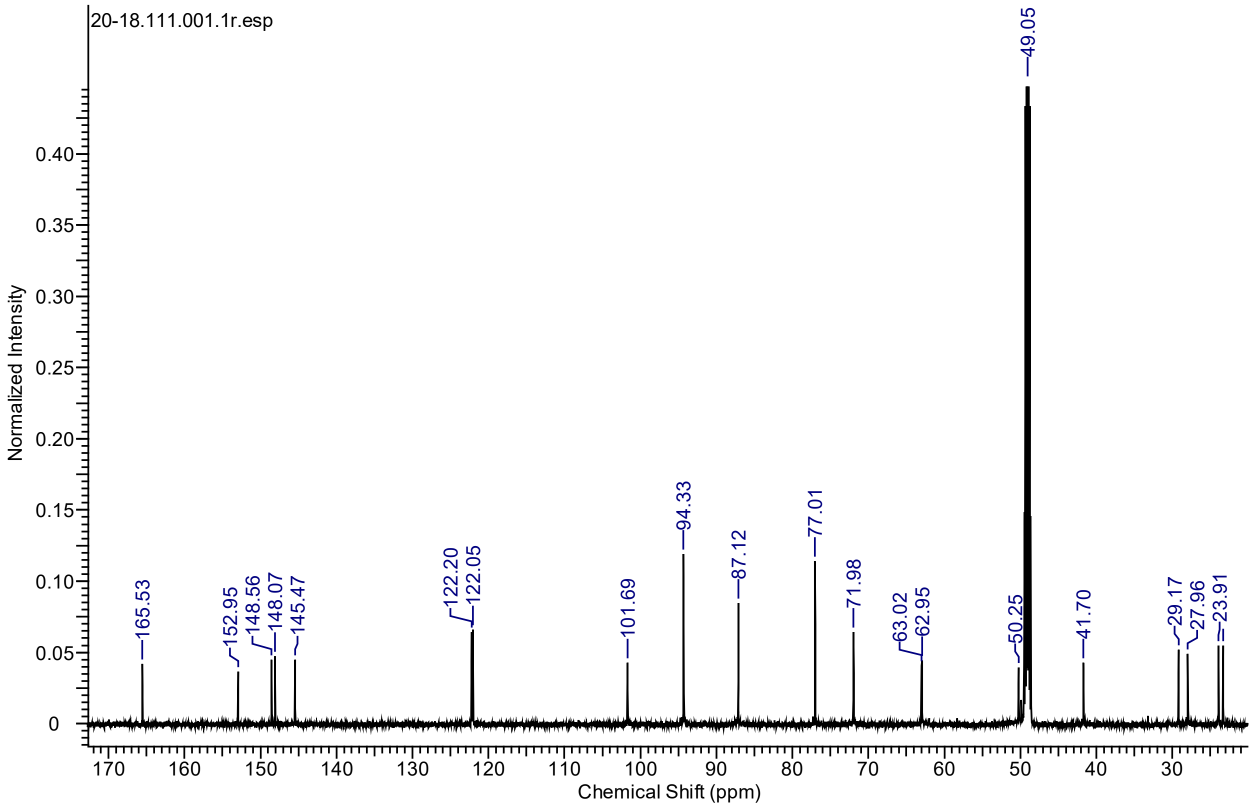

Figure S42. 1D ^{13}C-NMR spectrum of **1h** in CD_3OD at T = 303K.

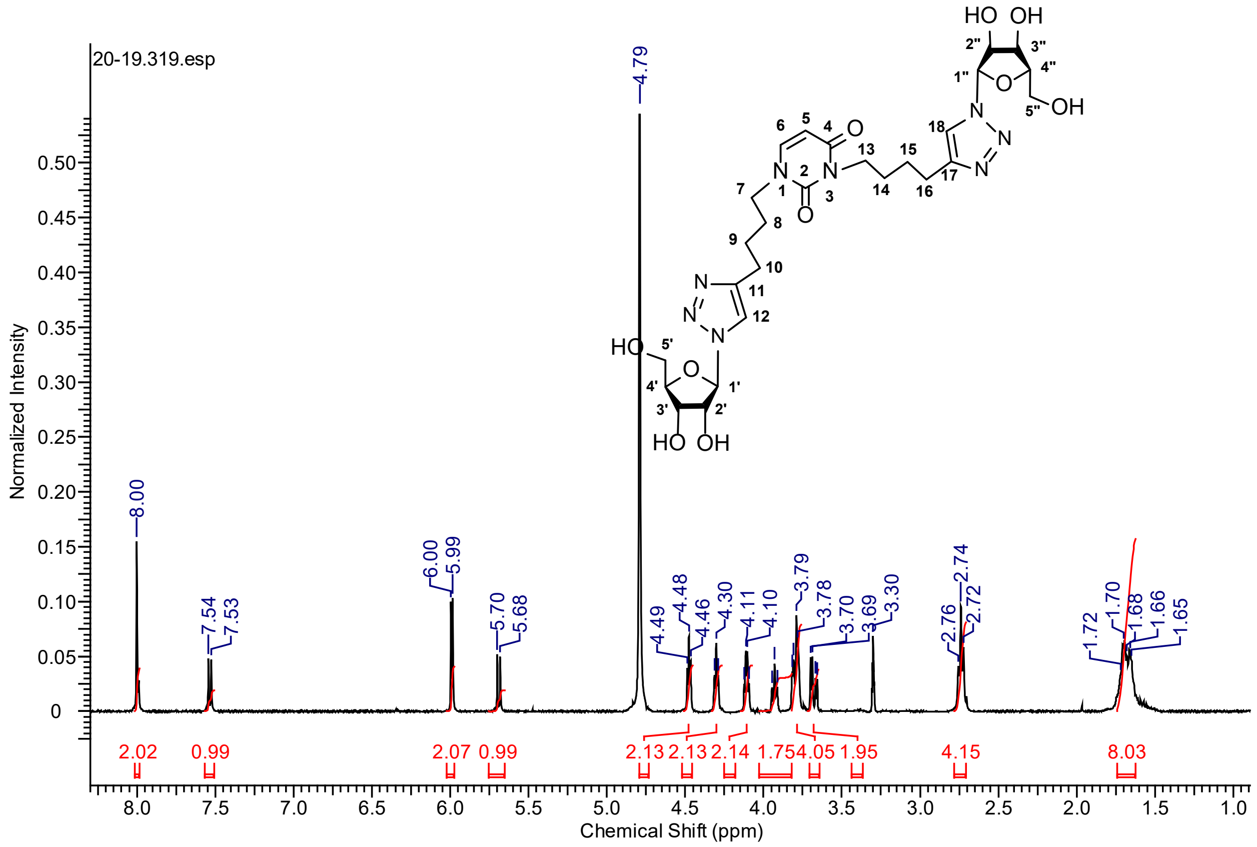

Figure S43. 1D ^{1}H-NMR spectrum of **1i** in CD_3OD at T = 303K.

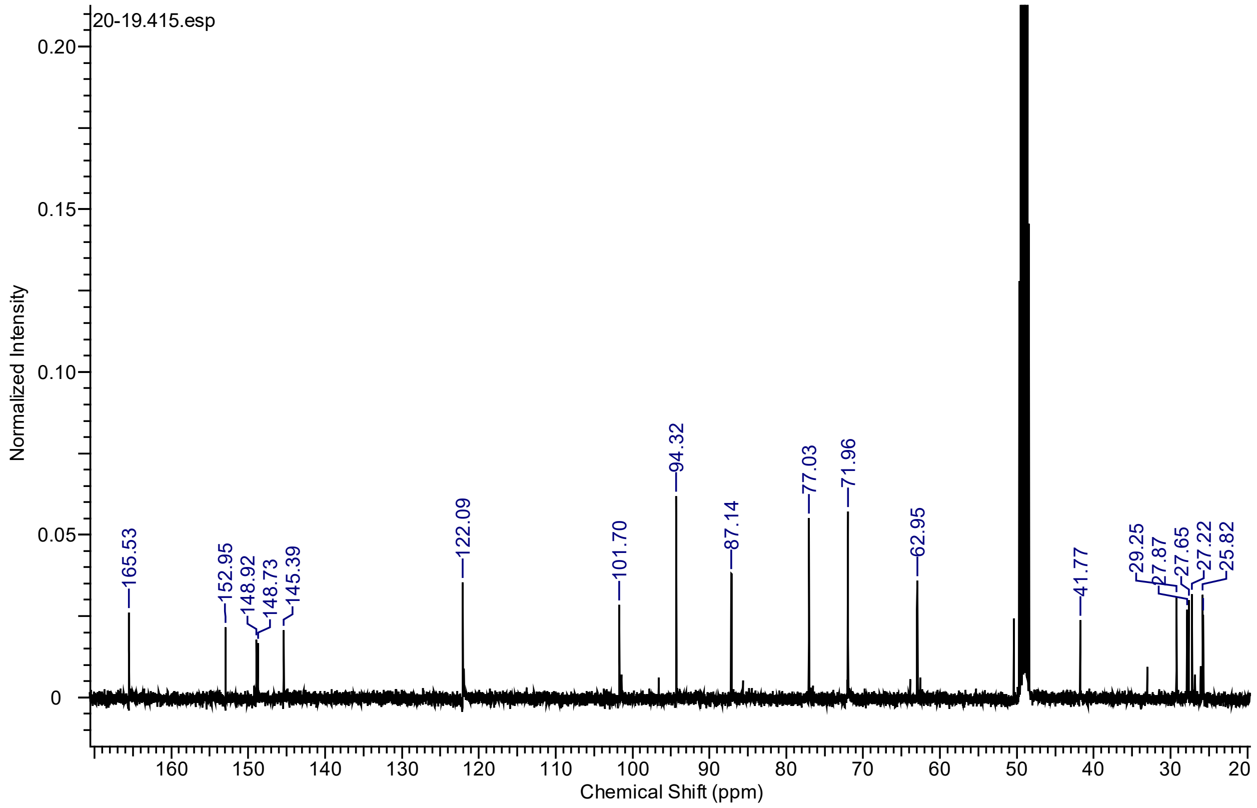

Figure S44. 1D ^{13}H-NMR spectrum of **1i** in CD_3OD at T = 303K.

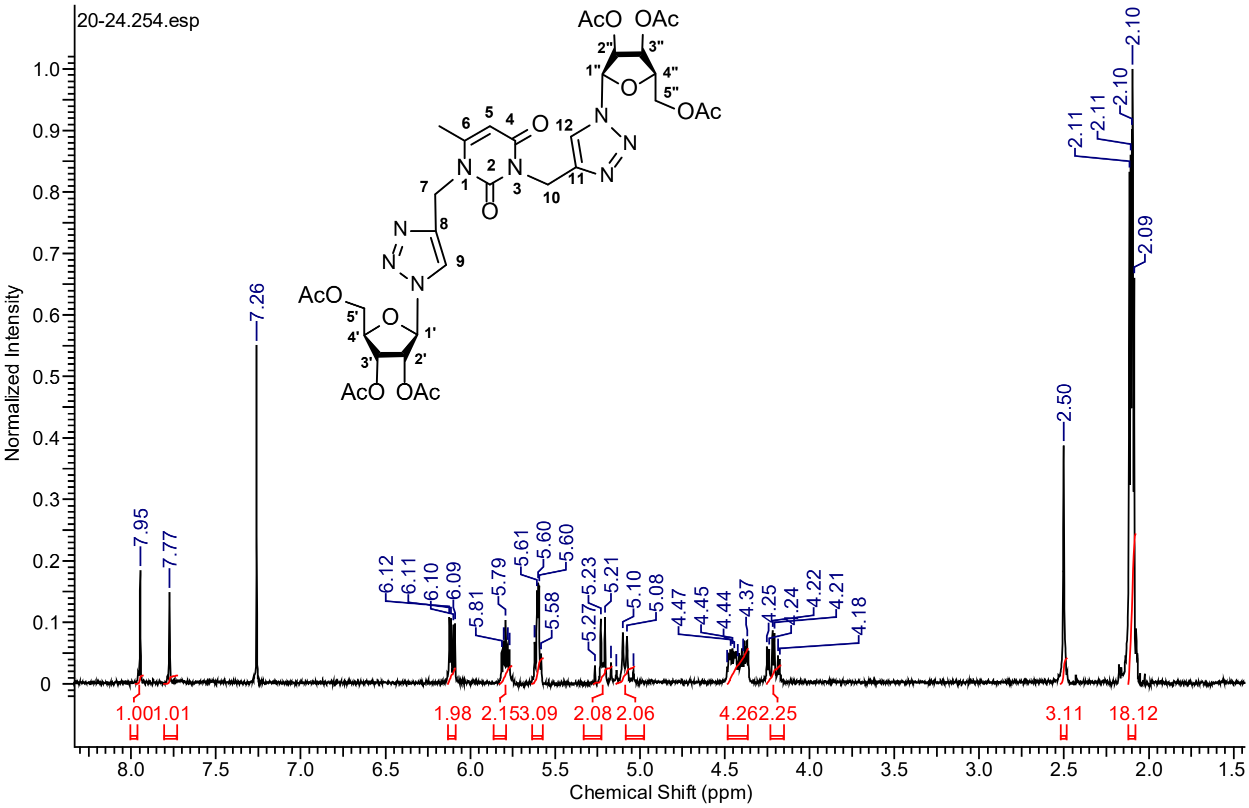

Figure S45. 1D ^{1}H-NMR spectrum of **2d** in CDCl3 at T = 303K.

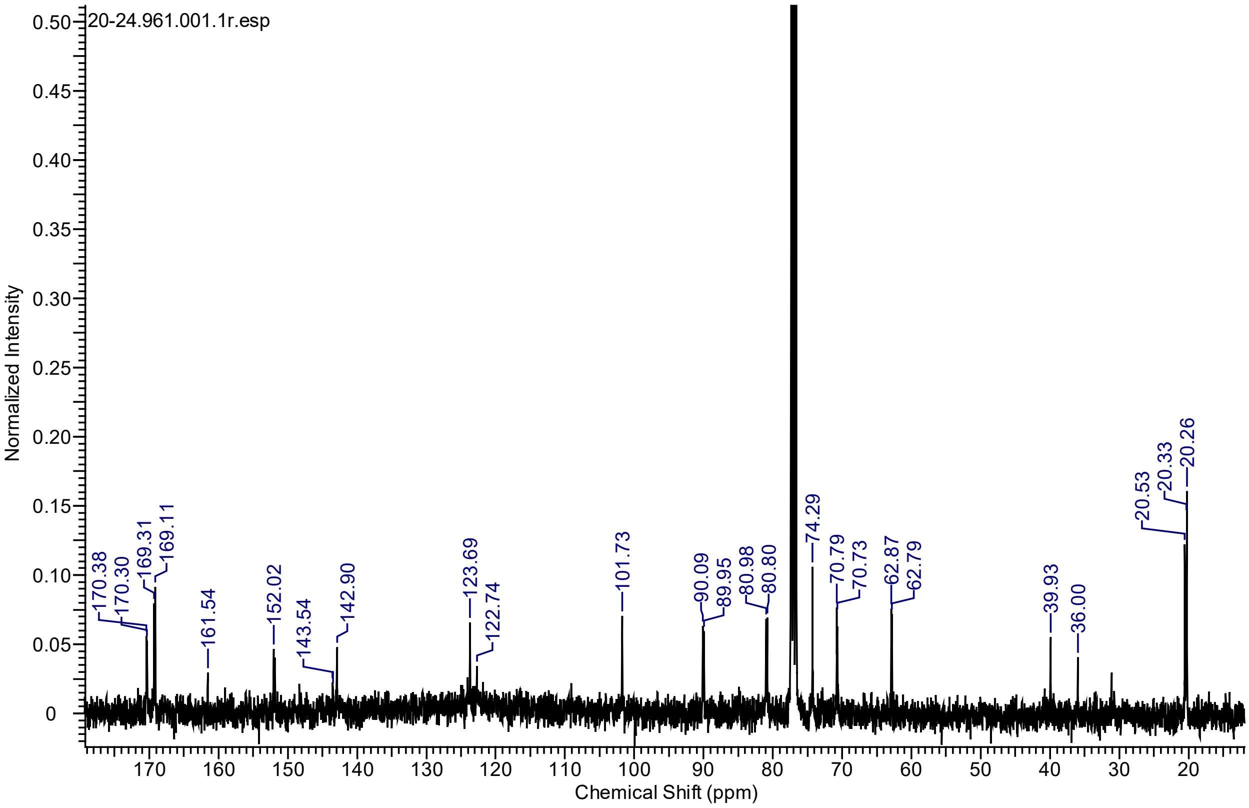

Figure S46. 1D ^{1}H-NMR spectrum of **2d** in CDCl3 at T = 303K.

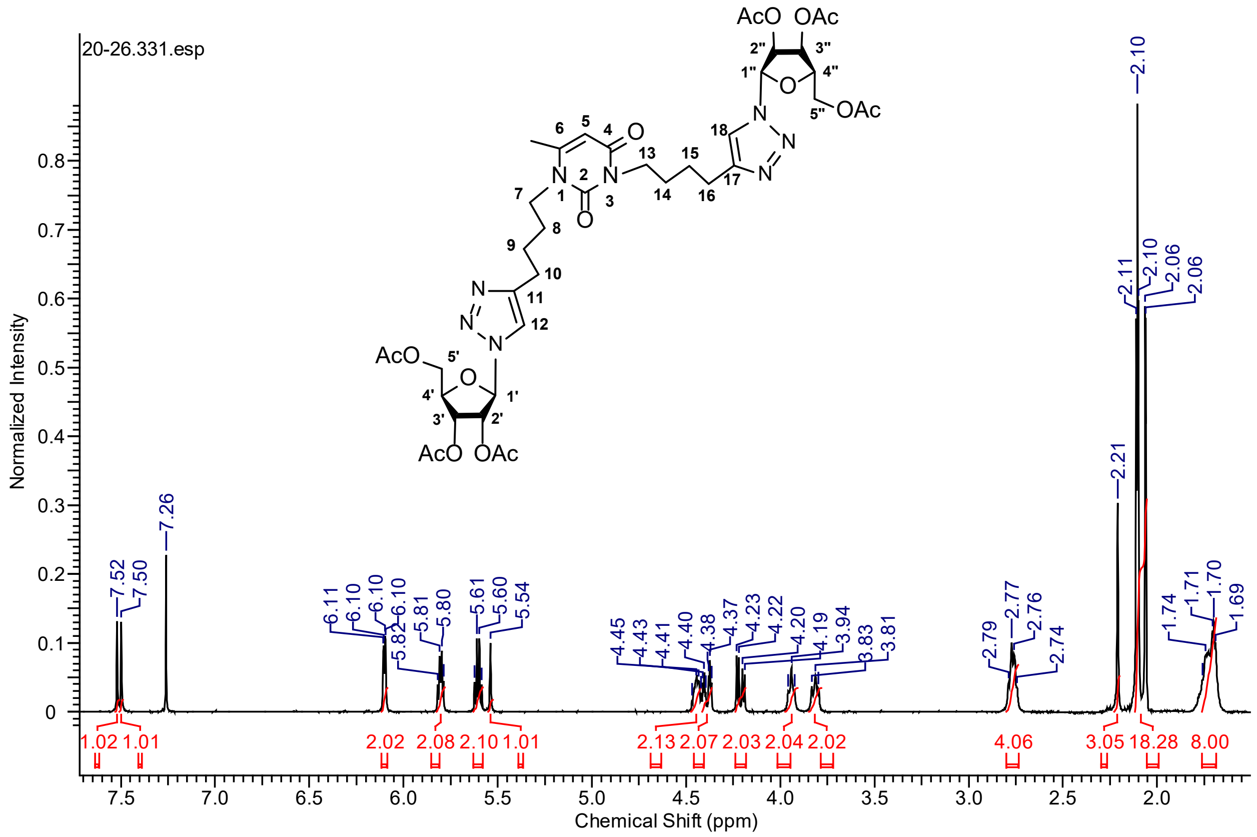

Figure S47. 1D ^{1}H-NMR spectrum of **2f** in $CDCl_3$ at T = 303K.

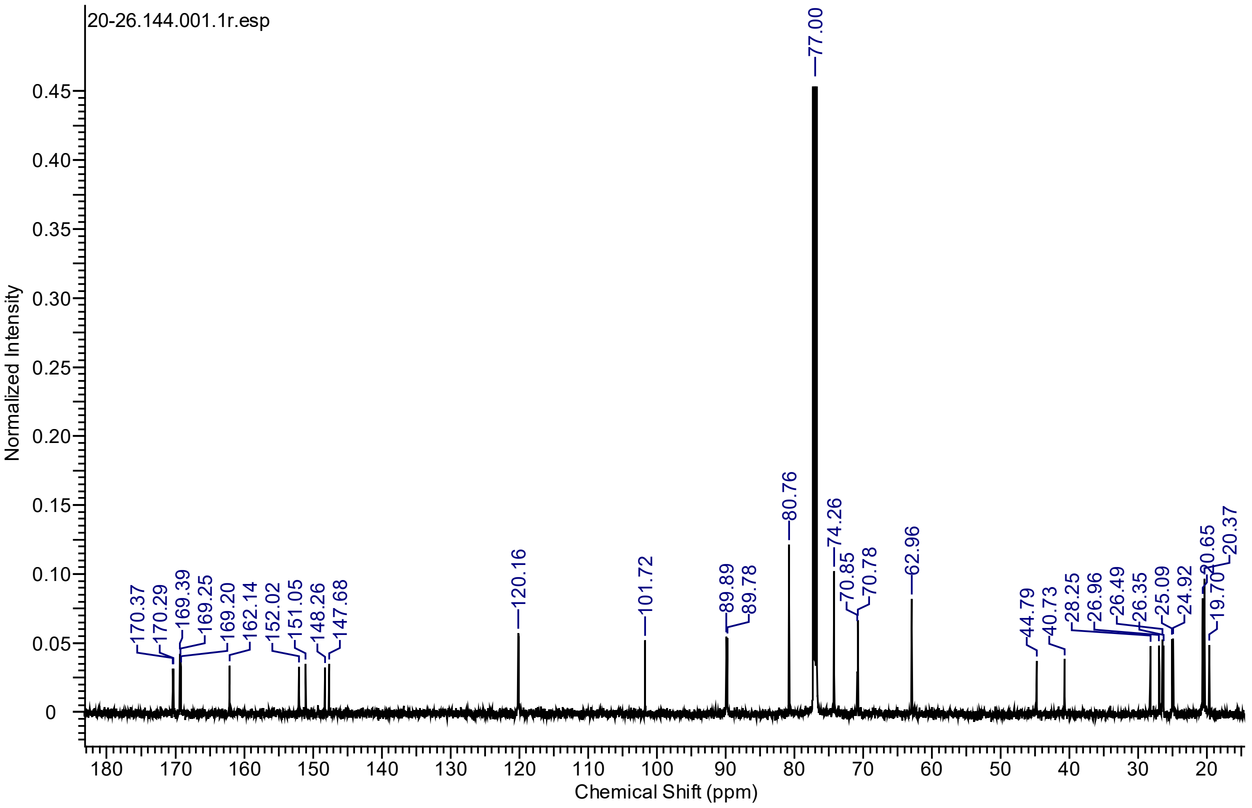

Figure S48. 1D ^{13}C-NMR spectrum of **2f** in $CDCl_3$ at T = 303K.

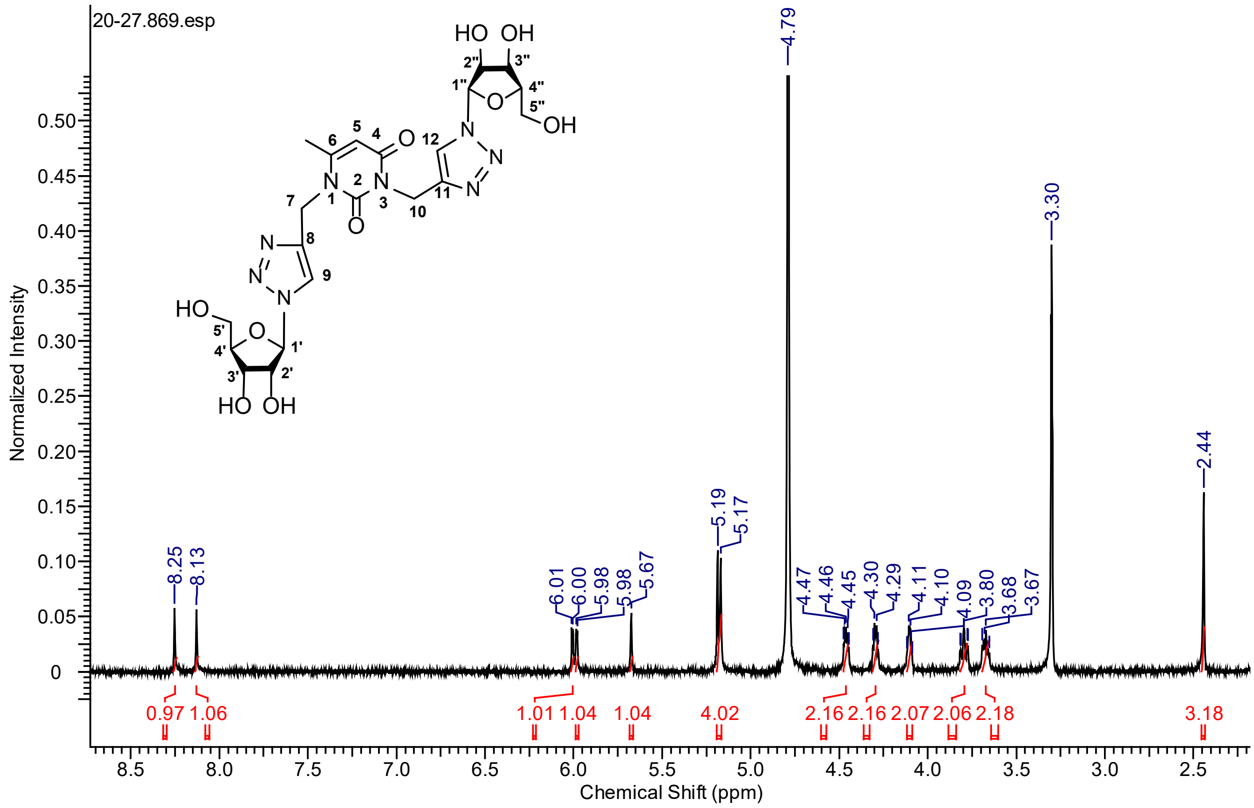

Figure S49. 1D ^{1}H-NMR spectrum of **2g** in CD$_3$OD at T = 303K.

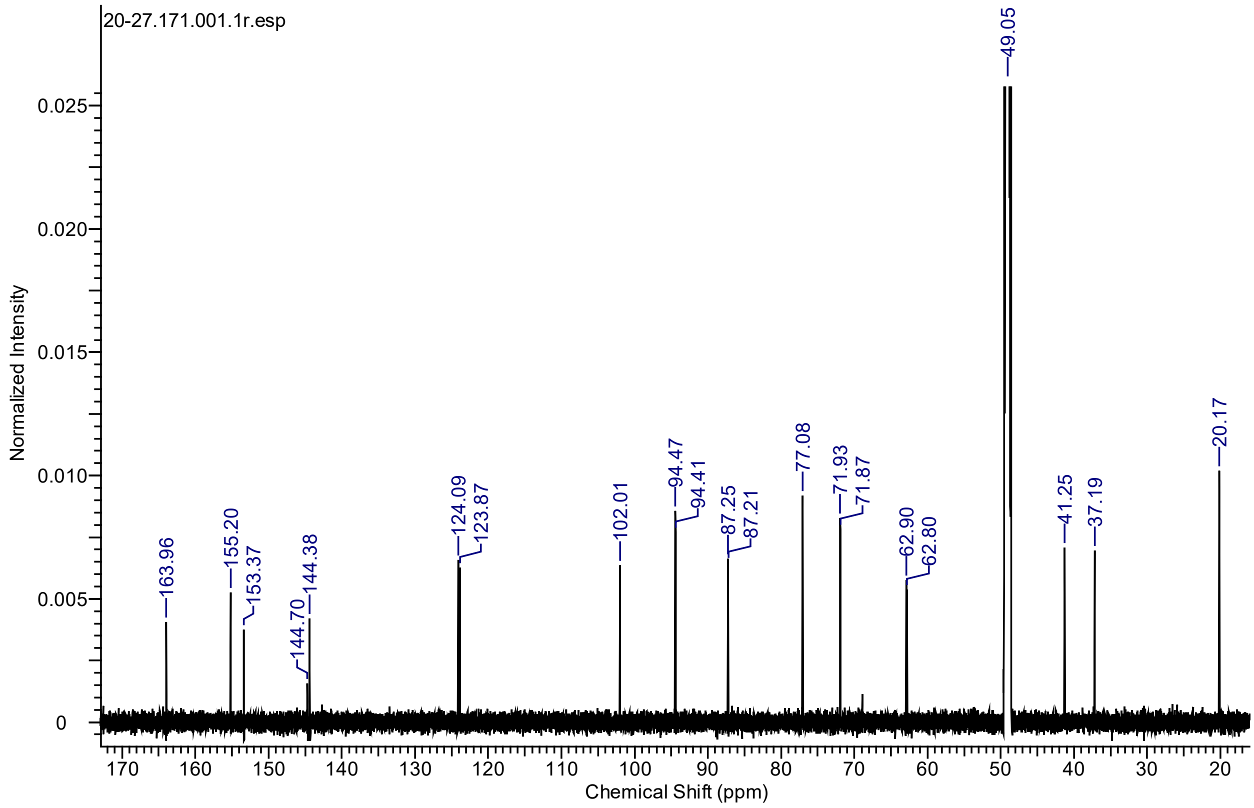

Figure S50. 1D ^{13}C-NMR spectrum of **2g** in CD$_3$OD at T = 303K.

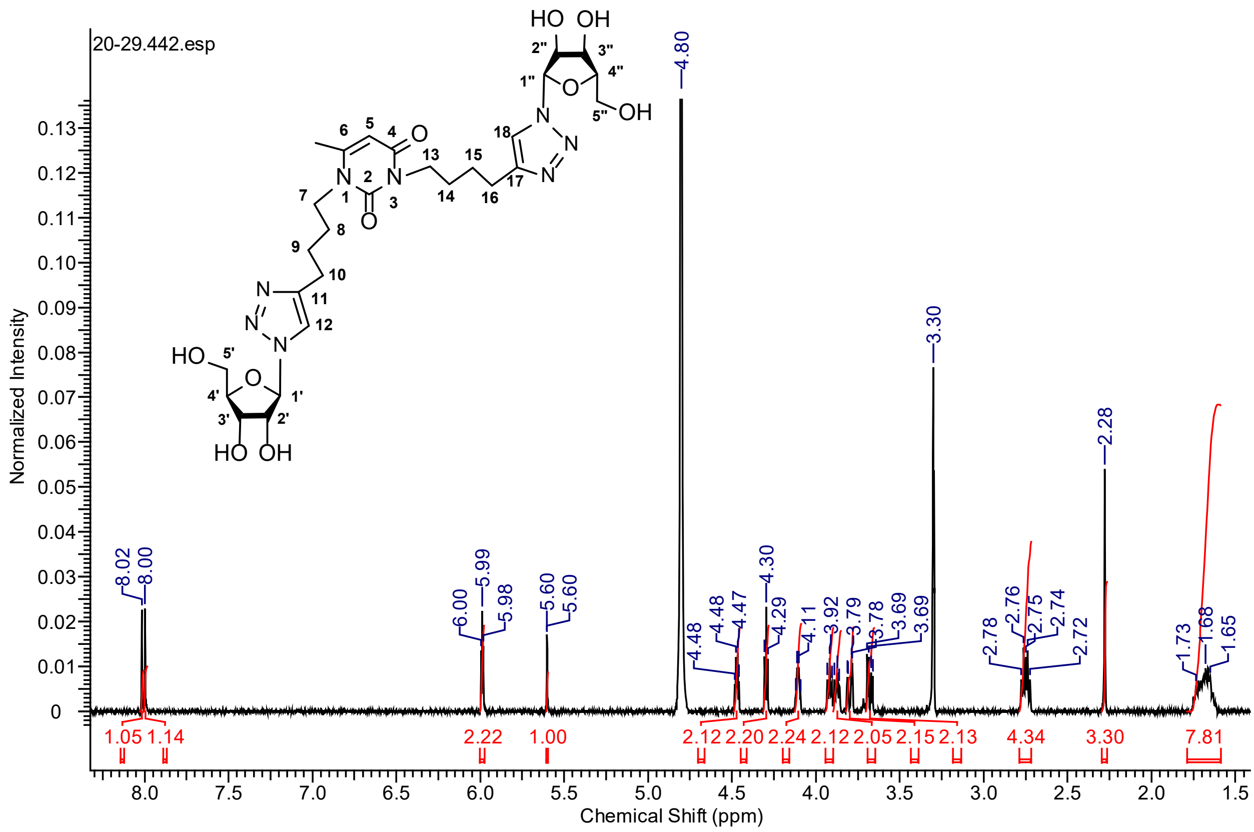

Figure S51. 1D ^{1}H-NMR spectrum of **2i** in CD_3OD at T = 303K.

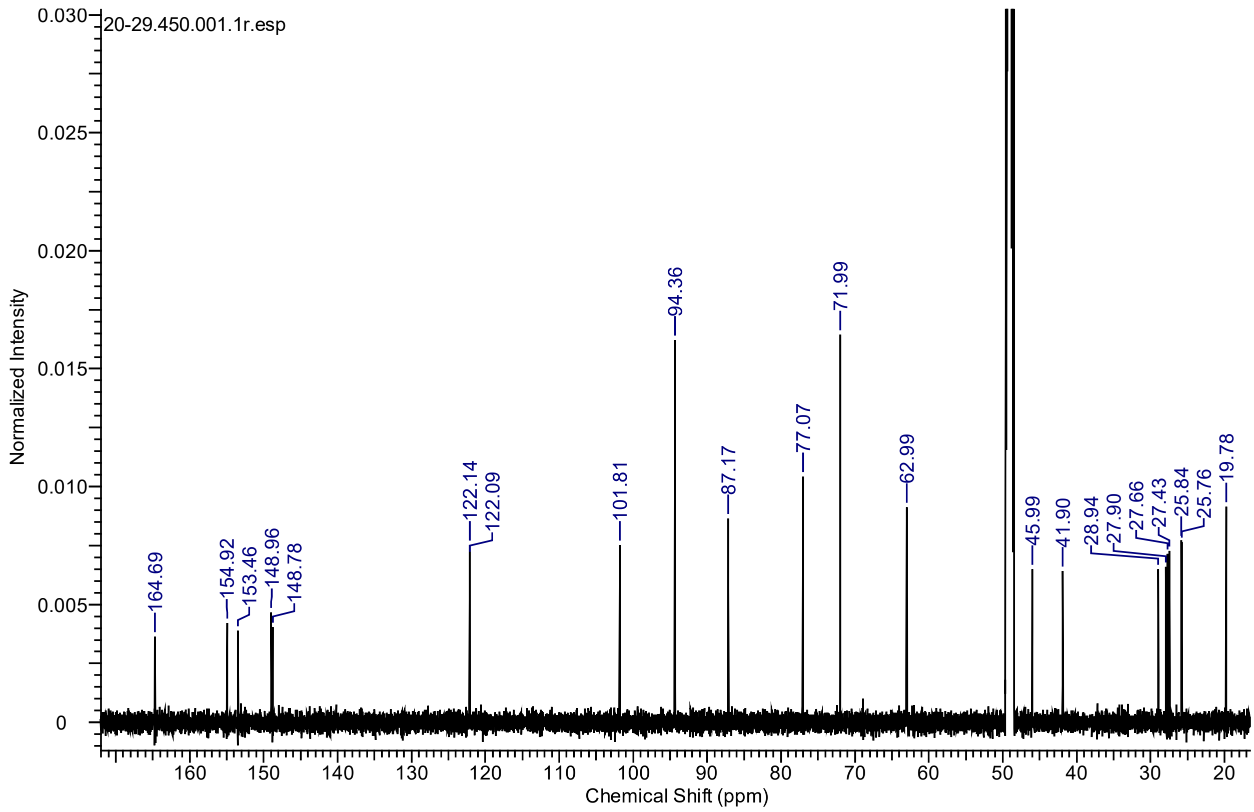

Figure S52. 1D ^{13}C-NMR spectrum of **2i** in CD_3OD at T = 303K.

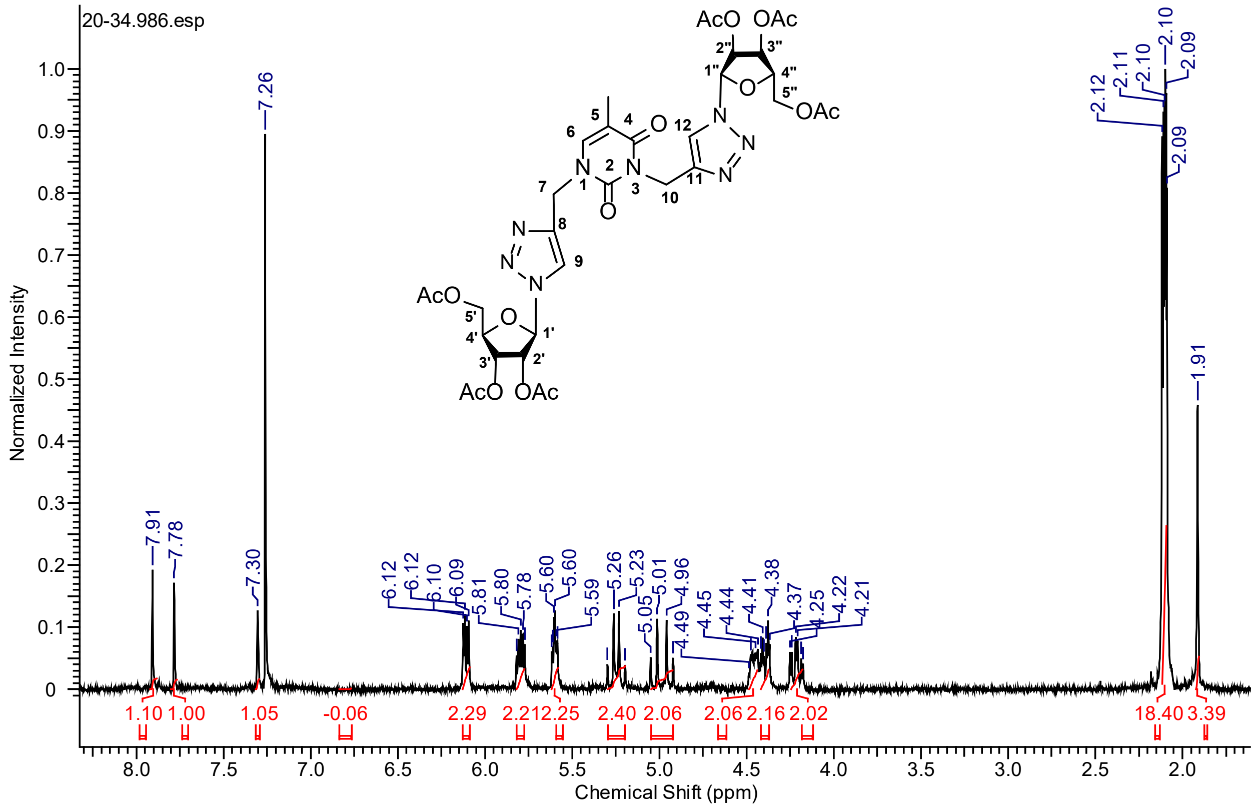

Figure S53. 1D ^{1}H-NMR spectrum of **3d** in $CDCl_3$ at T = 303K.

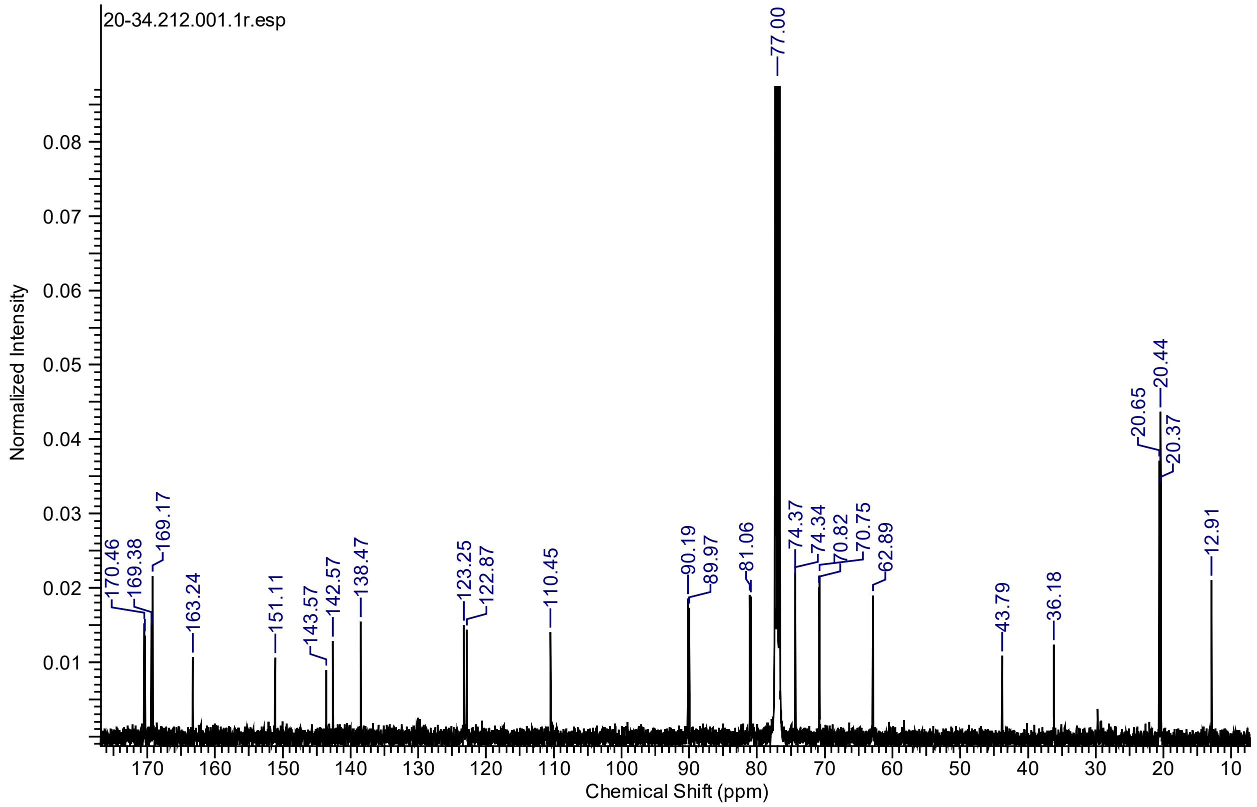

Figure S54. 1D ^{13}C-NMR spectrum of **3d** in $CDCl_3$ at T = 303K.

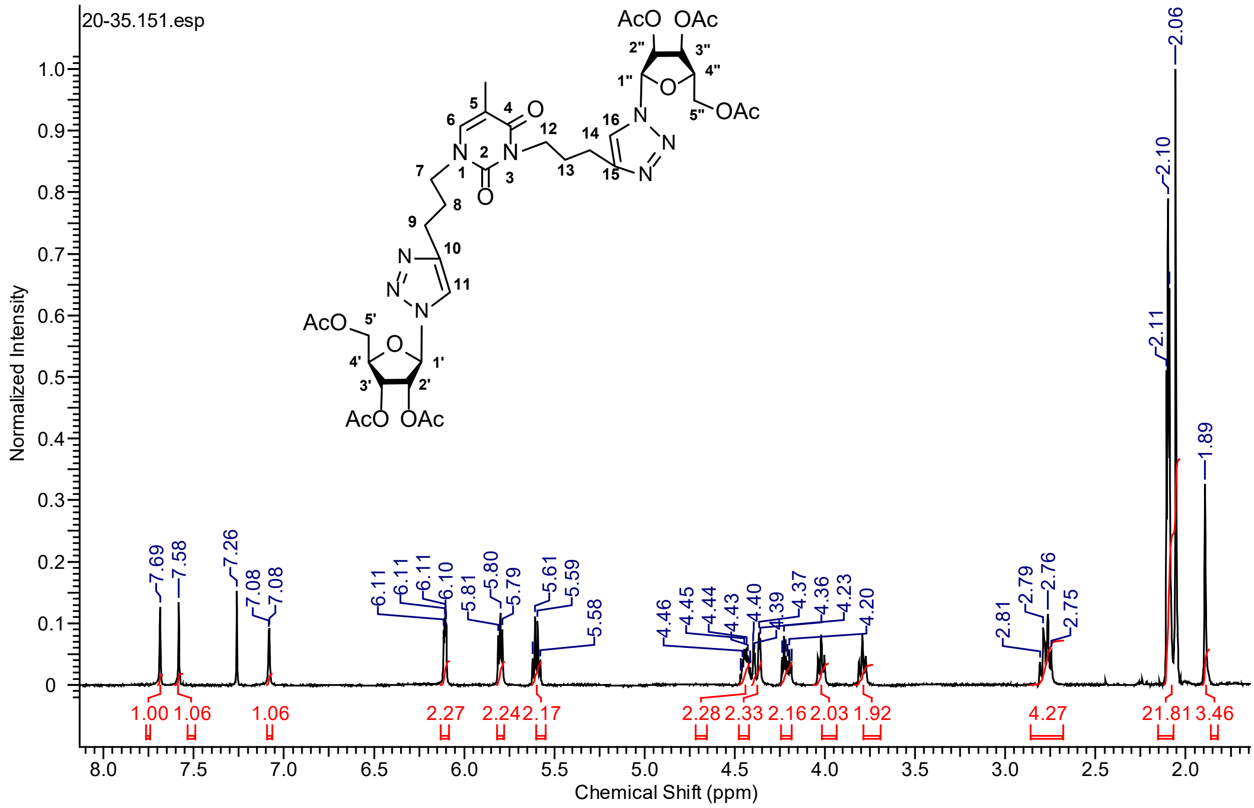

Figure S55. 1D ^{1}H-NMR spectrum of **3e** in $CDCl_3$ at T = 303K.

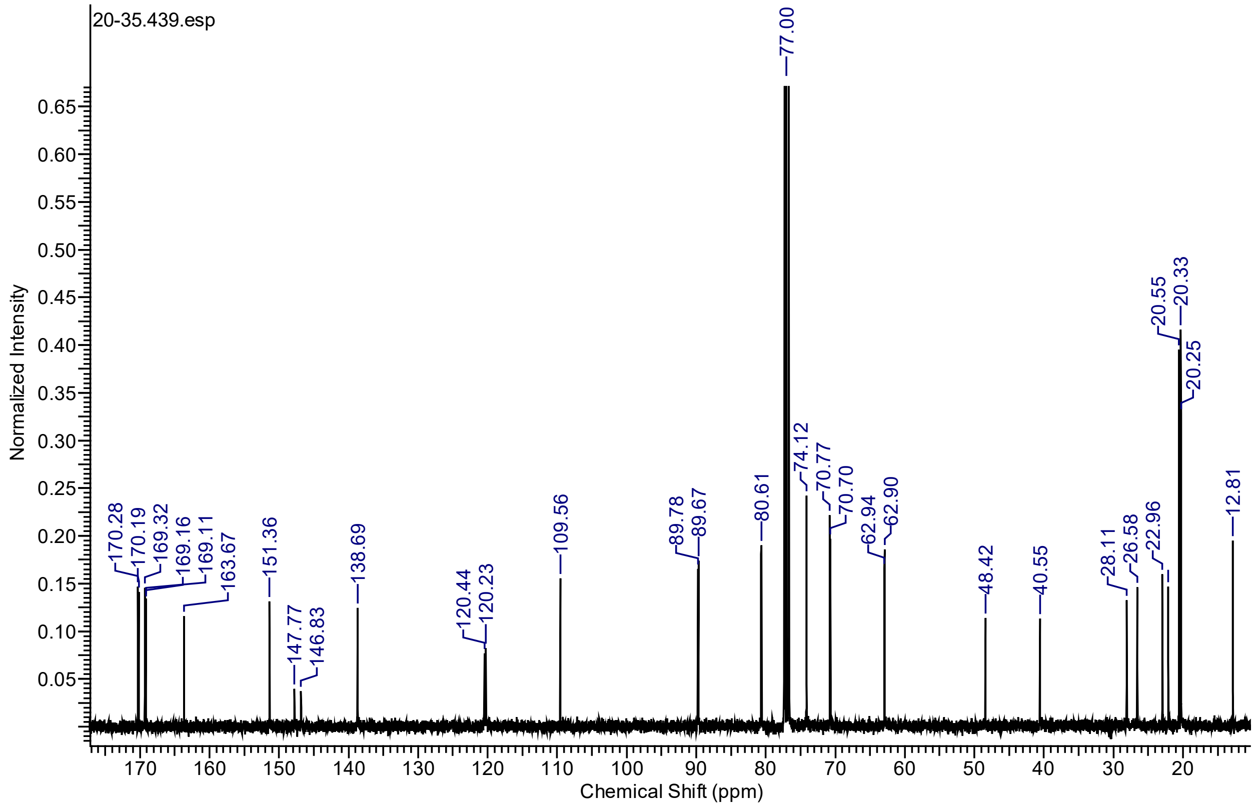

Figure 56. 1D ^{13}C-NMR spectrum of **3e** in $CDCl_3$ at T = 303K.

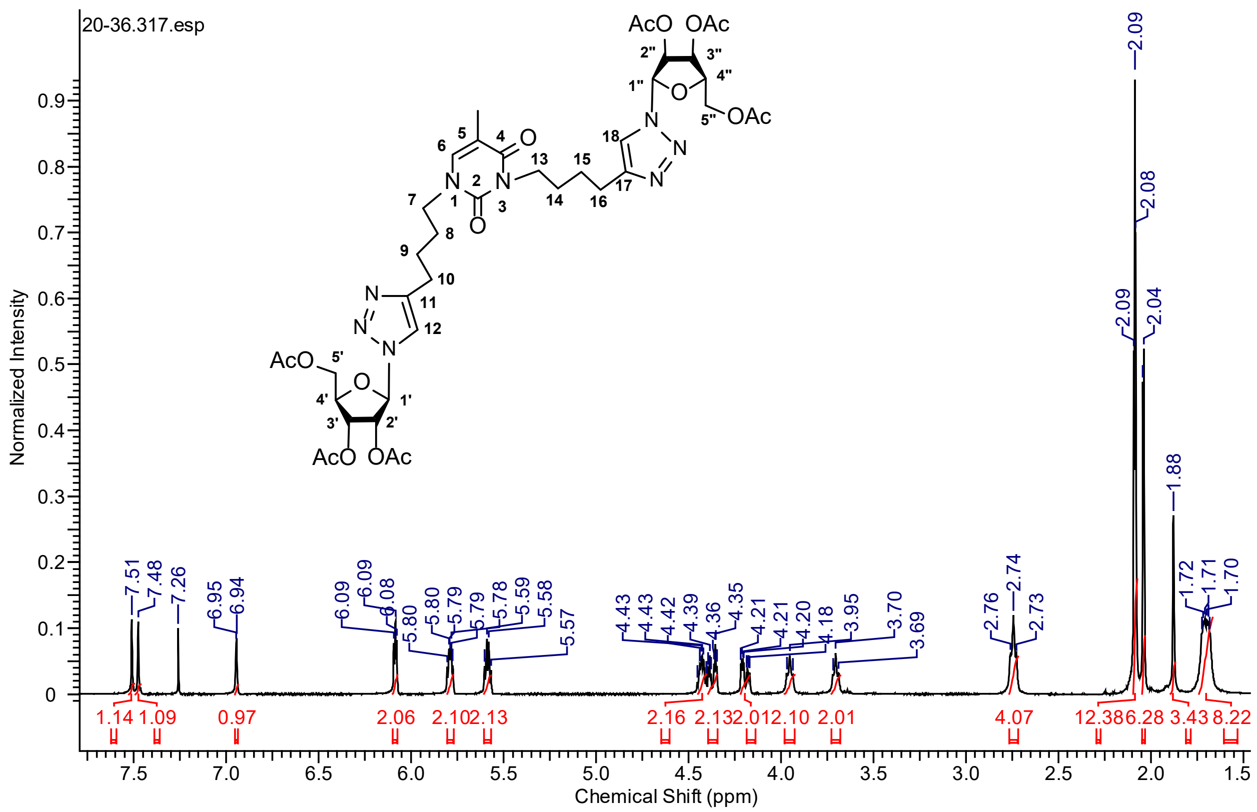

Figure S57. 1D ^{1}H-NMR spectrum of **3f** in $CDCl_3$ at T = 303K.

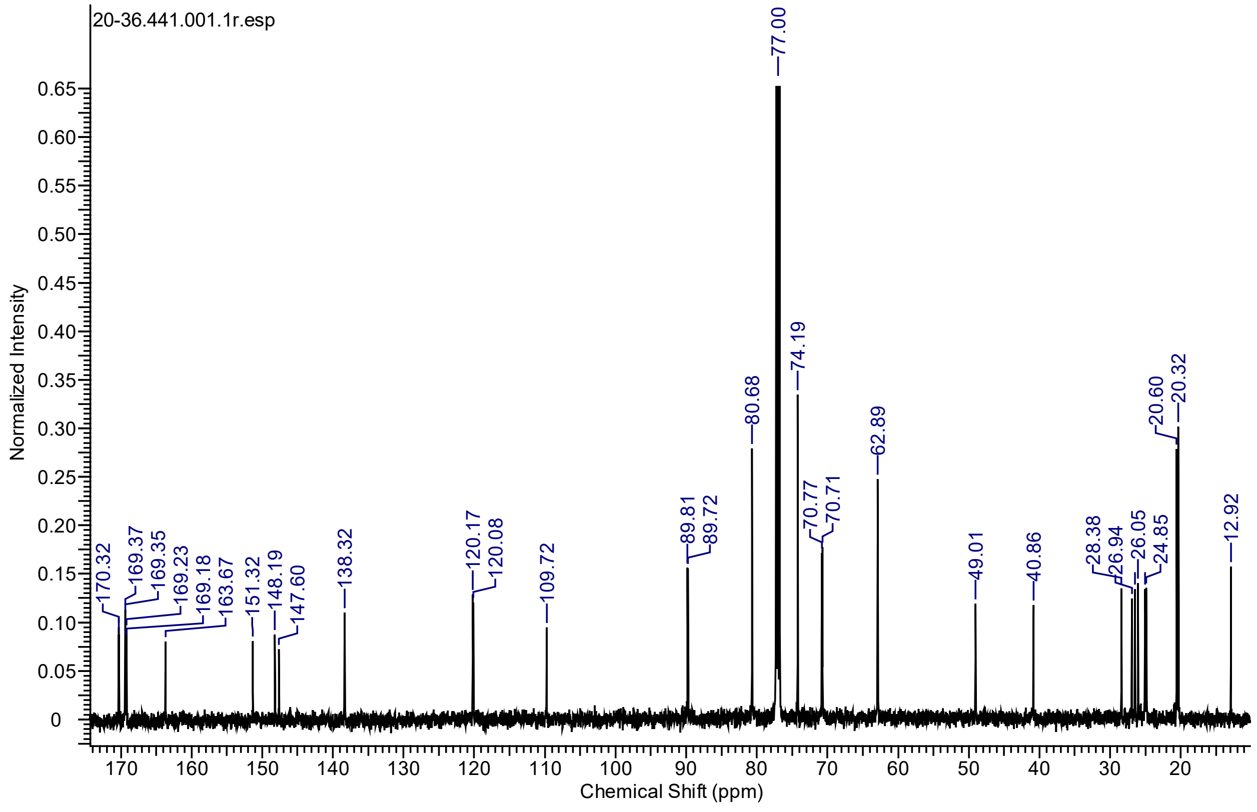

Figure S58. 1D ^{13}C-NMR spectrum of **3f** in $CDCl_3$ at T = 303K.

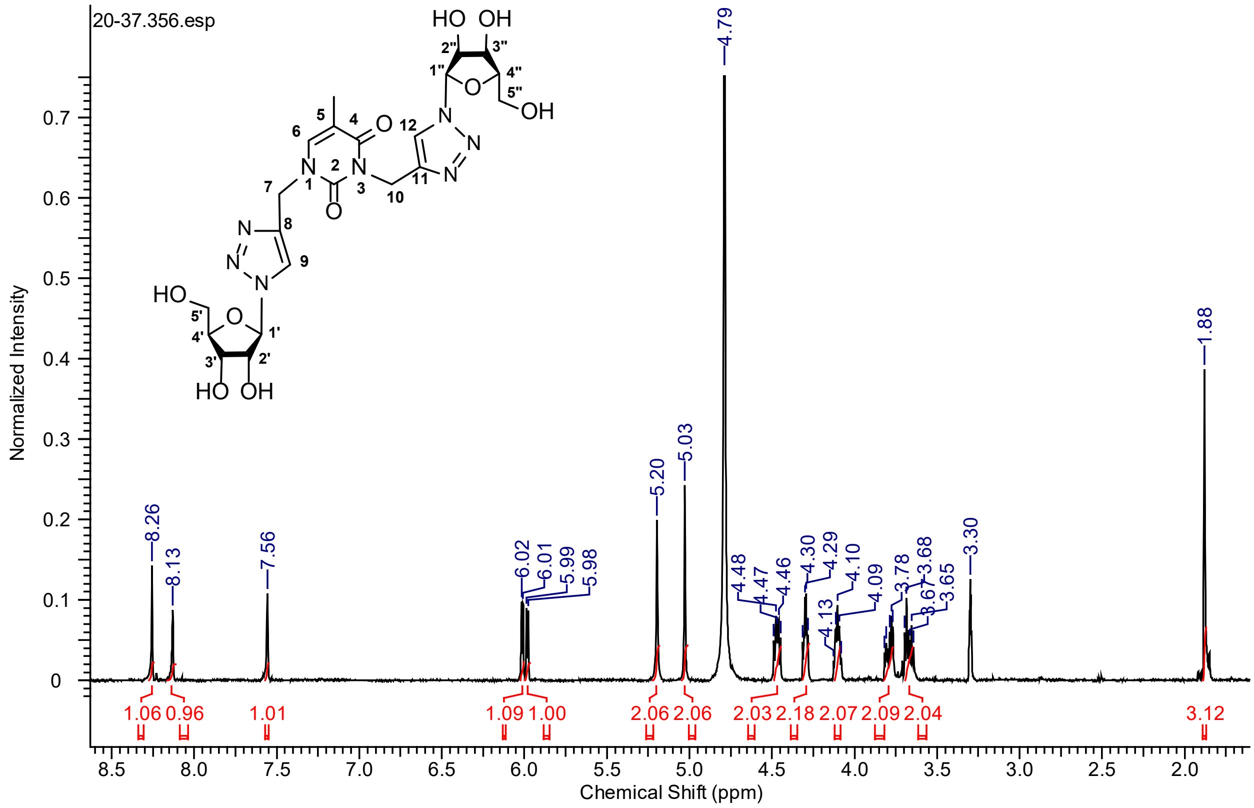

Figure S59. 1D ^{1}H-NMR spectrum of **3g** in CD_3OD at T = 303K.

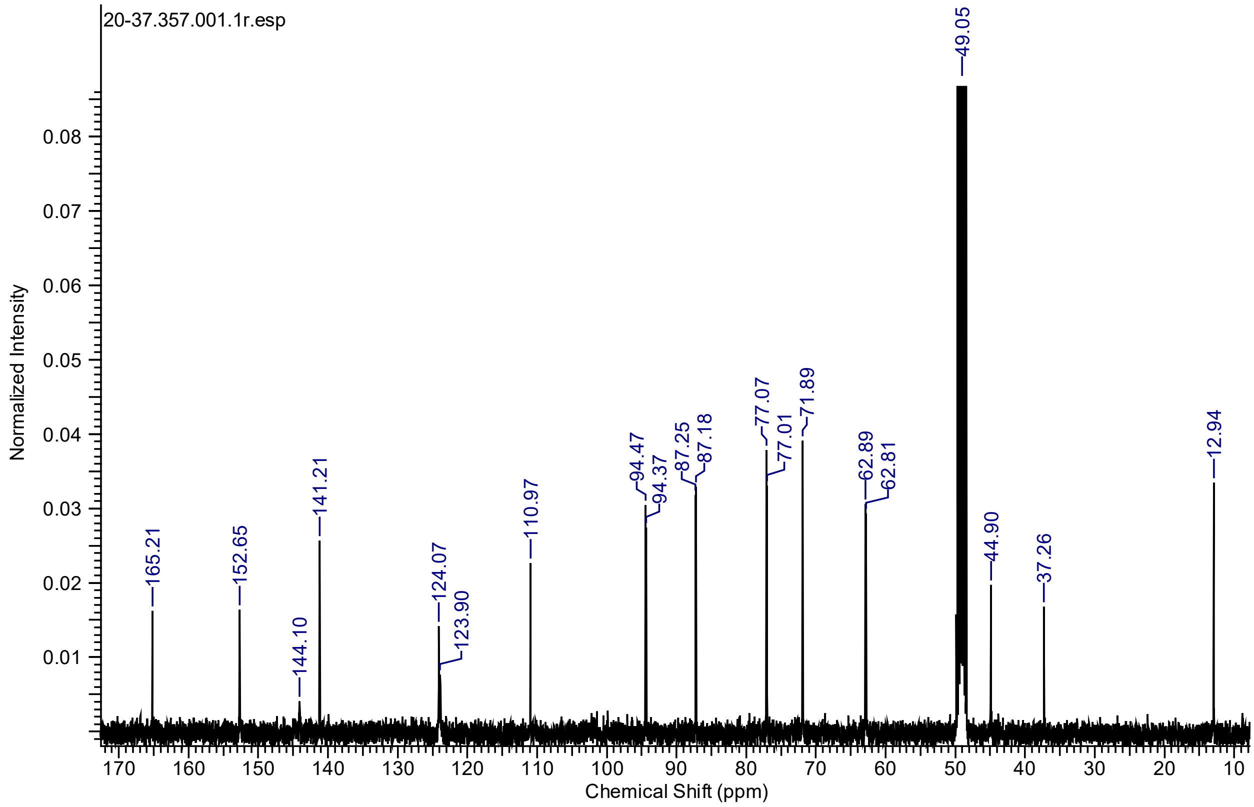

Figure S60. 1D ^{13}C-NMR spectrum of **3g** in CD_3OD at T = 303K.

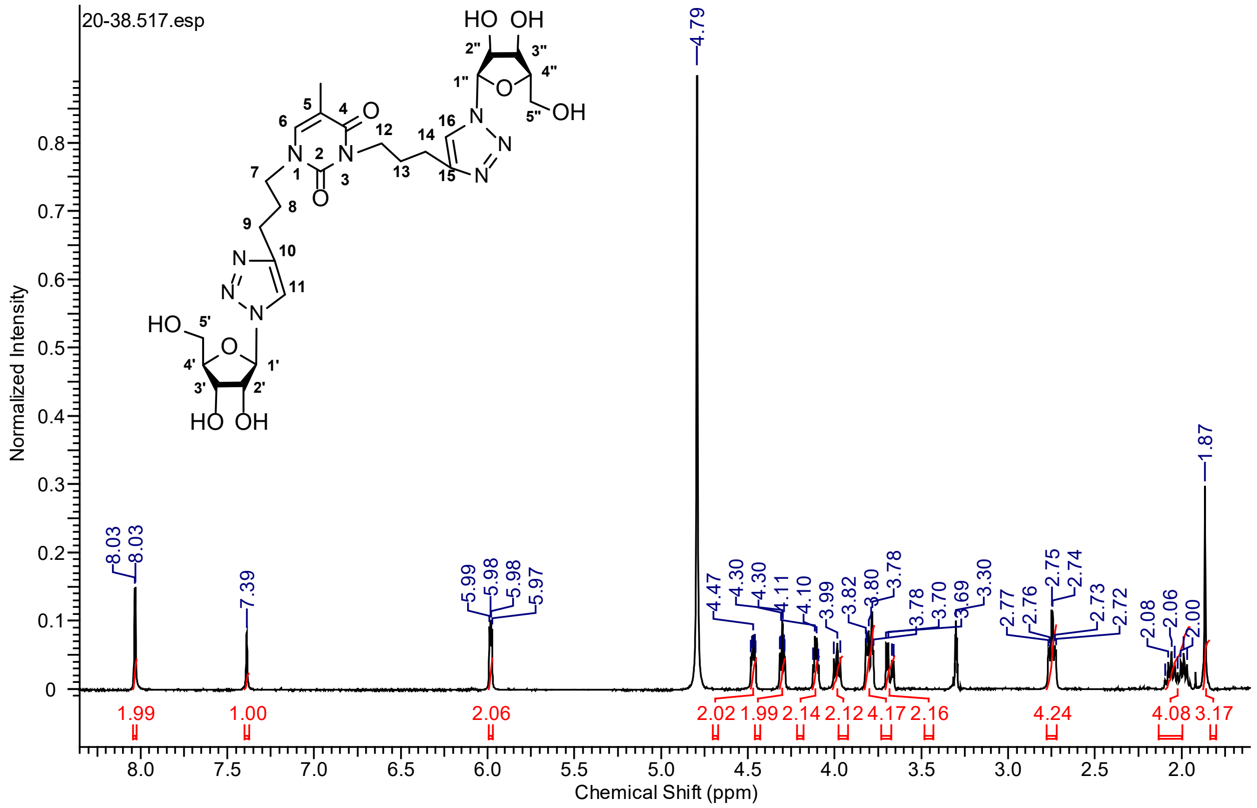

Figure S61. 1D ^{1}H-NMR spectrum of **3h** in CD_3OD at T = 303K.

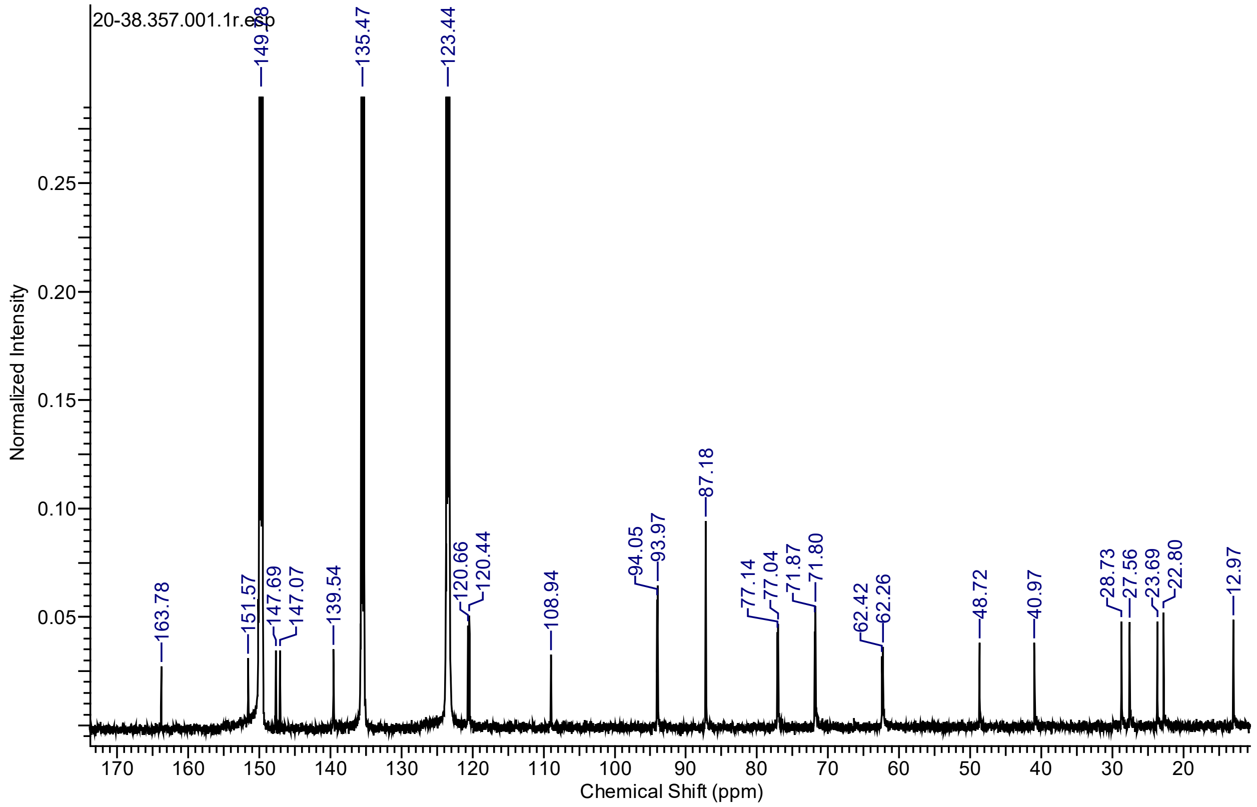

Figure S62. 1D ^{13}C-NMR spectrum of **3h** in CD_3OD at T = 303K.

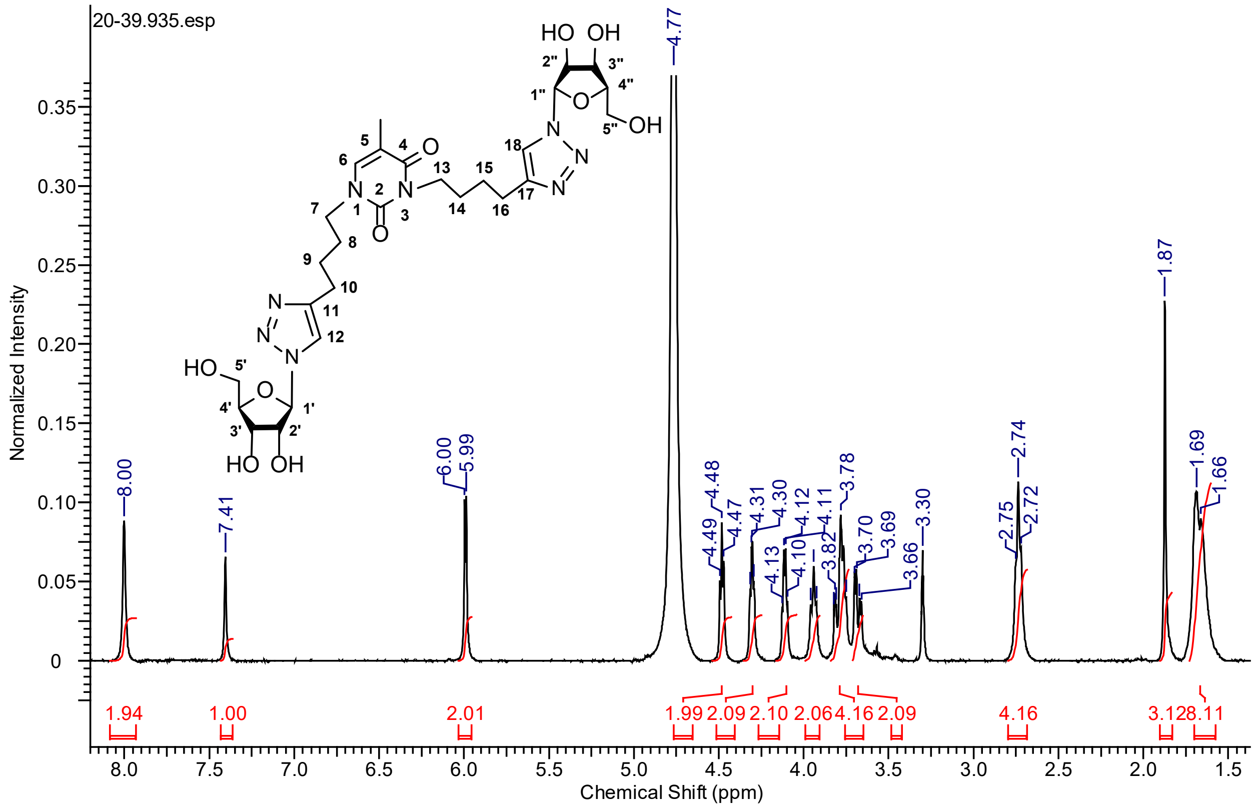

Figure S63. 1D ^{1}H-NMR spectrum of **3i** in CD_3OD at T = 303K.

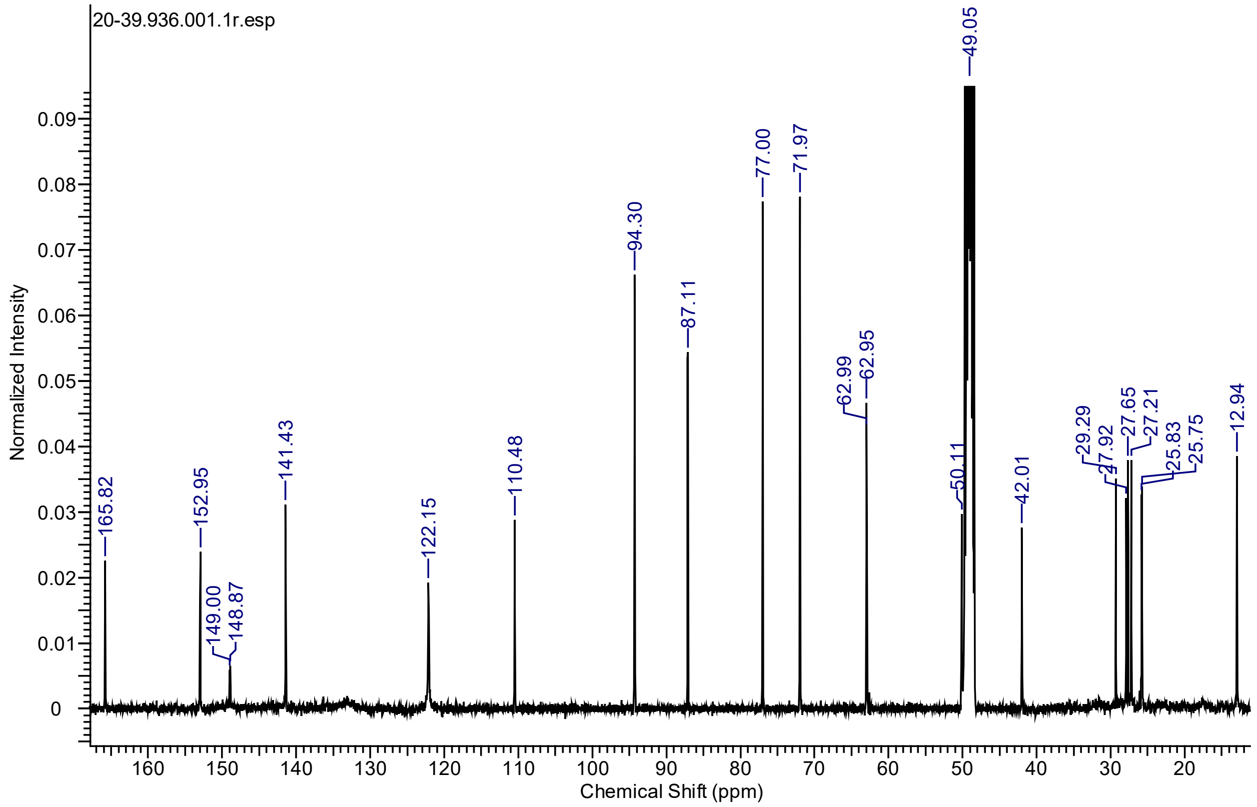

Figure S64. 1D ^{13}C-NMR spectrum of **3i** in CD_3OD at T = 303K.

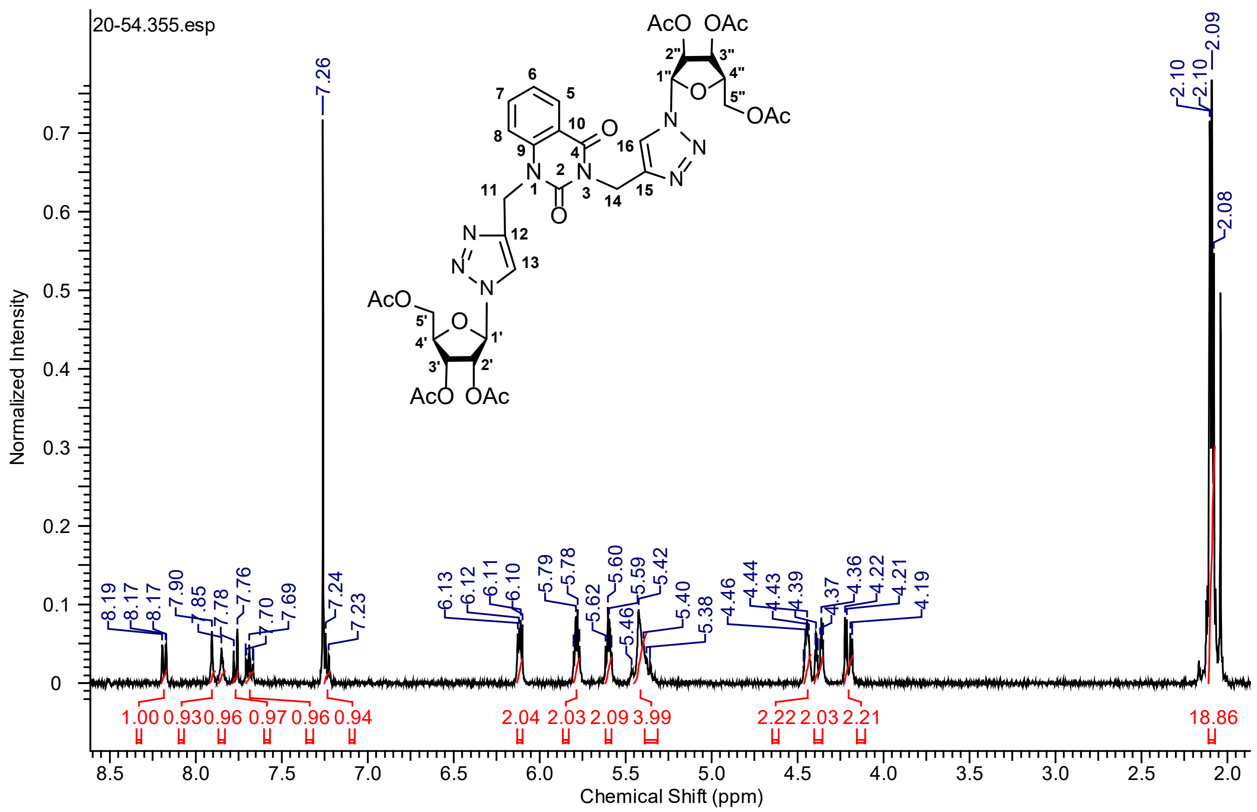

Figure S65. 1D ^{1}H-NMR spectrum of **4d** in $CDCl_3$ at T = 303K.

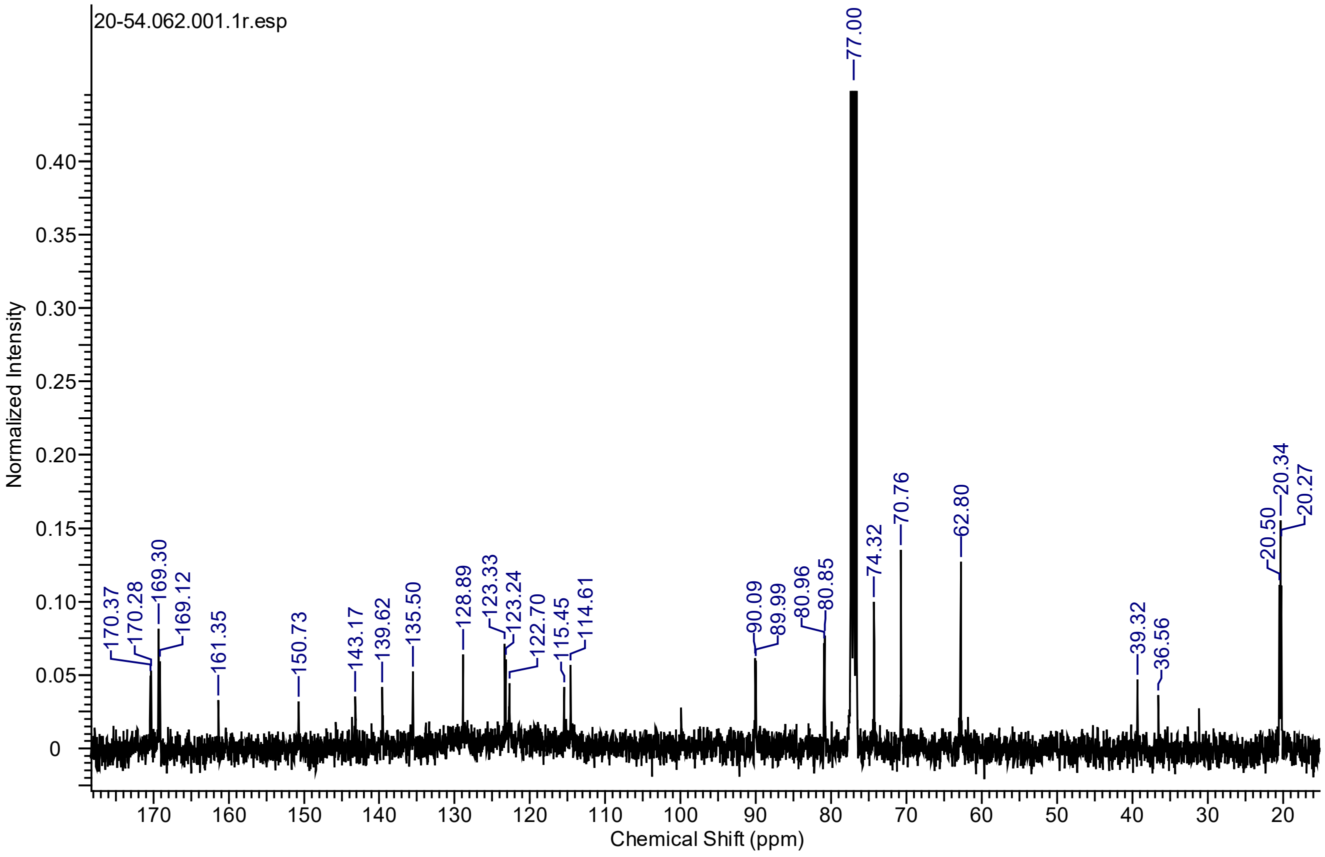

Figure S66. 1D ^{13}C-NMR spectrum of **4d** in $CDCl_3$ at T = 303K.

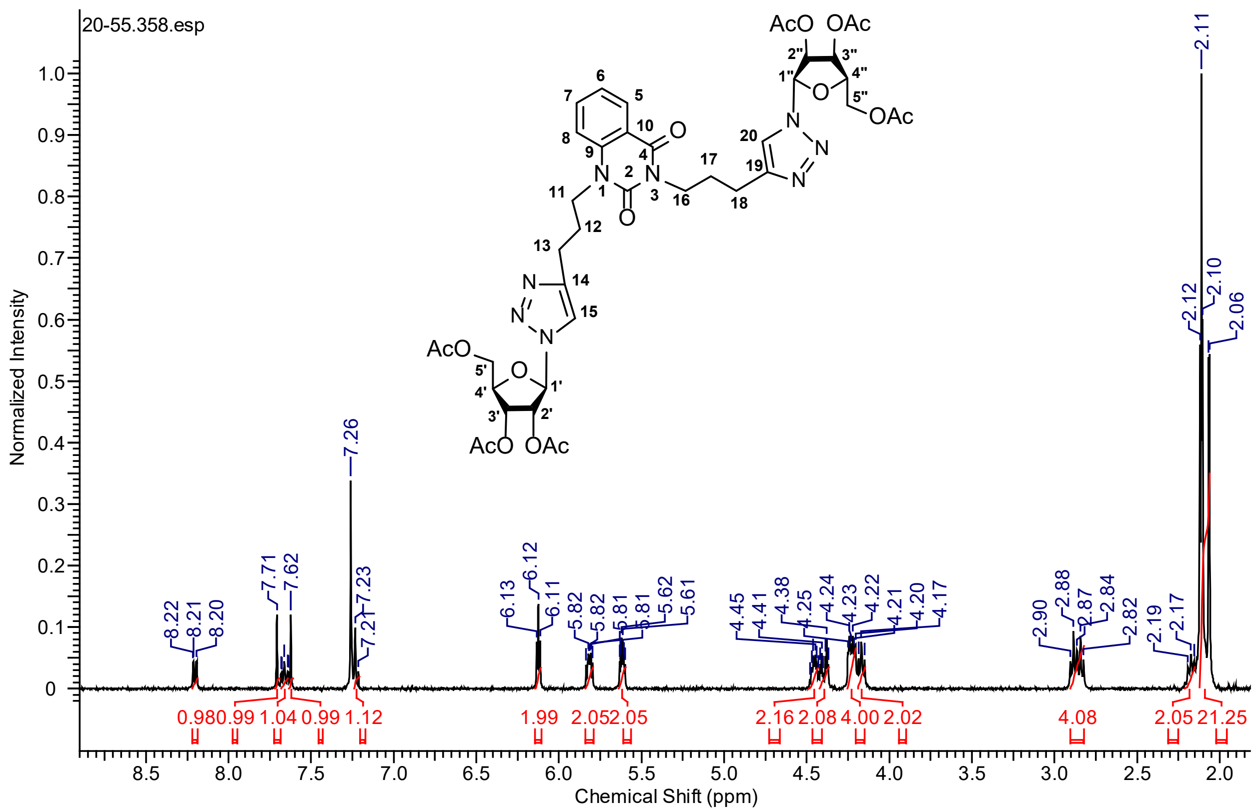

Figure S67. 1D ^{1}H*NMR spectrum of **4e** in $CDCl_3$ at T = 303K.

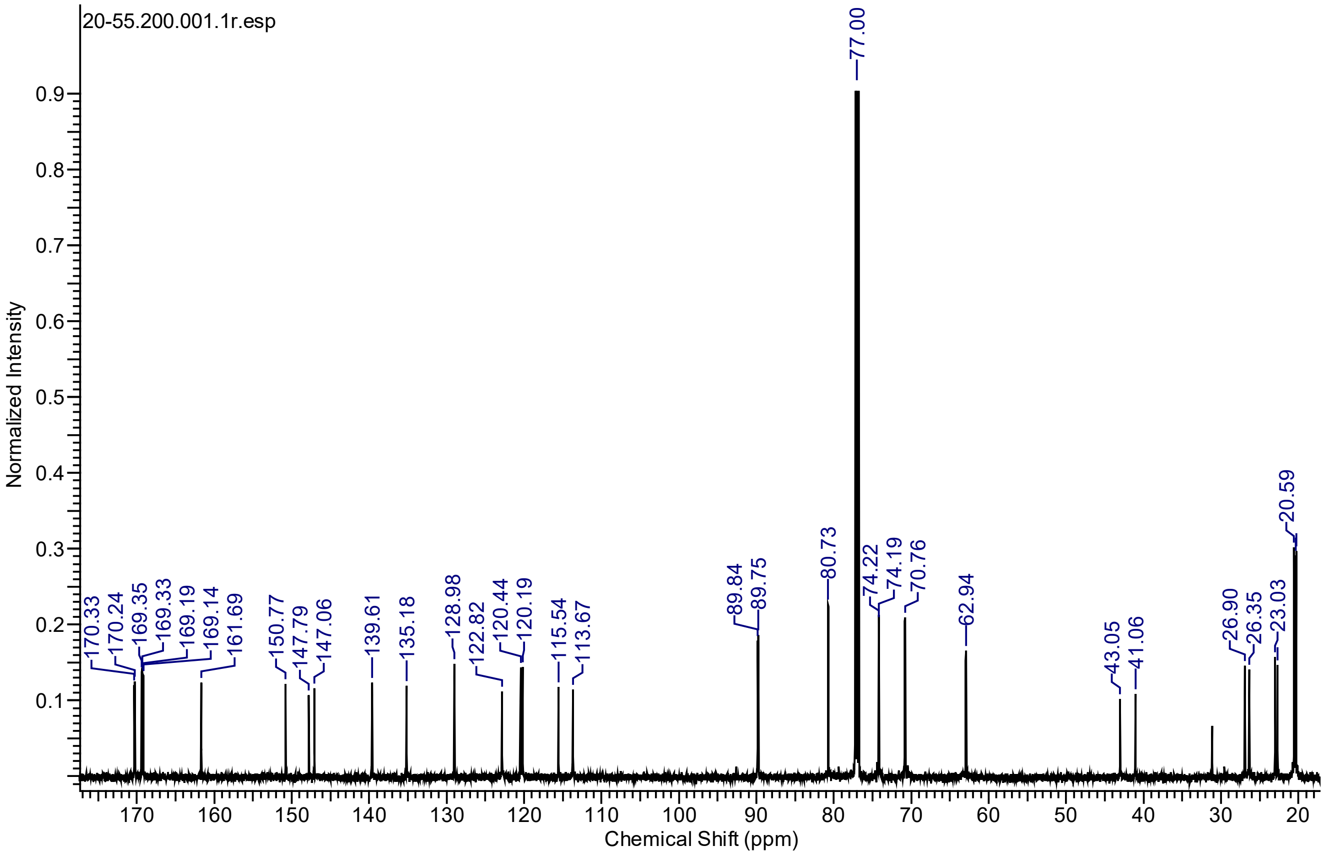

Figure S68. 1D ^{13}C-NMR spectrum of **4e** in $CDCl_3$ at T = 303K.

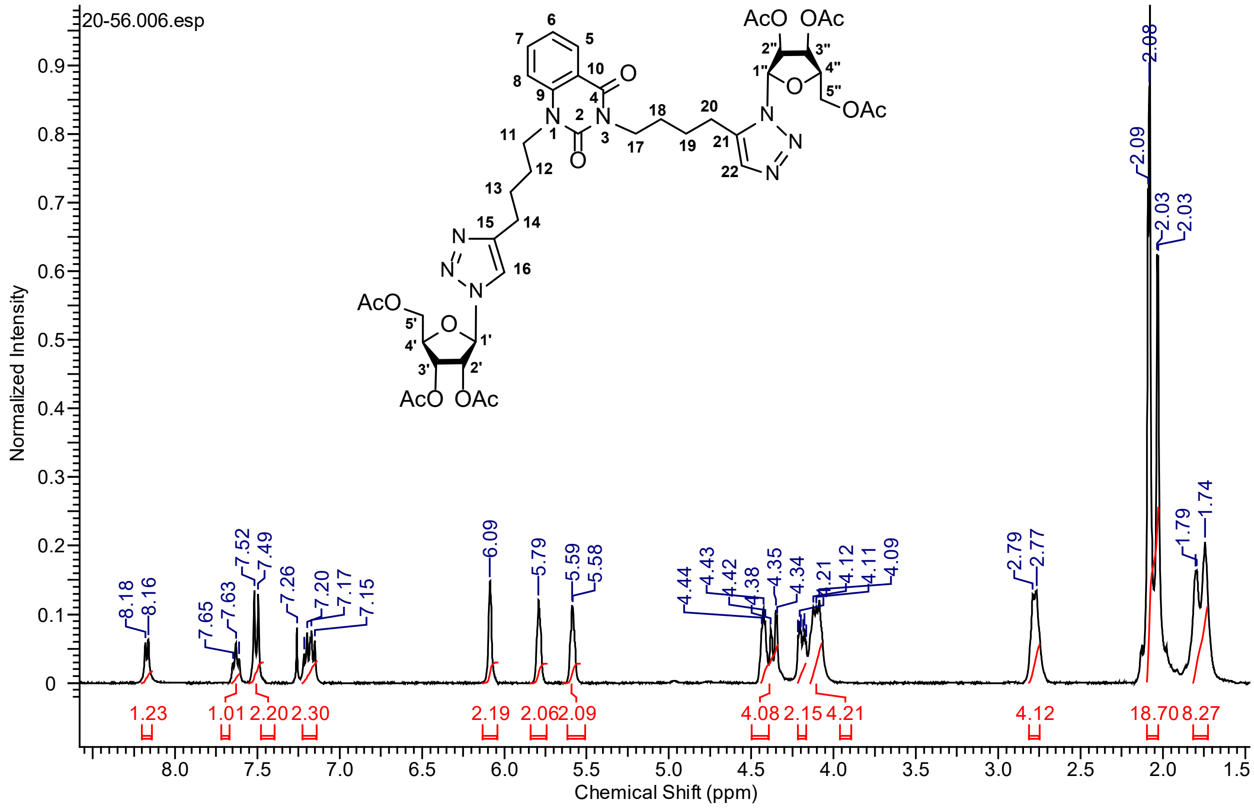

Figure 69. 1D ^{1}H-NMR spectrum of **4f** in $CDCl_3$ at T = 303K.

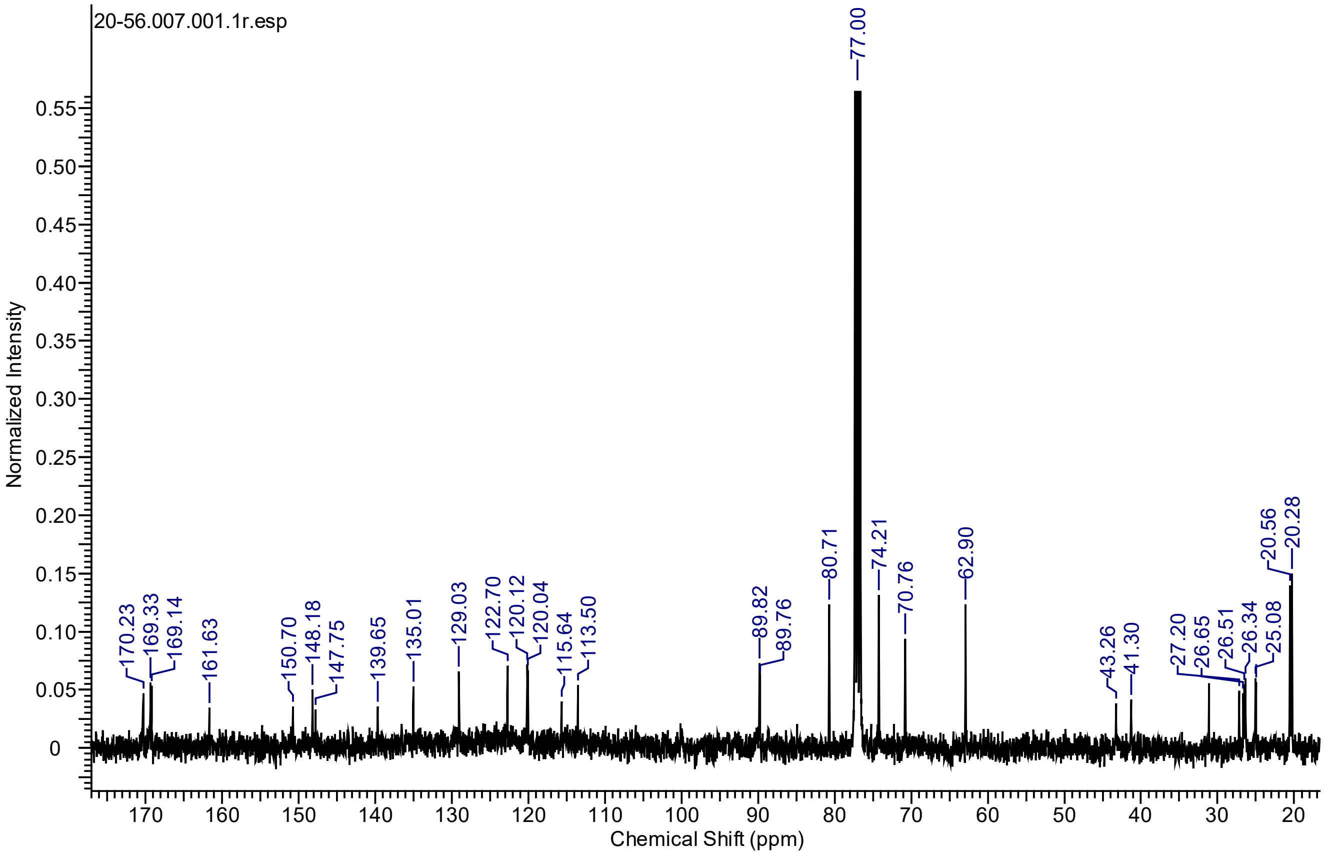

Figure S70. 1D ^{1}H-NMR spectrum of **4f** in $CDCl_3$ at T = 303K.

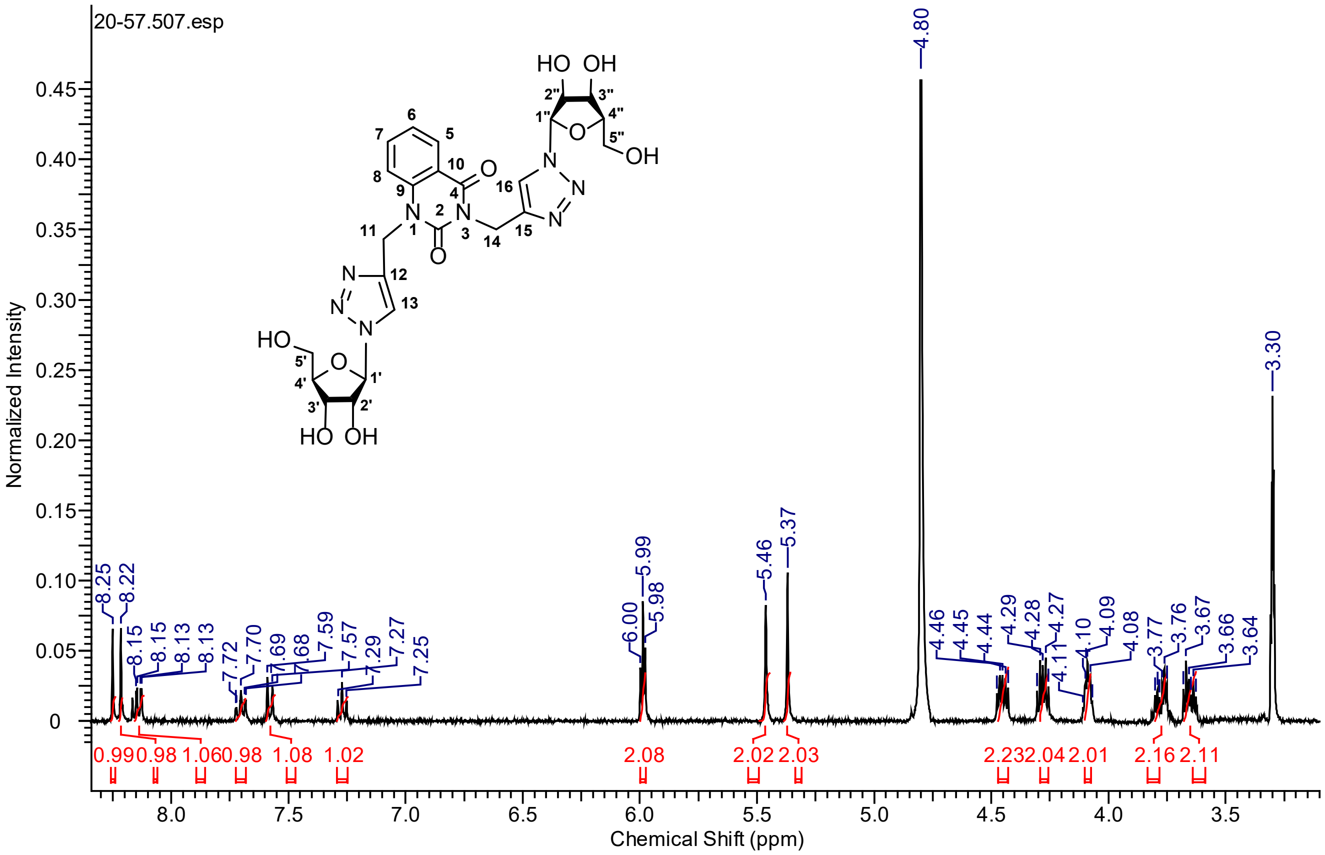

Figure S71. 1D ^{1}H-NMR spectrum of **4g** in CD_3OD at T = 303K.

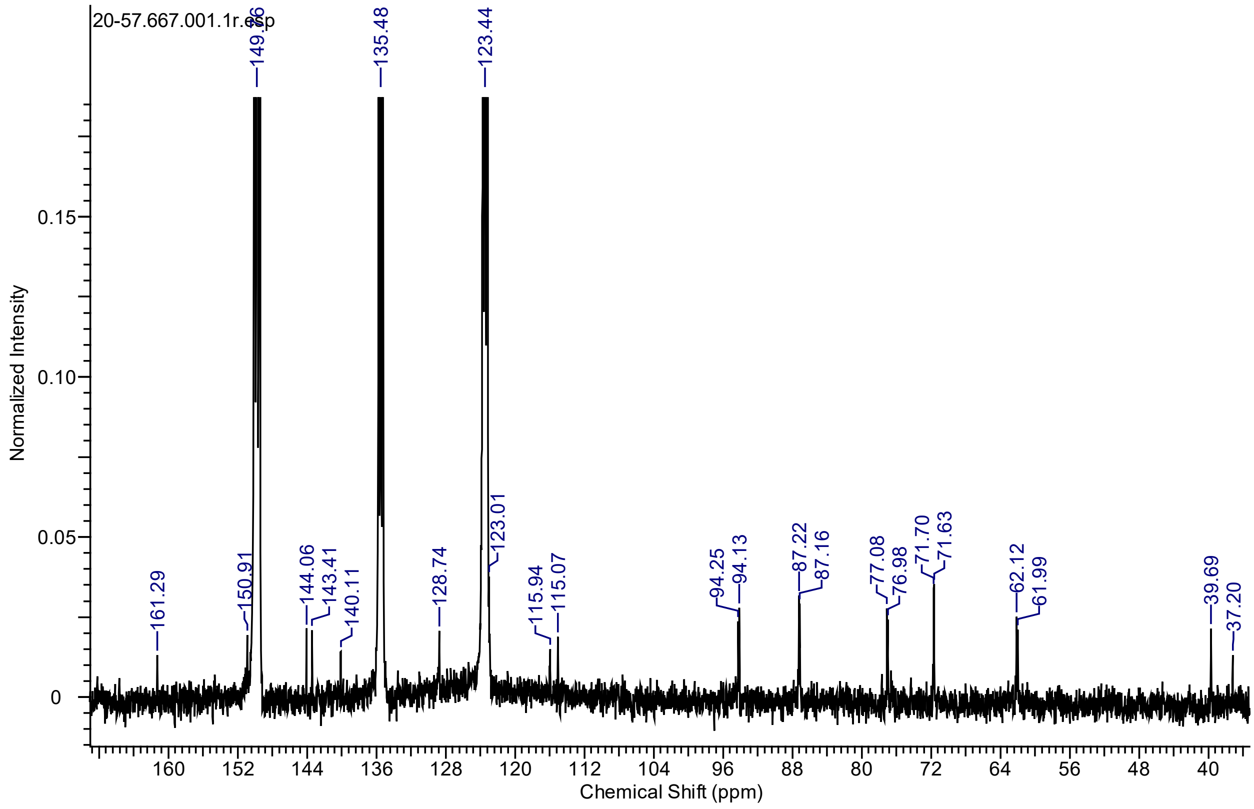

Figure S72. 1D ^{13}C-NMR spectrum of **4g** in pyridine-d_5 at T = 303K.

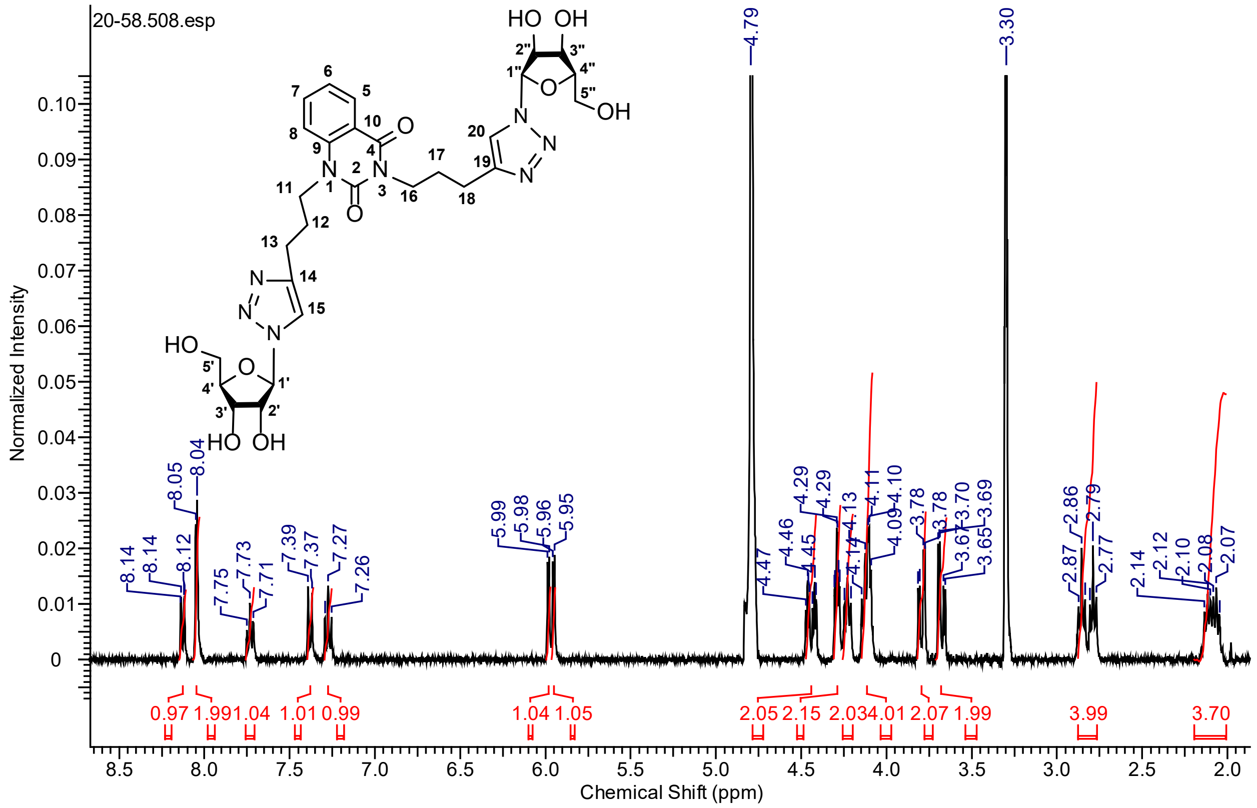

Figure S73. 1D ^{1}H-NMR spectrum of **4h** in CD_3OD at T = 303K.

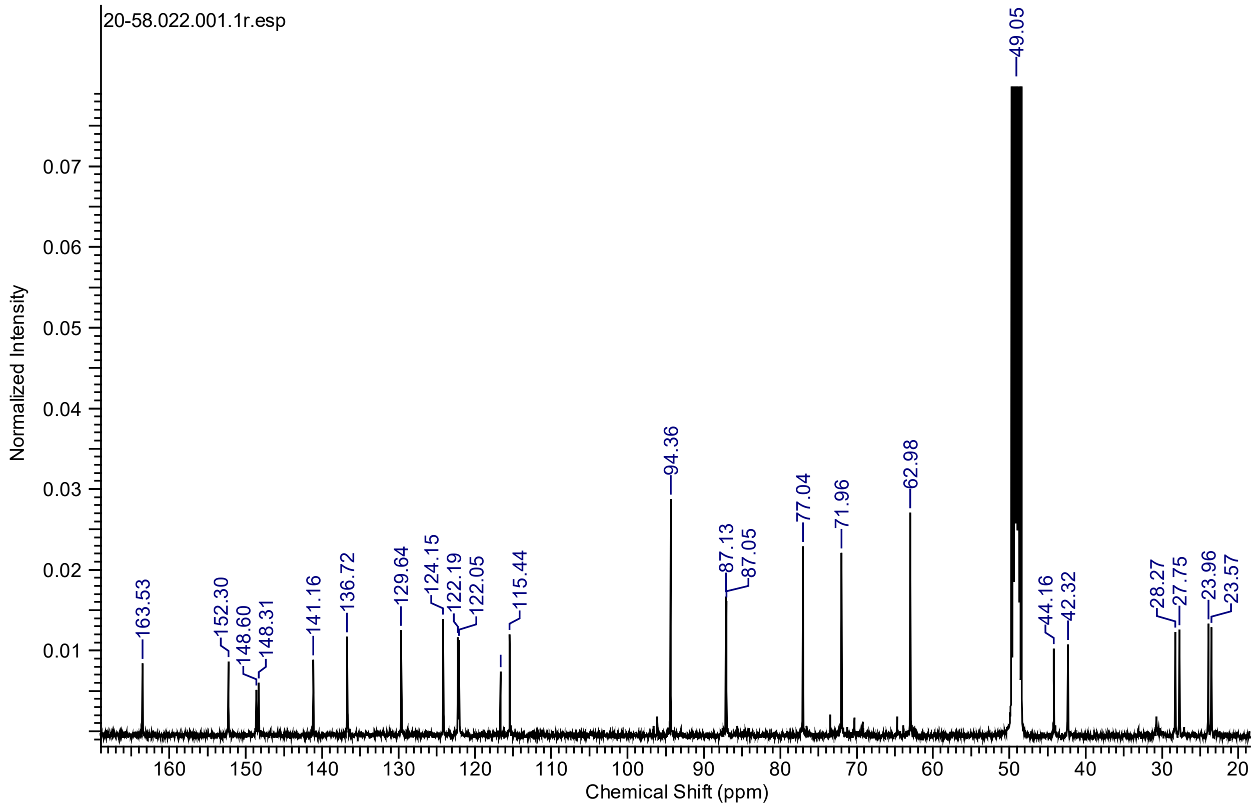

Figure S74. 1D ^{13}C-NMR spectrum of **4h** in CD_3OD at T = 303K.

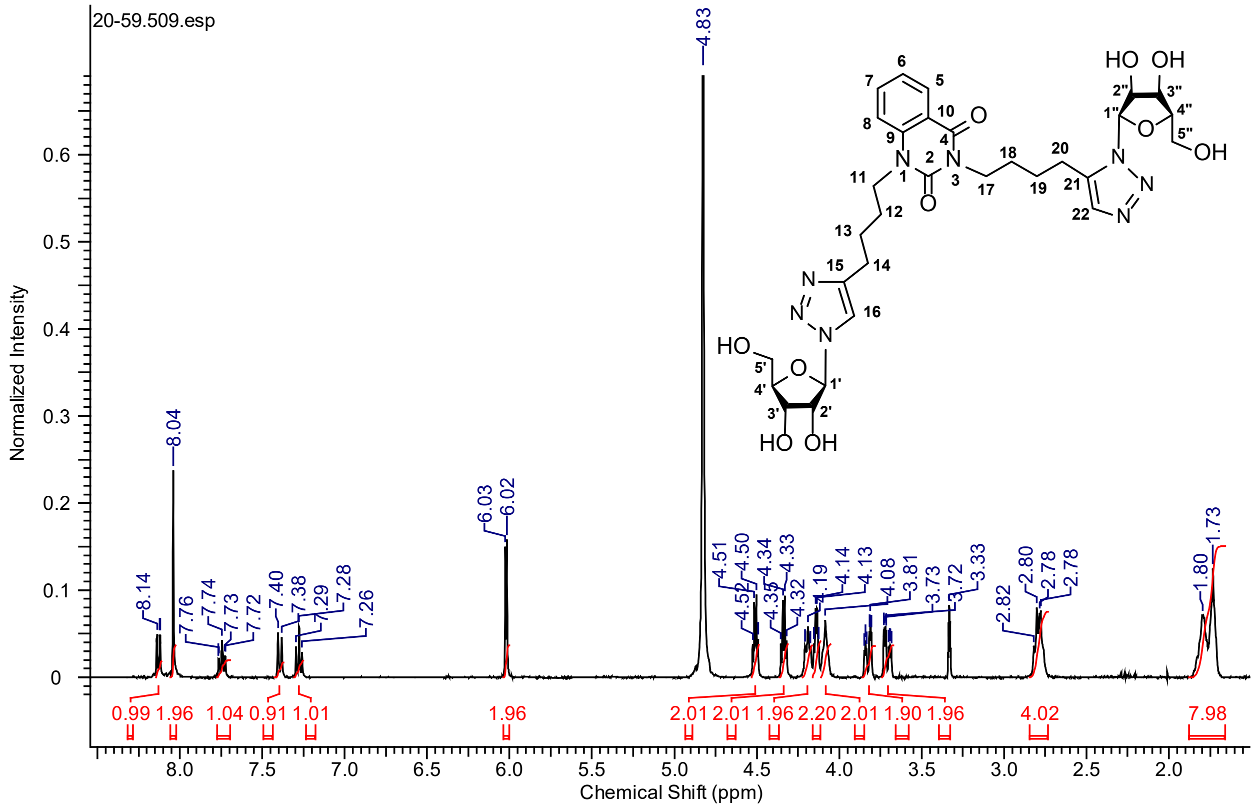

Figure S75. 1D ^{1}H-NMR spectrum of **4i** in CD_3OD at T = 303K.

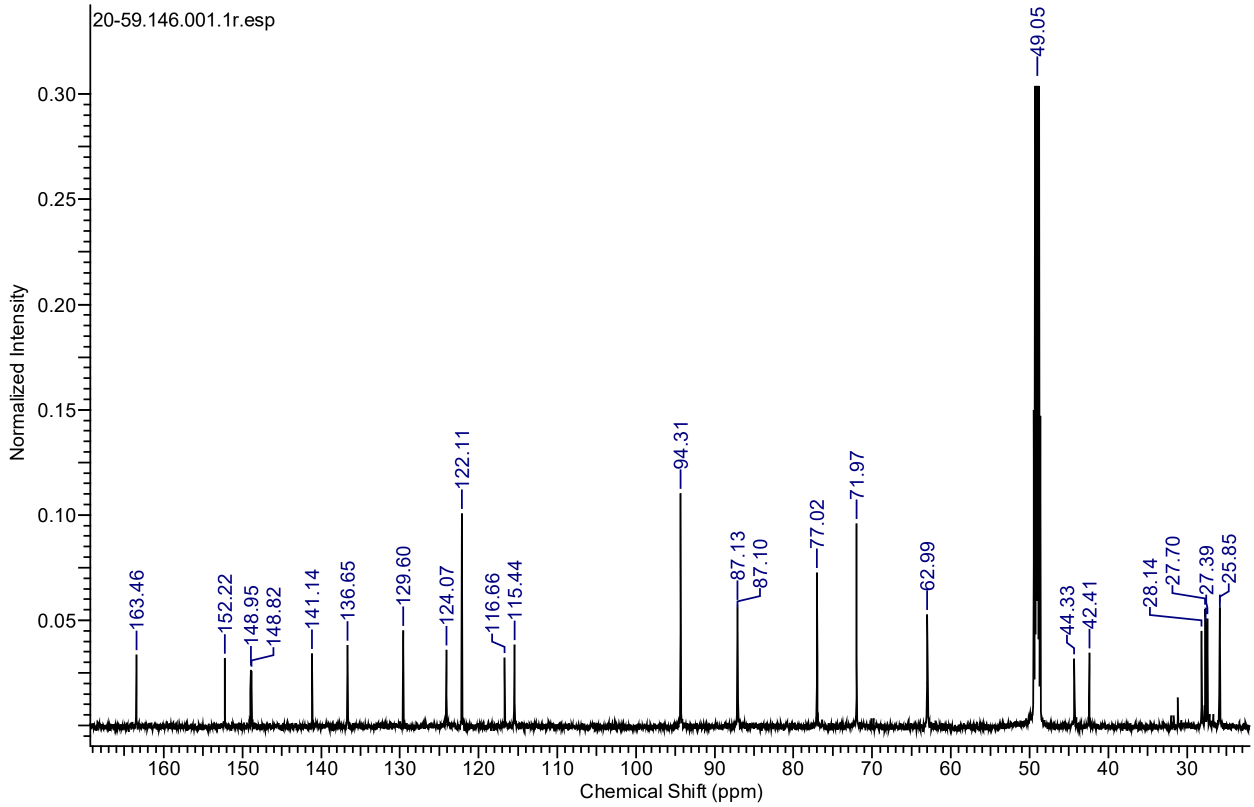

Figure S76. 1D ^{13}C-NMR spectrum of **4i** in CD_3OD at T = 303K.

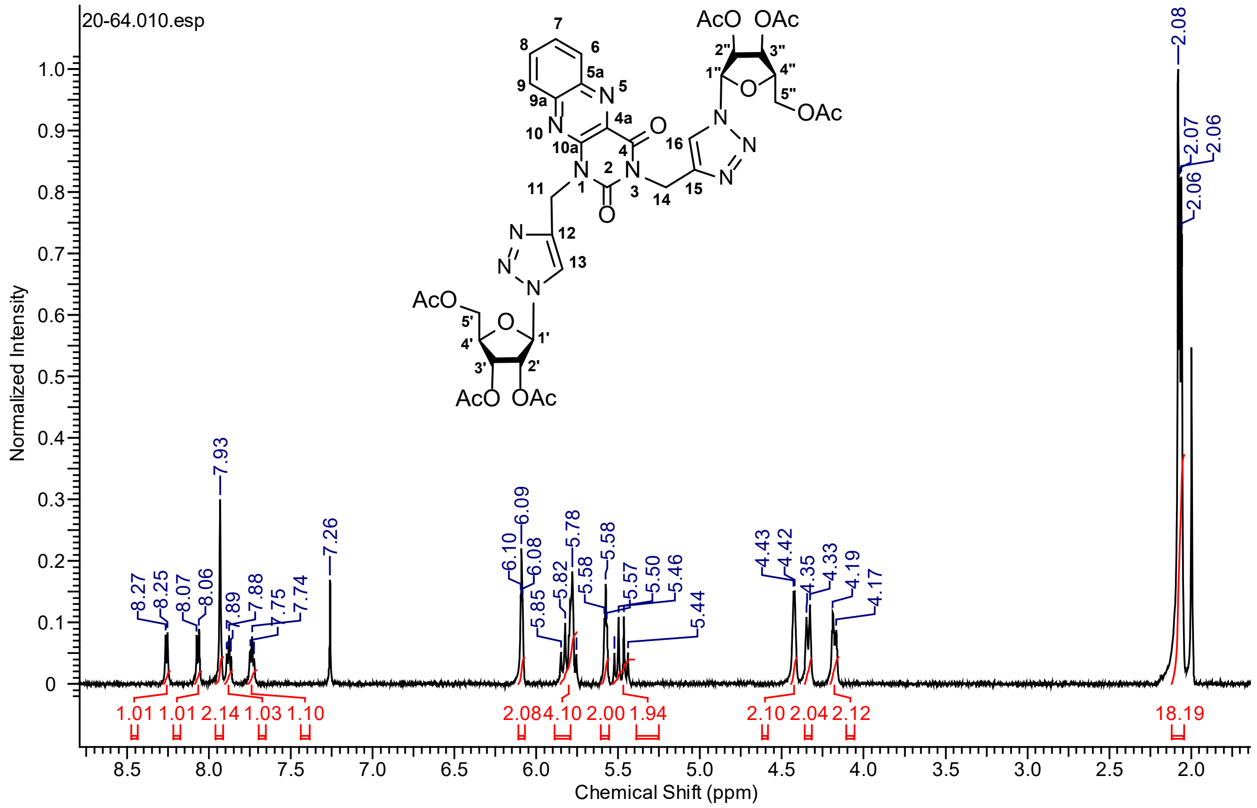

Figure S77. 1D ^{1}H-NMR spectrum of **5d** in $CDCl_3$ at T = 303K.

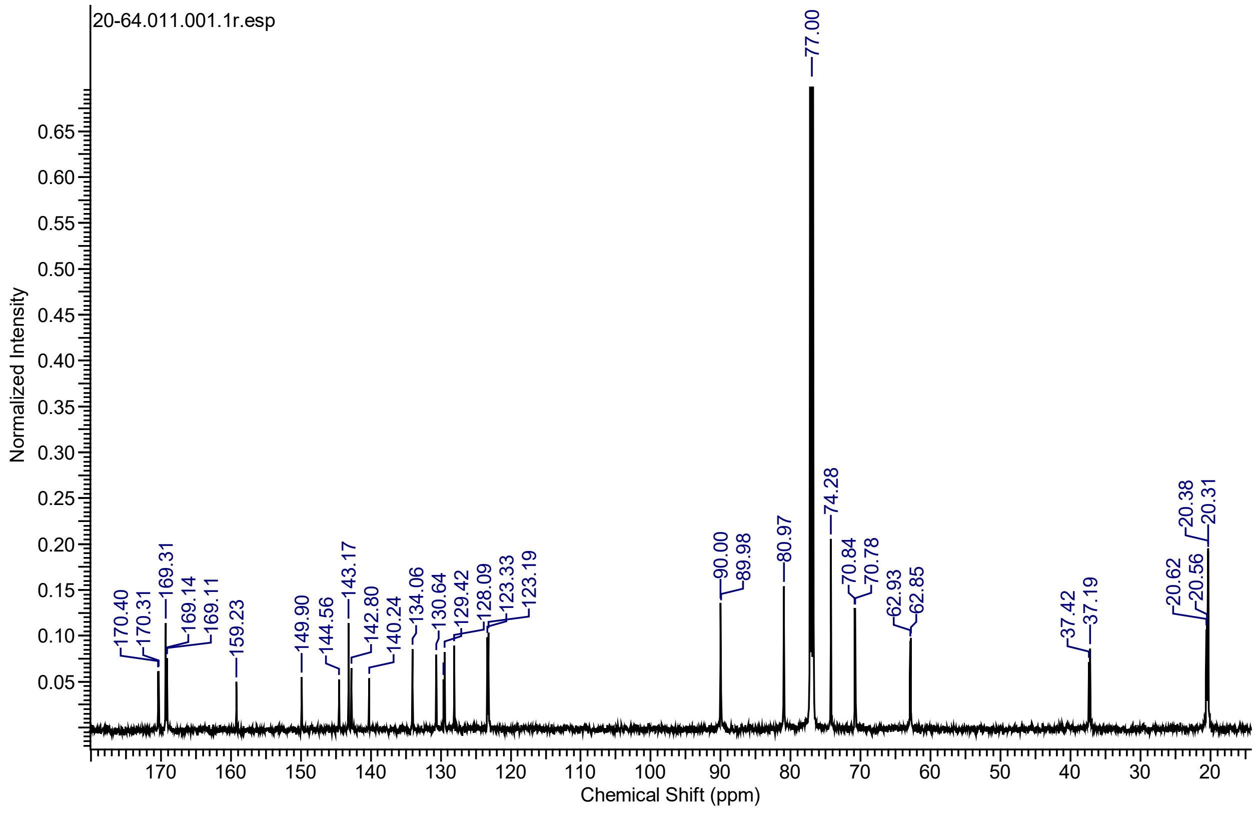

Figure S78. 1D ^{13}C-NMR spectrum of **5d** in $CDCl_3$ at T = 303K.

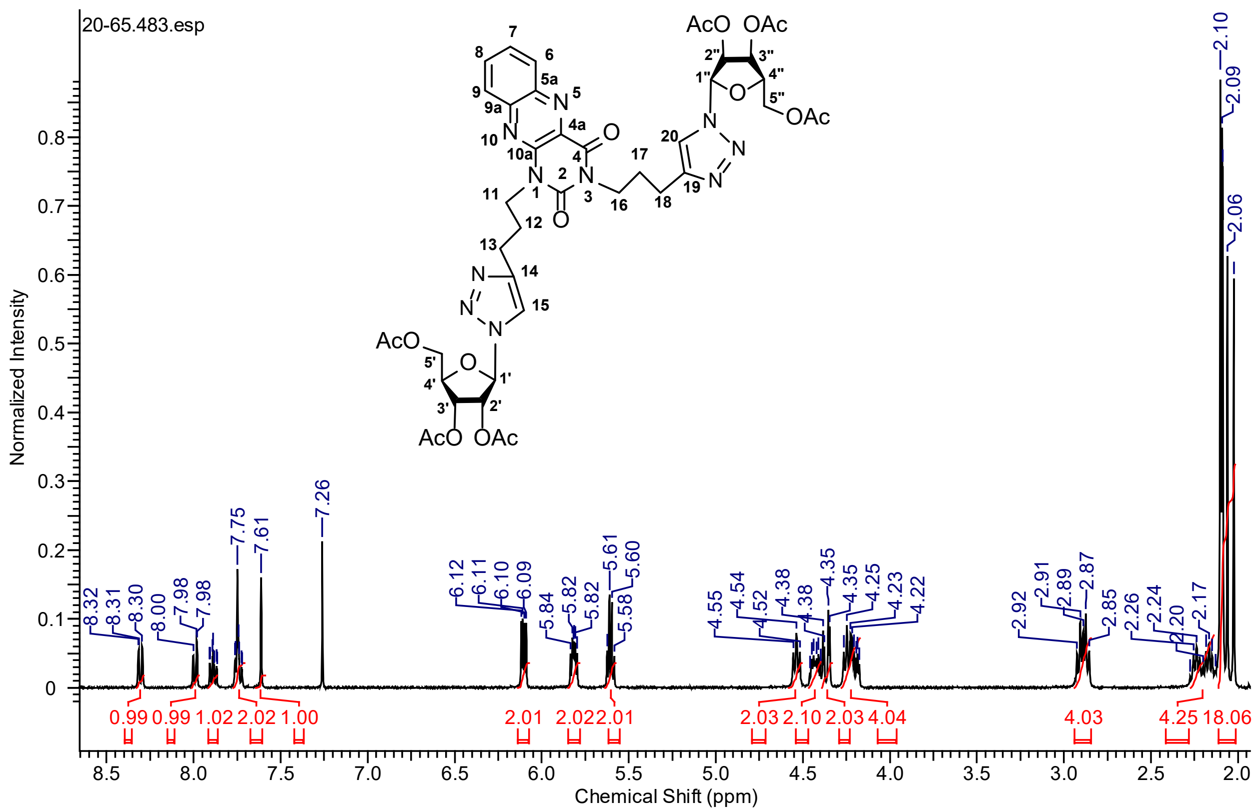

Figure S79. 1D ^{1}H-NMR spectrum of **5e** in $CDCl_3$ at T = 303K.

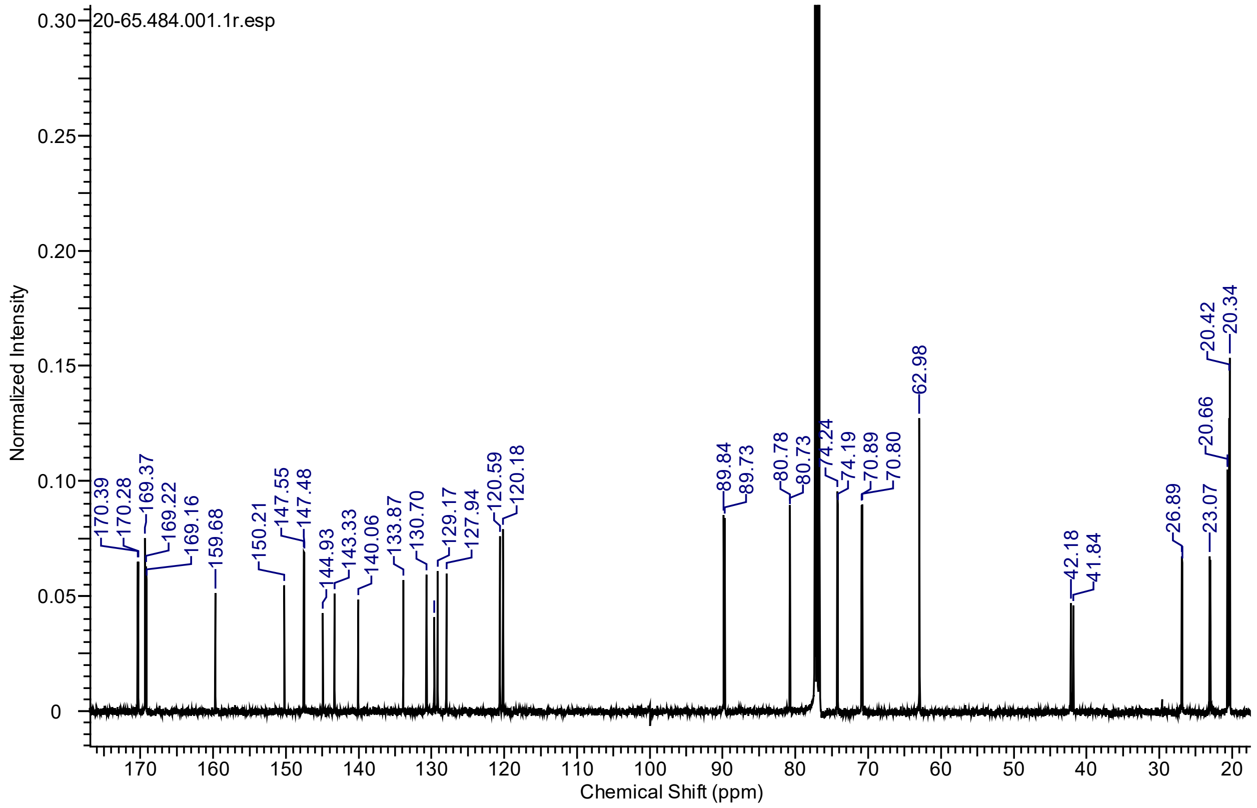

Figure S80. 1D ^{13}C-NMR spectrum of **5e** in $CDCl_3$ at T = 303K.

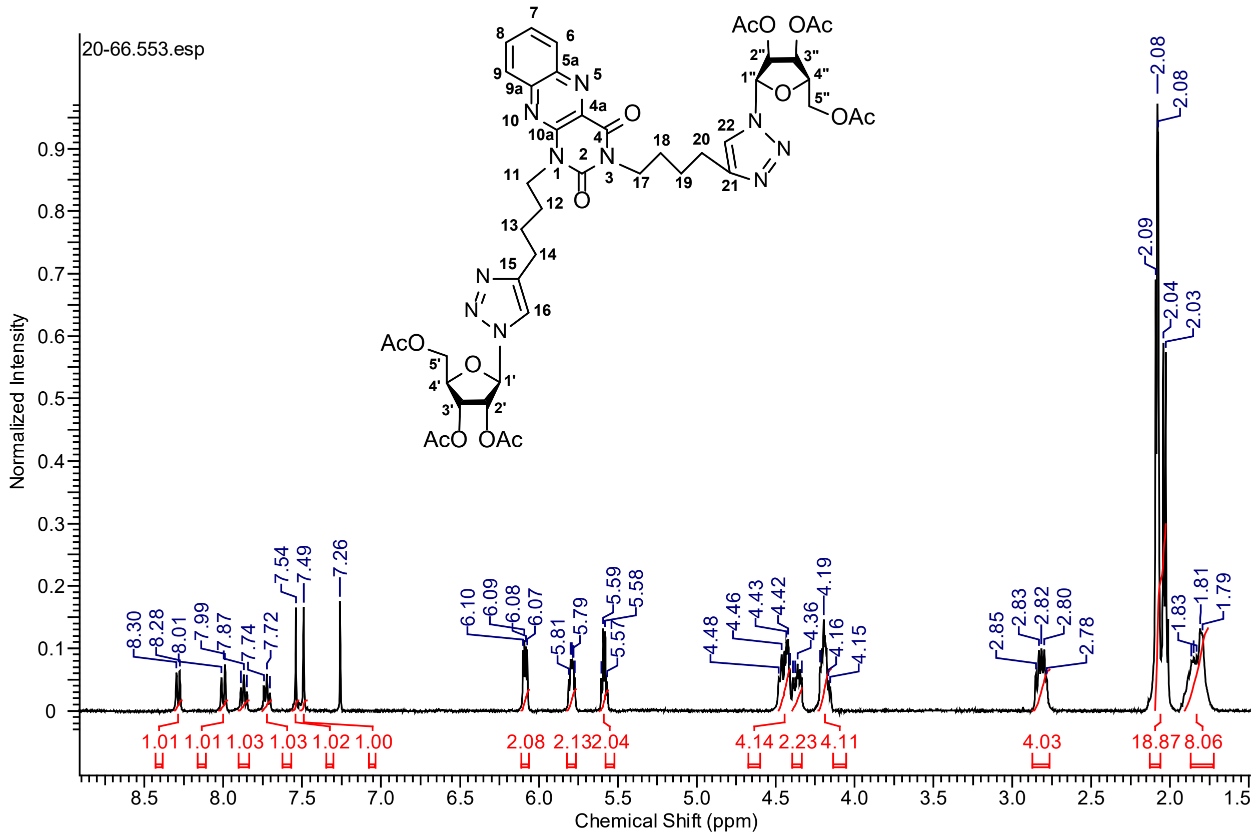

Figure S81. 1D ^{1}H-NMR spectrum of **5f** in $CDCl_3$ at T = 303K.

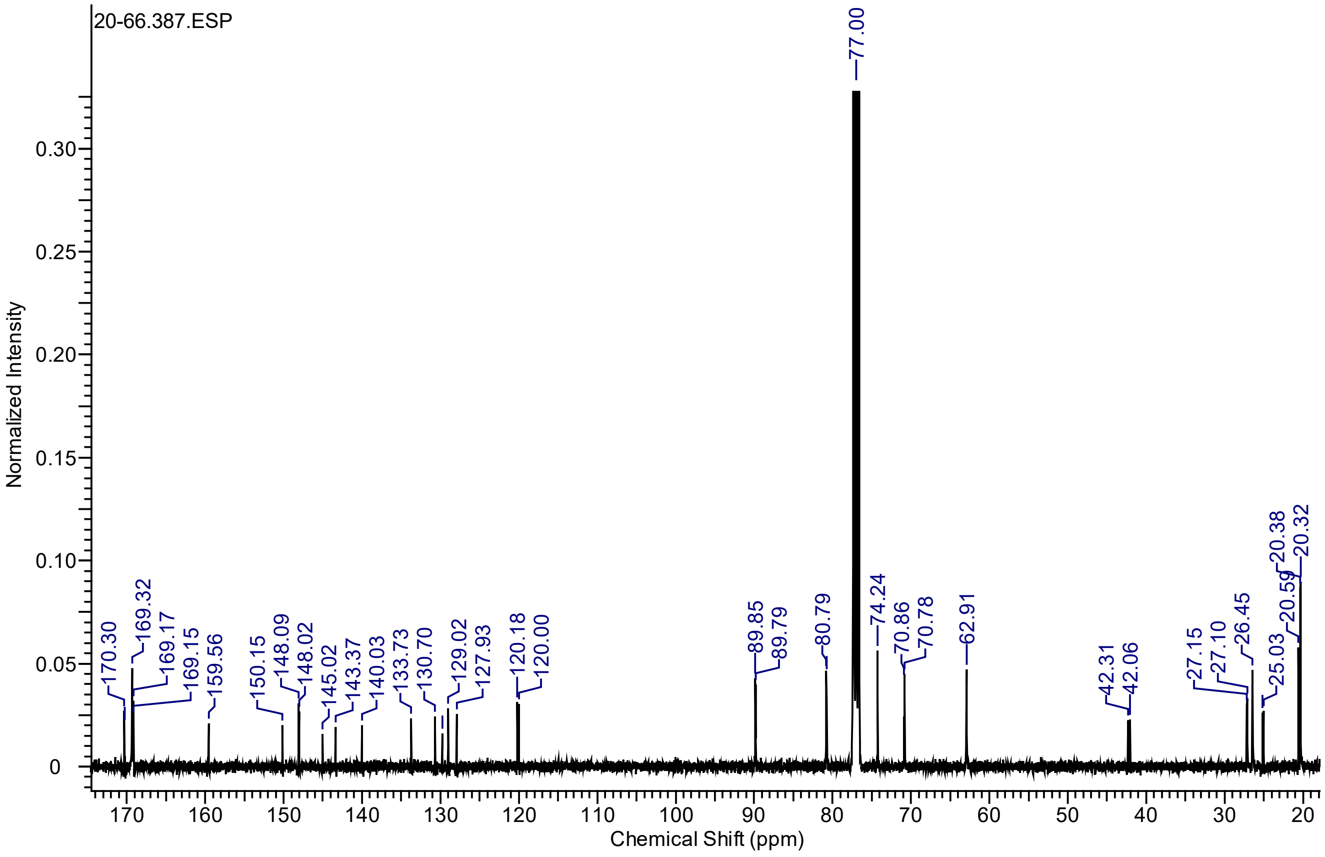

Figure S82. 1D ^{13}C-NMR spectrum of **5f** in $CDCl_3$ at T = 303K.

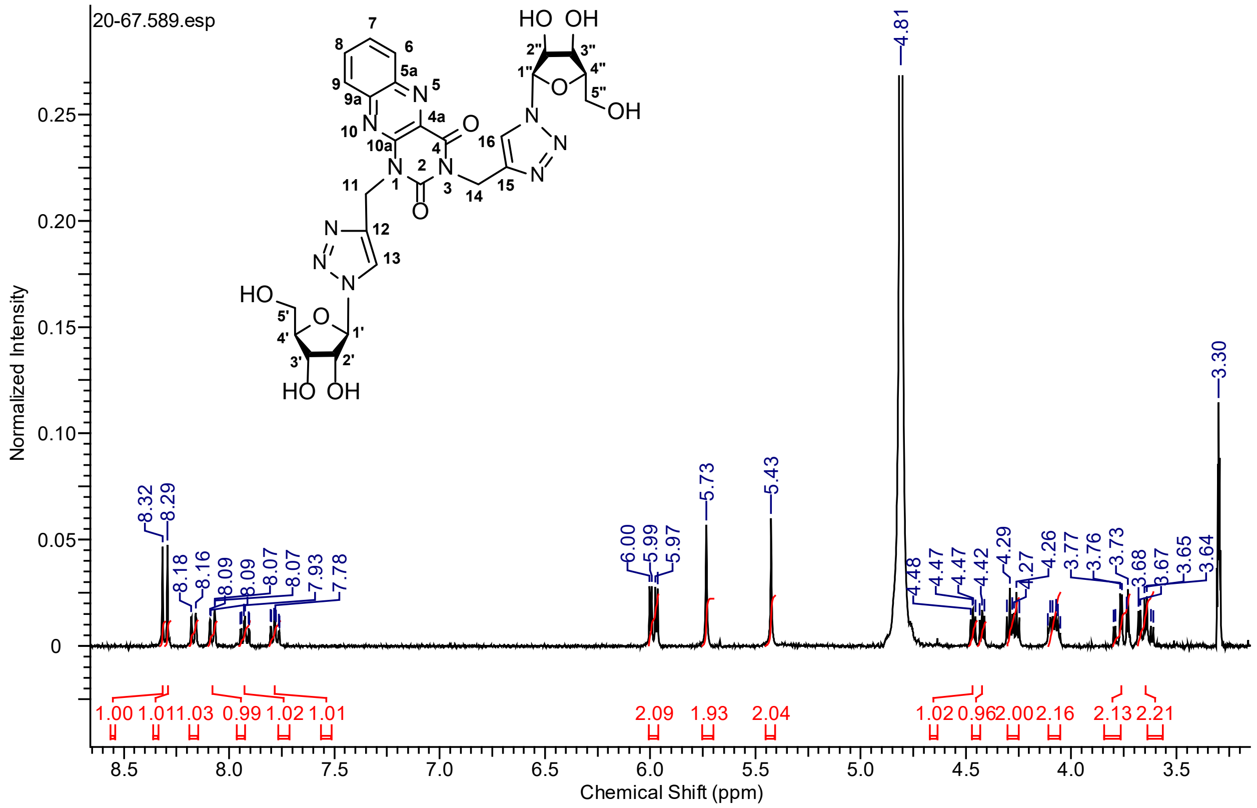

Figure S83. 1D ^{1}H-NMR spectrum of **5g** in CD_3OD at T = 303K.

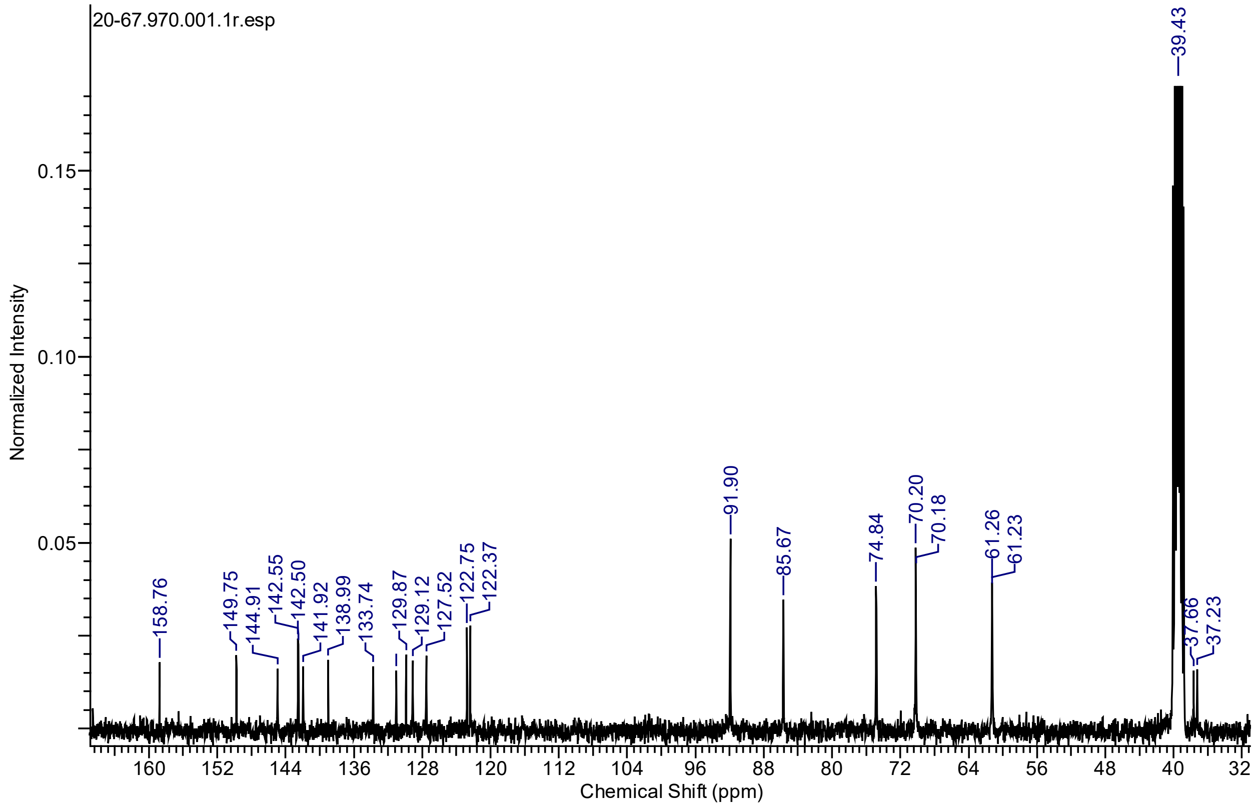

Figure S84. 1D ^{13}C-NMR spectrum of **5g** in DMSO-d_6 at T = 303K.

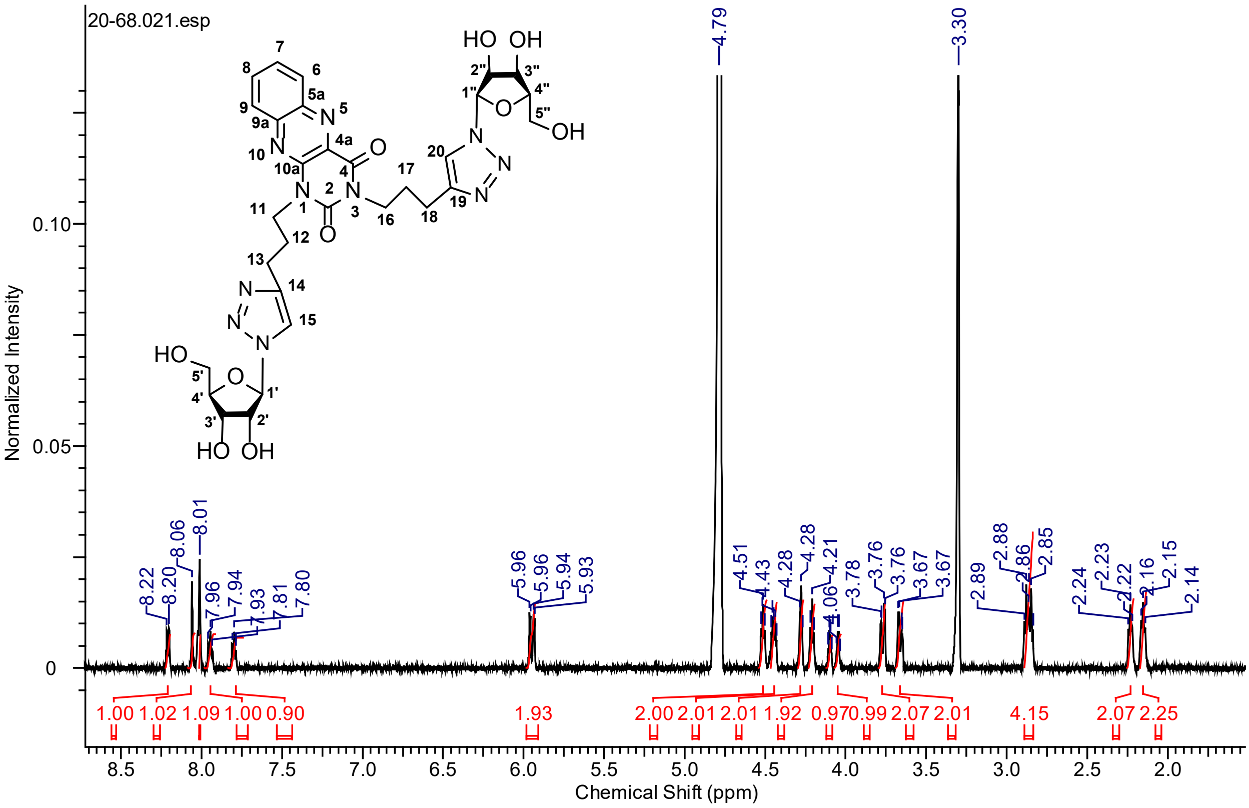

Figure S85. 1D ^{1}H-NMR spectrum of **5h** in CD_3OD at T = 303K.

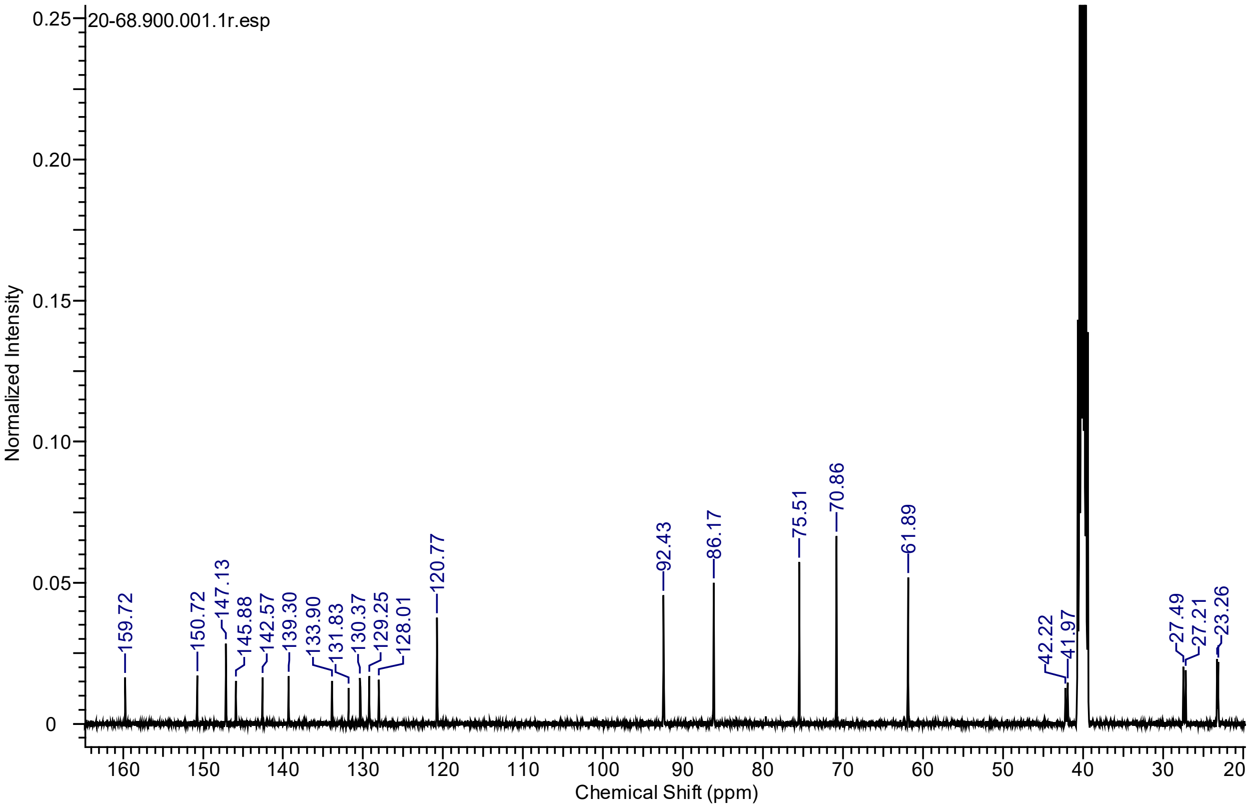

Figure S86. 1D ^{13}C-NMR spectrum of **5h** in DMSO-d_6 at T = 303K.

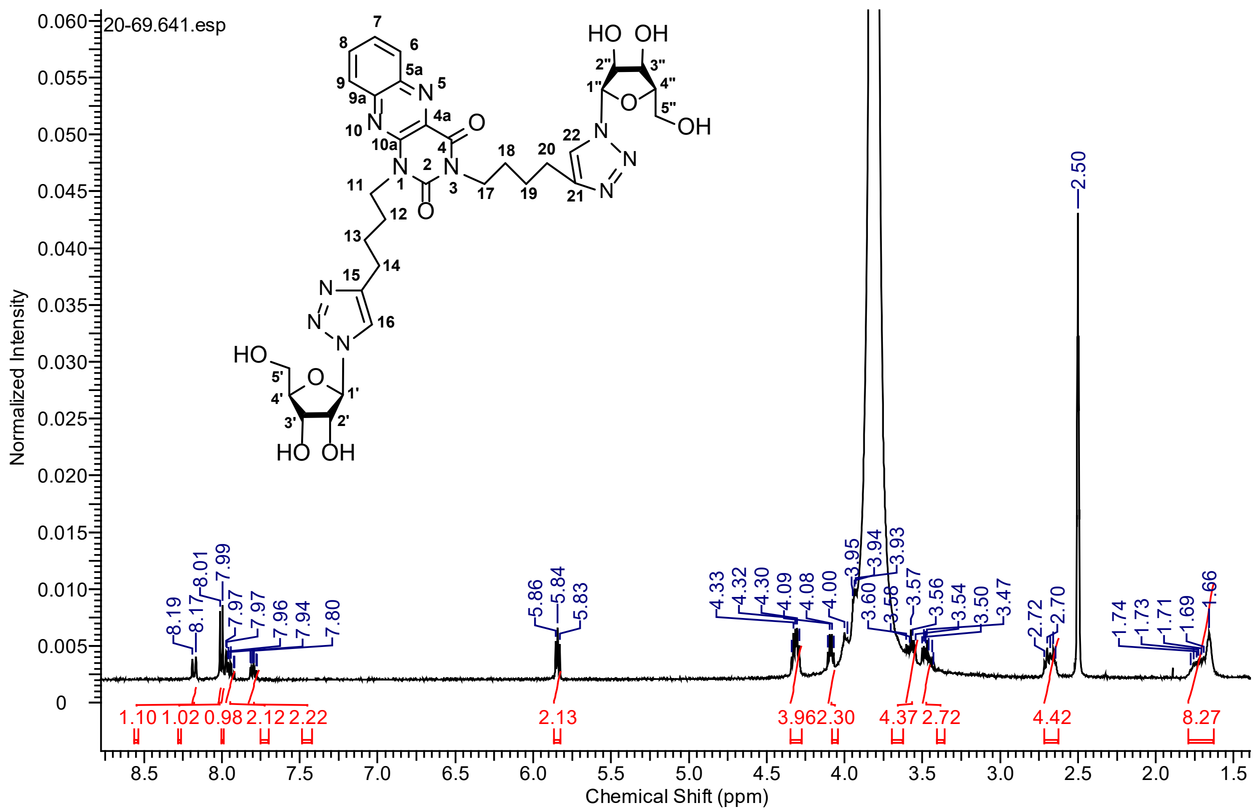

Figure S87. 1D ^{1}H-NMR spectrum of **5i** in CD_3OD at T = 303K.

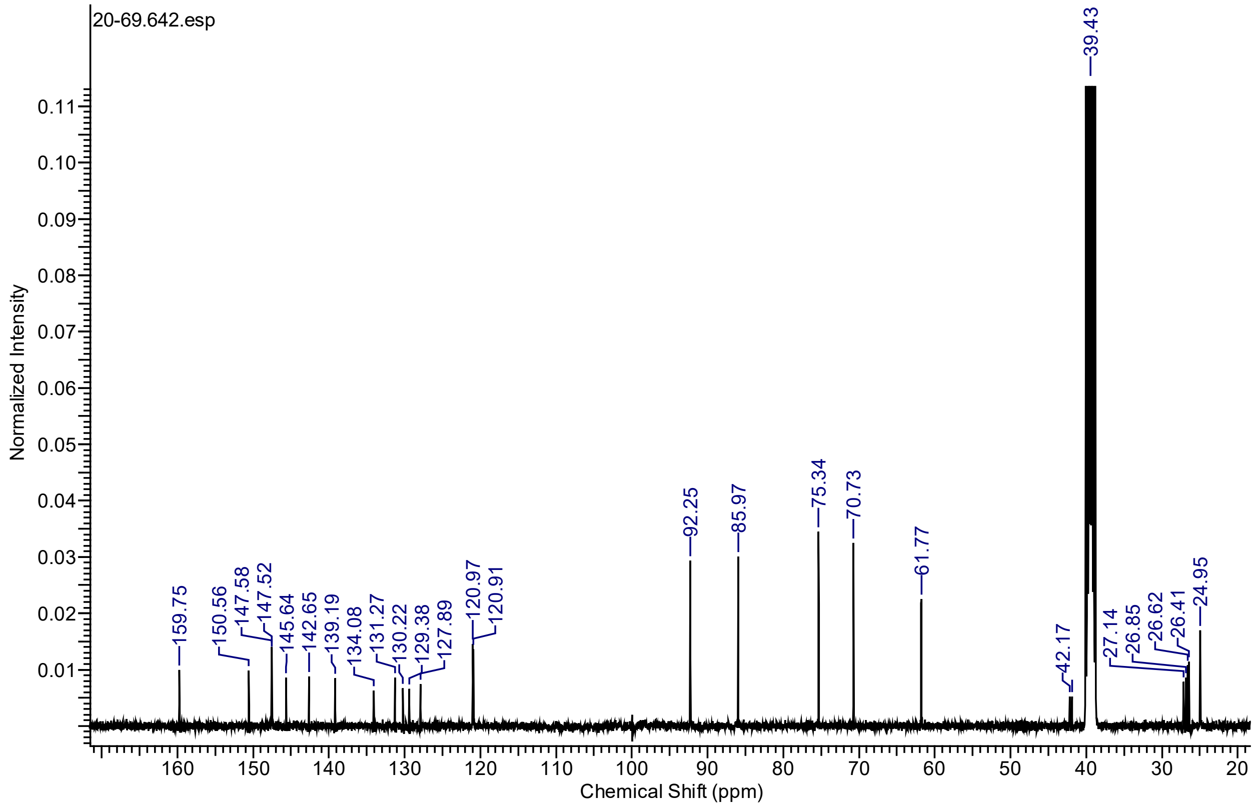

Figure S88. 1D ^{13}C-NMR spectrum of **5i** in DMSO-d_6 at T = 303K.

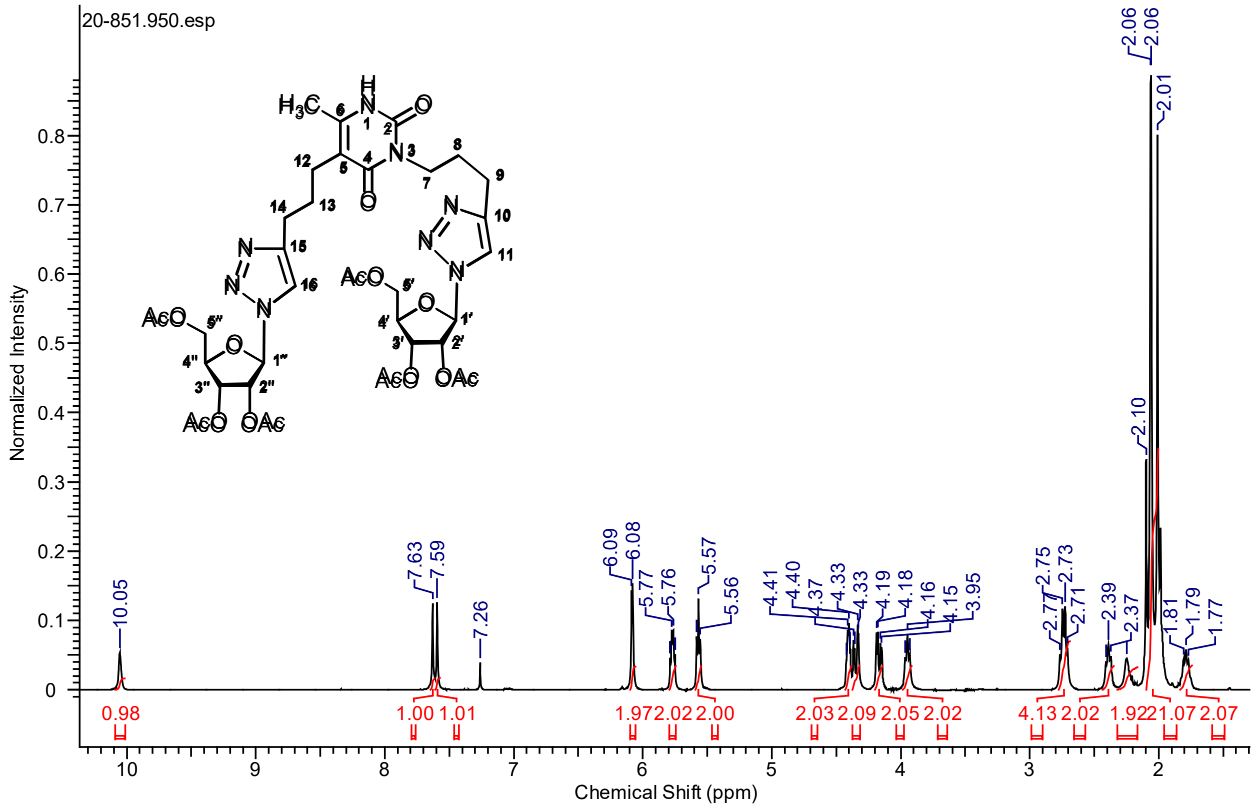

Figure S89. 1D ^{1}H-NMR spectrum of **11c** in $CDCl_3$ at T = 303K.

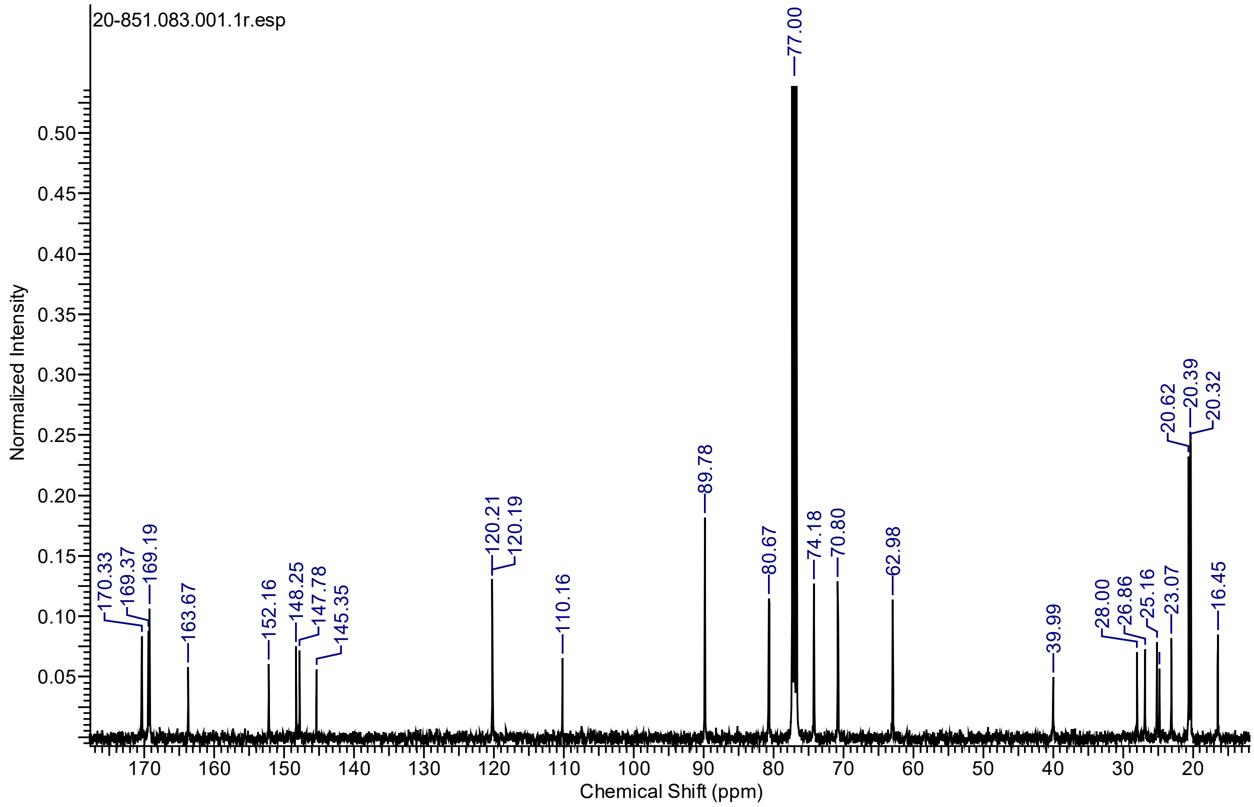

Figure S90. 1D ^{13}C-NMR spectrum of **11c** in $CDCl_3$ at T = 303K.

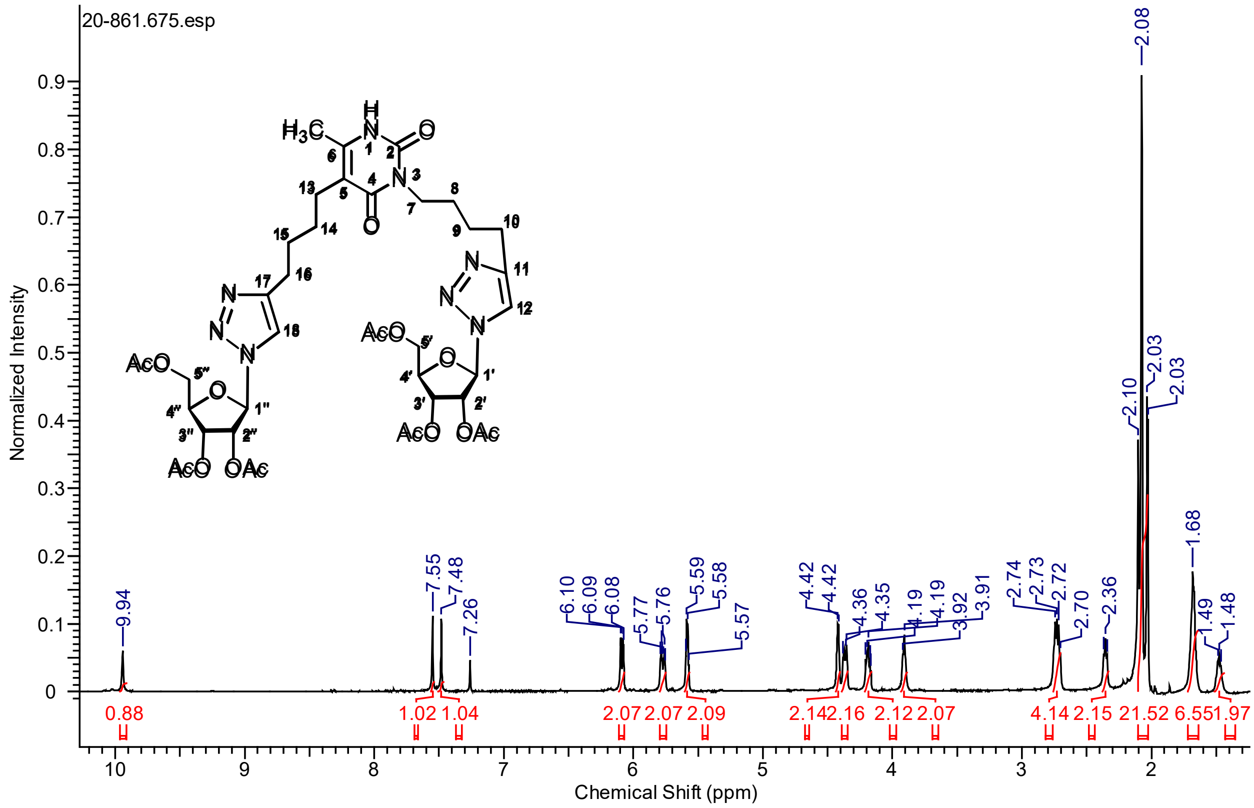

Figure S91. 1D ^{1}H-NMR spectrum of **11d** in $CDCl_3$ at T = 303K.

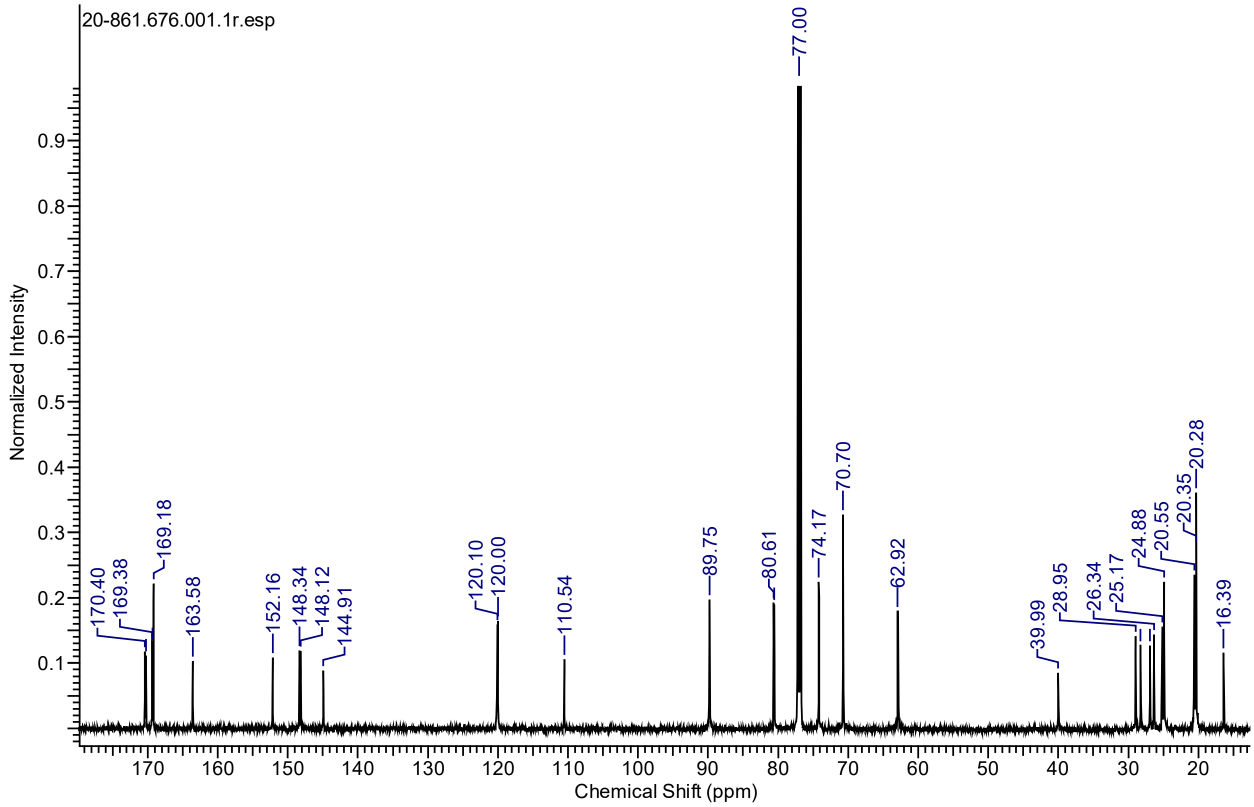

Figure S92. 1D ^{13}C-NMR spectrum of **11d** in $CDCl_3$ at T = 303K.

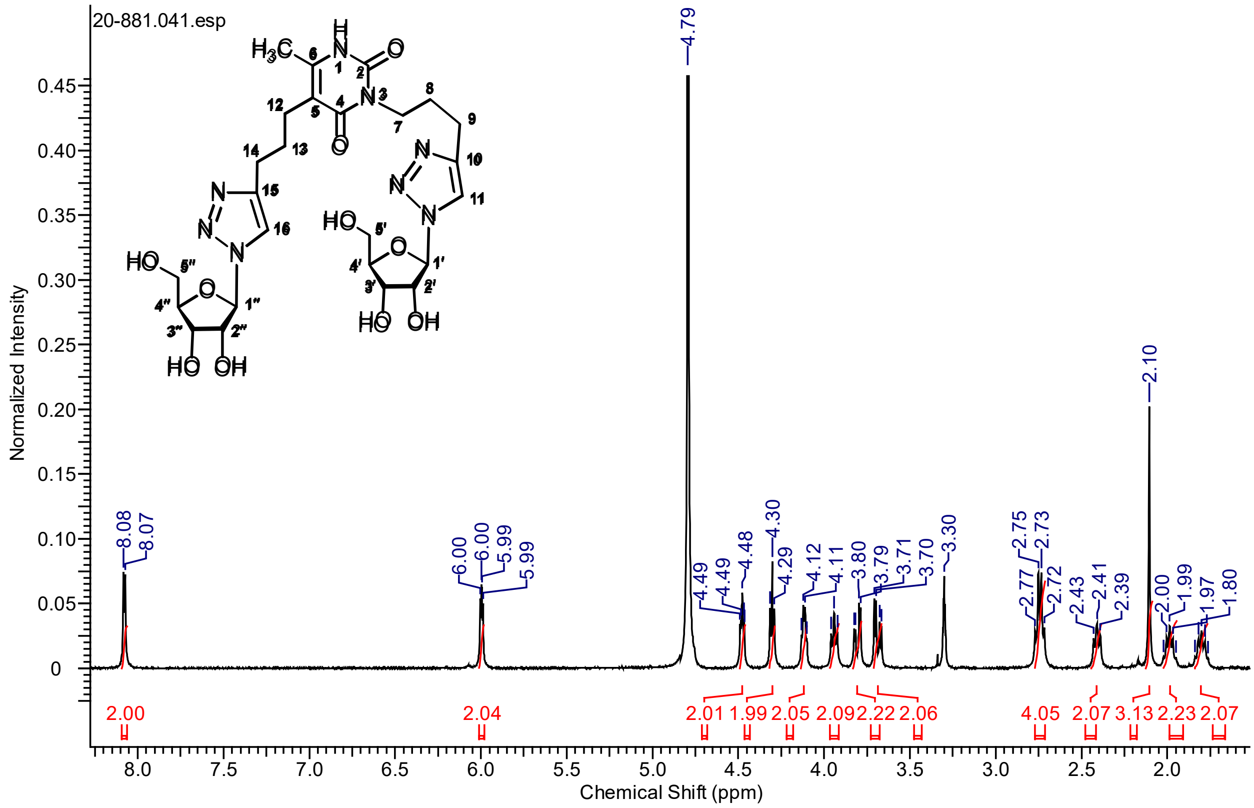

Figure S93. 1D ^{1}H-NMR spectrum of **11e** in CD_3OD at T = 303K.

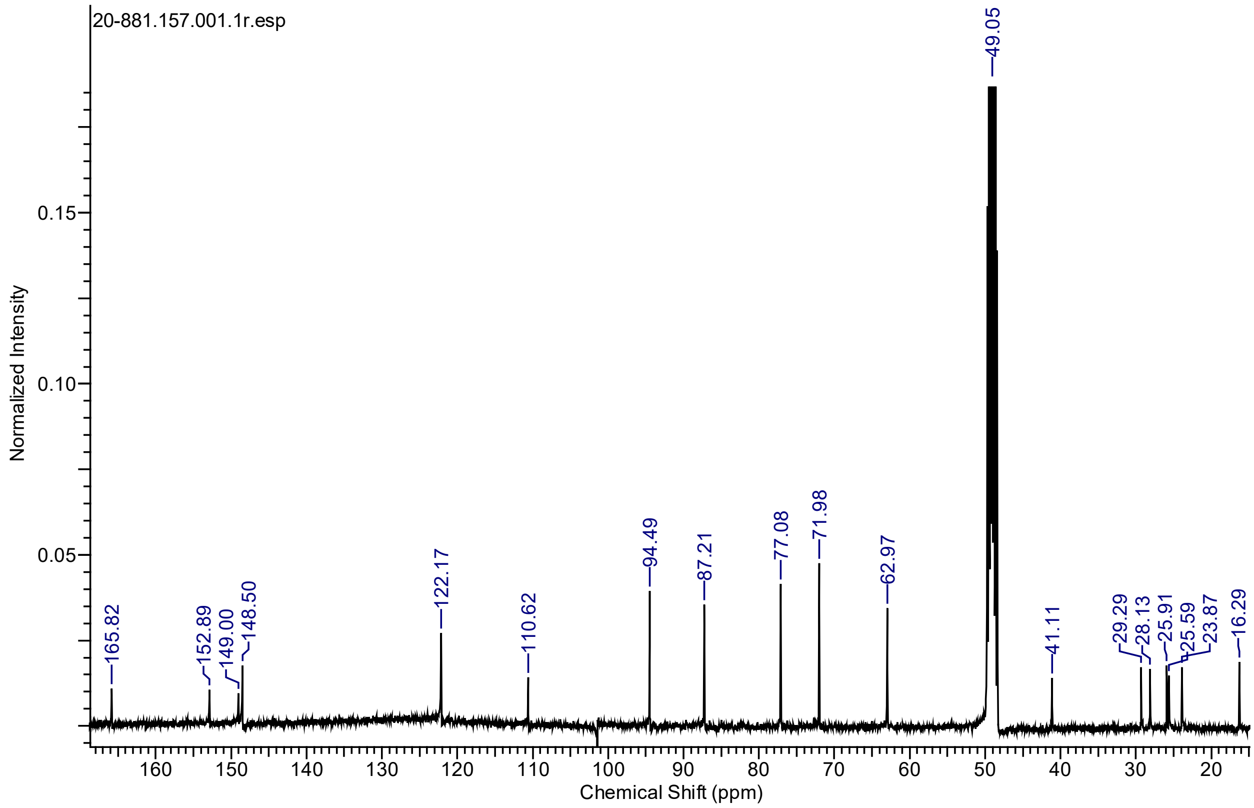

Figure S94. 1D ^{13}C-NMR spectrum of **11e** in CD_3OD at T = 303K.

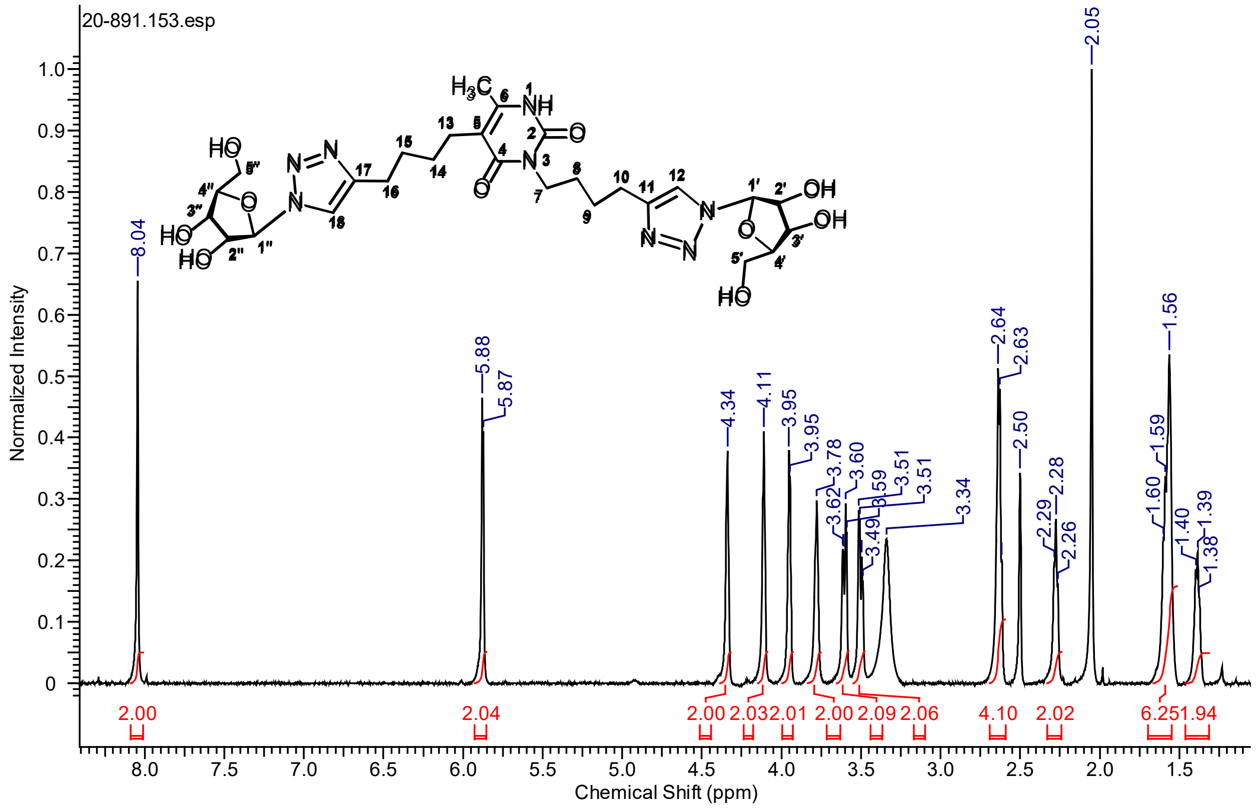

Figure S95. 1D ^{1}H-NMR spectrum of **11f** in DMSO-d_6 at T = 303K.

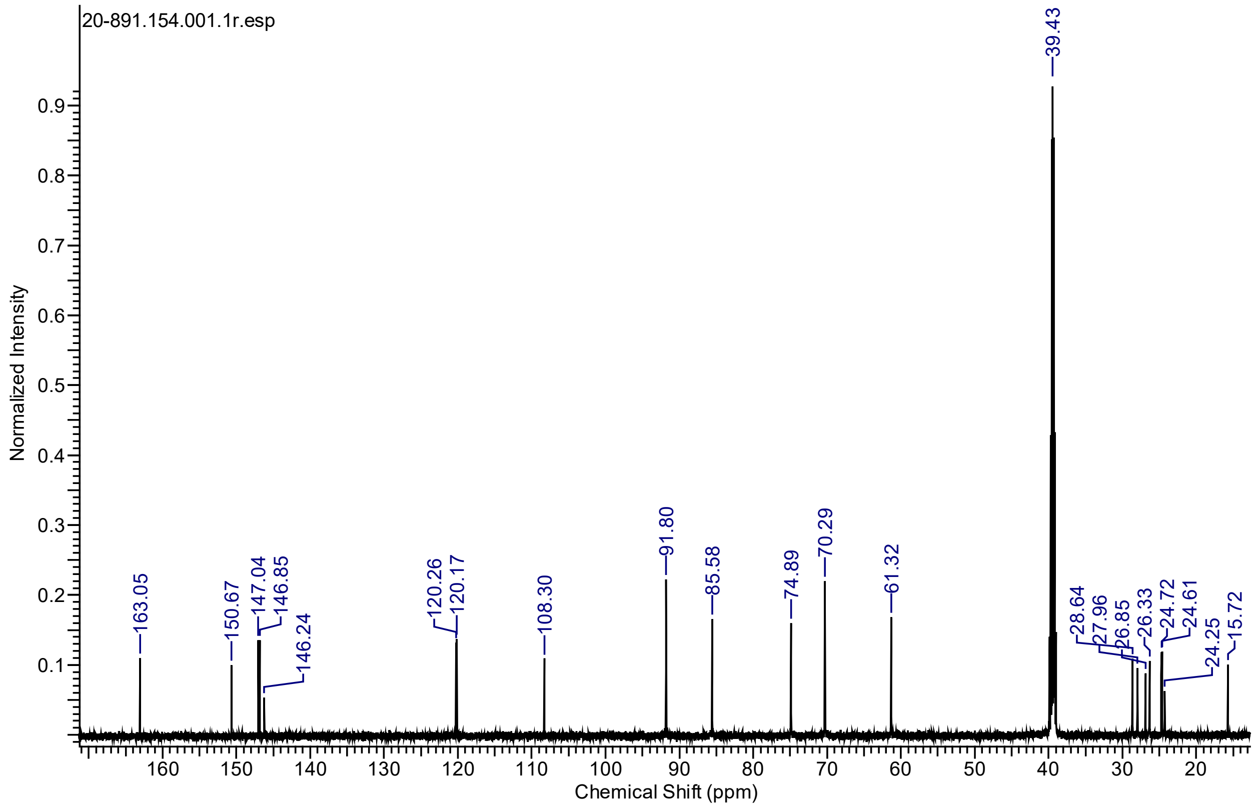

Figure S96. 1D ^{13}C-NMR spectrum of **11f** in DMSO-d_6 at T = 303K.

8. Antiviral assay

MDCK (ATCC CCL-34) and Vero (ATCC CCL-81) cells initially obtained from American Type Culture Collection (Rockville, MD, USA) were seeded into 96-well plates and incubated for 24 h at 36 °C at 5% CO_2 until confluent monolayer is formed. Three-fold dilutions (400–4 μg/mL) were prepared on Eagle's minimal essential medium (MEM) from the compounds under investigation, added to the cells and incubated for 24 h at 36 °C at 5% CO_2. The cell monolayer was washed twice with saline (0.9% NaCl) and 100 μL of MTT solution [3-(4,5-dimethylthiazole-2)-2,5-diphenyltetrazolium bromide], 0.5 μg/mL in MEM, were added into each well. The plates were incubated for 1 h at 36 °C, then the medium was removed and formazan pellets were dissolved in dimethyl sulfoxide (0.1 mL per well). The optical density in the wells was measured on a spectrophotometer Thermo Multiskan FC (Thermo Fisher Scientific Inc., Vantaa, Finland) at the wavelength of 540 nm. The results obtained were used for calculating the concentration of the compound resulting in death of 50% cells in the culture (CC_{50}) using GraphPad Prism software 6.01 employing the four-parameter logistic curve model. The values of CC_{50} were then converted from μg/mL to μM.

The compounds in appropriate concentrations were added to cells (0.1 mL per well). Cells were further infected with either A/Puerto Rico/8/34 (H1N1) influenza virus (for MDCK cells) or Coxsackie B3 virus (for Vero cells) (m.o.i 0,01 in both cases) and incubated for 48 hours at 36 °C at 5% CO_2. After that, cell viability was assessed by MTT test (see above). The cytoprotective activity of compounds was considered as their ability to increase the values of OD comparing to control wells (with virus only, no drugs). Based on the results obtained, the values of IC_{50}, i.e. concentration of compounds that result in 50% cells protection were calculated using GraphPad Prism software.

9. Cytotoxicity assay

Cytotoxic effects of compounds **2f**, **2i**, **5f**, **5i** on human cancer and normal cells were estimated by means of the multifunctional Cytell Cell Imaging system (GE Health Care Life Science, Sweden) using the Cell Viability Bio App 3.6.7.19 which precisely counts the number of cells and evaluates their viability from fluorescence intensity data [4]. Two fluorescent dyes that selectively penetrate the cell membranes and fluoresce at different wavelengths were used in the experiments. A low-molecular-weight 4′,6-diamidin-2-phenylindol dye (DAPI) is able to penetrate intact membranes of living cells and color nuclei in blue. High-molecular propidium iodide dye penetrates only dead cells with damaged membranes, staining them in yellow. As a result, living cells are painted in blue and dead cells are painted in yellow. DAPI and propidium iodide were purchased from Sigma. The WI-38 VA-13 cell culture, subline 2RA (human embryonic lung); M-HeLa clone 11 human, epithelioid cervical carcinoma, strain of HeLa, clone of M-HeLa; human duodenal cancer cell line (HuTu-80); PC-3 human Caucasian prostate adenocarcinoma from Type Culture Collection (ATCC, Manassas, VA, USA) were used in the experiments. The cells were cultured in a standard Eagle's nutrient medium manufactured at the Chumakov Institute of Poliomyelitis and Virus Encephalitis (PanEco company, Moscow, Russia) and supplemented with 10% fetal calf serum and 1% nonessential amino acids. The cells were plated into a 96-well plate (Eppendorf) at a concentration of 1×10^5 cells/ml, 150 μl of medium per well, and cultured in a CO_2 incubator at 37 °C. Twenty four hours after seeding the cells into wells, the compound under study was added at a preset dilution, 150 μl to each well. The dilutions of the compounds were prepared immediately in nutrient media; 5% DMSO that does not induce the inhibition of cells at this concentration was added for better solubility. The experiments were repeated three times. Intact cells cultured in parallel with experimental cells were used as a control.

10. Molecular Docking Study

Molecular docking was carried out using the Autodock 4.2 Vina software and AutoDock Tools (ADT 1.5.6) [5]. The three-dimensional (3D) crystal structure of *N*-terminal endonuclease domain of polymerase acidic protein (PA) of RNA-dependent RNA polymerase (PDB code 5I13) [6] and Coxsackievirus B3 coat protein (PDB code 1COV) [7] was obtained from the RCSB Protein Data Bank [8]. The standard 3D structures of **2f**, **2i**, **2i-TP**, **5f**, **5i**, **5i-TP**, **11c** were constructed using the HyperChem 8.0 [9] and converted into a pdb file by Open Babel [10]. The cubic grid box of 22 × 20 × 20 Å (x, y, z) with a spacing of 1.000 Å and grid maps were generated. The docking parameters were used as the default settings. The enzyme-ligand interactions were detected using ADT 1.5.6 and have been presented as 2D diagrams.

11. References

1. Andreeva, O.V.; Belenok, M.G.; Saifina, L.F.; Shulaeva, M.M.; Dobrynin, A.B.; Sharipova, R.R.; Voloshina, A.D.; Saifina, A.F., Gubaidullin, A.T.; Khairutdinov, B.I.; Zuev, Y.F.; Semenov, V.E.; Kataev, V.E. Synthesis of novel 1,2,3-triazolyl nucleoside analogues bearing uracil, 6-methyluracil, 3,6-dimethyluracil, thymine, and quinazoline-2,4-dione moieties. *Tetrahedron Lett.* **2019**, *60*, 151276
2. Sharipova, R.R.; Saifina, L.F.; Belenok, M.G.; Semenov, V.E.; Kataev, V.E. First analog of pyrimidine nucleosides with two D-ribofuranose residues. *Russ. J. Org. Chem.* **2020**, *56*, 181-184.
3. Andreeva, O.V.; Garifullin, B.F.; Zarubaev, V.V.; Slita, A.V.; Yesaulkova, I.L.;·Saifina, L.F.; Shulaeva, M.M.; Belenok, M.G.; Semenov, V.E.; Kataev, V.E. Synthesis of 1,2,3‑triazolyl nucleoside analogues and their antiviral activity. *Mol. Diversity* **2020**, *25*, 473-490.
4. Voloshina, A.D.; Sapunova, A.S.; Kulik, N.V.; Belenok, M.G.; Strobykina, I.Yu.; Lyubina, A.P.; Gumerova S.K.; Kataev, V.E. Antimicrobial and cytotoxic effects of ammonium derivatives of diterpenoids steviol and isosteviol. *Bioorg. Med. Chem.* **2021**, *32*, 115974.
5. Trott, O.; Olson, A.J. AutoDock Vina: improving the speed and accuracy of docking with a new scoring function, efficient optimization and multithreading. *J. Comput. Chem.* **2010**, *31*, 455–461.
6. Fudo S., Yamamoto N., Nukaga M., Odagiri T., Tashiro M., Hoshino T. Two Distinctive Binding Modes of Endonuclease Inhibitors to the N-Terminal Region of Influenza Virus Polymerase Acidic Subunit. Biochemistry **2016**, *55*, 2646–2660.
7. Muckelbauer J. K. , Kremer M., Minor I., Tong L., Zlotnick A., Johnson J. E., Rossmann M. G. Structure determination of coxsackievirus B3 to 3.5 Å resolution. Acta Cryst. **1995**, *D51*, 871–887.
8. Bermsn, H.M.; Westbrook, J.; Feng, Z.; Gilliland, G.; Bhat, T.N.; Weissig, H.; Shindyalov, I.N.; Bourne, P.E. The protein data bank. *Nucleic Acids Research* **2000**, *28*, 235–242.
9. HyperChem Professional 8.0 (2007). Hypercube, Inc. http://www.hyper .com/?tabid =360. Accessed 14 Sept 2020
10. O'Boyle, N.M.; Banck, M.; James, C.A.; Morley, C.; Vandermeersch, T.; Hutchison, G.R. Open babel: An open chemical toolbox. *J. Cheminform* **2011**, *3*, 33.
